# Arylpiperazinyl-Benzocycloheptapyrrole-Carboxamides Endowed with Dual Anticancer and Antiviral Activities

**DOI:** 10.3390/molecules30204052

**Published:** 2025-10-11

**Authors:** Gabriele Murineddu, Carlo Di Marzo, Paola Corona, Silvia Coinu, Erika Plicanti, Battistina Asproni, Sandra Piras, Giulia Freer, Antonio Carta

**Affiliations:** 1 Department of Medicine, Surgery and Pharmacy, University of Sassari, 07100 Sassari, Italy; pcorona@uniss.it (P.C.); s.coinu1@phd.uniss.it (S.C.); asproni@uniss.it (B.A.); piras@uniss.it (S.P.); 2 Centro Retrovirus, Department of Translational Research, University of Pisa, 56127 Pisa, Italy; dimarzo.carlo@virgilio.it (C.D.M.); e.plicanti@studenti.unipi.it (E.P.); giulia.freer@med.unipi.it (G.F.)

**Keywords:** tricyclic pyrroles, anticancer, antiviral, microwave irradiation

## Abstract

In this work, we synthesized a small library of tricyclic compounds to assess whether they might have both anticancer and antiviral activity against three viruses that have recently caused epidemics. Concerning their anti-tumour activity, derivative **1** was found to be the compound with the highest GI_50_ values on some cancer cell line panels. Particularly, in melanoma cell lines, its GI_50_ values ranged between 1.54 μM (MALME-3M) and 2.03 μM (M14). Several derivatives with considerable anti-tumour activity showed antiviral activity as well, against influenza A virus (e.g., derivative **19**, selectivity index of 21.36 in MDCK cells) or against Zika virus (compound **13**, selectivity index of 20.20 in Huh-7 cells). Moreover, compounds **13** and **12** showed anti-SARS-CoV-2 activity, with selectivity indices of 150.00 and 63.63, respectively. Compound **1**, for its anticancer activity, and **13**, for its anti-SARS-CoV-2 activity, together with the compounds active against Zika virus and influenza A virus, are promising candidates for further studies.

## 1. Introduction

Cancer and viral infections are serious global health problems due to the great number of deaths they cause [1]. Vector-borne viruses, transmitted by mosquitoes or other arthropods, are an example of emerging public health threats: globalization, climate change, and urbanization contribute to the widening distribution of competent vectors, facilitating the circulation of arboviruses [2]. At the same time, modern patterns of mobility and trade accelerate the international spread of pathogens, transforming local outbreaks into global health emergencies. Thus, from influenza pandemics in the early 1900s [3] to more recent ones, such as the spread of Zika virus (ZIKV) [4] and the unprecedented COVID-19 crisis, it has become increasingly evident that viral infections are not isolated medical problems but global concerns [5]. Viral replication and carcinogenic processes share similar biological mechanisms: they both hijack the host cell machinery. In addition, immunosuppressed patients are prone to both cancer and viral infection. Therefore, research on compounds with both anticancer and antiviral mechanisms is likely to yield safer, more effective, and versatile therapies that can be exploited in the clinics.

**Figure 1:**
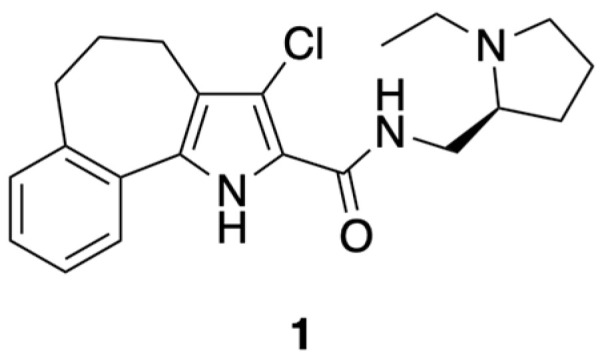
(*S*)-3-Chloro-*N*-(1-ethyl-2-pyrrolidinylmethyl)-5,6-dihydro-*4H*-benzo[6,7]cyclohepta[1,2-*b*]pyrrol-2-carboxamide **1**.

As part of a research project aimed at identifying new potential chemotherapeutic agents, we synthesized new tricyclic heteroaromatic systems and evaluated their cytotoxicity in vitro against nine different NCI cancer cell lines. Among these new templates, the benzocycloheptapyrrole-carboxamide **1** (Figure 1) proved to be the most promising, showing significant inhibition percentages across different cell lines, as reported below.

**Figure 2:**
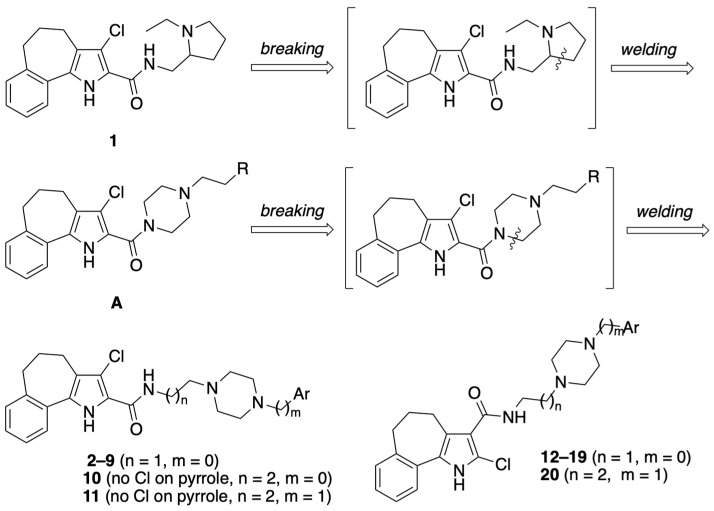
Design of analogues **A**, carboxamides **2**–**11**, and isomers **12**–**20**.

To improve the efficacy of the compound, we aimed at investigating the effect of conformational stiffening of the *N*-ethyl-pyrrolidinmethylamine system of the carboxamide portion of **1**, achievable by virtual breaking of the C2–C3 bond of the pyrrolidine ring and simultaneous welding of the methylene carbon of the ethyl residue with amide nitrogen, which led to the synthesis of a series of derivatives with general structure **A** (Figure 2). Preliminary biological data suggest that this modification allowed it to maintain considerable anticancer activity, although lower compared to derivative **1**.

To further evaluate the structural features responsible for the cytotoxic activity against tumour cell lines, carboxamides **2**–**9** have been designed again by hypothetical splitting and welding processes involving the piperazine portion of **A** (Figure 2). A first break in the N1-C2 bond of the piperazine ring of **A**, followed by welding between the methylene terminal residue of the ethyl chain and the ethylamine bioisostere of the propyl chain, would lead to carboxamides **2**–**9** and their structural non-halogenated derivatives **10** and **11**. Finally, the effect of the Cl/carboxamide shift on the pyrrole ring was studied by synthesizing isomers **12**–**20** (Figure 2).

Due to their considerable activity against several cancer cell lines, we also evaluated their antiviral activity.

In this work, we report the synthesis and preliminary biological data of compounds listed in Table 1.

## 2. Results

### 2.1. Synthesis of Benzo[6,7]cyclohepta[1,2-b]pyrrole-3-carboxamides (***1**–**11***) and isomers 3-chloro-2-carboxamides (***12**–**20***)

**Scheme 1:**
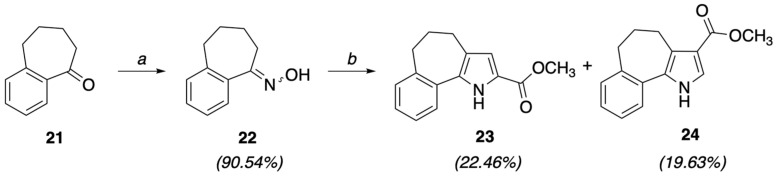
Reagents and conditions: (**a**) NaOAc·3H_2_O, NH_2_OH·HCl, EtOH, H_2_O, 4 h, rfx; (**b**) methyl propiolate, DABCO, toluene, MW (abs. normal) 80 °C, 10 min, then MW (abs. normal) 170 °C, 10 min. The numbers in parentheses represents the reaction yields.

The synthetic route to obtain esters **23** and **24**, key intermediates for the synthesis of the two sets of amides **1**–**11** and **12**–**20**, is reported in Scheme 1. Commercial benzosuberone (**21**) was reacted with NH_2_OH and NaOAc in EtOH and H_2_O to obtain the oxime **22**. Then, it was reacted with methyl propiolate in toluene using 1,4-diazabicyclo[2.2.2]octane (DABCO) as a catalyst. The reaction involved two consecutive steps of microwave irradiation: in the former, the mixture was subjected to an irradiation cycle at 80 °C for 10 min with normal absorption to provide the acrylate adduct (not reported in the scheme), which was isolated and characterized only once. Then the acrylate intermediate was irradiated for another 10 min with normal absorption at 170 °C; after flash chromatography purification, esters **23** and **24** were isolated.

The structures of the two esters were determined unequivocally. Previously, we reported the synthesis of **24** by thermal cyclization of *O*-vinyl oximes [6]. Moreover, by a different synthetic route, we synthesized its α-chloroester analogue by cyclization of a cyano-keto ester intermediate with gaseous HCl and final dehalogenation with HCOONH_4_ in the presence of Pd/C 10% [7]. The resulting compound showed the same spectroscopic and analytical data as **24**, confirming the β-position of the ester group. Therefore, analyzing the spectra of compound **23**, we confirmed its structure as the α-isomer of ester **24**. To further confirm the two structures, a two-dimensional NOE spectroscopy (NOESY) experiment was carried out (Figures S1 and S2 in the Supplementary Materials). In ester **24**, the doublet at 7.50 ppm (with a *J*_o_ = 3.2 Hz), corresponding to the hydrogen on the α-position of the pyrrole ring, shows a correlation peak with the pyrrole NH at 8.59 ppm. In isomer **23**, the signal for the hydrogen on the β-position appears at 7.50 ppm (with a *J*_m_ = 2.8 Hz) and does not correlate with the pyrrole NH.

**Scheme 2:**
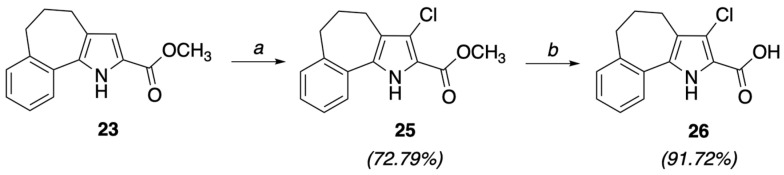
Reagents and conditions: (**a**) NCS, CHCl_3_, 3 h, rfx; (**b**) NaOH_aq_ 10% (EtOH/H_2_O, 2/1), 12 h, rfx. The numbers in parentheses represent the reaction yields.

**Scheme 3:**
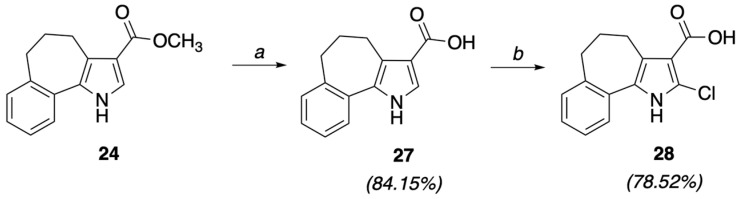
Reagents and conditions: (**a**) NaOH_aq_ 20%, CH_3_OH, 9 h, rfx; (**b**) NCS, CHCl_3_, 3 h, rfx. The numbers in parentheses represent the reaction yields.

Esters underwent hydrolysis and functionalization as reported in Scheme 2 and Scheme 3. β-Chloroacid **26** (Scheme 2) was synthesized by an initial chlorination of ester **23** with N-chlorosuccinimide (NCS) to chloro-ester **25**, then saponification with 10% aqueous NaOH to provide the expected acid **26**.

Isomer **28** was obtained as shown in Scheme 3 by inversion of functionalization of the pyrrole moiety of the tricyclic ester **24**, via intermediate **27**, compared to Scheme 2.

**Scheme 4:**
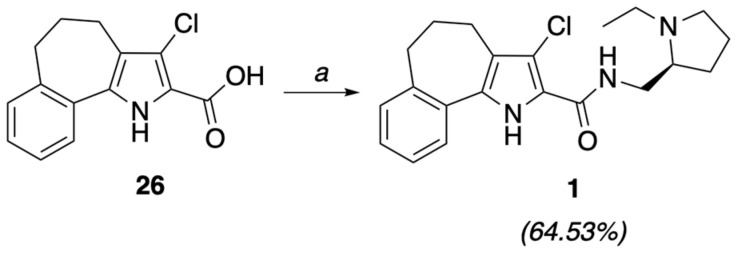
Reagents and conditions: (**a**) CDI, HOBt, CH_2_Cl_2_, 1.5 h, r.t., then (*S*)-(−)-2-(aminomethyl)-1-ethylpyrrolidine, 12 h, r.t. The numbers in parentheses represent the reaction yields.

The carboxylic acid **26** served as the starting product for the synthesis of carboxamides **1**–**9**. The reaction of **26** with 1,1′-carbonyldiimidazole (CDI), 1-hydroxybenzotriazole (HOBt), and (*S*)-(−)-2-(aminomethyl)-1-ethylpyrrolidine gave compound **1** (Scheme 4).

**Scheme 5:**
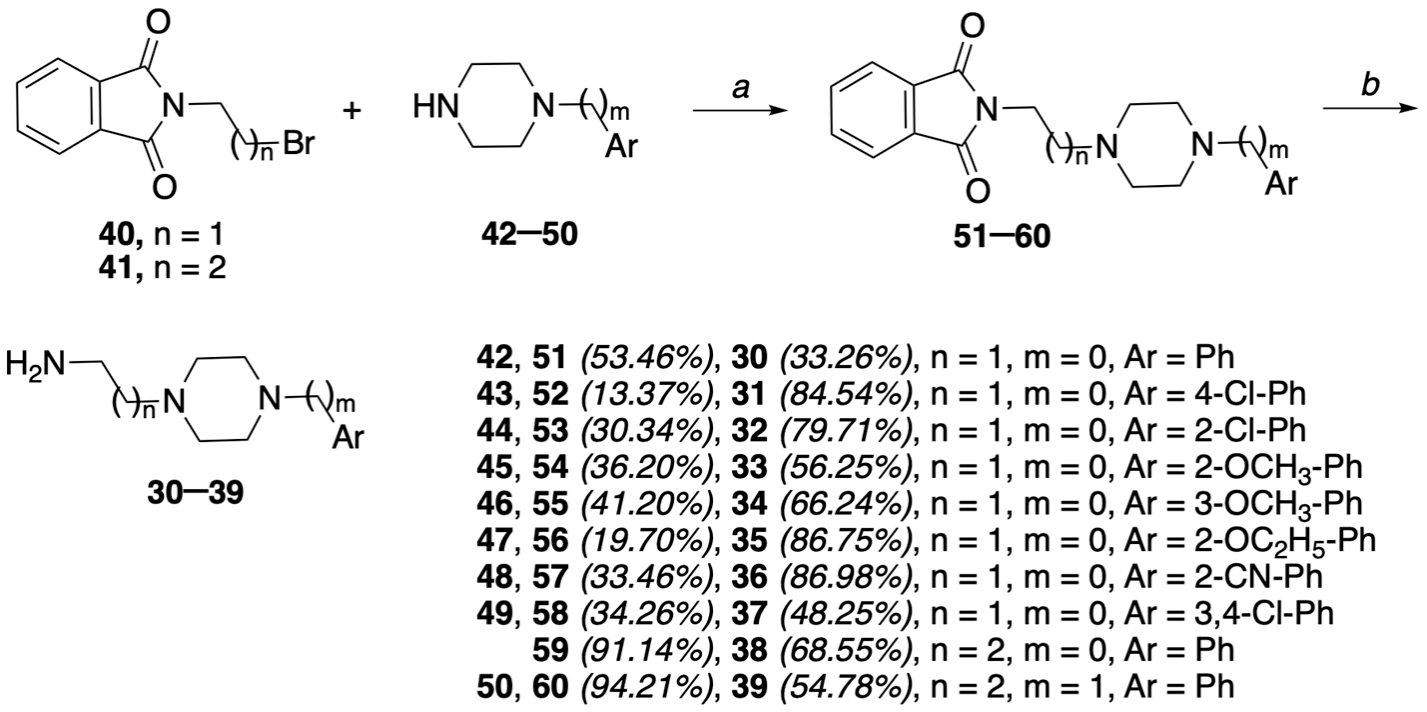
Reagents and conditions: (**a**) K_2_CO_3,_ DMF, MW (abs. normal), 55 °C, 10 min; (**b**) EtOH, NH_2_NH_2_·H_2_O, MW (abs. low), 80 °C, 20 min. The numbers in parentheses represent the reaction yields.

The synthesis of arylpiperazinyl-alkylamines **30**–**39** is reported in Scheme 5. Nucleophilic substitution between *N*-(2-bromoethyl)phthalimide **40** or *N*-(3-bromoproyl)phthalimide **41** and the appropriate aryl-piperazine (**42**–**50**) in the presence of K_2_CO_3_ led to the corresponding isoindoline-1,3-dione derivatives **51**–**60**. Microwave-assisted Gabriel’s reaction provided the desired amines **30**–**39**.

**Scheme 6:**
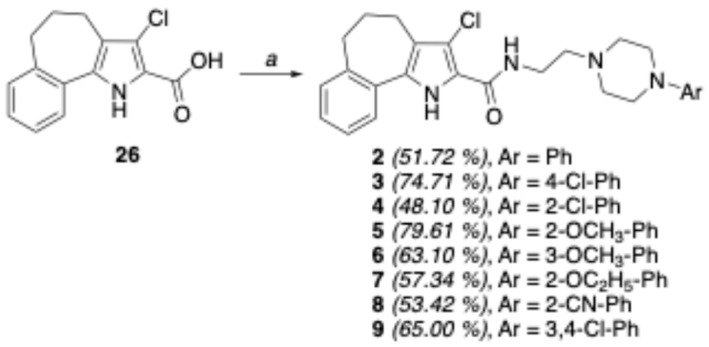
Reagents and conditions: (**a**) HOBt, DCC, CH_2_Cl_2_, 2 h, r.t., then appropriate arylpiperazinyl-ethanamine (**30**–**37**), 24 h, r.t. The numbers in parentheses represent the reaction yields.

The synthesis of carboxamides **2**–**9** is reported in Scheme 6. Acid **26** was activated with 1-hydroxybenzotriazole (HOBt) and *N*,*N’*-dicyclohexylcarbodiimide (DCC), and then the appropriate arylpiperazinyl-ethanamine (**30**–**37**) was added to give final compounds **2**–**9**.

**Scheme 7:**
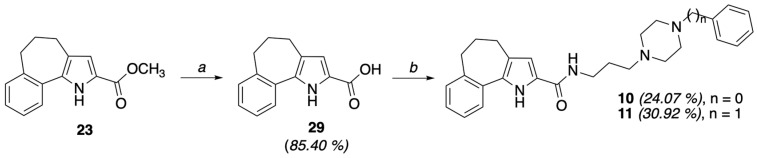
Reagents and conditions: (**a**) NaOH_aq_ 20%, CH_3_OH, 9 h, rfx; (**b**) HOBt, DCC, CH_2_Cl_2_, 2 h, r.t., then appropriate arylpiperazinyl-propanamine **38** or **39**, 24 h, r.t. The number in parentheses represents the reaction yields.

A similar synthetic route was used for the synthesis of dechlorinated derivatives, using the dechlorinated analogue of **26**, acid **29**, as the starting compound, and *N*_4_-phenyl-N_1_-propylamine or its *N*_4_-benzyl analogue to give compounds **10** and **11**, respectively (Scheme 7).

**Scheme 8:**
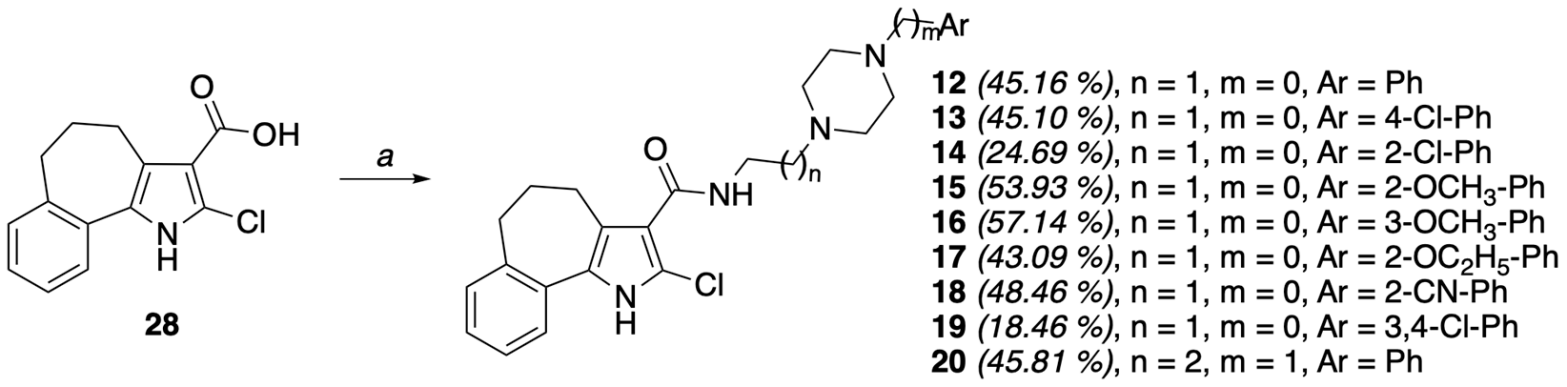
Reagents and conditions: (**a**) HOBt, DCC, CH_2_Cl_2_, 2 h, r.t., then appropriate arylpiperazinyl-alkylamine (**30**–**37**, **39**), 24 h, r.t. The numbers in parentheses represent the reaction yields.

Carboxamides **12**–**20** were synthesized by a similar synthetic route as described for isomers **2**–**11** starting from carboxylic acid **28** (Scheme 8).

### 2.2. Biology

We first evaluated the cytotoxic activity of tricyclic-pyrrole carboxamides **1**–**20** using the NCI-60 cell line panel, representing nine different human cancers: leukemia, melanoma, and cancers of the lung, colon, central nervous system (CNS), ovary, kidney, prostate, and breast (the one-dose graph for compounds **1**–**20** is reported in Supplementary Materials, Figures S43–S62). Compounds **1**, **5**, **10**, **11**, **17**, and **20** satisfied pre-determined threshold inhibition criteria in a minimum number of cell lines; therefore, they were also tested in the full five-dose assay [8] (Figures S63–S68 in the Supplementary Materials).

Next, derivatives **1**–**20** were evaluated for their antiviral activity against ZIKV, IAV, and SARS-CoV-2 on the respective permissive cell lines using three 10-fold serial dilutions (from 0.5 to 50 μM) for an initial screening. The toxicity of the compounds at the same concentration was tested on the same cells in parallel. Compounds **12**, **13**, and **19** showed promising antiviral effects and were further characterized to determine their IC_50_, CC_50_, and selectivity index (SI) values.

#### 2.2.1. Anticancer Activity

In general, leukemia, colon cancer, and melanoma were the main cell lines affected by the activity of the new compounds, whereas CNS, ovarian, and prostate cancer were only marginally affected. Eight compounds (**1**, **5**, **10**, **11**, **17**, **18**, **19**, and **20**) showed both a >90% growth inhibition (GI%) and cytotoxicity against several cancer cell lines; nine (**4**, **6**, **7**, **8**, **12**, **13**, **14**, **15**, and **16**) elicited 60% to 88% GI% without cytotoxicity, whereas only three were not cytotoxic and showed <60% GI% (**2**, **3**, and **9**; data reported in Supplementary Materials).

In the leukemia panel (Table 2), compounds **1**, **5**, **10**, **11**, **15**, **17**, and **20** showed high percentages of GI% ranging from 90.09% (**5**, SR cell line) to 99.95% (**17**, K-562 cell line). Compound **1** exhibited the highest GI% values on almost all panels. Except for **15**, these seven carboxamides also showed considerable cytotoxicity values on HL-60(TB) cells and, for compounds **10**, **11**, and **17**, on SR, K-562, RPMI-8226, and MCF-7 cells. By contrast, derivatives **7**, **8**, **12**, **13**, **14**, **16**, and **18** showed only modest GI% values.

Three colon cancer cell lines, HCT-116, HCT-15, and HT29, were targeted by compounds **5**, **10**, **11**, and **20**, which produced GI% ranging from 91.03% to 99.04% (Table 3). Analogues **1**, **7**, and **17** also showed high GI% values (>80%), whereas derivatives **6**, **12**, **13**, **14**, **15**, **16**, and **18** showed only low to moderate GI% values (61.27–79.37%). Seven compounds, carboxamides **1**, **5**, **10**, **11**, **15**, **17**, and **20**, showed cytotoxicity against several colon cancer cell lines: the most affected were HCC-2998, COLO 205, and HT29, although SW-620 and HCT-116 cell lines also showed sensitivity. Compound **1** was cytotoxic against six cell lines with values ranging from −21.01 (HCC-2998) to −52.66 (HCT-15). High cytotoxicity values in the COLO 205 cell line were also recorded for compounds **10**, **11**, and **20**: −58.03, −58.61, and −59.92, respectively, with compound **20** being the most cytotoxic against the HCC-2998 cell line (−91.84). Moreover, compound **20** showed the highest GI% (99.04%, HCT-15) among the whole series of novel compounds.

Twelve of the compounds tested were shown to be particularly cytotoxic against several melanoma cell lines (Table 4). Compound **20** was cytotoxic against all nine cell lines in the panel, similarly to derivative **1**, which, in contrast, showed 85.81% GI% against SK-MEL-2 cells. Both derivatives **10** and **11** elicited 95.25% and 99.73% GI% values against LOX and IMVI, respectively, and cytotoxicity against almost all other cell lines in the melanoma panel. Interestingly, eleven of the twenty compounds tested turned out to be cytotoxic against the SK-MEL-5 cell line, with values ranging from −3.84 (**14**) to −94.54 (**11**).

Compounds **5**, **10**, **11**, **18**, and **20** elicited GI% ranging from 90.73% to 98.58% in three of the nine cell lines of lung cancer (Table 5), with compound **11** showing the highest GI% value on the NCI-H460 cell line, whereas derivative **1** was the only one that showed cytotoxicity (−54.58) against the same line and, in general, against the whole non-small cell lung cancer panel. On this panel, derivatives **7**, **12**, and **14** showed only modest GI%.

Ten compounds (**1**, **10**, **11**, **13**, **14**, **15, 17**, **18**, **19**, and **20**) showed activity against the breast cancer cell line MDA-MB-468 (Table 6). Four of them (**13**, **15, 18**, and **19**) showed high GI% values ranging from 94.16% to 98.72%, and compound **14** also exhibited a high GI% (82.38%) against the same cell line. Five derivatives (**1**, **10**, **11**, **17**, and **20**) showed cytotoxicity against the MDA-MB-468 cell line with values between −3.75 (**1**) and −52.92 (**11**). Interestingly, the latter five compounds showed a moderate to high GI% against MCF-7 cells (**10**, 80.23%; **17**, 82.21%; **1**, 87.23%; **11**, 90.34%; and **20**, 95.45%).

Six of them, **11**, **13**, **15, 18**, **19**, and **20**, showed high GI% values (90.34–98.72%) against MCF7 (**11** and **20**) and MDA-MB-468 cell lines. Only carboxamide **14** showed a lower GI% value of 82.38%, whereas analogue **15** was the sole one to elicit considerable activity against BT-549.

Nine compounds (**1**, **5**, **10**, **11**, **14**, **15, 17**, **18**, and **20**) showed activity against five renal cancer cell lines (Table 7). In this panel, compound **11** emerged as the most interesting, with a GI% of 91.53% for CAKI-1 cells, although derivatives **1**, **5**, **10**, and **20** showed good GI% values between 83.00% and 89.42% for renal cancer. Analogues **14**, **15, 17**, and **18** showed only low-to-moderate values (60.70–71.64%). Carboxamide **1** showed cytotoxicity against 786-0 (−22.61) and CAKI-1 (−27.03) cell lines, whereas **10** and **11** were cytotoxic against RXF 393 cells with values of −8.69 and −9.45, respectively.

Finally, the anticancer activity of these carboxamides was assayed against CNS, ovarian, and prostate cancer panels. Concerning the CNS cancer, two compounds resulted in cytotoxicity against the SF-295 cell line, **1** (−24.35) and **11** (−38.81), whereas derivative **10** showed a GI% of 90.84% in the same cells. Other compounds of the series and the other five cell lines assayed gave GI% values < 60%, showing no cytotoxicity.

Ovarian and prostate cancer were not particularly affected by the activity of these derivatives, with compound **15** being the only one with moderate GI% against the ovarian OVCAR-4 (66.06%) and prostate PC-3 (75.11) cell lines.

Due to these preliminary results, six compounds, **1**, **5**, **10**, **11**, **17**, and **20**, were selected for the five-dose screen NCI-programme against the same panel of tumour cell lines [9,10,11,12,13,14,15,16,17].

In Table 8, the GI_50_ values are reported for compounds **1**, **10**, **11**, and **20** in the five-dose screen for each cell line of the three tumour panels—leukemia, colon cancer, and melanoma—on which they showed GI_50_ values < 3 μM. This value has been chosen as the threshold for the presentation of results in the text. Other cell line panels and results for derivatives **5** and **17** were not reported because they exceeded this value, except for carboxamide **17** (GI_50_: 2.57 μM) against RXF 393 renal cancer cells.

Compound **1** exhibited good activity against the leukemia panel (GI_50_: 1.76 μM–5.96 μM), with values < 3 μM against four of the six cell lines. A similar trend was observed in colon cancer cell lines (GI_50_: 1.80 μM–3.12 μM) and in eight melanoma cell lines (GI_50_: 1.54 μM–2.03 μM). The latter cell line was the one in which compound **1** is active, as reported in Table 8. Moreover, derivative **1** showed promising GI_50_ values on single cell lines of other tumours: 2.03 μM in NCI-H460 (non-small cell lung cancer), 2.68 μM in MDA-MB-468 (breast cancer), 2.29 μM and 2.70 μM on renal cancer cell lines CAKI-1 and RXF 393, respectively, and 1.89 μM in SF-295 (CNS cancer).

Compound **10** showed values ranging from 1.65 μM to 2.78 μM in five melanoma cell lines, whereas it had GI_50_ values < 3 μM against leukemia and colon cancer: cell lines SR (GI_50_: 2.29 μM) and K-562 (GI_50_: 2.58 μM) in the leukemia panel, and COLO 205 (GI_50_: 1.92 μM) and HCC-2998 (GI_50_: 2.51 μM) in the colon cancer cell panel. As for compound **1**, derivative **10** showed GI_50_ values < 3 μM in other cell lines, such as 2.93 μM in MDA-MB-468 (breast cancer) and 2.06 μM in RXF 393 (renal cancer).

Concerning derivative **11**, it showed GI_50_ values ranging from 2.20 μM to 2.92 μM in 83% of leukemia cell lines and from 1.72 μM to 2.59 μM in eight out of nine melanoma cell lines. Compound **11** showed the best GI% values compared with compounds **1**, **10**, and **20** in COLO 205 (GI_50_: 1.65 μM) and HCC-2998 (GI_50_: 1.73 μM) colon cancer cell lines. Moreover, **11** showed GI_50_ values < 3 μM on the following cell lines: HOP-92 (non-small cell lung cancer, 2.72 μM), MDA-MB-468 (MCF-7 (breast cancer, 2.69 and 2.77 μM, respectively), and RXF 393 (renal cancer, 1.96 μM).

Finally, derivative **20** elicited promising GI_50_ values both on the melanoma and on colon cancer, with a GI_50_ value of 0.53 μM against the SR leukemia cell line, resulting in the best GI_50_ value of the whole series of new compounds.

Next, a mean graph midpoint (MG_MD) was calculated [18,19] for compounds **1**, **5**, **10**, **11**, **17**, and **20** and reported as waterfall graphs GI_50_, TGI, and LC_50_ in the Supplementary Materials (Figures S69–S86). In Table 9, we reported the average GI_50_ for the nine-cancer panels, underlining both the activity and selectivity for compounds **1**, **10**, **11**, and **20**. All derivatives showed selectivity against melanoma cell lines. Among them, the best result was obtained for compound **1** with a GI_50_ value of 2.14 μM. In the same panel, compound **11** showed a GI_50_ value of 2.34 μM, similarly to **1**, whereas derivatives **10** and **20**, with GI_50_ values of 2.95 μM and 3.39 μM, respectively, showed similar selectivity against leukemia as **1** (GI_50_: 2.82 μM) and **11** (GI_50_: 2.88 μM).

Finally, Total Growth Inhibition (TGI) and Lethal Concentration 50% (LC_50_) values are reported in Table 10 for compounds **1**, **10**, **11** and **20**. Except for the SK-MEL-2 cell line, derivative **1** showed both TGI and LC_50_ values in the range of single-digit micromolar, whereas derivative **11** had similar TGI values only. Compounds **10** and **20** are similar to each other, showing also several single-digit micromolar values both for total GI% and LC_50_.

#### 2.2.2. Antiviral Activity

We developed a protocol to screen the potential antiviral activity of compounds **1**–**20** against ZIKV (strain Brazil) on Huh-7 cells, against an H1N1 IAV strain (PR/8/1934) on MDCK cells, and against SARS-CoV-2 (strain Milano) on Vero-TMPRSS. Each compound was tested in duplicate at concentrations of 50 μM, 5 μM, and 0.5 μM. Treatment was applied immediately after infection, and at 24–48 h post-infection, supernatants were collected to assess viral yield, determined as PFU/mL for ZIKV and SARS-CoV-2 or as TCID_50_/mL for IAV. The test allowed us to evaluate both the antiviral action and the cytotoxicity of each compound, comparing them with reference compounds, such as sofosbuvir for ZIKV and EIDD-1931 for IAV and SARS-CoV-2 (Supplementary Materials, Figures S87–S89).

**Figure 3:**
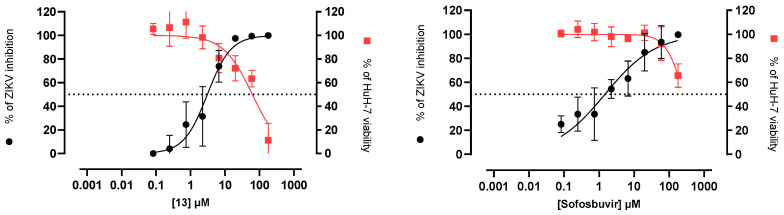
Dose–response curves of the % ZIKV replication inhibition (black line) and % cell viability (red line) for compound **13** and for control drug sofosbuvir.

From the initial screening, only compound **13** (Figure 3) showed a modest effect against ZIKV in Huh-7 cells, with a selectivity index (SI) of 20.20 (EC_50_ = 3.08 μM; CC_50_ = 62.22 μM), indicating limited antiviral potential under the tested conditions. In comparison, sofosbuvir exhibited a markedly higher SI of 178.32 (EC_50_ = 1.47 μM; CC_50_ = 261.60 μM).

**Figure 4:**
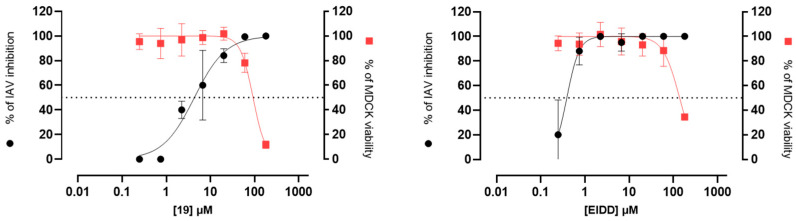
Dose–response curves of % IAV replication inhibition (black line) and % cell viability (red line) of compound **19** and of control drug EIDD.

Similarly, only compound **19** exhibited slight antiviral activity against IAV in MDCK cells (Figure 4) with a selectivity index (SI) of 21.36 (EC_50_ = 4.31 μM; CC_50_ = 92.06 μM). In contrast, the reference drug EIDD-1931 exhibited a significantly higher SI of 353.85 (EC_50_ = 0.39 μM; CC_50_ = 138.00 μM).

**Figure 5:**
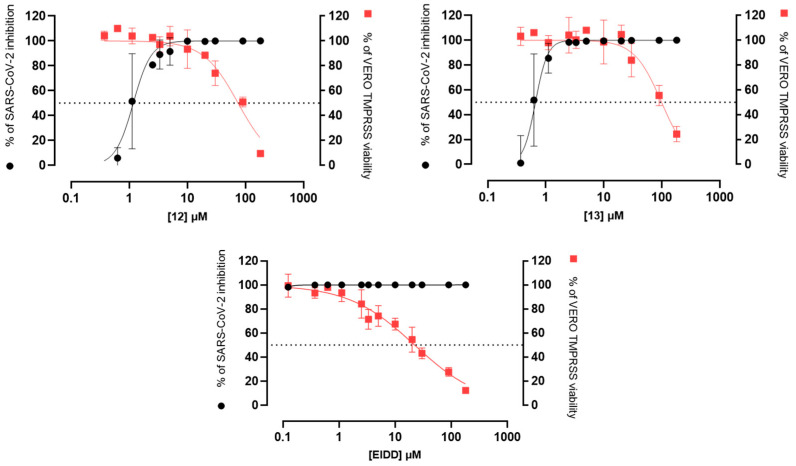
Dose–response curves of the % SARS-CoV-2 replication inhibition (black line) and % cell viability (red line) of the compounds **12** and **13** and of control drug EIDD.

Despite the limited activity observed against ZIKV and IAV, the results obtained with SARS-CoV-2 were quite encouraging. Notably, compounds **12** and **13** emerged as the most promising candidates, exhibiting significant inhibition of viral replication in VERO TMPRSS cells. Compound **12** showed an SI of 63.63 (EC_50_ = 1.17 μM; CC_50_ = 74.45 μM), while compound **13** showed an SI of 150.00 (EC_50_ = 0.66 μM; CC_50_ = 99.00 μM) (Figure 5). EIDD-1931 was tested as a control, with a SI of 443.60 (EC_50_ = 0.05 μM; CC_50_ = 22.18 μM). However, one must bear in mind that only one cell line was tested in this in vitro assay; therefore, the results need to be interpreted with caution.

## 3. Discussion

The preliminary results from the full NCI 60 cell panel test and antiviral assays suggested some considerations about the relationships between the chemical structure and the activity of the new compounds synthesized.

Firstly, concerning the anti-tumoural activity, we identified compound **1** as the most promising benzocycloheptapyrrole carboxamide with GI% and cytotoxicity on different cell lines. It showed interesting GI% values in almost all cell lines in the leukemia panel. Moreover, compound **1** showed good cytotoxic activity against colon cancer and melanoma cell lines, except for MK12 (colon) and SK-MEL-2 (melanoma). Therefore, we investigated the activity of aryl-piperazines **2**–**9**, designed as reported above. Phenyl-piperazine **2** and its 4-Cl (**3**) and 3,4-Cl (**9**) analogues had poor-to-low activity. The other two compounds, **4** and **8**, with electron-withdrawing groups (2-Cl and 2-CN, respectively) showed good activity. The same trend was observed with the introduction of electron-donating (**5**, **6**, and **7**) groups, with the 2-OCH3 (**5**) substituent on the phenyl ring turning out to be the one with the highest anticancer activity. The dechlorination of the pyrrole and the lengthening of the linker between carboxamide and piperazine led to compound **10**, bearing a phenyl on the *N*_4_-piperazine atom, which, like its benzyl analogue **11**, showed interesting effects on the same cell lines affected by **1**. Indeed, as for **1**, compounds **10** and **11** elicited significant GI% values against several leukemia, colon cancer, and melanoma cell lines, turning out to be cytotoxic for some of them.

We also evaluated the effect of the Cl/carboxamide position exchange on the pyrrole ring in compounds **12**–**20**. All compounds exhibited moderate-to-good activity against several cell lines, particularly those bearing a 2-OC_2_H_5_ (**17**), 2-CN (**18**), and 3,4-Cl (**19**) group. Interestingly, derivative **20**, bearing a propyl linker, showed the highest GI% value among all compounds, with a GI_50_ of 0.53 μM in the SR leukemia cell line.

Concerning the anti-tumour activity of these carboxamides, derivative **1** showed an MG_MID Log_10_ GI_50_ = −5.32, which accounts for the highest micromolar activity against all cell lines, particularly in the leukemia (GI_50_: 2.82 μM), colon cancer (GI_50_: 2.34 μM), melanoma (GI_50_: 2.14 μM), and ovarian cancer panels (GI_50_: 2.29 μM).

From this preliminary structure–activity relationship (SAR) study, we can conclude that compound **1** has structural features responsible for its promising anticancer activity and that in its piperazinyl-benzocycloheptapyrrole-carboxamide derivatives, the substituents on the phenyl ring do not seem to be as important in both series of compounds of this newly synthesized series.

Concerning the antiviral activity, only three compounds (**12, 13**, and **19**) out of twenty showed interesting effects against emerging RNA viruses, ZIKV and SARS-CoV-2 included in the screening. The three were further characterized to determine their EC_50_, CC_50_, and SI and compared with reference antiviral drugs sofosbuvir for ZIKV and EIDD-1931 for both IAV and SARS-CoV-2. Compounds **13** and **19** displayed modest inhibitory activity against ZIKV (SI = 20.20 in Huh-7 cells) and IAV (SI = 21.36 in MDCK cells), respectively. The most notable antiviral activity was observed in the SARS-CoV-2 infection model. Compound **12** showed a good selectivity index (SI) of 63.63 in VERO TMPRSS cells with a good balance between efficacy and cytotoxicity. Even more interestingly, **13** achieved an SI of 150.00, a result that positions it as the most promising candidate antiviral drug in the series. The potent antiviral activity demonstrates the potential for these compounds to be valuable leads for development against SARS-CoV-2.

## 4. Materials and Methods

### 4.1. Chemistry

#### 4.1.1. General Methods

All reactions involving air- or moisture-sensitive compounds were performed with atmospheric N_2_. Solvents and reagents were obtained from commercial suppliers and were used without further purification. Compound **22** [6] was synthesized and identified according to and by comparison with literature. (*S*)-(−)-2-(aminomethyl)-1-ethylpyrrolidine, bromoalkyl-phtalimides (**40** and **41**), and aryl piperazines (**42**–**50**) were purchased.

Microwave-assisted reactions were performed with Microwave Initiator Eight 2.5 (Biotage^®^, Uppsala, Sweden) in the standard configuration as delivered, including proprietary software. All experiments were carried out in sealed microwave process vials under normal absorption. At the end of the reaction, the vial was cooled to 25 °C by air jet cooling before opening. Reaction temperatures were monitored by an IR sensor on the outside wall of the reaction.

Flash column chromatography purification (FC) was performed automatically on Flash-master (Biotage^®^, Uppsala, Sweden) with pre-packed Biotage^®^ SNAP silica gel cartridges or manually on silica gel (Kieselgel 60, 0.040−0.063 mm, Merck^®^, Darmstadt, Germany). Thin-layer chromatography (TLC) was performed with Polygram SIL N-HR/HV_254_ pre-coated plastic sheets (0.2 mm) on aluminum sheets (Kieselgel 60 F254, Merck^®,^ Darmstadt, Germany). Melting points were obtained on a Köfler melting point apparatus and are uncorrected.

IR spectra were recorded as KBr pellets with a Jasco^®^ (Cremella, Italy) FT/IR 460 plus spectrophotometer after 64 scans at 4 cm^−1^ resolution and are expressed in *ν* (cm^−1^). NMR experiments were recorded at room temperature, and all spectra were recorded on a Varian^®^ (Irvine, CA, USA) XL-200 NMR spectrometer (200 MHz for ^1^H and 50 MHz for ^13^C). Spectra were acquired in deuterated chloroform (chloroform-d) as a solvent. Chemical shifts (δ) for ^1^H-NMR spectra are reported in parts per million (ppm) using the residual non-deuterated solvent resonance as the internal standard (chloroform-d: 7.26 ppm, ^1^H, and 77.16 ppm, ^13^C). Data are reported as follows: chemical shift (sorted in descending order), multiplicity (s for singlet, br s for broad singlet, d for doublet, t for triplet, dd for double doublet, q for quadruplet, qu for quintuplet, and m for multiplet), and integration and coupling constants *(J*) in Hertz (Hz) (Figures S3–S22). Spectroscopic data are in accordance with the structures. LC/MS analyses were run on an Agilent^®^ (Santa Clara, CA, USA) 1100 LC/MSD system consisting of a single quadrupole detector (SQD) mass spectrometer (MS) equipped with an electrospray ionization (ESI) interface and a photodiode array (PDA) detector, range 120−550 nm. ESI in positive mode was applied. Mobile phases: (A) MeOH in H_2_O (8:2) (Figures S23–S42). Analyses were performed with a flow rate of 0.9 mL/min and a temperature of 350 °C. The purity of all final compounds was determined by elemental analysis on a PerkinElmer^®^ (Waltham, MA, USA) 240B analyzer (C, H, and N). All the final compounds were found to be >95% when analyzed.

#### 4.1.2. Synthesis of Methyl-5,6-dihydro-*4H*-benzo[6,7]cyclohepta[1,2-*b*]pyrrole-2-carboxylate (**23**) and Methyl-5,6-dihydro-*4H*-benzo[6,7]cyclohepta[1,2-*b*]pyrrole-3-carboxylate (**24**)

To a solution of oxime **22** (10.43 mmol) and DABCO (1.04 mmol, 0.1 eq) in toluene (18 mL), methyl propiolate (10.43 mmol, 1 eq) was dropwise added at −5 °C. The solution was brought back to room temperature, then underwent a double microwave irradiation (normal abs) cycle: the first at 80 °C for 10 min, followed by the second at 170 °C for another 10 min. After solvent evaporation by rotavapor, the crude product was purified by FC (petroleum ether/EtOAc 9/1) to give ester **23** with a retention factor (R*_f_*) of 0.237 as a yellow solid (0.56 g, 22.46%), mp = 126–128 °C, ^1^H NMR (CDCl_3_) ppm: 2.03–2.08 (m, 2H), 2.78–2.83 (m, 4H), 3.86 (s, 3H), 6.80 (d, 1H, J = 2.8 Hz), 7.18–7.19 (m, 2H), 7.22–7.28 (m, 2H), 7.46 (d, 1H, J = 7 Hz), 9.11 (br s, 1H, NH exch. with D_2_O); Anal. calcd for C_15_H_15_NO_2_: C, 74.67; H, 6.27; N, 5.81. Found: C, 74.89; H, 6.29; N, 5.83; and **24** with a lower R*_f_* (0.106) as a yellow solid (0.494 g, 19.63%), mp = 166–168 °C, ^1^H NMR (200 MHz, CDCl_3_) ppm: 2.02–2.04 (m, 2H), 2.79–2.82 (m, 2H), 3.14 (t, 2H, J = 6.8 Hz), 3.81 (s, 3H), 7.13–7.18 (m, 2H), 7.20–7.25 (m, 1H), 7.34 (d, 1H, J = 7.6 Hz), 7.50 (d, 1H, *J* = 3.2 Hz), 8.59 (br s, 1H, NH exch. with D_2_O). Anal. calcd for C_15_H_15_NO_2_: C, 74.67; H, 6.27; N, 5.81. Found: C, 74.93; H, 6.29; N, 5.83.

#### 4.1.3. Methyl 3-chloro-5,6-dihydro-*4H*-benzo[6,7]cyclohepta[*b*]pyrrole-2-carboxylate (**25**)

A solution of **23** (1.76 mmol) and *N*-chlorosuccinimide (1.94 mmol, 1.1 eq) in chloroform (8 mL) was refluxed for 3 h, then allowed to cool to room temperature and washed with saturated aqueous NaHCO_3_. The organic phase was dried (Na_2_SO_4_) and concentrated to yield a crude product, which trituration with ether gave **25** as a white solid (0.353 g, 72.79%), mp = 160–162 °C, ^1^H NMR (CDCl_3_) ppm: 1.98–2.12 (m, 2H), 2.70–2.90 (m, 4H), 3.92 (s, 3H), 7.18–7.50 (m, 4H), 9.01 (br s, 1H, NH exch. with D_2_O). Anal. calcd for C_15_H_14_ClNO_2_: C, 65.34; H, 5.12; Cl, 12.86; N, 5.08. Found: C, 65.50; H, 5.13; Cl, 12.89; N, 5.09.

#### 4.1.4. 3-Chloro-5,6-dihydro-*4H*-benzo[6,7]cyclohepta[*b*]pyrrole-2-carboxylic acid (**26**)

A mixture of ester **25** (1.09 mmol) in hydro-alcoholic solution (H_2_O/EtOH 1/2, 10.5 mL) of 2.5 M NaOH was refluxed for 12 h, then poured onto ice. The solution was acidified with concd. HCl and the solid precipitate were filtered, taken up in 5% aqueous NaHCO_3_ and filtered off. Concentrated HCl was dropwise added to the filtrate to give a white solid, which was filtered and air-dried to give acid **26** as a white solid (0.260 g, 91.72%), mp = 171–173 °C, ^1^H NMR (CDCl_3_) ppm: 2.28–2.98 (m, 2H), 2.60–2.90 (m, 4H), 3.89 (br s, 1H, OH exch. with D_2_O), 7.12–7.60 (m, 4H), 9.16 (br s, 1H, NH exch. with D_2_O). Anal. calcd for C_14_H_12_ClNO_2_: C, 64.24; H, 4.62; Cl, 13.55; N, 5.35. Found: C, 64.37; H, 4.63; Cl, 13.58; N, 5.36.

#### 4.1.5. General Procedure I: Synthesis of 5,6-dihydro-*4H*-benzo[6,7]cyclohepta[*b*]pyrrole-3-carboxylic acid (**27**) and 5,6-dihydro-*4H*-benzo[6,7]cyclohepta[*b*]pyrrole-2-carboxylic acid (**29**)

To a solution of the appropriate ester **23** or **24** (0.83 mmol) in CH_3_OH (10 mL), an aqueous 20% solution of NaOH (10 mL) was added, and the mixture was refluxed for 9 h. The resulting solution was poured onto ice and concentrated HCl was added. The resulting precipitate was filtered and air-dried to give the corresponding acid. Acid **27** (from **24**) as a beige solid (0.159 g, 84.15%), mp = 218–220 °C, ^1^H NMR (CDCl_3_) ppm: 1.58 (br. s, 1H, OH exch. with D_2_O), 1.99–2.10 (m, 2H), 2.80 (t, 2H, *J* = 5.0 Hz), 3.11 (t, 2H, *J* = 7.2 Hz), 7.16–7.20 (m, 2H), 7.33–7.38 (m, 2H), 7.59–7.63 (m, 1H), 8.53 (br. s, 1H, NH exch. with D_2_O). Anal. calcd for C_14_H_13_NO_2_: C, 73.99; H, 5.77; N, 6.16. Found: C, 74.25; H, 5.79; N, 6.18. Acid **29** (from **23**) as a white solid (0.161 g, 84.15%), mp = 198–200 °C, ^1^H NMR (CDCl_3_) ppm: 2.02–2.04 (m, 2H), 2.75–2.80 (m, 4H), 3.80 (br. s, 1H, OH exch. with D_2_O), 6.90 (d, 1H, *J* = 1.6 Hz), 7.17–7.24 (m, 3H), 7.43 (d, 1H, *J* = 7.6 Hz), 9.14 (br. s, 1H, NH exch. with D_2_O). Anal. calcd for C_14_H_13_NO_2_: C, 73.99; H, 5.77; N, 6.16. Found: C, 74.24; H, 5.79; N, 6.17.

#### 4.1.6. 2-Chloro-5,6-dihydro-*4H*-benzo[6,7]cyclohepta[*b*]pyrrole-3-carboxylic acid (**28**)

A solution of **27** (2.07 mmol) and *N*-chlorosuccinimide (2.48 mmol, 1.2 eq) in chloroform (12 mL) was refluxed for 4 h, then allowed to cool to room temperature and extracted with saturated aqueous NaHCO_3_. The aqueous solution was acidified with concentrated HCl. And the precipitate was filtered, washed (H_2_O), and air-dried to yield **25** as a grey solid (0.424 g, 78.52%), mp = 188–190 °C, ^1^H NMR (CDCl_3_) ppm: 1.68 (br. s, 1H, OH exch. with D_2_O), 1.98–2.10 (m, 2H), 2.80 (t, 2H, *J* = 5.0 Hz); 3.11 (t, 2H, *J* = 7.2 Hz), 7.16–7.20 (m, 2H); 7.26–7.31 (m, 2H); 8.48 (br. s, 1H, NH exch. with D_2_O). Anal. calcd for C_14_H_12_ClNO_2_: C, 64.24; H, 4.62; Cl, 13.55; N, 5.35. Found: C, 64.10; H, 4.61; Cl, 13.51; N, 5.33.

#### 4.1.7. (*S*)-3-Chloro-*N*-(1-ethyl-2-pyrrolidinylmethyl)-5,6-dihydro-*4H*-benzo[6,7]cyclohepta[1,2-*b*]pyrrol-2-carboxamide (**1**)

To a stirred solution of **26** (0.38 mmol) in CH_2_Cl_2_ (1 mL) were added CDI (0.41 mmol, 1.1 eq) and HOBt (0.41 mmol, 1.1 eq). After stirring the reaction mixture at room temperature for 1.5 h, (*S*)-(−)-2-(aminomethyl)-1-ethylpyrrolidine (0.38 mmol, 1 eq) was added and stirring continued for 12 h. The reaction mixture was filtered on Celite^®^ and the filtrate evaporated. The crude residue was taken up with EtOAc and washed (saturated aqueous NaCl). The organic phase was dried (Na_2_SO_4_) and evaporated to give a crude product, which was purified by flash chromatography (CHCl_3_/CH_3_OH 9/1) to give pure title compound **1** as a beige solid (0.091 g, 64.53%), mp = 136–139 °C, ^1^H NMR (CDCl_3_) ppm: 1.15 (t, 3H, *J* = 7.2 Hz), 1.60–1.90 (m, 4H), 1.99–2.15 (m, 2H), 2.16–2.38 (m, 2H), 2.72–2.95 (m, 6H), 3.22–3.411 (m, 2H), 3.66–3.80 (m, 1H), 7.17–7.26 (m, 3H), 7.50 (d, 1H, *J* = 7.6 Hz), 7.58 (br s, 1H, NH exch. with D_2_O), 9.43 (br s, 1H, exch. with D_2_O). ^13^C NMR (CDCl_3_) ppm: 14.20 (CH_3_), 23.07 (CH_2_), 25.31 (CH_2_ × 2), 26.42 (CH_2_), 28.47 (CH_2_), 34.76 (CH_2_), 40.95 (CH_2_), 48.18 (CH_2_), 53.56 (CH), 113.75 (C), 118.03 (C), 121.04 (CH), 124.66 (CH), 126.06 (CH), 127.27 (C), 129.27 (C), 129.69 (CH), 130.25 (C), 141.38 (C), 160.10 (C). MS (ESI): C_21_H_26_ClN_3_O requires *m*/*z* 371.18, found 372.18 [M+H]^+^. C_21_H_26_ClN_3_O: C, 67.82; H, 7.05; Cl, 9.53; N, 11.30. Found: C, 67.58; H, 6.96; Cl, 9.51; N, 11.27.

#### 4.1.8. General Procedure II: Synthesis of *N*-(2-(4-arylpiperazin-1-yl)ethyl)phtalimides **51**–**58** and *N*-(3-(4-arylpiperazin-1-yl)propyl) phtalimides **59** and **60**

To a solution of *N*-bromoalkylphtalimide **40** or **41** (1.23 mmol) in anhydrous DMF (3.8 mL) were added the appropriate aryl-piperazine (**42**–**50**) (1 eq) and K_2_CO_3_ (2.5 eq): the suspension underwent microwave irradiation (normal abs) at 55 °C for 2 h, then was poured onto water, and the crude precipitate was taken up in EtOAc. The solution was washed (H_2_O), dried (Na_2_SO_4_), and concentrated to give a crude product, which was purified by FC to yield the *N*-(arylpiperazin-1-yl)alkyl)phtalimides **51**–**60**.

#### 4.1.9. *N*-(2-(4-Phenylpiperazin-1-yl)ethyl)phtalimide (**51**)

The title compound was prepared from **40** and 1-phenylpiperazine (**42**) using the general procedure II to afford **51** as a pale yellow solid (0.317 g, 53.46%), after FC purification (petroleum ether/EtOAc 8/2), mp = 152–155 °C, ^1^H NMR (CDCl_3_) ppm: 2.60–2.80 (m, 6H), 3.13 (t, 4H, *J* = 5.0 Hz), 3.87 (t, 2H, *J* = 6.6 Hz), 6.78–6.93 (m, 3H), 7.19–7.28 (m, 2H), 7.67–7.75 (m, 2H), 7.80–7.88 (m, 2H). Anal. calcd for C_20_H_21_N_3_O_2_: C, 71.62; H, 6.31; N, 12.53. Found: C, 71.44; H, 6.29; N, 12.50.

#### 4.1.10. *N*-(2-(4-(4-Chlorophenyl)piperazin-1-yl)ethyl)phtalimide (**52**)

The title compound was prepared from **40** and 1-(4-chlorophenyl)piperazine (**43**) using the general procedure II to afford **52** as a pale yellow solid (0.061 g, 13.37%), after FC purification (petroleum ether/EtOAc 8/2, then CHCl_3_/CH_3_OH 9.8/0.2), mp = 165–167 °C, ^1^H NMR (CDCl_3_) ppm: 2.63–2.74 (m, 6H), 3.09 (t, 4H, *J* = 5.2 Hz), 3.86 (t, 2H, *J* = 6.6 Hz), 6.81 (d, 2H, *J* = 9.2 Hz), 7.17 (d, 2H, *J* = 9.0 Hz), 7.68–7.73 (m, 2H), 7.80–7.88 (m, 2H). Anal. calcd for C_20_H_20_ClN_3_O_2_: C, 64.95; H, 5.45; Cl, 9.85; N, 11.36. Found: C, 65.14; H, 5.47; Cl, 9.88; N, 11.39.

#### 4.1.11. *N*-(2-(4-(2-Chlorophenyl)piperazin-1-yl)ethyl)phtalimide (**53**)

The title compound was prepared from **40** and 1-(2-chlorophenyl)piperazine (**44**) using the general procedure II to afford **53** as a white solid (0.138 g, 30.34%), after FC purification (petroleum ether/EtOAc 7/3), mp = 106–109 °C, ^1^H NMR (CDCl_3_) ppm: 2.63–2.74 (m, 6H), 2.96–3.10 (m, 4H), 3.87 (t, 2H, *J* = 6.6 Hz), 6.89–7.31 (m, 2H), 7.18 (dd, 1H, *J*_o_ = 7.4 Hz, *J*_m_ = 1.6 Hz), 7.34 (dd, 1H, *J*_o_ = 7.6 Hz, *J*_m_ = 1.6 Hz), 7.65–7.77 (m, 2H), 7.82–7.90 (m, 2H). Anal. calcd for C_20_H_20_ClN_3_O_2_: C, 64.95; H, 5.45; Cl, 9.85; N, 11.36. Found: C, 64.82; H, 5.44; Cl, 9.82; N, 11.34.

#### 4.1.12. *N*-(2-(4-(2-Methoxyphenyl)piperazin-1-yl)ethyl)phtalimide (**54**)

The title compound was prepared from **40** and 1-(2-methoxyphenyl)piperazine (**45**) using the general procedure II to afford **54** as a white solid (0.163 g, 36.20%), after FC purification (petroleum ether/EtOAc 1/1), mp = 115–117 °C, ^1^H NMR (CDCl_3_) ppm: 2.56–2.76 (m, 2H), 2.99–3.10 (m, 4H), 3.85 (s, 3H), 3.86–3.91 (m, 2H), 6.84–6.96 (m, 4H), 7.67–7.74 (m, 2H), 7.80–7.90 (m, 2H). Anal. calcd for C_21_H_23_N_3_O_3_: C, 69.02; H, 6.34; N, 11.50. Found: C, 61.90; H, 6.33; N, 11.48.

#### 4.1.13. *N*-(2-(4-(3-Methoxyphenyl)piperazin-1-yl)ethyl)phtalimide (**55**)

The title compound was prepared from **40** and 1-(3-methoxyphenyl)piperazine (**46**) using the general procedure II to afford **55** as a yellow solid (0.186 g, 41.20%), after FC purification (petroleum ether/EtOAc 1/1), mp = 110–112 °C, ^1^H NMR (CDCl_3_) ppm: 2.62–2.75 (m, 6H), 3.13 (t, 4H, J = 4.8 Hz), 3.78 (s, 3H), 3.86 (t, 2H, *J* = 6.6 Hz), 6.33–6.45 (m, 2H), 6.46–6.54 (m, 1H), 7.15 (t, 1H, *J* = 8.2 Hz), 7.66–7.76 (m, 2H), 7.80–7.90 (m, 2H). Anal. calcd for C_21_H_23_N_3_O_3_: C, 69.02; H, 6.34; N, 11.50. Found: C, 61.83; H, 6.32; N, 11.46.

#### 4.1.14. N-(2-(4-(2-Ethoxyphenyl)piperazin-1-yl)ethyl)phtalimide (56)

The title compound was prepared from **40** and 1-(2-ethoxyphenyl)piperazine (**47**) using the general procedure II to afford **56** as a yellow solid (0.100 g, 19.70%), after FC purification (CHCl_3_/CH_3_OH 98/2), mp = 102–105 °C, ^1^H NMR (CDCl_3_) ppm: 1.45 (t, 3H, *J* = 6.8 Hz), 2.65–2.76 (m, 6H), 3.00–3.20 (m, 4H), 3.87 (t, 2H, *J* = 6.6 Hz), 4.05 (q, 2H, *J* = 7.2 Hz), 6.80–6.97 (m, 4H), 7.66–7.79 (m, 2H), 7.84–7.92 (m, 2H). Anal. calcd for C_22_H_25_N_3_O_3_: C, 69.64; H, 6.64; N, 11.07. Found: C, 69.85; H, 6.66; N, 11.10.

#### 4.1.15. 4-(4-(2-(Phtalimido)ethyl)piperazin-1-yl)benzonitrile (**57**)

The title compound was prepared from **40** and 2-(piperazin-1-yl)benzonitrile (**48**) using the general procedure II to afford **57** as an orange solid (0.171 g, 33.46%), after FC purification (petroleum ether/EtOAC 6/4), mp = 111–113 °C, ^1^H NMR (CDCl_3_) ppm: 2.69–2.77 (m, 6H), 3.14–3.19 (m, 4H), 3.86 (t, 2H, *J* = 6.6 Hz), 6.90–7.10 (m, 2H), 7.44 (dd, 1H, *J*_o_ = 9.6 Hz, *J*_m_ = 1.6 Hz), 7.54 (dd, 1H, *J*_o_ = 8.0 Hz, *J*_m_ = 1.6 Hz), 7.68–7.78 (m, 2H), 7.80–7.90 (m, 2H). Anal. calcd for C_21_H_20_N_4_O_2_: C, 69.98; H, 5.59; N, 15.55. Found: C, 69.84; H, 5.58; N, 15.52.

#### 4.1.16. *N*-(2-(4-(3,4-Dichlorophenyl)piperazin-1-yl)ethyl)phtalimide (**58**)

The title compound was prepared from **40** and 1-(3,4-dichlorophenyl)piperazine (**49**) using the general procedure II to afford **58** as a white solid (0.170 g, 34.26%), after FC purification (petroleum ether/EtOAC 7/3), mp = 165–168 °C, ^1^H NMR (CDCl_3_) ppm: 2.68 (dt, 6H, *J* = 5.2 Hz), 3.10 (dt, 4H, *J* = 5.2 Hz), 3.86 (t, 2H, *J* = 6.1 Hz), 6.71 (dd, 1H, *J*_o_ = 9 Hz, *J*_m_ = 3.0 Hz), 6.92 (d, 1H, *J* = 3.0 Hz), 7.22 (s, 1H), 7.67–7.76 (m, 2H), 7.80–7.90 (m, 2H). Anal. calcd for C_20_H_19_Cl_2_N_3_O_2_: C, 59.42; H, 4.74; Cl, 17.54; N, 10.39. Found: C, 59.54; H, 4.83; Cl, 17.57; N, 10.41.

#### 4.1.17. *N*-(2-(4-Phenylpiperazin-1-yl)propyl)phtalimide (**59**)

The title compound was prepared from **41** and 1-phenylpiperazine (**42**) using the general procedure II to afford **59** as a pale yellow solid (0.392 g, 91.14%), after trituration with petroleum ether, mp = 117–119 °C, ^1^H NMR (CDCl_3_) ppm: 1.91 (qu, 2H, *J* = 6.8 Hz), 2.48 (t, 2H, *J* = 6.8 Hz), 2.54 (t, 4H, *J* = 4.8 Hz), 3.04 (t, 4H, *J* = 4.8 Hz), 3.79 (t, 2H, *J* = 6.8 Hz), 6.81–6.87 (m, 3H), 7.21–7.26 (m, 2H), 7.68–7.70 (m, 2H), 7.82–7.84 (m, 2H). Anal. calcd for C_21_H_23_N_3_O_2_: C, 72.18; H, 6.63; N, 12.03. Found: C, 72.32; H, 6.64; N, 12.05.

#### 4.1.18. *N*-(2-(4-Benzylpiperazin-1-yl)propyl)phtalimide (**60**)

The title compound was prepared from **41** and 1-benzylpiperazine (**50**) using the general procedure II to afford **60** as a yellow solid (0.424 g, 94.21%), after trituration with petroleum ether, mp = 132–135 °C, ^1^H NMR (CDCl_3_) ppm: 1.86 (qu, 2H, *J* = 6.8 Hz), 2.34–2.43 (m, 10H), 3.40 (s, 2H), 3.75 (t, 2H, *J* = 6.8 Hz), 6.81–6.87 (m, 3H), 7.21–7.26 (m, 2H), 7.68–7.70 (m, 2H), 7.82–7.84 (m, 2H). Anal. calcd for C_22_H_25_N_3_O_2_: C, 72.70; H, 6.93; N, 11.56. Found: C, 72.55; H, 6.92; N, 11.54.

#### 4.1.19. General Procedure III: Synthesis of amino-alkylaryl-piperazines **30**–**39**

To a solution of phtalimide (**51**–**60**) (0.724 mmol) in EtOH (3 mL) was added NH_2_NH_2_·H_2_O (5.2 eq), and the resulting solution was reacted under microwave irradiation (abs. low) at 80 °C for 20 min. The resulting suspension was filtered and then washed with EtOH, whose evaporation gave a residue, washed with EtOAc, and filtered. The organic solution was then dried (Na_2_SO_4_) and evaporated, providing a crude product that, purified by FC with an appropriate mixture of eluents, allowed us to obtain the desired amine (**30**–**39**).

#### 4.1.20. 2-(4-Phenylpiperazin-1-yl)ethyl-1-amine (**30**)

The title compound was prepared from **51** using the general procedure III to afford **30** as a hygroscopic brown solid (0.058 g, 33.14%), after FC purification (CHCl_3_/CH_3_OH 9/1), mp = 90–95 °C, ^1^H NMR (CDCl_3_) ppm: 1.71 (br s, 2H, NH_2_ exch. with D_2_O), 2.50 (t, 2H, *J* = 5.8 Hz), 2.59–2.66 (m, 4H), 2.85 (t, 2H, *J* = 6.6 Hz), 3.17–3.25 (m, 4H), 6.80–6.97 (m, 3H), 7.21–7.33 (m, 2H). Anal. calcd for C_12_H_19_N_3_: C, 70.20; H, 9.33; N, 20.47. Found: C, 70.27; H, 9.34; N, 20.49.

#### 4.1.21. 2-(4-(4-Chlorophenyl)piperazin-1-yl)ethyl-1-amine (**31**)

The title compound was prepared from **52** using the general procedure III to afford **31** as a hygroscopic pink solid (0.169 g, 84.54%), after FC purification (CHCl_3_/CH_3_OH 8/2), mp = 56–58 °C, ^1^H NMR (CDCl_3_) ppm: 1.69 (br s, 2H, NH_2_ exch. with D_2_O), 2.49 (t, 2H, *J* = 6.2 Hz), 2.55–2.65 (m, 4H), 2.84 (t, 2H, *J* = 6.0 Hz), 3.11–3.21 (m, 4H), 6.79–6.89 (m, 3H), 7.17–7.27 (m, 2H). Anal. calcd for C_12_H_18_ClN_3_: C, 60.12; H, 7.57; Cl, 14.79; N, 17.53. Found: C, 60.00; H, 7.55; Cl, 14.76; N, 17.49.

#### 4.1.22. 2-(4-(2-Chlorophenyl)piperazin-1-yl)ethyl-1-amine (**32**)

The title compound was prepared from **53** using the general procedure III to afford **32** as a hygroscopic pink solid (0.159 g, 79.71%), after FC purification (CHCl_3_/CH_3_OH 8/2), mp = 98–100 °C, ^1^H NMR (CDCl_3_) ppm: 1.80 (br s, 2H, NH_2_ exch. with D_2_O), 2.51 (t, 2H, *J* = 6.0 Hz), 2.62–2.68 (m, 4H), 2.85 (t, 2H, *J* = 6.0 Hz), 2.99–3.11 (m, 4H), 6.95–7.07 (m, 2H), 7.23 (dd, 1H, *J*_o_ = 8.6 Hz, *J*_m_ = 1.6 Hz), 7.36 (dd, 1H, *J*_o_ = 7.8 Hz, *J*_m_ = 1.4 Hz). Anal. calcd for C_12_H_18_ClN_3_: C, 60.12; H, 7.57; Cl, 14.79; N, 17.53. Found: C, 60.30; H, 7.59; Cl, 14.83; N, 17.58.

#### 4.1.23. 2-(4-(2-Methoxyphenyl)piperazin-1-yl)ethyl-1-amine (**33**)

The title compound was prepared from **54** using the general procedure III to afford **33** as a hygroscopic pink solid (0.111 g, 56.25%), after FC purification (CHCl_3_/CH_3_OH 9/1), mp = 61–64 °C, ^1^H NMR (CDCl_3_) ppm: 1.65 (br s, 2H, NH_2_ exch. with D_2_O), 2.50 (t, 2H, *J* = 6.4 Hz), 2.60–2.70 (m, 4H), 2.84 (t, 2H, *J* = 6.4 Hz), 2.99–3.10 (m, 4H), 3.79 (s, 3H), 6.83–6.89 (m, 1H), 6.90–7.10 (m, 1H). Anal. calcd for C_13_H_21_N_3_O: C, 66.35; H, 8.99; N, 17.86. Found: C, 66.42; H, 9.00; N, 17.88.

#### 4.1.24. 2-(4-(3-Methoxyphenyl)piperazin-1-yl)ethyl-1-amine (**34**)

The title compound was prepared from **55** using the general procedure III to afford **34** as a hygroscopic pink solid (0.130 g, 66.24%), after FC purification (CHCl_3_/CH_3_OH 8/2), mp = 62–65 °C, ^1^H NMR (CDCl_3_) ppm: 1.91 (br s, 2H, NH_2_ exch. with D_2_O), 2.48 (t, 2H, *J* = 5.8 Hz), 2.56–2.63 (m, 4H), 2.84 (t, 2H, *J* = 6.2 Hz), 3.10–3.23 (m, 4H), 3.79 (s, 3H), 6.38–6.50 (m, 2H), 6.54 (d, 1H, *J* = 8.6 Hz), 7.17 (t, 1H, *J* = 8.0 Hz). Anal. calcd for C_13_H_21_N_3_O: C, 66.35; H, 8.99; N, 17.86. Found: C, 66.22; H, 8.97; N, 17.82.

#### 4.1.25. 2-(4-(2-Ethoxyphenyl)piperazin-1-yl)ethyl-1-amine (**35**)

The title compound was prepared from **56** using the general procedure III to afford **35** as a hygroscopic pink solid (0.179 g, 86.57%), after FC purification (CHCl_3_/CH_3_OH 9/1), mp = 55–58 °C, ^1^H NMR (CDCl_3_) ppm: 1.46 (t, 3H, *J* = 7.0 Hz), 1.83 (br s, 2H, NH_2_ exch. with D_2_O), 2.50 (t, 2H, *J* = 6.0 Hz), 2.61–2.70 (m, 4H), 2.85 (t, 2H, *J* = 6.4 Hz), 3.10–3.21 (m, 4H), 4.07 (q, 2H, *J* = 6.8 Hz), 6.82–6.97 (m, 4H). Anal. calcd for C_14_H_23_N_3_O: C, 67.43; H, 9.30; N, 16.85. Found: C, 67.30; H, 9.28; N, 16.82.

#### 4.1.26. 4-(4-(2-Aminoethyl)piperazin-1-yl)benzonitrile (**36**)

The title compound was prepared from **57** using the general procedure III to afford **36** as a hygroscopic yellow solid (0.179 g, 86.98%), after FC purification (CHCl_3_/CH_3_OH 9/1), mp = 72–75 °C, ^1^H NMR (CDCl_3_) ppm: 1.60 (br s, 2H, NH_2_ exch. with D_2_O), 2.52 (t, 2H, *J* = 6.2 Hz), 2.60–2.71 (m, 4H), 2.84 (t, 2H, *J* = 6.0 Hz), 3.20–3.29 (m, 4H), 6.96–7.04 (m, 2H), 7.44–7.59 (m, 2H). Anal. calcd for C_13_H_18_N_4_: C, 67.80; H, 7.88; N, 24.33. Found: C, 67.94; H, 7.90; N, 24.38.

#### 4.1.27. 2-(4-(3,4-Dichlorophenyl)piperazin-1-yl)ethyl-1-amine (**37**)

The title compound was prepared from **58** using the general procedure III to afford **37** as a hygroscopic pink solid (0.108 g, 48.25%), after FC purification (CHCl_3_/CH_3_OH 8/2), mp = 89–92 °C, ^1^H NMR (CDCl_3_) ppm: 1.60 (br s, 2H, NH_2_ exch. with D_2_O), 2.48 (t, 2H, *J* = 6.2 Hz), 2.55–2.65 (m, 4H), 2.84 (t, 2H, *J* = 5.8 Hz), 3.13–3.21 (m, 4H), 6.74 (dd, 1H, *J*_o_ = 8.6 Hz, *J*_m_ = 2.4 Hz), 6.95 (d, 1H, *J* = 2.4 Hz), 7.22–7.30 (m, 1H). Anal. calcd for C_12_H_17_Cl_2_N_3_: C, 52.57; H, 6.25; Cl, 25.86; N, 15.33. Found: C, 52.62; H, 6.26; Cl, 25.89; N, 15.34.

#### 4.1.28. 2-(4-Phenylpiperazin-1-yl)propyl-1-amine (**38**)

The title compound was prepared from **59** using the general procedure III to afford **38** as a white solid (0.109 g, 68.55%), after FC purification (CHCl_3_/CH_3_OH 8/2), mp = 101–103 °C, ^1^H NMR (CDCl_3_) ppm: 1.45 (br s, 2H, NH_2_ exch. with D_2_O), 1.65–1.69 (m, 2H), 2.46 (t, 2H, *J* = 5.6 Hz), 2.59–2.62 (m, 4H), 2.78 (t, 2H, *J* = 5.6 Hz), 3.19–3.21 (m, 4H), 6.85 (t, 1H, *J* = 7.2 Hz), 6.93 (d, 2H, *J* = 7.2 Hz), 7.26 (t, 2H, *J* = 7.6 Hz). Anal. calcd for C_13_H_21_N_3_: C, 71.19; H, 9.65; N, 19.16. Found: C, 71.33; H, 9.67; N, 19.20.

#### 4.1.29. 2-(4-Benzylpiperazin-1-yl)propyl-1-amine (**39**)

The title compound was prepared from **60** using the general procedure III to afford **39** as an oil (0.092 g, 54.78%), after trituration with petroleum ether, ^1^H NMR (CDCl_3_) ppm 1.59–1.66 (m, 4H), 1.59–1.66 (m, 4H), 2.37 (t, 2H, *J* = 5.8 Hz), 2.40–2.47 (m, 6H), 2.73 (t, 2H, *J* = 5.2 Hz), 7.24–7.2316 (m, 5H). Anal. calcd for C_14_H_23_N_3_: C, 72.06; H, 9.93; N, 18.01. Found: C, 72.20; H, 9.95; N, 18.05.

#### 4.1.30. General Procedure IV: Synthesis of carboxamides **2**–**20**

Carboxamides **2**–**20** were prepared starting from the appropriate acid **26**, **28**, or **29** (0.38 mmol) and aryl-piperazin-alkyl amines **30**–**39**, as above reported for compound **1**. The purification of the crude product by FC yielded the desired final compounds **2**–**20**.

#### 4.1.31. 3-Chloro-*N*-(2-(4-phenylpiperazin-1-yl)ethyl)-1,4,5,6-tetrahydrobenzo[6,7]cyclohepta[1,2-*b*]pyrrole-2-carboxamide (**2**)

The title compound was prepared from acid **26** and 2-(4-phenylpiperazin-1-yl)ethyl-1-amine (**30**) using the general procedure IV to afford **2** as a beige solid (0.088 g, 51.72%), after FC purification (CHCl_3_/CH_3_OH 95/5), mp = 201–203 °C, ^1^H NMR (CDCl_3_) ppm: 1.98–2.10 (m, 2H), 2.68–2.80 (m, 10H), 3.20–3.30 (m, 4H), 3.55–3.65 (m, 2H), 6.84–6.90 (m, 1H), 6.95 (d, 1H, *J* = 7.8 Hz), 7.17–7.19 (m, 2H), 7.23–7.31 (m, 3H), 7.48 (d, 1H, *J* = 7.6 Hz), 7.58 (br s, 1H, exch. with D_2_O), 9.26 (br s, 1H, exch. with D_2_O). ^13^C NMR (CDCl_3_) ppm: 25.81 (CH_2_), 26.57 (CH_2_), 34.66 (CH_2_), 36.01 (CH_2_), 49.29 (CH_2_ × 2), 52.67 (CH_2_ × 2), 55.95 (CH_2_), 115.98 (CH × 2), 117.99 (CH × 2), 119.72 (C), 120.96 (CH), 125.01 (C), 126.46 (C), 127.20 (C), 129.08 (CH), 129.60 (CH), 130.30 (C), 141.34 (CH), 141.48 (C), 151.17 (CH), 159.78 (C). MS (ESI): C_26_H_29_ClN_4_O requires *m*/*z* 448.20, found 449.21 [M+H]^+^. Anal. calcd for C_26_H_29_ClN_4_O: C, 69.55; H, 6.51; Cl, 7.90; N, 12.48. Found: C, 69.51; H, 6.50; Cl, 7.89; N, 12.50.

#### 4.1.32. 3-Chloro-*N*-(4-(4-chlorophenyl)piperazin-1-yl)ethyl)-1,4,5,6-tetrahydrobenzo[6,7]cyclohepta[1,2-*b*]pyrrole-2-carboxamide (**3**)

The title compound was prepared from acid **26** and 2-(4-(4-chlorophenyl)piperazin-1-yl)ethyl-1-amine (**31**) using the general procedure IV to afford **3** as a beige solid (0.137 g, 74.71%), after FC purification (CHCl_3_/CH_3_OH 99/1), mp = 193–195 °C, ^1^H NMR (CDCl_3_) ppm: 1.99–2.10 (m, 2H), 2.65–2.79 (m, 10H), 3.14–3.21 (m, 4H), 3.55–3.64 (m, 2H), 6.86 (d, 2H, *J* = 9.0 Hz), 7.17–7.20 (m, 3H), 7.21–7.26 (m, 3H), 7.29–7.32 (m, 1H), 7.47 (d, 1H, *J* = 8.6 Hz), 7.50 (br s, 1H, NH exch. with D_2_O), 9.31 (br s, 1H, exch. with D_2_O). ^13^C NMR (CDCl_3_) ppm: 24.92 (CH_2_), 25.82 (CH_2_), 33.92 (CH_2_), 36.00 (CH_2_), 49.33 (CH_2_ × 2), 52.54 (CH_2_ × 2), 55.95 (CH_2_), 113.63 (C), 117.18 (CH × 2), 118.00 (CH × 2), 120.99 (C), 124.53 (C), 124.83 (C), 126.54 (C), 127.29 (C), 128.91 (CH × 2), 129.66 (C), 130.23 (C), 141.37 (CH), 149.77 (CH), 159.75 (C). MS (ESI): C_26_H_28_Cl_2_N_4_O requires *m*/*z* 482.16; found 483.17 [M+H]^+^. Anal. calcd for C_26_H_28_Cl_2_N_4_O: C, 64.60; H, 5.84; Cl, 14.67; N, 11.59. Found: C, 64.59; H, 5.83; Cl, 14.65; N, 11.48.

#### 4.1.33. 3-Chloro-*N*-(4-(2-chlorophenyl)piperazin-1-yl)ethyl)-1,4,5,6-tetrahydrobenzo[6,7]cyclohepta[1,2-*b*]pyrrole-2-carboxamide (**4**)

The title compound was prepared from acid **26** and 2-(4-(2-chlorophenyl)piperazin-1-yl)ethyl-1-amine (**32**) using the general procedure IV to afford **4** as a beige solid (0.088 g, 48.10%), after FC purification (CHCl_3_/CH_3_OH 99/1), mp = 169–171 °C, ^1^H NMR (CDCl_3_) ppm: 1.99–2.13 (m, 2H), 2.64–2.85 (m, 10H), 3.08–3.14 (m, 4H), 3.54–3.62 (m, 2H), 6.95–7.02 (m, 1H), 7.06 (d, 1H, *J* = 8.0 Hz), 7.08–7.17 (m, 2H), 7.20–7.30 (m, 2H), 7.36 (d, 1H, *J* = 7.8 Hz), 7.49 (d, 1H, *J* = 7.6 Hz), 7.59 (br s, 1H, exch. with D_2_O), 9.33 (br s, 1H, NH exch. with D_2_O). ^13^C NMR (CDCl_3_) ppm: 25.85 (CH_2_), 26.56 (CH_2_), 34.66 (CH_2_), 36.05 (CH_2_), 51.39 (CH_2_ × 2), 52.82 (CH_2_ × 2), 55.96 (CH_2_), 113.57 (C), 120.28 (CH), 120.93 (CH), 120.95 (C), 123.64 (C), 124.96 (C), 126.50 (CH), 127.22 (CH), 127.52 (CH), 128.67 (CH), 129.56 (CH), 129.57 (CH), 130.29 (C), 130.61 (C), 141.33 (C), 149.14 (C), 159.76 (C). MS (ESI): C_26_H_28_Cl_2_N_4_O requires *m*/*z* 482.16; found 483.17 [M+H]^+^. Anal. calcd for C_26_H_28_Cl_2_N_4_O: C, 64.60; H, 5.84; Cl, 14.67; N, 11.59. Found: C, 64.59; H, 5.83; Cl, 14.65; N, 11.48.

#### 4.1.34. 3-Chloro-*N*-(4-(2-methoxyphenyl)piperazin-1-yl)ethyl)-1,4,5,6-tetrahydrobenzo[6,7]cyclohepta[1,2-*b*]pyrrole-2-carboxamide (**5**)

The title compound was prepared from acid **26** and 2-(4-(2-methoxyphenyl)piperazin-1-yl)ethyl-1-amine (**33**) using the general procedure IV to afford **5** as a beige solid (0.145 g, 79.61%), after FC purification (CHCl_3_/CH_3_OH 99/1), mp = 163–165 °C, ^1^H NMR (CDCl_3_) ppm: 1.99–2.10 (m, 2H), 2.65–2.80 (m, 10H), 3.10–3.13 (m, 4H), 3.55–3.63 (m, 2H), 3.88 (s, 3H), 6.88 (d, 1H, *J* = 7.6 Hz), 6.90–7.00 (m, 2H), 7.16–7.20 (m, 2H), 7.21–7.27 (m, 2H), 7.49 (d, 1H, *J* = 7.2 Hz), 7.60 (br s, 1H, NH exch. with D_2_O), 9.33 (br s, 1H, NH exch. with D_2_O). ^13^C NMR (CDCl_3_) ppm: 25.79 (CH_2_), 26.56 (CH_2_), 34.65 (CH_2_), 36.02 (CH_2_), 50.81 (CH_2_ × 2), 52.87 (CH_2_ × 2), 55.29 (CH_3_), 55.96 (CH_2_), 111.79 (CH), 113.59 (C), 118.11 (CH), 120.90 (CH × 2), 121.03 (C × 2), 122.92 (CH), 125.02 (C), 126.46 (CH), 127.17 (C), 129.57 (CH × 2), 130.32 (C), 141.31 (C), 152.15 (C), 159.78 (C). MS (ESI): C_27_H_31_ClN_4_O_2_ requires *m*/*z* 478.21; found 479.22 [M+H]^+^. Anal. calcd for C_27_H_31_ClN_4_O_2_: C, 67.70; H, 6.52; Cl, 7.40; N, 11.70. Found: C, 67.68; H, 6.51; Cl, 7.39; N, 11.68.

#### 4.1.35. 3-Chloro-*N*-(4-(3-methoxyphenyl)piperazin-1-yl)ethyl)-1,4,5,6-tetrahydrobenzo[6,7]cyclohepta[1,2-*b*]pyrrole-2cCarboxamide (**6**)

The title compound was prepared from acid **26** and 2-(4-(3-methoxyphenyl)piperazin-1-yl)ethyl-1-amine (**34**) using the general procedure IV to afford **6** as a beige solid (0.115 g, 63.10%), after FC purification (CHCl_3_/CH_3_OH 98/2), mp = 167–170 °C, ^1^H NMR (CDCl_3_) ppm: 1.89–2.05 (m, 2H), 2.64–2.78 (m, 10H), 3.20–3.25 (m, 4H), 3.52–3.59 (m, 2H), 3.80 (s, 3H), 6.46–6.60 (m, 3H), 7.14–7.35 (m, 4H), 7.46 (s, 1H), 7.50 (br s, 1H, NH exch. with D_2_O), 9.35 (br s, 1H, NH exch. with D_2_O). ^13^C NMR (CDCl_3_) ppm: 25.39 (CH_2_), 26.31 (CH_2_), 34.55 (CH_2_), 35.71 (CH_2_), 48.88 (CH_2_ × 2), 52.33 (CH_2_ × 2), 54.86 (CH_3_), 55.68 (CH_2_), 102.02 (CH), 104.15 (CH), 108.45 (CH), 113.35 (C), 120.62 (CH), 124.82 (CH), 126.19 (C), 126.93 (C), 129.32 (CH × 2), 129.80 (CH), 130.00 (C), 141.02 (C), 152.29 (C), 157.03 (C), 159.44 (C), 160.19 (C). MS (ESI): C_27_H_31_ClN_4_O_2_ requires *m*/*z* 478.21; found 479.22 [M+H]^+^. Anal. calcd for C_27_H_31_ClN_4_O_2_: C, 67.70; H, 6.52; Cl, 7.40; N, 11.70. Found: C, 67.67; H, 6.51; Cl, 7.38; N, 11.69.

#### 4.1.36. 3-Chloro-*N*-(4-(2-ethoxyphenyl)piperazin-1-yl)ethyl)-1,4,5,6-tetrahydrobenzo[6,7]cyclohepta[1,2-*b*]pyrrole-2-carboxamide (**7**)

The title compound was prepared from acid **26** and 2-(4-(2-ethoxyphenyl)piperazin-1-yl)ethyl-1-amine (**35**) using the general procedure IV to afford **7** as a white solid (0.107 g, 57.34%), after FC purification (CHCl_3_/CH_3_OH 98/2), mp = 110–113 °C, ^1^H NMR (CDCl_3_) ppm: 1.47 (t, 3H, *J* = 7.2 Hz), 1.99–2.10 (m, 2H), 2.65–2.81 (m, 10H), 3.14–3.18 (m, 4H), 3.55–3.65 (m, 2H), 4.08 (q, 2H, J = 6.8 Hz), 6.87–6.98 (m, 4H), 7.17–7.19 (m, 2H), 7.25–7.30 (m, 1H), 7.48 (d, 1H, *J* = 8.2 Hz), 7.62 (br s, 1H, NH exch. with D_2_O), 9.30 (br s, 1H, NH exch. with D_2_O). ^13^C NMR (CDCl_3_) ppm: 14.92 (CH_3_), 25.88 (CH_2_), 26.54 (CH_2_), 34.71 (CH_2_), 36.06 (CH_2_), 50.75 (CH_2_ × 2), 52.96 (CH_2_ × 2), 56.05 (CH_2_), 63.54 (CH_2_), 112.50 (CH), 113.63 (C), 118.12 (CH), 120.95 (CH), 121.01 (C), 121.09 (C), 122.73 (CH), 124.85 (CH), 126.55 (CH), 127.25 (CH), 129.46 (C), 129.67 (CH), 130.32 (C), 141.31 (C), 141.37 (C), 151.54 (C), 159.76 (C). MS (ESI): C_28_H_33_ClN_4_O_2_ requires *m*/*z* 492.23, found 493.23 [M+H]^+^. Anal. calcd for C_28_H_33_ClN_4_O_2_: C, 68.21; H, 6.75; Cl, 7.19; N, 11.36. Found: C, 68.23; H, 6.76; Cl, 7.21; N, 11.38.

#### 4.1.37. 3-Chloro-*N*-(2-(4-(2-cyanophenyl)piperazin-1-yl)ethyl)-1,4,5,6-tetrahydrobenzo[6,7]cyclohepta[1,2-*b*]pyrrole-2-carboxamide (**8**)

The title compound was prepared from acid **26** and 2-(4-(2-aminoethyl)piperazin-1-yl)benzonitrile (**36**) using the general procedure IV to afford **8** as a beige solid (0.096 g, 53.42%), after FC purification (CHCl_3_/CH_3_OH 99/1), mp = 171–174 °C, ^1^H NMR (CDCl_3_) ppm: 1.90–2.05 (m, 2H), 2.66–2.80 (m, 10H), 3.25–3.30 (m, 4H), 3.56–3.63 (m, 2H), 6.96–7.05 (m, 1H), 7.16–7.20 (m, 2H), 7.22–7.27 (m, 2H), 7.45–7.47 (m, 3H), 7.59 (br s, 1H, NH exch. with D_2_O), 9.40 (br s, 1H, NH exch. with D_2_O). ^13^C NMR (CDCl_3_) ppm: 25.81 (CH_2_), 26.58 (CH_2_), 34.64 (CH_2_), 36.00 (CH_2_), 51.69 (CH_2_ × 2), 52.62 (CH_2_ × 2), 55.86 (CH_2_), 105.92 (C), 113.63 (C), 118.41 (CH), 118.60 (CH), 120.91 (CH), 120.99 (C), 121.74 (CH), 125.00 (C), 126.49 (C), 127.25 (CH), 129.62 (CH), 130.27 (C), 133.74 (CH), 134.31 (CH), 141.31 (C), 155.54 (C), 159.75 (C). MS (ESI): C_27_H_28_ClN_5_O requires *m*/*z* 473.20; found 474.20 [M+H]^+^. Anal. calcd for C_27_H_28_ClN_5_O: C, 68.42; H, 5.95; Cl, 7.48; N, 14.78. Found: C, 68.43; H, 5.96; Cl, 7.50; N, 14.80.

#### 4.1.38. 3-Chloro-*N*-(4-(3,4-dichlorophenyl)piperazin-1-yl)ethyl)-1,4,5,6-tetrahydrobenzo[6,7]cyclohepta[1,2-*b*]pyrrole-2-carboxamide (**9**)

The title compound was prepared from acid **26** and 2-(4-(3,4-dichlorophenyl)piperazin-1-yl)ethyl-1-amine (**37**) using the general procedure IV to afford **9** as a light brown solid (0.128 g, 65.00%), after FC purification (CHCl_3_/CH_3_OH 99/1), mp = 199–201 °C, ^1^H NMR (CDCl_3_) ppm: 1.99–2.10 (m, 2H), 2.65–2.85 (m, 10H), 3.16–3.25 (m, 4H), 3.56–3.62 (m, 2H), 6.76 (dd, 1H, *J*_o_ = 8.8 Hz, *J*_m_ = 2.8 Hz), 6.94–6.99 (m, 1H), 7.16–7.19 (m, 2H), 7.20–7.31 (m, 2H), 7.46 (s, 1H), 7.50 (br s, 1H, exch. with D_2_O), 9.30 (br s, 1H, NH exch. with D_2_O). ^13^C NMR (CDCl_3_) ppm: 25.81 (CH_2_), 26.56 (CH_2_), 34.65 (CH_2_), 35.98 (CH_2_), 48.84 (CH_2_ × 2), 52.35 (CH_2_ × 2), 55.94 (CH_2_), 113.55 (CH), 115.28 (CH), 117.14 (CH), 118.00 (C), 120.93 (CH), 122.11 (C), 124.97 (C), 126.46 (C), 127.24 (C), 129.63 (CH × 2), 130.27 (C), 130.35 (CH), 132.70 (C), 141.35 (C), 150.53 (C), 159.74 (C). MS (ESI): C_26_H_27_Cl_3_N_4_O requires *m*/*z* 516.13; found 517.13 [M+H]^+^. Anal. calcd for C_26_H_27_Cl_3_N_4_O: C, 60.30; H, 5.26; Cl, 20.54; N, 10.82. Found: C, 60.29; H, 5.25; Cl, 20.53; N, 10.81.

#### 4.1.39. *N*-(3-(4-Phenylpiperazin-1-yl)propyl)-1,4,5,6-tetrahydrobenzo[6,7]cyclohepta[1,2-*b*]pyrrole-2-carboxamide (**10**)

The title compound was prepared from acid **29** and 2-(4-phenylpiperazin-1-yl)propyl-1-amine (**38**) using the general procedure IV to afford **10** as a beige solid (0.039 g, 24.07%), without FC purification, mp = 185–187 °C, ^1^H NMR (CDCl_3_) ppm: 1.55–1.60 (m, 2H), 1.80–1.83 (m, 2H), 1.95–1.99 (m, 2H), 2.61 (t, 2H, *J* = 6.0 Hz), 2.67–2.71 (m, 4H), 2.76–2.78 (m, 2H), 3.26–3.29 (m, 4H), 3.55–3.59 (m, 2H), 6.45 (d, 1H, *J*_m_ = 2.8 Hz), 6.88 (t, 1H, *J* = 7.2 Hz), 6.95 (d, 2H, *J* = 7.6 Hz), 7.14–7.16 (m, 2H), 7.23–7.30 (m, 3H), 7.45 (d, 1H, *J* = 7.6 Hz), 7.51 (br s, 1H, exch. with D_2_O), 9.18 (br s, 1H, exch. with D_2_O). MS (ESI): C_27_H_32_N_4_O requires *m*/*z* 428.26; found 429.26 [M+H]^+^. Anal. calcd for C_27_H_32_N_4_O: C, 75.67; H, 7.53; N, 13.07. Found: C, 75.64; H, 7.51; N, 13.05.

#### 4.1.40. *N*-(3-(4-Benzylpiperazin-1-yl)propyl)-2-chloro-1,4,5,6-tetrahydrobenzo[6,7]cyclohepta[1,2-*b*]pyrrole-2-carboxamide (**11**)

The title compound was prepared from acid **29** and 2-(4-benzylpiperazin-1-yl)propyl-1-amine (**39**) using the general procedure IV to afford **11** as a white solid (0.052 g, 30.92%) after FC purification (CHCl_3_/CH_3_OH 98/2), mp = 103–105 °C, ^1^H NMR (CDCl_3_) ppm: 1.65–1.72 (m, 2H), 1.97–2.03 (m, 2H), 2.47–2.50 (m, 10H), 2.75–2.77 (m, 2H), 2.80–2.84 (m, 2H), 3.42–3.47 (m, 4H), 6.50 (d, 1H, *J*_m_ = 2.8 Hz), 7.08–7.11 (m, 2H), 7.17–7.19 (m, 3H), 7.24–7.26 (m, 3H), 7.41 (d, 1H, *J* = 7.6 Hz), 7.67 (br s, 1H, exch. with D_2_O), 9.18 (br s, 1H, exch. with D_2_O). ^13^C NMR (CDCl_3_) ppm: 24.73 (CH_2_), 27.57 (CH_2_), 27.64 (CH_2_), 34.94 (CH_2_), 39.97 (CH_2_), 53.23 (CH_2_ × 2), 53.28 (CH_2_ × 2), 58.14 (CH_2_), 63.34 (CH_2_), 111.29 (CH), 122.98 (C), 124.87 (CH), 126.07 (C), 126.52 (CH), 126.66 (CH × 2), 127.18 (CH), 128.30 (CH × 2), 129.22 (CH × 2), 129.65 (CH), 130.74 (C), 131.35 (C), 137.93 (C), 141.09 (C), 161.14 (C). MS (ESI): C_28_H_34_N_4_O requires *m*/*z* 442.27; found 443.28 [M+H]^+^. Anal. calcd for C_28_H_34_N_4_O: C, 75.98; H, 7.74; N, 12.66. Found: C, 76.00; H, 7.75; N, 12.68.

#### 4.1.41. 2-Chloro-*N*-(2-(4-phenylpiperazin-1-yl)ethyl)-1,4,5,6-tetrahydrobenzo[6,7]cyclohepta[1,2-*b*]pyrrole-3-carboxamide (**12**)

The title compound was prepared from acid **28** and 2-(4-phenylpiperazin-1-yl)ethyl-1-amine (**30**) using the general procedure IV to afford **12** as a brown solid (0.077 g, 45.16%), after FC purification (CHCl_3_/CH_3_OH 98/2), mp = 167–170 °C, ^1^H NMR (CDCl_3_) ppm: 1.90–2.10 (m, 2H), 2.63–2.80 (m, 8H), 3.10–3.12 (m, 2H), 3.13–3.22 (m, 4H), 3.46–3.58 (m, 2H), 6.90–6.97 (m, 5H, NH exch. with D_2_O), 7.17–7.36 (m, 4H), 8.29 (br s, 1H, NH exch. with D_2_O). ^13^C NMR (CDCl_3_) ppm: 27.53 (CH_2_), 27.82 (CH_2_), 34.71 (CH_2_), 35.83 (CH_2_), 49.19 (CH_2_ × 2), 52.65 (CH_2_ × 2), 56.17 (CH_2_), 115.39 (C), 115.95 (CH × 2), 118.01 (CH), 119.74 (CH), 123.65 (C), 124.34 (CH), 126.19 (CH), 126.40 (C), 127.32 (C), 129.09 (CH × 2), 129.38 (CH), 130.60 (C), 141.28 (C), 151.12 (C), 164.10 (C). MS (ESI): C_26_H_29_ClN_4_O requires *m*/*z* 448.20, found 449.20 [M+H]^+^. Anal. calcd for C_26_H_29_ClN_4_O: C, 69.55; H, 6.51; Cl, 7.90; N, 12.48. Found: C, 69.48; H, 6.52; Cl, 7.87; N, 12.46.

#### 4.1.42. 2-Chloro-*N*-(4-(4-chlorophenyl)piperazin-1-yl)ethyl)-1,4,5,6-tetrahydrobenzo[6,7]cyclohepta[1,2-*b*]pyrrole-3-carboxamide (**13**)

The title compound was prepared from acid **28** and 2-(4-(4-chlorophenyl)piperazin-1-yl)ethyl-1-amine (**31**) using the general procedure IV to afford **13** as a beige solid (0.083 g, 45.10%), after FC purification (CHCl_3_/CH_3_OH 98/2), mp = 200–203 °C, ^1^H NMR (CDCl_3_) ppm: 1.99–2.10 (m, 2H), 2.60–2.65 (m, 6H), 2.70–2.78 (m, 2H), 3.01–3.18 (m, 6H), 3.51–3.62 (m, 2H), 6.85 (d, 4H, *J* = 8.8 Hz), 7.14–7.20 (m, 2H), 7.25–7.31 (m, 3H, NH exch. with D_2_O), 8.32 (br s, 1H, exch. with D_2_O). ^13^C NMR (CDCl_3_) ppm: 27.55 (CH_2_), 27.89 (CH_2_), 34.78 (CH_2_), 35.89 (CH_2_), 49.25 (CH_2_ × 2), 52.60 (CH_2_ × 2), 56.30 (CH_2_), 115.22 (C), 115.61 (C), 117.20 (CH × 2), 123.80 (C), 124.26 (CH), 124.61 (C), 126.30 (C), 126.56 (CH), 127.36 (C), 128.97 (CH × 2), 129.21 (CH), 130.62 (C), 141.40 (C), 149.81 (C), 164.04 (C). MS (ESI): C_26_H_28_Cl_2_N_4_O requires *m*/*z* 482.16; found 483.17 [M+H]^+^. Anal. calcd for C_26_H_28_Cl_2_N_4_O: C, 64.60; H, 5.84; Cl, 14.67; N, 11.59. Found: C, 64.56; H, 5.83; Cl, 14.66; N, 11.56.

#### 4.1.43. 2-Chloro-*N*-(4-(2-chlorophenyl)piperazin-1-yl)ethyl)-1,4,5,6-tetrahydrobenzo[6,7]cyclohepta[1,2-*b*]pyrrole-3-carboxamide (**14**)

The title compound was prepared from acid **28** and 2-(4-(2-chlorophenyl)piperazin-1-yl)ethyl-1-amine (**32**) using the general procedure IV to afford **14** as a beige solid (0.045 g, 24.69.10%), after FC purification (CHCl_3_/CH_3_OH 98/2), mp = 178–180 °C, ^1^H NMR (CDCl_3_) ppm: 1.99–2.10 (m, 2H), 2.60–2.80 (m, 8H), 3.06–3.20 (m, 6H), 3.50–3.60 (m, 2H), 6.94 (br s, 1H, exch. with D_2_O), 6.98–7.02 (m, 4H), 7.15–7.35 (m, 4H), 8.33 (br s, 1H, NH exch. with D_2_O). ^13^C NMR (CDCl_3_) ppm: 27.50 (CH_2_), 27.83 (CH_2_), 34.72 (CH_2_), 35.86 (CH_2_), 49.28 (CH_2_ × 2), 52.69 (CH_2_ × 2), 56.23 (CH_2_), 115.57 (CH), 120.28 (C), 120.93 (CH), 120.95 (C), 123.64 (C), 124.96 (CH), 126.50 (C), 127.22 (CH), 127.52 (CH), 128.67 (CH), 129.56 (CH), 129.57 (CH), 130.29 (C), 130.61 (C), 141.33 (C), 149.14 (C), 159.76 (C). MS (ESI): C_26_H_28_Cl_2_N_4_O requires *m*/*z* 482.16; found 483.17 [M+H]^+^. Anal. calcd for C_26_H_28_Cl_2_N_4_O: C, 64.60; H, 5.84; Cl, 14.67; N, 11.59. Found: C, 64.61; H, 5.85; Cl, 14.64; N, 11.60.

#### 4.1.44. 2-Chloro-*N*-(4-(2-methoxyphenyl)piperazin-1-yl)ethyl)-1,4,5,6-tetrahydrobenzo[6,7]cyclohepta[1,2-b]pyrrole-3-carboxamide (**15**)

The title compound was prepared from acid **28** and 2-(4-(2-methoxyphenyl)piperazin-1-yl)ethyl-1-amine (**33**) using the general procedure IV to afford **15** as a brown solid (0.098 g, 53.93%), after FC purification (CHCl_3_/CH_3_OH 98/2), mp = 88–90 °C, ^1^H NMR (CDCl_3_) ppm: 1.98–2.10 (m, 2H), 2.63–2.80 (m, 8H), 3.00–3.20 (m, 6H), 3.52–3.60 (m, 2H), 3.87 (s, 3H), 6.80–6.96 (m, 5H, NH exch. with D_2_O), 7.12–7.16 (m, 2H), 7.25–7.32 (m, 2H), 8.34 (br s, 1H, NH exch. with D_2_O). ^13^C NMR (CDCl_3_) ppm: 27.53 (CH_2_), 27.83 (CH_2_), 34.71 (CH_2_), 35.84 (CH_2_), 50.73 (CH_2_ × 2), 52.85 (CH_2_ × 2), 55.31 (CH_3_), 56.22 (CH_2_), 11.15 (CH), 115.31 (CH), 115.53 (CH), 120.88 (CH), 120.90 (C), 122.95 (CH), 123.63 (CH), 124.26 (C), 126.19 (C), 126.38 (C), 127.26 (CH), 129.39 (C), 130.61 (CH), 141.14 (C), 141.30 (C), 152.18 (C), 164.04 (C). MS (ESI): C_27_H_31_ClN_4_O_2_ requires *m*/*z* 478.21; found 479.22 [M+H]^+^. Anal. calcd for C_27_H_31_ClN_4_O_2_: C, 67.70; H, 6.52; Cl, 7.40; N, 11.70. Found: C, 67.69; H, 6.51; Cl, 7.41; N, 11.72.

#### 4.1.45. 2-Chloro-*N*-(4-(3-methoxyphenyl)piperazin-1-yl)ethyl)-1,4,5,6-tetrahydrobenzo[6,7]cyclohepta[1,2-*b*]pyrrole-3-carboxamide (**16**)

The title compound was prepared from acid **28** and 2-(4-(3-methoxyphenyl)piperazin-1-yl)ethyl-1-amine (**34**) using the general procedure IV to afford **16** as a brown solid (0.104 g, 57.14%), after FC purification (CHCl_3_/CH_3_OH 98/2), mp = 89–92 °C, ^1^H NMR (CDCl_3_) ppm: 1.99–2.10 (m, 2H), 2.60–2.80 (m, 8H), 3.00–3.20 (m, 6H), 3.50–3.58 (m, 2H), 3.80 (s, 3H), 6.40–6.54 (m, 3H), 6.90 (br s, 1H, NH exch. with D_2_O), 7.13–7.36 (m, 5H), 8.61 (br s, 1H, NH exch. with D_2_O). ^13^C NMR (CDCl_3_) ppm: 27.53 (CH_2_), 27.79 (CH_2_), 34.70 (CH_2_), 35.81 (CH_2_), 49.08 (CH_2_ × 2), 52.57 (CH_2_ × 2), 55.14 (CH_3_), 56.12 (CH_2_), 102.43 (CH), 104.37 (CH), 108.79 (CH), 115.32 (CH × 2), 123.62 (CH), 124.42 (CH), 126.17 (C), 126.37 (C), 127.35 (C), 129.35 (C), 129.76 (CH), 130.63 (C), 141.25 (C), 152.53 (C), 160.47 (C), 164.15 (C). MS (ESI): C_27_H_31_ClN_4_O_2_ requires *m*/*z* 478.21; found 479.22 [M+H]^+^. Anal. calcd for C_27_H_31_ClN_4_O_2_: C, 67.70; H, 6.52; Cl, 7.40; N, 11.70. Found: C, 67.68; H, 6.51; Cl, 7.39; N, 11.68.

#### 4.1.46. 2-Chloro-*N*-(4-(2-ethoxyphenyl)piperazin-1-yl)ethyl)-1,4,5,6-tetrahydrobenzo[6,7]cyclohepta[1,2-*b*]pyrrole-3-carboxamide (**17**)

The title compound was prepared from acid **28** and 2-(4-(2-ethoxyphenyl)piperazin-1-yl)ethyl-1-amine (**35**) using the general procedure IV to afford **17** as a brown solid (0.008 g, 43.09%), after FC purification (CHCl_3_/CH_3_OH 98/2), mp = 150–152 °C, ^1^H NMR (CDCl_3_) ppm: 1.46 (t, 3H, J = 6.8 Hz), 1.99–2.10 (m, 2H), 2.61–2.80 (m, 8H), 3.00–3.20 (m, 6H), 3.47–3.58 (m, 2H), 4.07 (q, 2H, *J* = 7.0 Hz), 6.86–6.98 (m, 5H, NH exch. with D_2_O), 7.14–7.18 (m, 2H), 7.20–7.33 (m, 2H), 8.33 (br s, 1H, NH exch. with D_2_O). ^13^C NMR (CDCl_3_) ppm: 14.91 (CH_3_), 27.53 (CH_2_), 27.82 (CH_2_), 34.73 (CH_2_), 35.85 (CH_2_), 50.66 (CH_2_ × 2), 52.90 (CH_2_ × 2), 56.23 (CH_2_), 63.50 (CH_2_), 112.40 (CH), 115.32 (CH), 115.55 (CH), 120.87 (CH), 122.74 (CH), 123.65 (CH), 124.17 (C), 124.25 (C), 126.19 (CH), 126.39 (C), 127.24 (C), 129.39 (C), 130.62 (CH), 141.19 (C), 141.30 (C), 151.47 (C), 164.02 (C). MS (ESI): C_28_H_33_ClN_4_O_2_ requires *m*/*z* 492.23, found 493.23 [M+H]^+^. Anal. calcd for C_28_H_33_ClN_4_O_2_: C, 68.21; H, 6.75; Cl, 7.19; N, 11.36. Found: C, 68.19; H, 6.74; Cl, 7.18; N, 11.34.

#### 4.1.47. 2-Chloro-*N*-(2-(4-(2-cyanophenyl)piperazin-1-yl)ethyl)-1,4,5,6-tetrahydrobenzo[6,7]cyclohepta[1,2-*b*]pyrrole-3-carboxamide (**18**)

The title compound was prepared from acid **28** and 2-(4-(2-aminoethyl)piperazin-1-yl)benzonitrile (**36**) using the general procedure IV to afford **18** as a beige solid (0.087 g, 48.46%), after FC purification (CHCl_3_/CH_3_OH 98/2), mp = 87–90 °C, ^1^H NMR (CDCl_3_) ppm: 1.99–2.10 (m, 2H), 2.65–2.81 (m, 8H), 3.11 (t, 2H, *J* = 7.0 Hz), 3.15–3.27 (m, 4H), 3.54–3.60 (m, 2H), 6.83 (br s, 1H, NH exch. with D_2_O), 6.96–7.10 (m, 2H), 7.14–7.20 (m, 2H), 7.22–7.31 (m, 2H), 7.42–7.61 (m, 2H), 8.36 (br s, 1H, NH exch. with D_2_O). ^13^C NMR (CDCl_3_) ppm: 27.30 (CH_2_), 27.84 (CH_2_), 34.74 (CH_2_), 35.20 (CH_2_), 51.52 (CH_2_ × 2), 52.66 (CH_2_ × 2), 56.21 (CH_2_), 105.83 (CH), 115.19 (CH), 15.28 (CH), 118.59 (CH), 121.77 (C), 123.71 (CH), 124.23 (C), 126.22 (C), 126.26 (C), 127.29 (CH), 128.56 (CH), 130.58 (CH), 133.77 (C), 134.34 (C), 141.34 (C), 155.44 (C), 164.02 (C). MS (ESI): C_27_H_28_ClN_5_O requires *m*/*z* 473.20, found 471.20 [M+H]^+^. Anal. calcd for C_27_H_28_ClN_5_O: C, 68.42; H, 5.95; Cl, 7.48; N, 14.78. Found: C, 68.40; H, 5.94; Cl, 7.47; N, 14.76.

#### 4.1.48. 2-Chloro-*N*-(4-(3,4-dichlorophenyl)piperazin-1-yl)ethyl)-1,4,5,6-tetrahydrobenzo[6,7]cyclohepta[1,2-*b*]pyrrole-3-carboxamide (**19**)

The title compound was prepared from acid **28** and 2-(4-(3,4-dichlorophenyl)piperazin-1-yl)ethyl-1-amine (**37**) using the general procedure IV to afford **19** as a grey solid (0.036 g, 18.46%), after FC purification (CHCl_3_/CH_3_OH 99/1), mp = 213–216 °C, ^1^H NMR (CDCl_3_) ppm: 1.90–2.10 (m, 2H), 2.30–2.40 (m, 2H), 2.63–2.68 (m, 4H), 2.73–2.80 (m, 2H), 2.85–2.95 (m, 2H), 3.00–3.18 (m, 4H), 3.45–3.58 (m, 2H), 6.06 (br s, 1H, NH exch. with D_2_O), 6.69–6.79 (m, 3H), 7.93–7.17 (m, 2H), 7.18–7.34 (m, 2H), 8.58 (br s, 1H, exch. with D_2_O). ^13^C NMR (CDCl_3_) ppm: 25.83 (CH_2_), 26.58 (CH_2_), 34.67 (CH_2_), 36.01 (CH_2_), 48.87 (CH_2_ × 2), 52.34 (CH_2_ × 2), 55.92 (CH_2_), 113.52 (CH), 115.25 (CH), 117.10 (CH), 118.12 (C), 121.93 (CH), 122.21 (C), 124.94 (C), 126.47 (C), 127.32 (C), 129.62 (CH × 2), 130.29 (C), 131.85 (CH), 132.65 (C), 140.96 (C), 151.52 (C), 158.06 (C). MS (ESI): C_26_H_27_Cl_3_N_4_O requires *m*/*z* 516.13; found 517.13 [M+H]^+^. Anal. calcd for C_26_H_27_Cl_3_N_4_O: C, 60.30; H, 5.26; Cl, 20.54; N, 10.82. Found: C, 60.33; H, 5.27; Cl, 20.55; N, 10.83.

#### 4.1.49. *N*-(3-(4-Benzylpiperazin-1-yl)propyl)-2-chloro-1,4,5,6-tetrahydrobenzo[6,7]cyclohepta[1,2-*b*]pyrrole-3-carboxamide (**20**)

The title compound was prepared from acid **28** and 2-(4-benzylpiperazin-1-yl)propyl-1-amine (**39**) using the general procedure IV to afford **20** as a beige solid (0.083 g, 45.81%), after FC purification (CHCl_3_/CH_3_OH 95/5), mp = 169–171 °C, ^1^H NMR (CDCl_3_) *δ*: ^1^H NMR (CDCl_3_) ppm: 1.65–1.72 (m, 2H), 1.97–2.03 (m, 2H), 2.47–2.50 (m, 10H), 2.75–2.77 (m, 2H), 2.80–2.84 (m, 2H), 3.42–3.47 (m, 4H), 6.50 (d, 1H, *J*_m_ = 2.8 Hz), 7.08–7.11 (m, 3H), 7.17–7.19 (m, 2H), 7.24–7.26 (m, 2H), 7.41 (d, 1H, *J* = 7.6 Hz), 7.67 (br s, 1H, exch. with D_2_O), 9.18 (br s, 1H, exch. with D_2_O). ^13^C NMR (CDCl_3_) ppm: 25.97 (CH_2_), 27.35 (CH_2_), 27.71 (CH_2_), 34.84 (CH_2_), 38.52 (CH_2_), 52.82 (CH_2_ × 2), 53.14 (CH_2_ × 2), 56.74 (CH_2_), 63.02 (CH_2_), 114.75 (C), 116.42 (C), 123.56 (C), 124.13 (CH), 126.31 (CH), 126.52 (CH), 127.09 (CH), 127.24 (C), 128.22 (CH × 2), 129.29 (CH × 2), 129.40 (CH), 130.66 (C), 137.81 (C), 141.38 (C), 164.11 (C). MS (ESI): C_28_H_33_ClN_4_O requires *m*/*z* 476.23, found 477.23 [M+H]^+^. Anal. calcd for C_28_H_33_ClN_4_O: C, 70.50; H, 6.97; Cl, 7.43; N, 11.74. Found: C, 70.47; H, 6.95; Cl, 7.1; N, 11.73.

### 4.2. Biological Methods

#### 4.2.1. Cell Culture

Huh-7 [20] and Vero E6 cells were cultured in DMEM (Gibco by ThermoFisher Scientific, Milano, Italy), 1 mM glutamine, 1 mM sodium pyruvate, and 7% fetal bovine serum (FBS, Gibco). Vero-TMPRSS [21] were cultured like Vero-E6 cells, with 1 mg/mL of G418 (Merck®, Darmstadt, Germany) added once a month. MDCK-NBL-2 cells (ATCC CCL-34) were grown in Ex-Cell serum-free medium (Merck®, Darmstadt, Germany), 1 mM glutamine, and 1 μg/mL di-TPCK trypsin (Merck®, Darmstadt, Germany). All cells were cultured without antibiotics and checked for *Mycoplasma* as reported in the literature [22].

#### 4.2.2. Preparation of Compounds

All compounds were stored at 10 mM in DMSO at −20 °C for 3 months. For longer storage, aliquots were kept at −80 °C.

#### 4.2.3. Viruses

ZIKV, strain Brazil/2016/INMI1 (ZIKV^Br^) (family Flaviviridae, genus Orthoflavivirus, National Institute for Infectious Diseases Spallanzani, Roma, Italy) was propagated on Huh-7 cells. IAV (family Orthomyxoviridae, genus Alphainfluenzavirus, species Alphainfluenzavirus influenzae) strain A/PR/8/34 (ATCC) was propagated on MDCK cells in Excel medium (Merck®, Darmstadt, Germany), 1 μg/mL of TPCK trypsin (Merck®, Darmstadt, Germany) with no serum. SARS-CoV-2 VR PV10734 (GISAID EPI_ISL_2544194) (family Coronaviridae, genus Betacoronavirus, species Betacoronavirus pandemicum, SARS-CoV2-Mi), courtesy of Università San Raffaele, Milan, Italy, was propagated on Vero-TMPRSS cells.

#### 4.2.4. Determination of Compound Cytotoxicity and Cytotoxic Concentration 50 (CC_50_)

Cytotoxic effects were evaluated on uninfected cells using the WST-8 assay (Orangu, Cell Guidance Systems, Cambridge, UK) and the crystal violet assay [23]. Cells were seeded in 96-well plates and treated with 10- or 3-fold serial dilutions of the compounds (from 0.5 to 50 μM for initial screening; from 0.25 to 180 μM for CC_50_ determination of the most promising compounds). Huh-7, MDCK, and Vero TMPRSS cells were seeded at 1 × 10^4^, 1.5 × 10^4^, and 1.2 × 10^4^ cells/well, respectively, and incubated for 48 h (Huh-7 and MDCK) or 24 h (VERO TMPRSS), following conditions matching those of the corresponding antiviral assays in terms of timing and culture medium. At the end of incubation, the medium was replaced with a 10% WST-8 solution in DMEM and incubated for 1 h at 37 °C. Optical density (OD) was then measured at 450 nm using a Varioskan LUX plate reader (ThermoFisher Scientific, Milano, Italy). Subsequently, cells were fixed with 4% buffered formalin solution (Merck®, Darmstadt, Germany), stained with 1% crystal violet (Merck®, Darmstadt, Germany), and the dye was solubilized in 30% acetic acid for 30 min under shaking. OD was then measured at 595 nm as an additional readout of cell viability. The percentage of viability at each concentration was calculated using the formula:

% viability = 100 × (ODcompound/ODuntreated control)

where ODcompound and ODuntreated are the OD of the cells treated with compound or DMSO, respectively.

The CC_50_ value was determined by nonlinear regression analysis on GraphPad Prism 7 (San Diego, CA, USA).

#### 4.2.5. Determination of Viral Yield Reduction and Effective Concentration 50 (EC_50_)

Viral yield reduction assays for ZIKV and SARS-CoV-2 were performed as previously described by Plicanti [24]. Briefly, 1 × 10^4^ Huh-7 cells/well and 1.2 × 10^4^ VERO TMPRSS cells/well were infected with ZIKV (MOI 1) or SARS-CoV-2 (MOI 0.05) in a primary 96-well plate for 2 h at 37 °C, 5% CO_2_. Following inoculum removal, cells were treated with serial dilutions of the compounds (from 0.5 to 50 μM for initial screening; from 0.12 to 180 μM for EC_50_ determination of the most promising compounds) in DMEM with 2% FBS. Supernatants were collected after 48 h (Huh-7) or 24 h (VERO TMPRSS), diluted 3-fold, and added onto VERO E6 or VERO TMPRSS monolayers, respectively. After 2 h at 37 °C, the inoculum was replaced with 0.75% carboxymethyl cellulose (Merck®, Darmstadt, Germany) in DMEM 2% FBS. After 48–72 h, cells were then fixed with 4% buffered formalin solution (Merck®, Darmstadt, Germany) and stained with 1% crystal violet (Merck®, Darmstadt, Germany). Titres and percentage of inhibition were calculated as follows:

PFU/mL = number of plaques*3^n/vol infection, where n is the dilution where plaques were counted:

% inhibition = 100 × (1 − Titre-compound/Titre-untreated control)

where Titre-compound and Titre-untreated control are the titres (PFU/mL) obtained on cells treated with compound or DMSO, respectively.

For IAV, viral yield was assessed by determining Tissue Culture Infectious Dose (TCID)50/mL, as previously described [25]. An amount of 1.5 × 10^4^ MDCK cells/well were infected with IAV at 10^3^ TCID_50_/mL in a 96-well plate and incubated for 2 h at 37 °C, 5% CO_2_. Following inoculum removal, cells were treated with serial dilutions of the compounds, prepared in Ex-Cell medium. Supernatants were collected at 48 hpi, diluted 5-fold, and used to infect fresh MDCK monolayers. After 96 h, cells were fixed and stained with crystal violet. Viral titre reduction was calculated using the Reed and Muench method [26].

The EC_50_ value was determined by nonlinear regression analysis on GraphPad Prism (San Diego, CA, USA).

SI was obtained by dividing CC_50_ by EC_50_ for each drug/virus/cell line. Compounds with SI values ≥ 10 were active in vitro.

## 5. Conclusions

The preliminary data on anticancer and antiviral activity of these novel carboxamides allowed us to identify compound **1**, which did not show any significant antiviral effect in our assays, as the most promising for the development of analogues with anti-tumour activity. At the same time, piperazine derivatives have also proven to be more interesting for their antiviral activity, particularly compound **13**, identified as a reference for the development of novel antiviral agents.

Further studies will be conducted to identify the targets of the anti-tumour and antiviral action of these new molecules, as well as to deepen SAR studies.

## Figures and Tables

**Table 1:** Arylpiperazinyl-benzocycloheptapyrrole-carboxamides **1**–**20**.

Compound	Structure	n	m	Ar
**1**	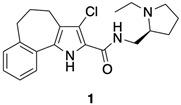	-	-	-
**2**	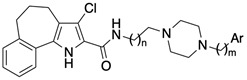	1	-	Ph
**3**		1	-	4-Cl-Ph
**4**		1	-	2-Cl-Ph
**5**		1	-	2-OCH_3_-Ph
**6**		1	-	3-OCH_3_-Ph
**7**		1	-	2-OC_2_H_5_-Ph
**8**		1	-	2-CN-Ph
**9**		1	-	3,4-Cl-Ph
10 *		2	-	Ph
11 *		2	1	Ph
**12**	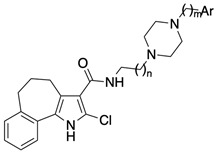	1	-	Ph
**13**		1	-	4-Cl-Ph
**14**		1	-	2-Cl-Ph
**15**		1	-	2-OCH_3_-Ph
**16**		1	-	3-OCH_3_-Ph
**17**		1	-	2-OC_2_H_5_-Ph
**18**		1	-	2-CN-Ph
**19**		1	-	3,4-Cl-Ph
**20**		2	1	Ph

* Compounds **10** and **11** do not contain the chlorine atom on the pyrrole ring.

**Table 2:** Percentage of growth inhibition (GI%) and cytotoxicity (negative values) of in vitro leukemia cell lines for compounds **1**, **5**, **7**, **10**, **11**, **12**, **13**, **14**, **15**, **16**, **17**, **18**, and **20** at 10^−5^ M (single-dose assay).

Leukemia Cell Line	Compound ^a^												
	1	5	7	10	11	12	13	14	15	16	17	18	20
CCRF-CEM	75.48	71.64	–	61.17	76.14	–	–	–	–	–	61.58	–	–
HL-60(TB)	−38.55	−8.91	–	−30.92	−26.72	67.22	69.56	71.71	86.56	–	−8.21	74.34	−23.10
K-562	93.98	95.81	82.58	99.43	−15.60	70.87	70.88	70.31	92.73	77.59	99.95	75.26	–
MOLT-4	96.19	92.98	66.54	–	99.74	–	61.66	–	76.47	–	95.53	81.80	–
RPMI-8226	98.61	– ^b^	63.39	–	92.04	–	67.38	60.85	70.12	60.02	–	69.13	−1.74
SR	96.28	90.09	70.00	−19.75	−11.99	78.66	70.73	–	95.50	73.62	−5.19	74.20	99.43

^a^ Compound **8** showed only a low GI% activity (62.37%) against the K-562 cell line. ^b^ GI% values < 60% and no cytotoxicity.

**Table 3:** Percentages of growth inhibition (GI%) and cytotoxicity (negative values) of in vitro colon cancer cell lines for compounds **1**, **5**, **7**, **10**, **11**, **12**, **13**, **14**, **15, 16**, **17**, **18**, and **20** at 10^−5^ M (single-dose assay).

Colon Cancer/ Cell Line	Compound ^a^												
	1	5	7	10	11	12	13	14	15	16	17	18	20
COLO 205	−52.22	−48.74	88.80	−58.03	−58.61	–	–	–	–	–	−47.24	–	−59.92
HCC-2998	−21.01	– ^b^	–	−9.32	−49.20	–	–	–	−18.77	–	−35.63	–	−91.84
HCT-116	−25.27	94.64	76.06	95.67	96.84	65.78	–	66.46	65.19	–	88.92	70.10	95.51
HCT-15	−52.66	92.46	82.07	91.24	84.78	–	–	–	66.42	–	81.00	61.27	99.04
HT29	−32.25	−17.44	72.69	−2.47	91.03	65.47	69.21	–	79.37	74.91	81.46	64.18	94.66
KM12	83.83	67.68	–	73.72	78.00	–	–	–	–	–	–	–	70.31
SW-620	−30.78	67.43	–	80.14	86.67	–	–	–	60.06	–	–	–	−40.23

^a^ Compound **6** showed very low activity (63.97%) against the HCT-116 cell line. ^b^ GI% values < 60% and no cytotoxicity.

**Table 4:** Percentages of growth inhibition (GI%) and cytotoxicity (negative values) of in vitro melanoma cell lines for compounds **1**, **5**, **10**, **11**, **12**, **13**, **14**, **15, 17**, **18**, **19**, and **20** at 10^−5^ M (single-dose assay).

Melanoma/ Cell Line	Compound ^a^											
	1	5	10	11	12	13	14	15	17	18	19	20
LOX IMVI	−25.00	82.82	95.25	99.73	–	–	–	60.57	74.35	–	–	−69.19
MALME-3M	−90.67	– ^b^	−72.64	−88.18	–	–	–	70.10	90.33	–	–	−84.03
M14	−68.50	−22.16	−59.25	−56.04	–	–	–	84.85	−7.74	–	–	−69.37
MDA-MB-435	−78.15	61.84	75.11	−20.99	–	–	–	–	69.31	64.54	–	−21.33
SK-MEL-2	85.81	–	74.95	−78.42	–	–	–	–	–	–	–	−52.93
SK-MEL-28	−85.77	–	−29.31	−73.62	–	–	–	–	71.48	–	–	−88.58
SK-MEL-5	−88.03	−62.59	−72.58	−94.54	−7.83	−21.64	−3.84	−75.22	−88.73	−67.02	−24.28	−89.68
UACC-257	−55.18	–	−8.50	−62.94	–	–	–	66.95	72.00	–	–	−58.02
UACC-62	−73.43	–	–	−22.97	–	–	–	–	–	–	–	−59.22

^a^ Compounds **7**, **8**, and **16** showed moderate to high GI% (77.71%, 75.28%, and 88.63%, respectively) against the SK-MEL-5 cell line, and compound **7** also had a high GI% value (88.63%) against the M14 cell line. ^b^ GI% values < 60% and no cytotoxicity.

**Table 5:** Percentages of growth inhibition (GI%) and cytotoxicity (negative values) of in vitro non-small cell lung cancer panel for compounds **1**, **5**, **10**, **11**, **15**, **17**, **18**, and **20** at 10^−5^ M (single-dose assay).

Non-Small Cell Lung Cancer Cell Line ^a^	Compound ^b^							
	1	5	10	11	15	17	18	20
EKVX	68.77	94.23	74.18	70.83	81.68	87.53	92.82	77.16
NCI-H226	– ^c^	–	75.15	64.87	–	60.44	–	–
NCI-H23	–	62.21	–	–	–	–	–	60.20
NCI-H460	−54.58	79.16	90.73	98.58	–	69.97	–	83.11
NCI-H522	73.49	85.46	71.42	82.24		65.65		92.20

^a^ Compounds showed no activity against A549/ATCC and HOP-62 cell lines. ^b^ Compound **7** showed only low GI% values (60.76%) against the NCI-H460 cell line, whereas derivatives **12, 14**, and **19** showed low to moderate GI% values (72.30%, 72.65%, and 61.72%, respectively) only against the EKVX cell line. ^c^ GI% values < 60% or no cytotoxicity.

**Table 6:** Percentages of in vitro growth inhibition (GI%) and cytotoxicity (negative values) of in vitro breast cancer panel cell line panel for compounds **1**, **10**, **11**, **13, 14**, **15**, **17**, **18**, **19**, and **20** at 10^−5^ M (single-dose assay).

Breast Cancer/ Cell Line ^a^	Compound ^b^									
	1	10	11	13	14	15	17	18	19	20
MCF-7	87.23	80.23	90.34	–	–	72.98	82.21	78.50	–	95.41
MDA-MB-231/ATCC	88.97	– ^c^	61.71	–	–	–	–	–	–	62.65
BT-549	62.87	–	–	–	–	66.54	80.67	78.35	–	68.37
T-47D	66.22	65.83	66.07				77.59	71.83	–	78.55
MDA-MB-468	−3.75	−13.75	−52.92	95.28	82.38	94.75	−3.78	98.72	94.16	−29.55

^a^ Compound **20** was the only one to show a moderate GI% value (72.36%) against the HS%/(T cell line). All the other compounds showed GI% values < 60% and no cytotoxicity. ^b^ Compounds **4**, **12**, and **16** showed a low to moderate GI% values (60.02%, 60.88%, and 72.98%, respectively) against the MCF7 cell line. ^c^ GI% values < 60% and no cytotoxicity.

**Table 7:** Percentage of growth inhibition (GI%) and cytotoxicity (negative values) of in vitro renal tumour cell line panel for compounds **1**, **5**, **10**, **11**, **15**, **17**, **18**, and **20** at 10^−5^ M (single-dose assay).

Renal Cancer/ Cell Line ^a^	Compound ^b^							
	1	5	10	11	15	17	18	20
786-0	−22.61	62.99	68.72	72.88	–	–	–	87.02
CAKI-1	−27.03	77.87	89.42	91.53	61.00	71.64	62.71	84.69
RXF 393	79.67	89.42	−8.69	−9.45	–	62.07	–	78.18
SN12C	– ^c^	71.82	–	–	–	60.70	–	–
UO-31	83.00	69.99	–	63.12	63.59	69.28	67.05	65.86

^a^ Compounds showed no activity against A489 and ACHN cell lines. ^b^ Compound **14** showed only a low GI% value (63.59%) against the UO-31 cell line. ^c^ GI% values < 60% and no cytotoxicity.

**Table 8:** Growth inhibition 50 (GI_50_) values of in vitro leukemia, colon, and melanoma tumour cell line panels for compounds **1**, **10**, **11**, and **20** at five doses (10^−4^–10^−8^ M).

Panel/Cell Line	Compound			
	1	10	11	20
**Leukemia**				
CCRF-CEM	5.96 × 10^−6^	8.21 × 10^−6^	3.89 × 10^−6^	1.07 × 10^−5^
HL-60(TB)	2.60 × 10^−6^	5.58 × 10^−6^	2.92 × 10^−6^	5.06 × 10^−6^
K-562	1.76 × 10^−6^	2.58 × 10^−6^	2.57 × 10^−6^	2.97 × 10^−6^
MOLT-4	3.57 × 10^−6^	4.97 × 10^−6^	2.69 × 10^−6^	4.78 × 10^−6^
RPMI-8226	2.29 × 10^−6^	3.00 × 10^−6^	2.20 × 10^−6^	3.69 × 10^−6^
SR	2.29 × 10^−6^	2.29 × 10^−6^	2.49 × 10^−6^	0.53 × 10^−6^
**Colon Cancer**				
COLO 205	1.80 × 10^−6^	1.92 × 10^−6^	1.65 × 10^−6^	1.96 × 10^−6^
HCC-2998	2.98 × 10^−6^	2.51 × 10^−6^	1.73 × 10^−6^	1.77 × 10^−6^
HCT-116	3.12 × 10^−6^	4.83 × 10^−6^	3.37 × 10^−6^	5.50 × 10^−6^
HCT-15	2.31 × 10^−6^	4.71 × 10^−6^	3.65 × 10^−6^	7.78 × 10^−6^
HT29	1.86 × 10^−6^	3.14 × 10^−6^	2.60 × 10^−6^	4.03 × 10^−6^
KM12	3.02 × 10^−6^	4.89 × 10^−6^	4.11 × 10^−6^	9.38 × 10^−6^
SW-620	1.75 × 10^−6^	8.89 × 10^−6^	3.57 × 10^−6^	3.59 × 10^−6^
**Melanoma**				
LOX IMVI	1.76 × 10^−6^	3.62 × 10^−6^	2.16 × 10^−6^	4.33 × 10^−6^
MALME-3M	1.54 × 10^−6^	1.65 × 10^−6^	1.90 × 10^−6^	2.02 × 10^−6^
M14	2.03 × 10^−6^	2.78 × 10^−6^	1.83 × 10^−6^	2.01 × 10^−6^
MDA-MB-435	1.76 × 10^−6^	7.88 × 10^−6^	2.59 × 10^−6^	4.90 × 10^−6^
SK-MEL-2	9.38 × 10^−6^	1.49 × 10^−5^	7.85 × 10^−6^	1.37 × 10^−5^
SK-MEL-28	1.87 × 10^−6^	2.51 × 10^−6^	1.75 × 10^−6^	2.11 × 10^−6^
SK-MEL-5	1.68 × 10^−6^	2.71 × 10^−6^	1.72 × 10^−6^	1.79 × 10^−6^
UACC-257	1.88 × 10^−6^	2.29 × 10^−6^	1.74 × 10^−6^	1.87 × 10^−6^
UACC-62	1.87 × 10^−6^	9.02 × 10^−6^	2.55 × 10^−6^	6.93 × 10^−6^

**Table 9:** GI_50_ mean graph midpoint (MG_MID, molar) for NCI cancer panel ^a^ for compounds **1**, **10**, **11**, and **20**.

Panel/Cell Line	Compound			
	1	10	11	20
Leukemia	2.81 × 10^−6^	2.95 × 10^−6^	2.88 × 10^−6^	3.39 × 10^−6^
Non-Small Cell Lung Cancer	7.24 × 10^−6^	9.33 × 10^−6^	7.08 × 10^−6^	10.47 × 10^−6^
Colon Cancer	2.34 × 10^−6^	3.98 × 10^−6^	2.81 × 10^−6^	4.17 × 10^−6^
CNS Cancer	4.79 × 10^−6^	16.98 × 10^−6^	5.25 × 10^−6^	12.02 × 10^−6^
Melanoma	2.14 × 10^−6^	4.07 × 10^−6^	2.34 × 10^−6^	3.39 × 10^−6^
Ovarian Cancer	2.29 × 10^−6^	12.30 × 10^−6^	7.94 × 10^−6^	12.02 × 10^−6^
Renal Cancer	5.25 × 10^−6^	8.71 × 10^−6^	5.89 × 10^−6^	9.12 × 10^−6^
Prostate Cancer	8.13 × 10^−6^	6.61 × 10^−6^	5.13 × 10^−6^	9.55 × 10^−6^
Breast Cancer	4.47 × 10^−6^	8.32 × 10^−6^	3.47 × 10^−6^	6.92 × 10^−6^
MG_MID	4.26 × 10^−6^	7.24 × 10^−6^	4.47 × 10^−6^	6.76 × 10^−6^

^a^ Each cancer type represents the average of two to nine cancer cell lines.

**Table 10:** TGI ^a^ and LC_50_ ^b^ values of compounds **1**, **10**, **11**, and **20**.

Melanoma/Cell Line	Compound							
	1		10		11		20	
	TGI	LC_50_	TGI	LC_50_	TGI	LC_50_	TGI	LC_50_
LOX IMVI	3.39 × 10^−6^	6.31 × 10^−6^	19.50 × 10^−6^	85.11 × 10^−6^	5.13 × 10^−6^	15.85 × 10^−6^	15.85 × 10^−6^	40.74 × 10^−6^
MALME-3M	2.95 × 10^−6^	5.62 × 10^−6^	4.36 × 10^−6^	15.49 × 10^−6^	3.48 × 10^−6^	6.46 × 10^−6^	3.98 × 10^−6^	7.76 × 10^−6^
M14	4.36 × 10^−6^	9.12 × 10^−6^	8.51 × 10^−6^	44.67 × 10^−6^	3.55 × 10^−6^	6.92 × 10^−6^	4.17 × 10^−6^	8.71 × 10^−6^
MDA-MB-435	3.55 × 10^−6^	5.62 × 10^−6^	18.62 × 10^−6^	40.74 × 10^−6^	7.41 × 10^−6^	30.20 × 10^−6^	17.78 × 10^−6^	48.98 × 10^−6^
SK-MEL-2	21.34 × 10^−6^	46.77 × 10^−6^	34.67 × 10^−6^	81.28 × 10^−6^	20.89 × 10^−6^	47.86 × 10^−6^	26.91 × 10^−6^	53.70 × 10^−6^
SK-MEL-28	7.71 × 10^−6^	7.24 × 10^−6^	8.51 × 10^−6^	29.51 × 10^−6^	3.31 × 10^−6^	6.16 × 10^−6^	4.90 × 10^−6^	13.18 × 10^−6^
SK-MEL-5	3.09 × 10^−6^	5.62 × 10^−6^	9.77 × 10^−6^	29.51 × 10^−6^	3.09 × 10^−6^	5.62 × 10^−6^	3.39 × 10^−6^	6.46 × 10^−6^
UACC-257	3.98 × 10^−6^	8.51 × 10^−6^	6.61 × 10^−6^	28.84 × 10^−6^	3.39 × 10^−6^	6.61 × 10^−6^	6.61 × 10^−6^	26.91 × 10^−6^
UACC-62	3.98 × 10^−6^	8.32 × 10^−6^	25.12 × 10^−6^	69.18 × 10^−6^	9.77 × 10^−6^	31.62 × 10^−6^	19.95 × 10^−6^	45.71 × 10^−6^

^a^ Molar concentration giving total growth inhibition (TGI). ^b^ Molar concentration leading 50% to net cell death (LC_50_).

## Data Availability

The original contributions presented in this study are included in the article/Supplementary Material. Further inquiries can be directed to the corresponding authors.

## Supplementary Materials

The following supporting information can be downloaded at: https://www.mdpi.com/article/10.3390/molecules30204052/s1, Figures S1 and S2: NOESY spectra of compounds **23** and **24**; Figures S3–S22: ^1^H and ^13^C NMR spectra of compounds **1**–**20**; Figures S23–S42: mass spectra of compounds **1**–**20**; Figures S43–S62: one-dose graphs of compounds **1**–**20**; Figures S63–S68: five-dose graphs of compounds **1**, **5**, **10**, **11**, **17** and **20**; Figures S69–S71: waterfall graphs GI_50_, TGI and LC_50_ of compound **1**; Figures S72–S74: waterfall graphs GI_50_, TGI and LC_50_ of compound **5**; Figures S75–S77: waterfall graphs GI_50_, TGI and LC_50_ of compound **10**; Figures S78–S80: waterfall graphs GI_50_, TGI and LC_50_ of compound **11**; Figures S81–S83: waterfall graphs GI_50_, TGI and LC_50_ of compound **17**; Figures S84–S86: waterfall graphs GI_50_, TGI and LC_50_ of compound **20**; Figure S87: preliminary ZIKV/Huh-7 antiviral screening results for compounds **1**–**20**; Figure S88: preliminary IAV/MDCK antiviral screening results for compounds **1**–**20**; Figure S89: preliminary SARS-CoV-2/Vero TMPRSS antiviral screening results for compounds **1**–**20**.
