# Supplementary material for: Arylpiperazinyl-Benzocycloheptapyrrole-Carboxamides Endowed with Dual Anticancer and Antiviral Activities
Source: Molecules. 2025 Oct 11;30(20):4052. doi: 10.3390/molecules30204052 (PMC12566046; doi:10.3390/molecules30204052)
Supplement: Supplementary file 1 [file molecules-30-04052-s001.zip › molecules-3886772-supplementary.pdf]

Supplementary Material

Arylpiperazinyl-Benzocycloheptapyrrole-Carboxamides Endowed with Dual Anticancer and Antiviral Activities

Gabriele Murineddu [1,*], Carlo Di Marzo [2,†], Paola Corona [1,†], Silvia Coinu [1], Erika Plicanti [2], Battistina Asproni [1], Sandra Piras [1], Giulia Freer [2] and Antonio Carta [1,*]

[1] Department of Medicine, Surgery and Pharmacy, University of Sassari, 07100 Sassari, Italy; pcorona@uniss.it (P.C.); s.coinu1@phd.uniss.it (S.C.); asproni@uniss.it (B.A.); piras@uniss.it (S.P.)
[2] Centro Retrovirus, Department of Translational Research, University of Pisa, 56127 Pisa, Italy; dimarzo.carlo@virgilio.it (C.D.M.); e.plicanti@studenti.unipi.it (E.P.); giulia.freer@med.unipi.it (G.F.)
* Correspondence: muri@uniss.it (G.M.); acarta@uniss.it (A.C.)
† These Authors contributed equally to this work.

Contents

Figures S1 and **S2**: NOESY spectra of compounds **23** and **24**

Figures S3–S22: ^{1}H and ^{13}C NMR spectra of compounds **1 – 20**

Figures S23–S42: mass spectra of compounds **1 – 20**

Figures S43–S62: one-dose graphs of compounds **1 – 20**

Figures S63–S68: five-dose graphs of compounds **1**, **5**, **10**, **11**, **17** and **20**

Figures S69–S71: waterfall graphs GI_{50}, TGI and LC_{50} of compound **1**

Figures S72–S74: waterfall graphs GI_{50}, TGI and LC_{50} of compound **5**

Figures S75–S77: waterfall graphs GI_{50}, TGI and LC_{50} of compound **10**

Figures S78–S80: waterfall graphs GI_{50}, TGI and LC_{50} of compound **11**

Figures S81–S83: waterfall graphs GI_{50}, TGI and LC_{50} of compound **17**

Figures S84–S86: waterfall graphs GI_{50}, TGI and LC_{50} of compound **20**

Figure S87: preliminary ZIKV/Huh-7 antiviral screening results for compounds **1–20**

Figure S88: preliminary IAV/MDCK antiviral screening results for compounds **1–20**

Figure S89: preliminary SARS-CoV-2/Vero TMPRSS antiviral screening results for compounds **1–20**

Figure S1: NOESY spectrum of compound **23**

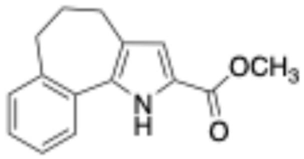

23

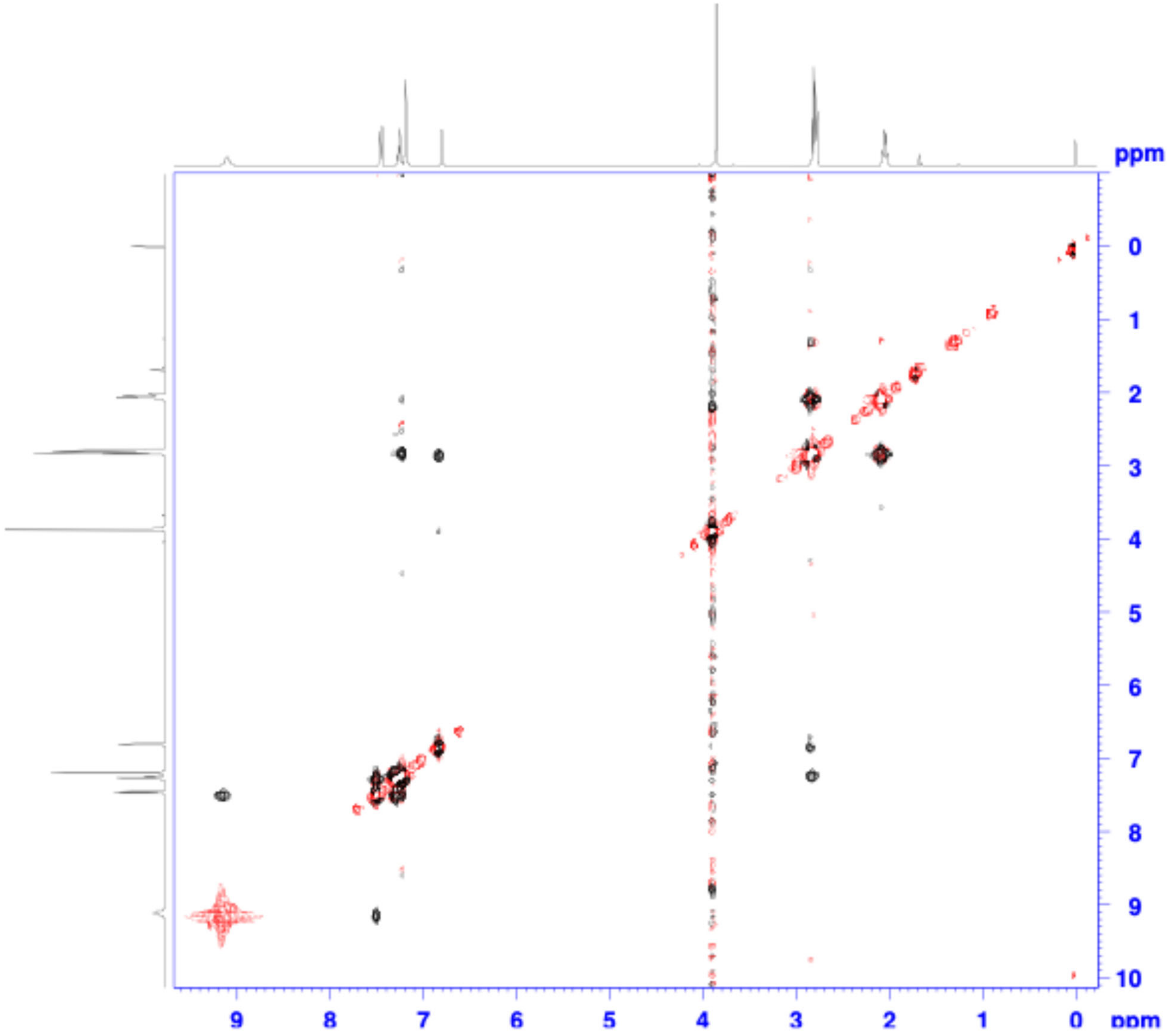

Figure S2: NOESY spectrum of compound **24**

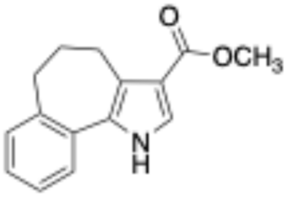

24

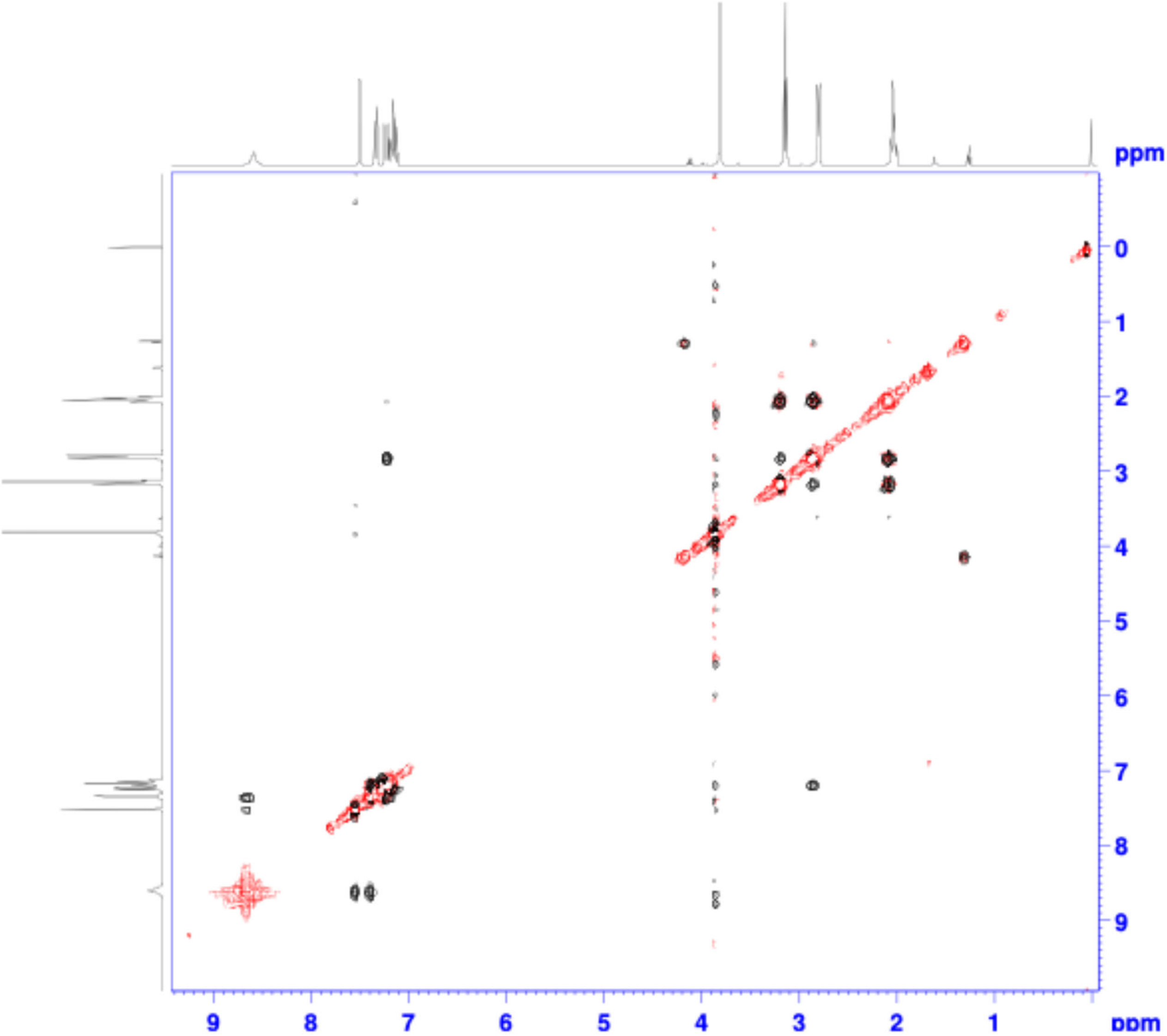

Figure S3: ^{1}H and ^{13}C NMR spectra of compound **1**

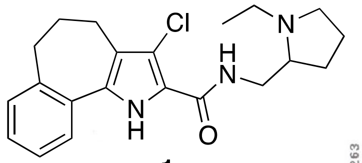

1

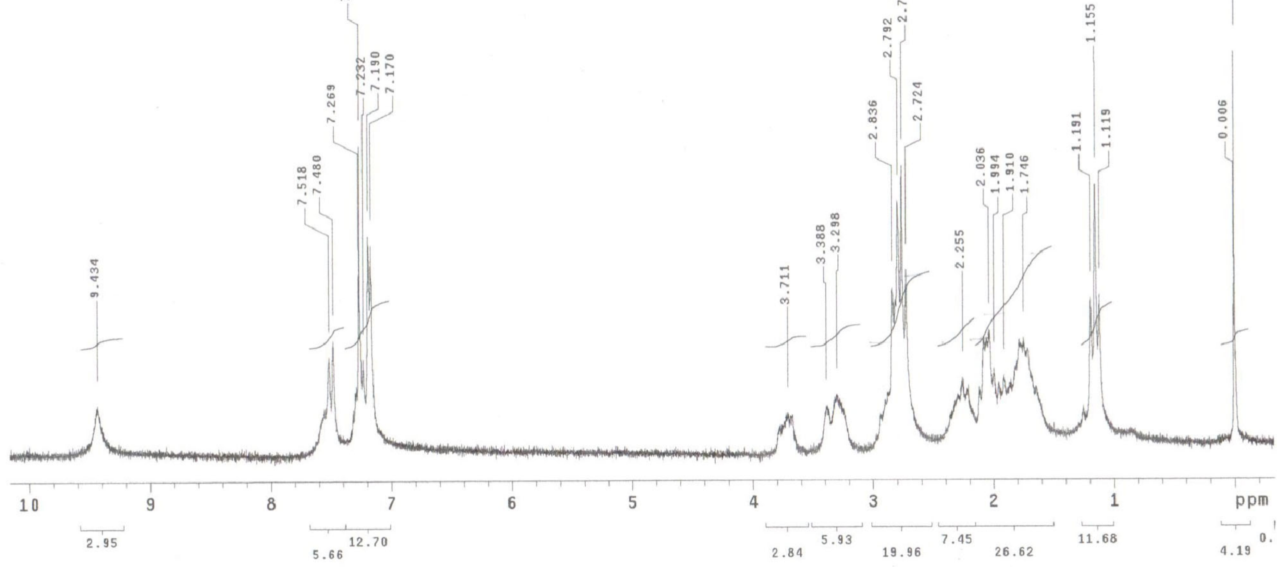

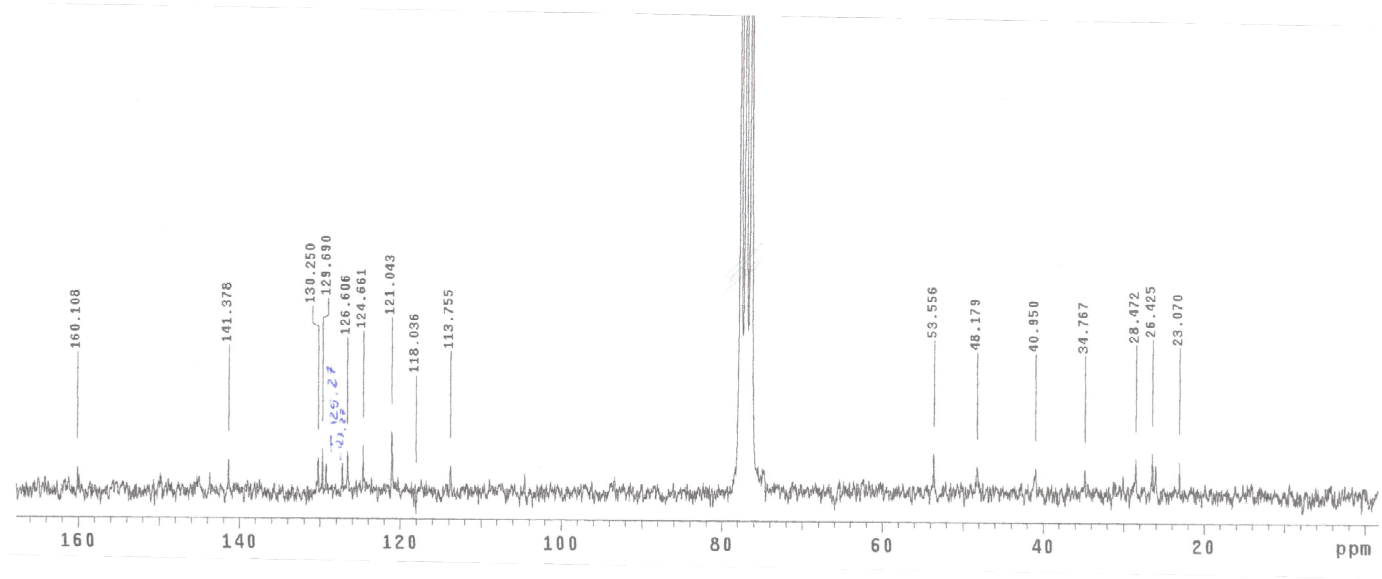

Figure S4: ^{1}H and ^{13}C NMR spectra of compound **2**

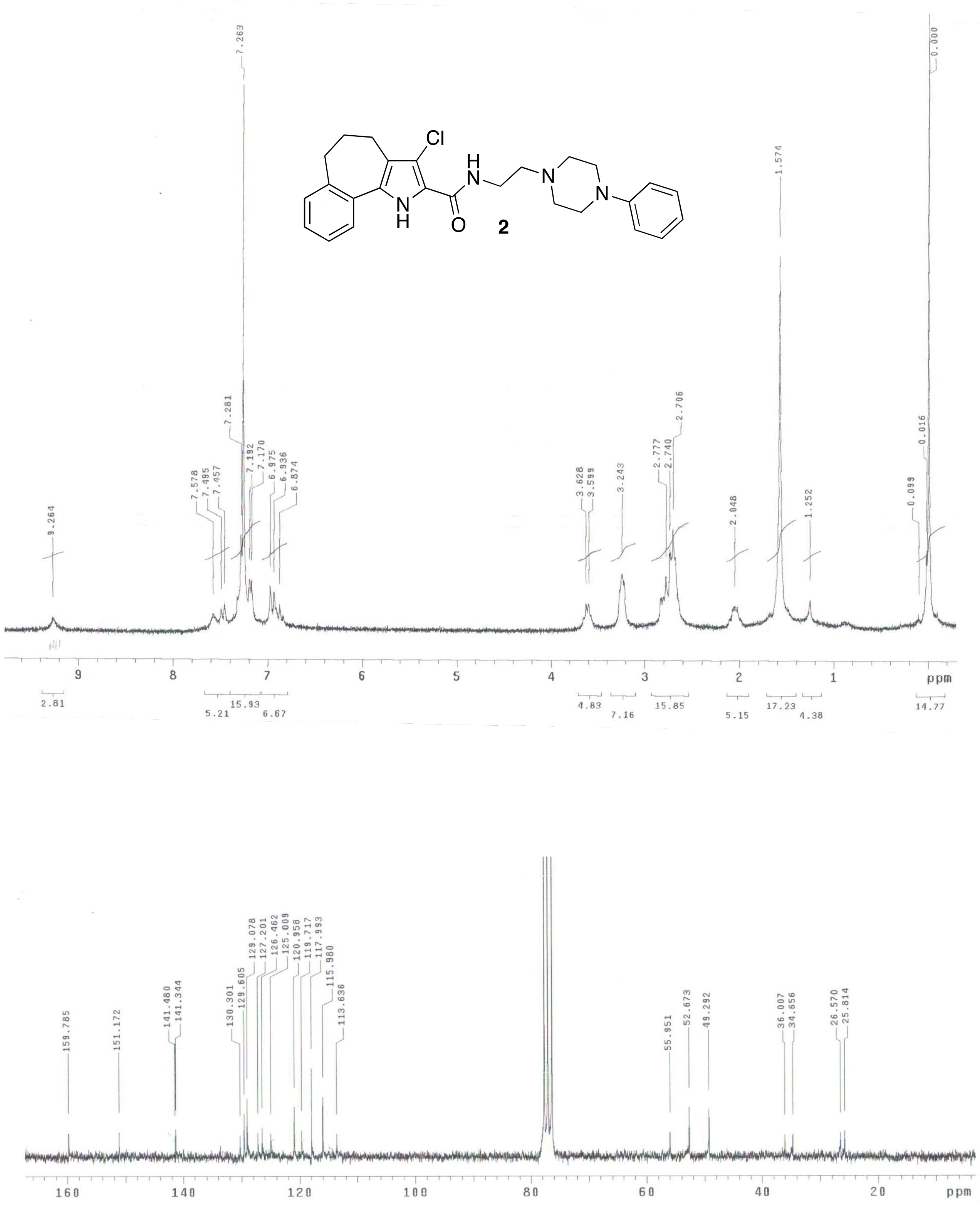

Figure S5: ^{1}H and ^{13}C NMR spectra of compound **3**

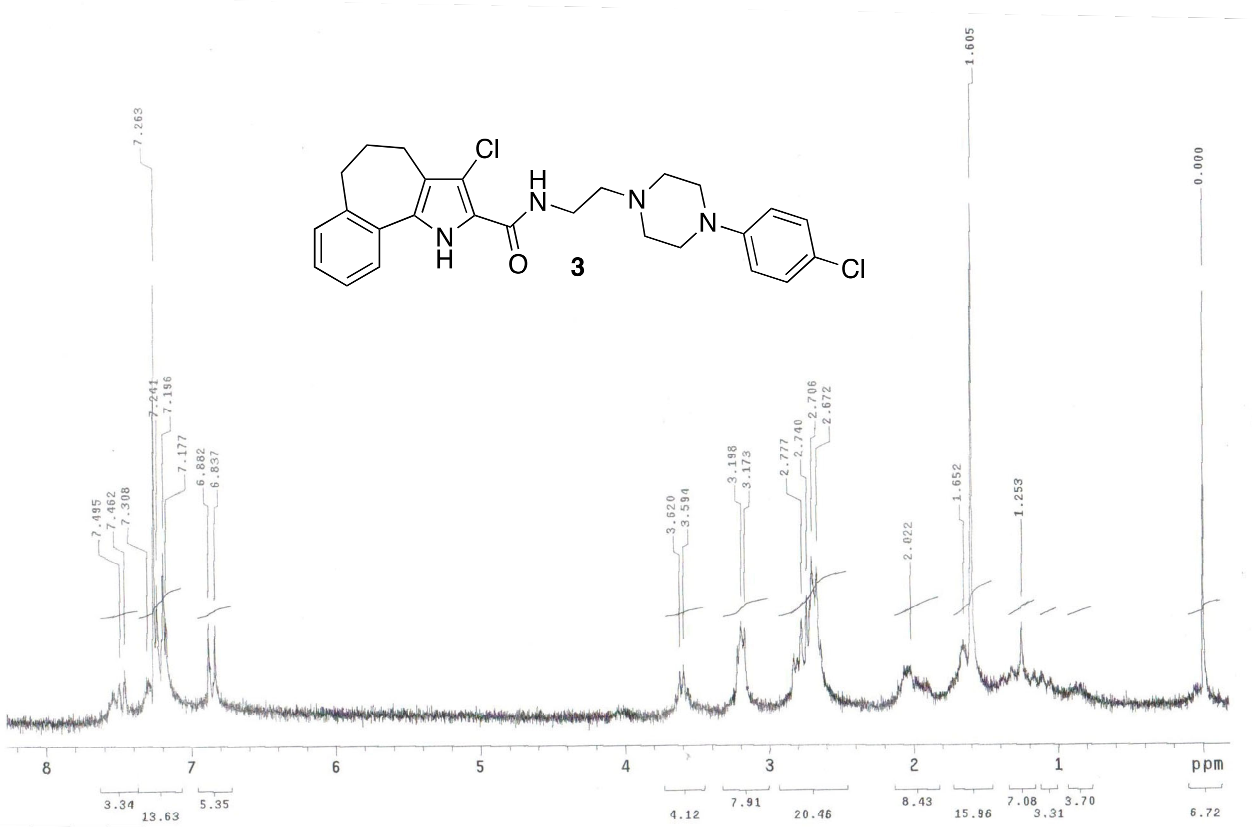

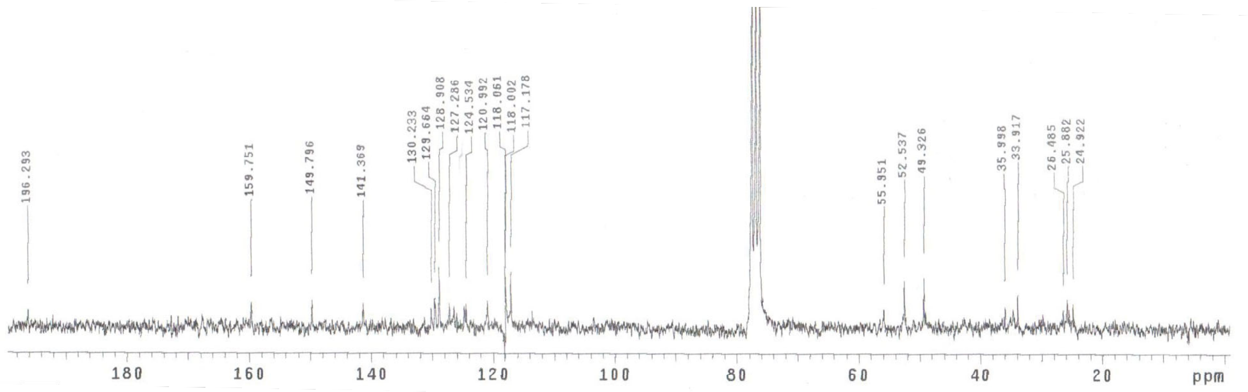

Figure S6: ^{1}H and ^{13}C NMR spectra of compound **4**

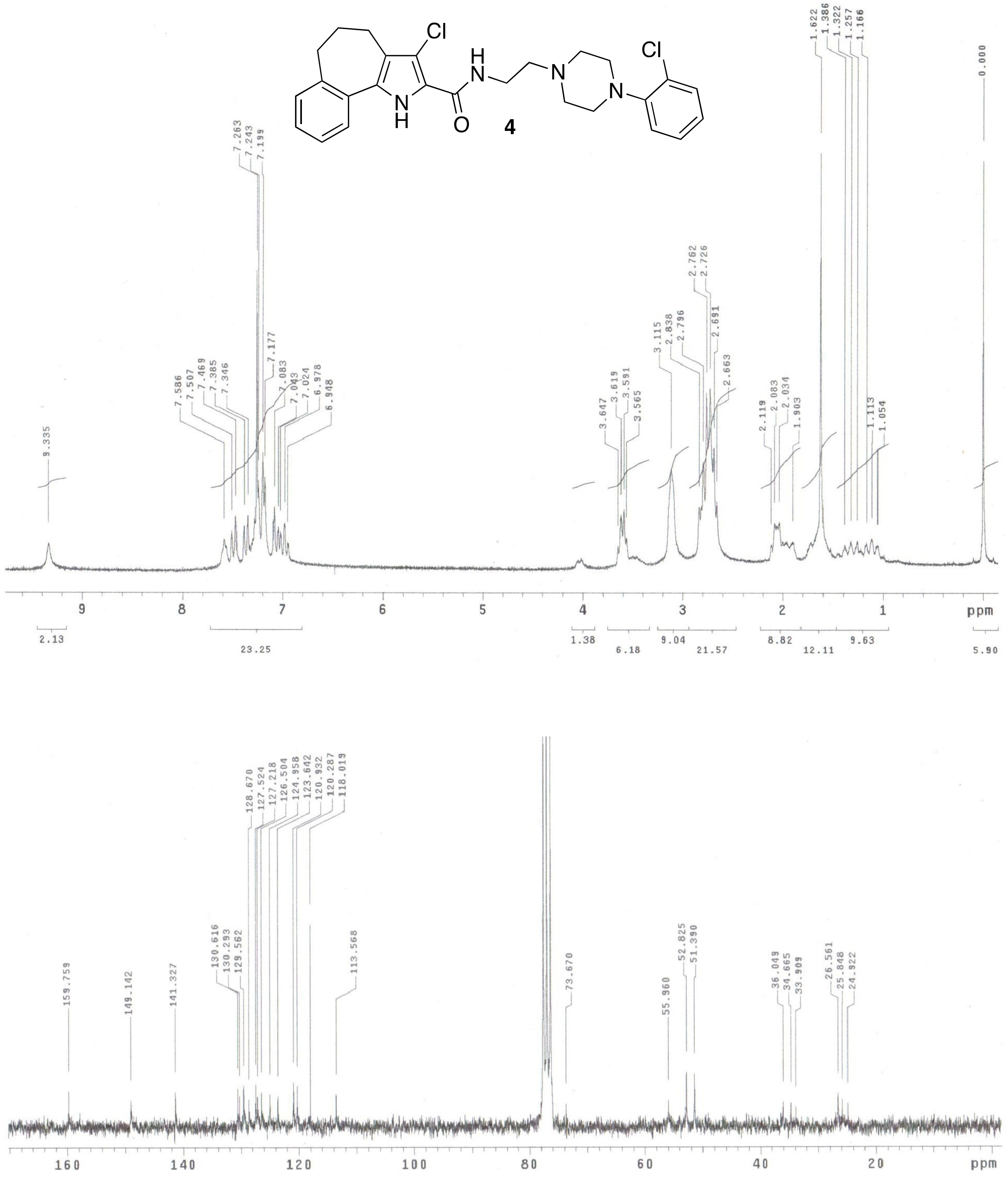

Figure S7: ^{1}H and ^{13}C NMR spectra of compound **5**

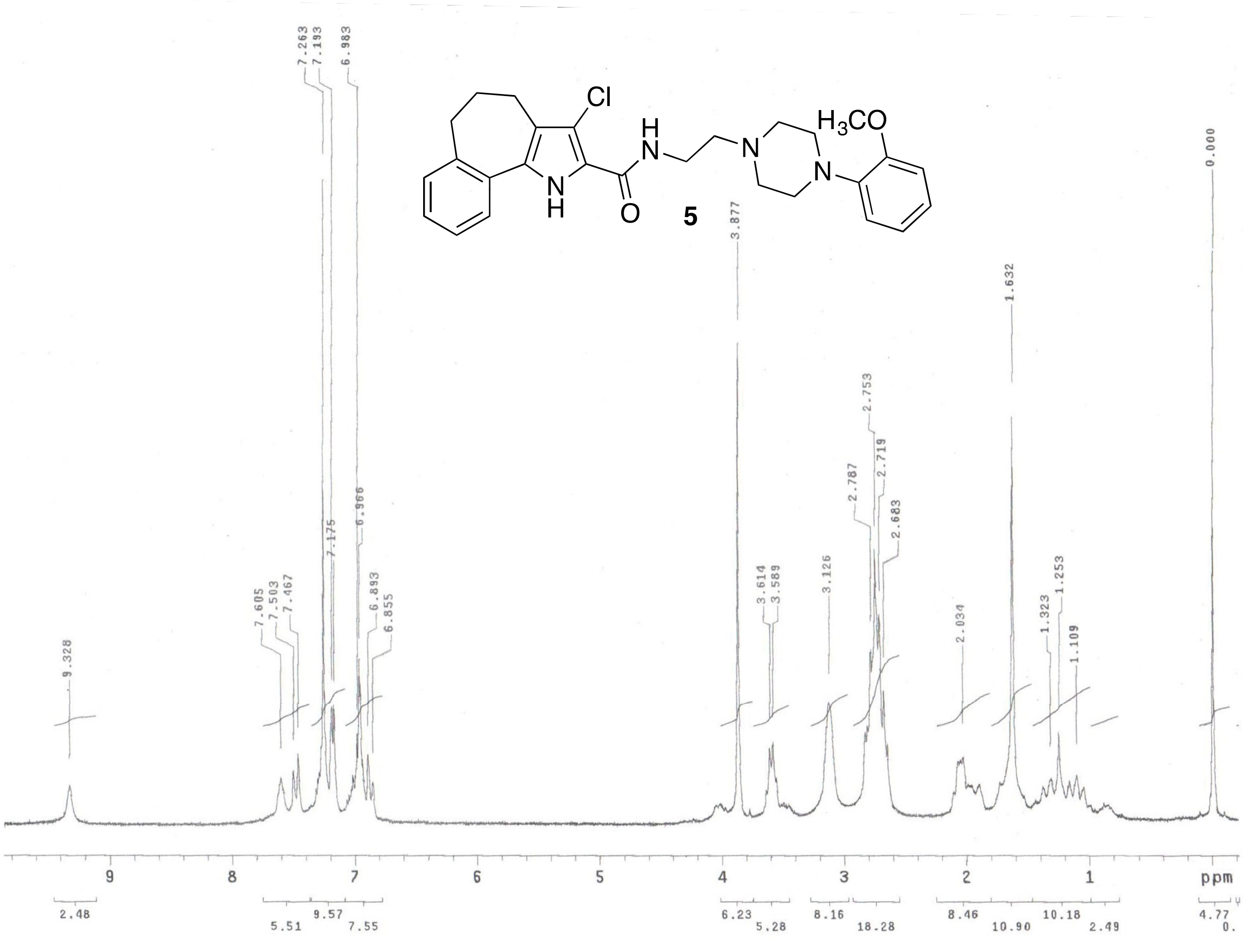

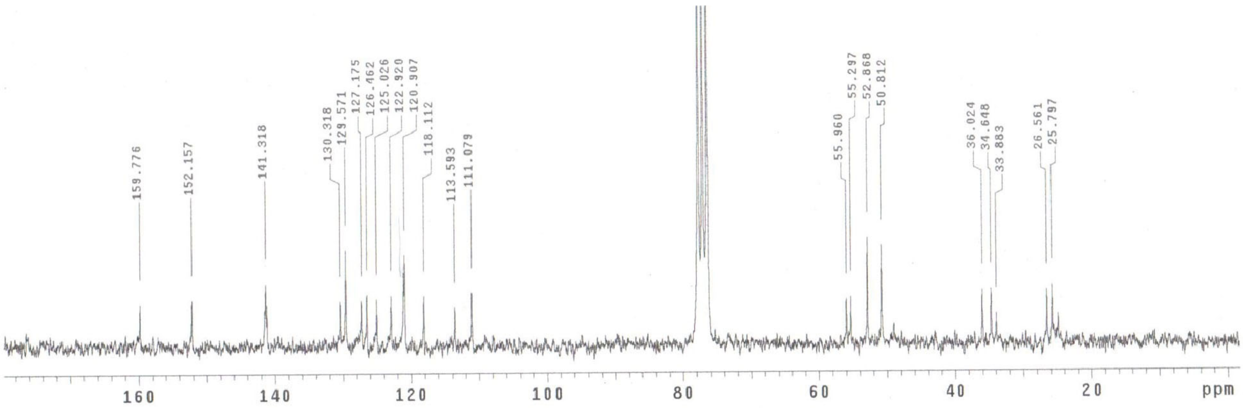

Figure S8: ^{1}H and ^{13}C NMR spectra of compound **6**

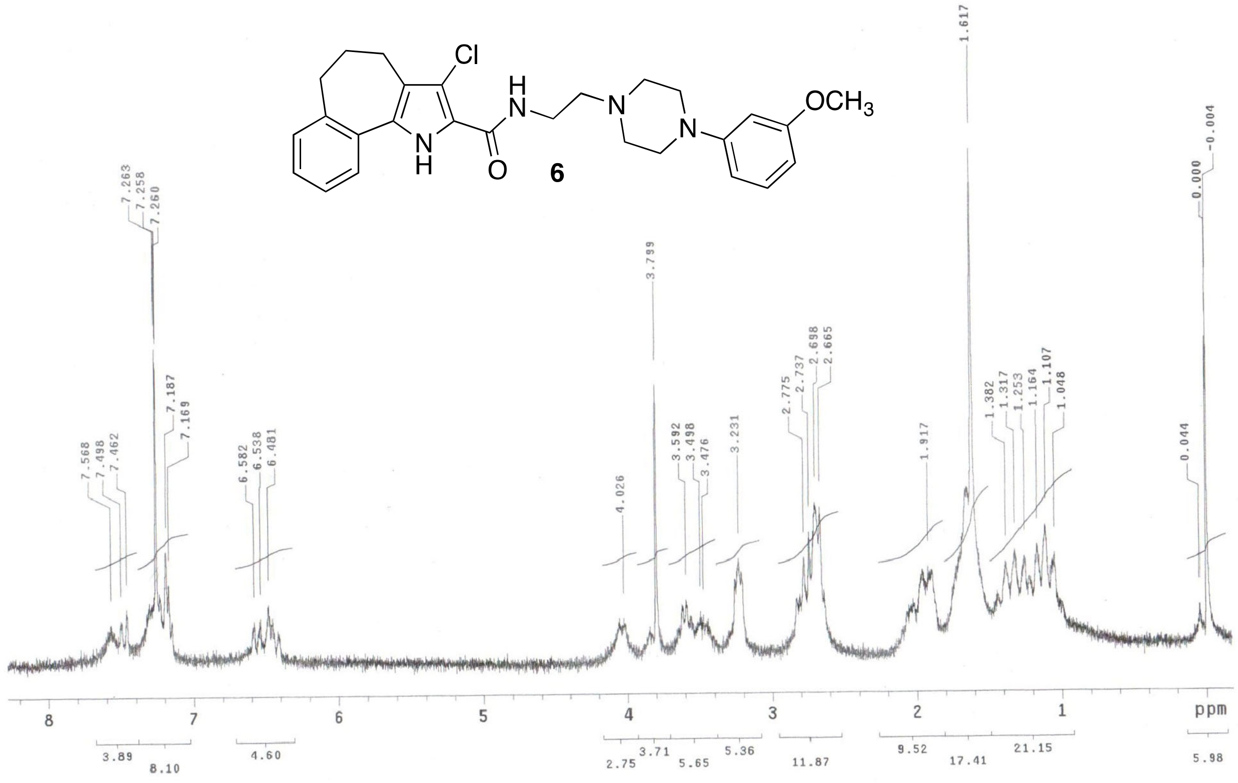

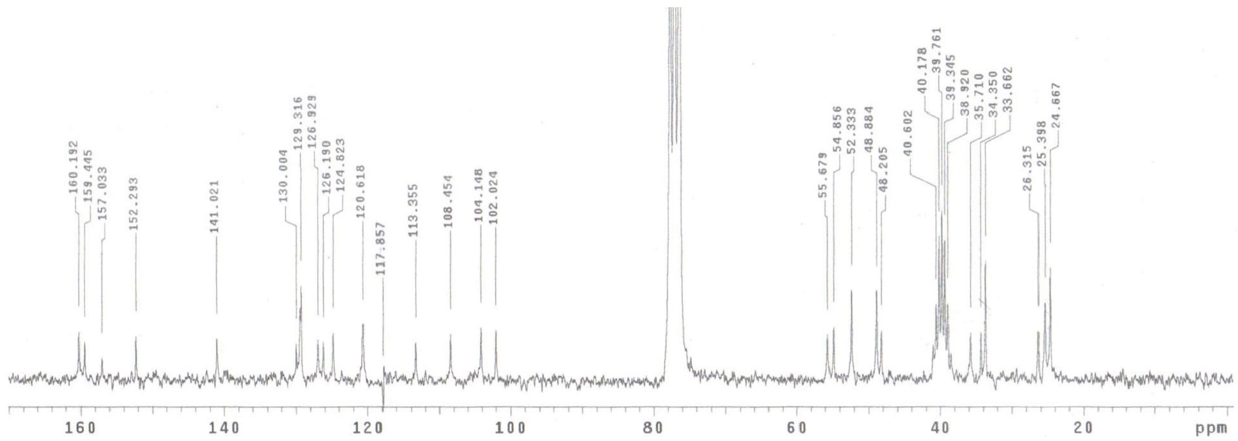

Figure S9: ^{1}H NMR and ^{13}C APT spectra of compound **7**

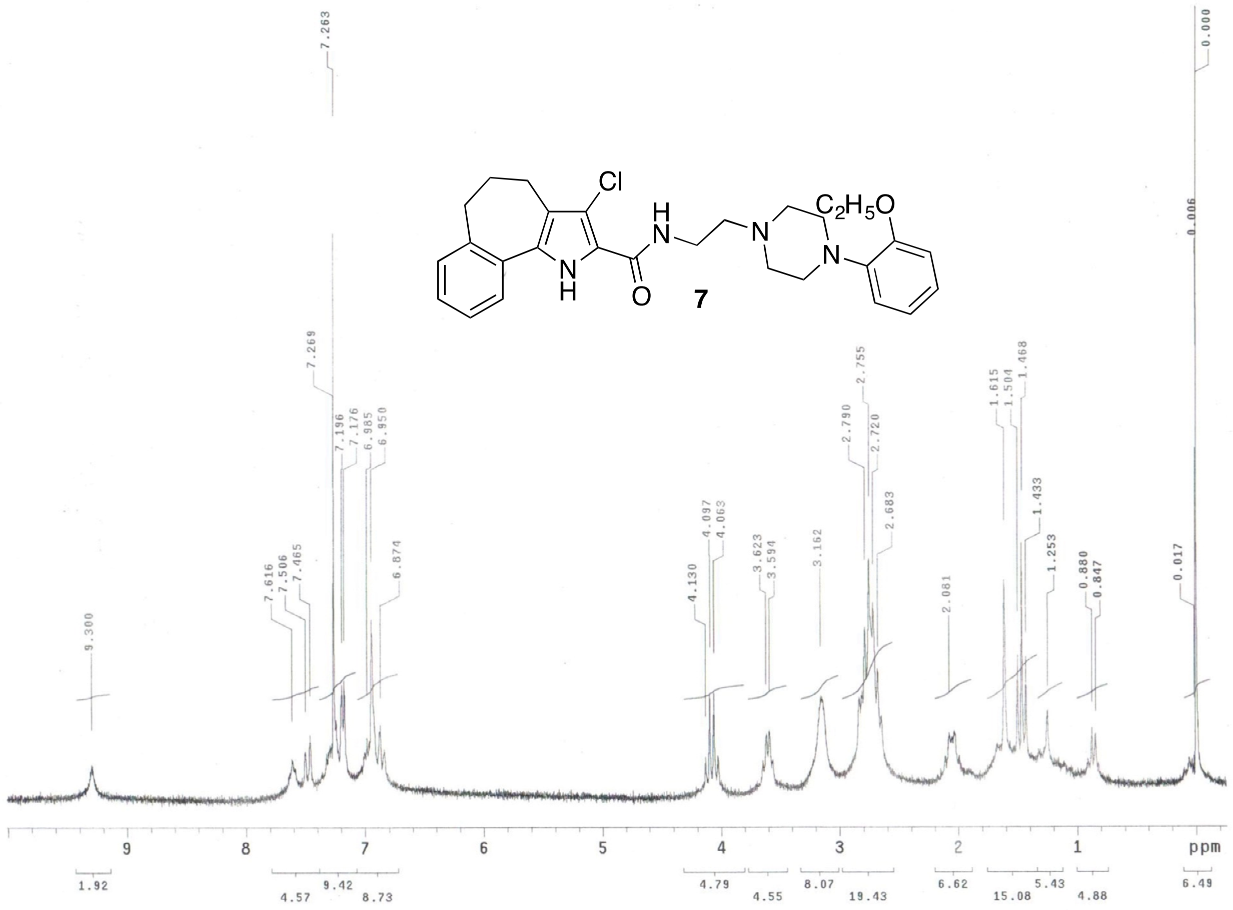

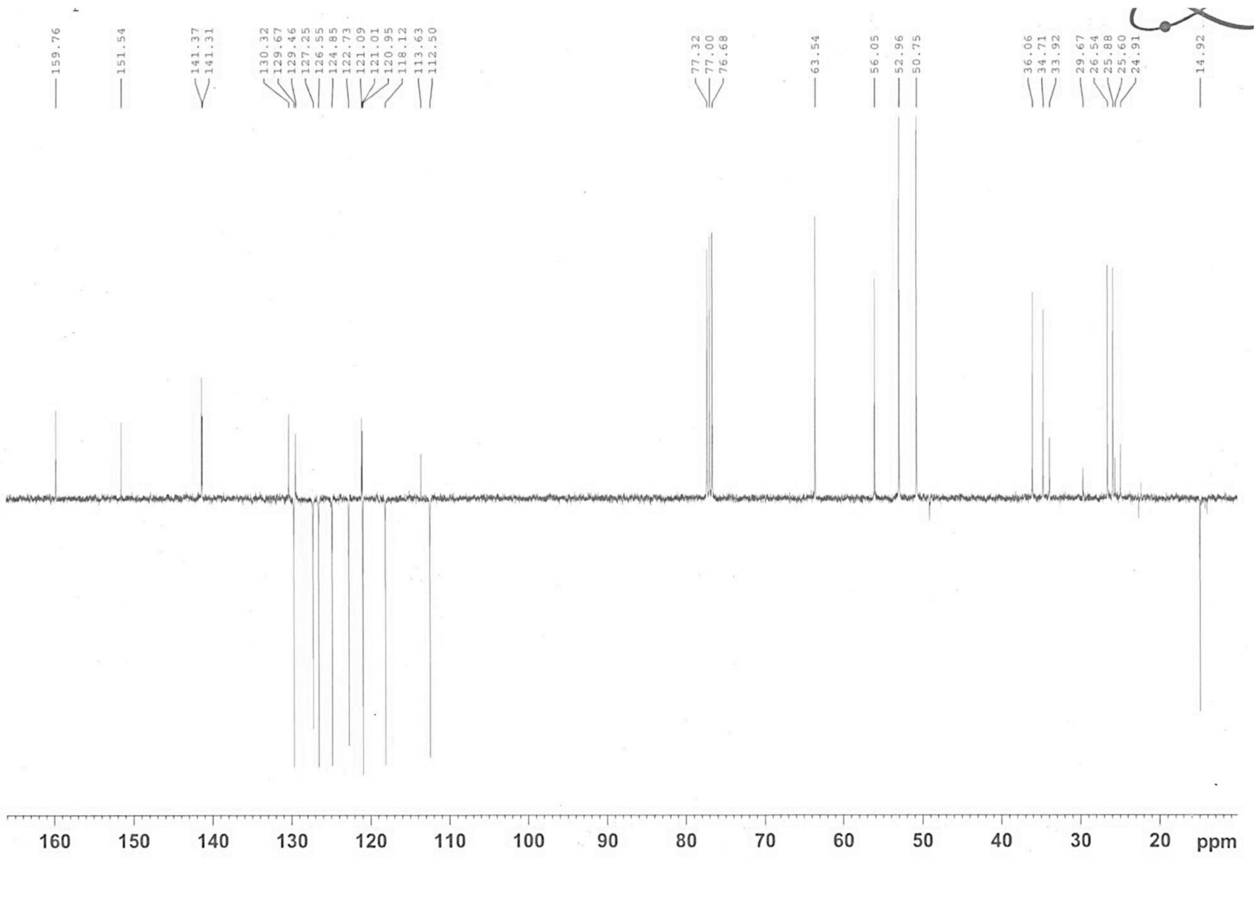

Figure S10: ^{1}H and ^{13}C NMR spectra of compound **8**

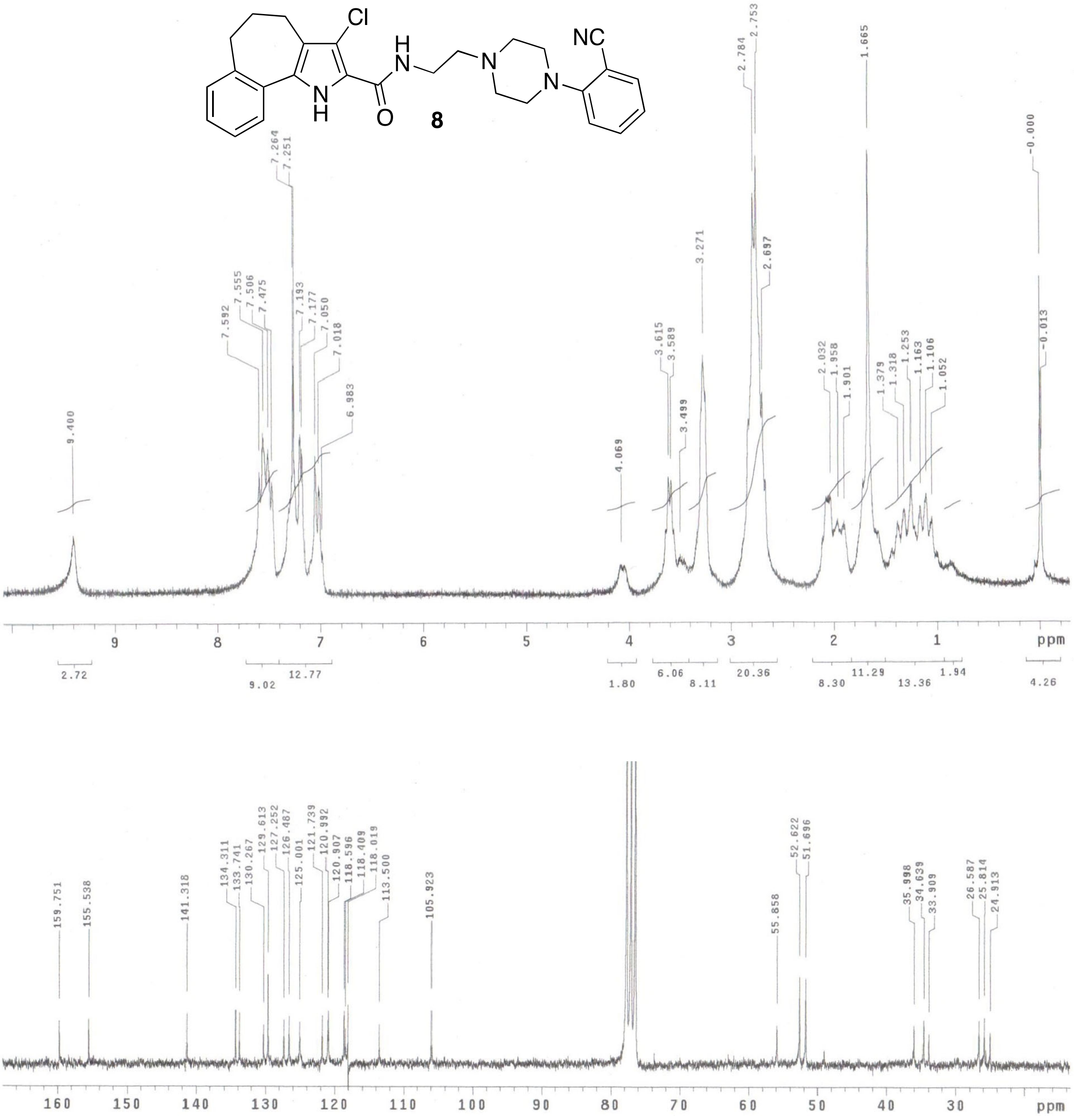

Figure S11: ^{1}H and ^{13}C NMR spectra of compound **9**

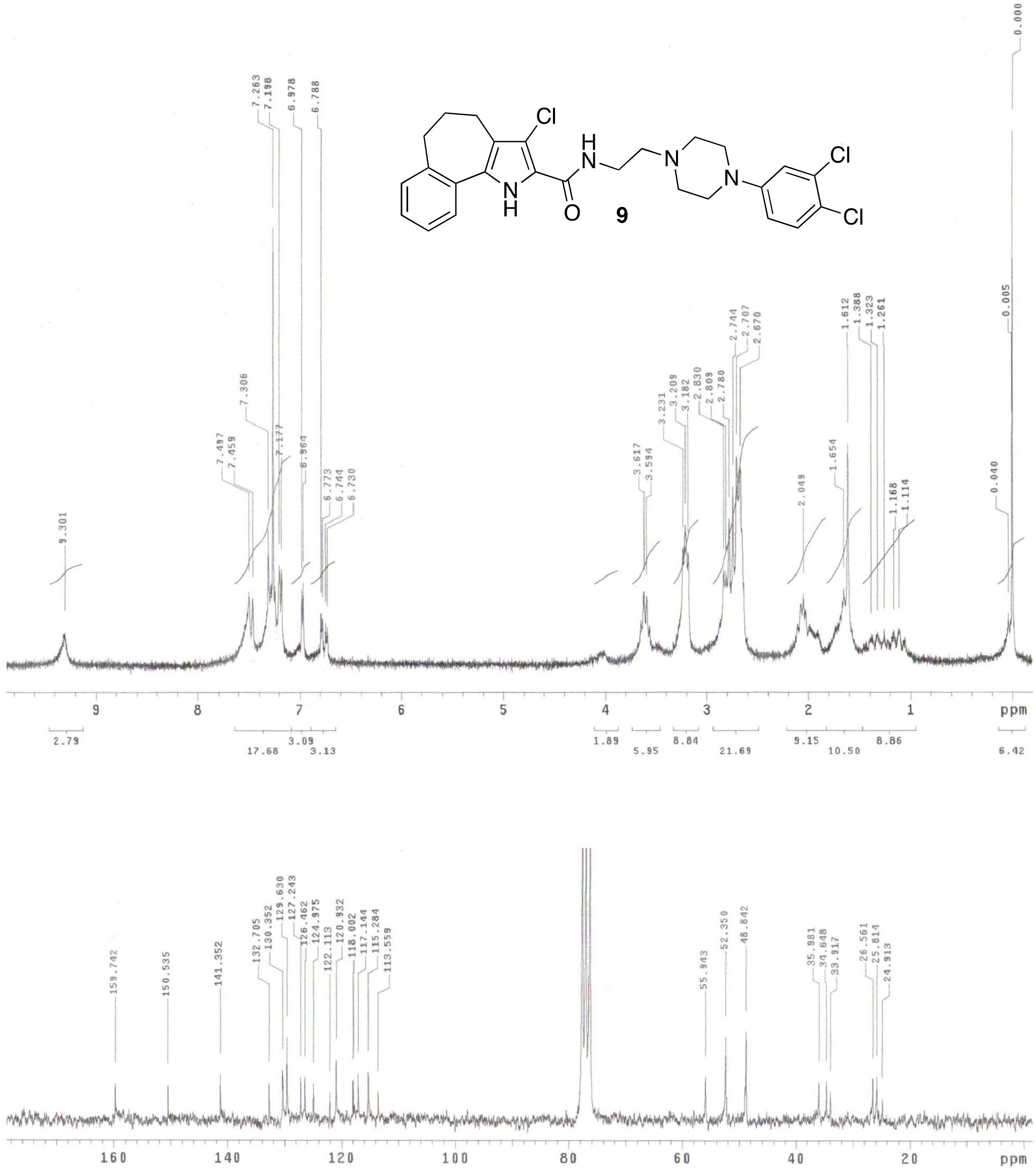

Figure S12: ^{1}H spectrum of compound **10**

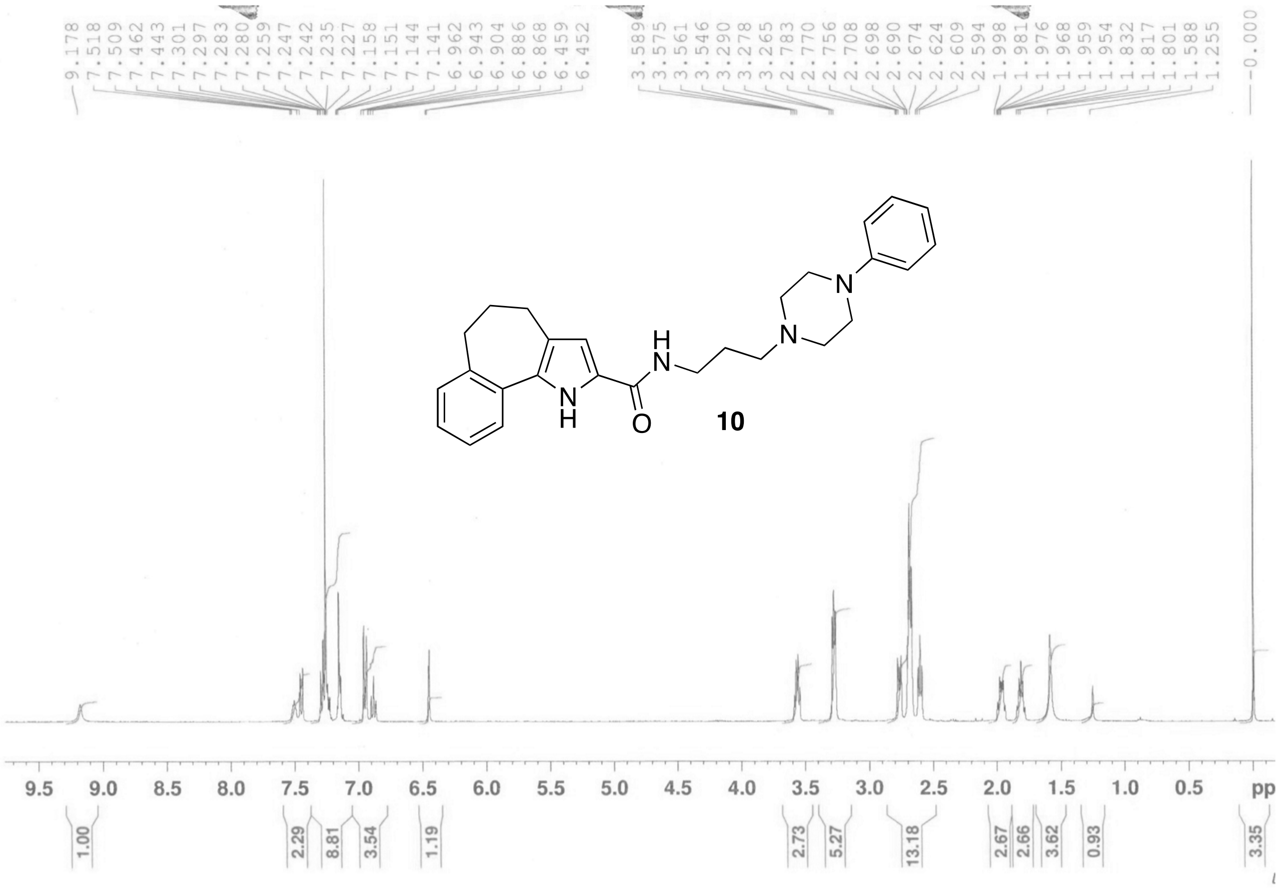

Figure S13: ^{1}H NMR and ^{13}C APT spectra of compound **11**

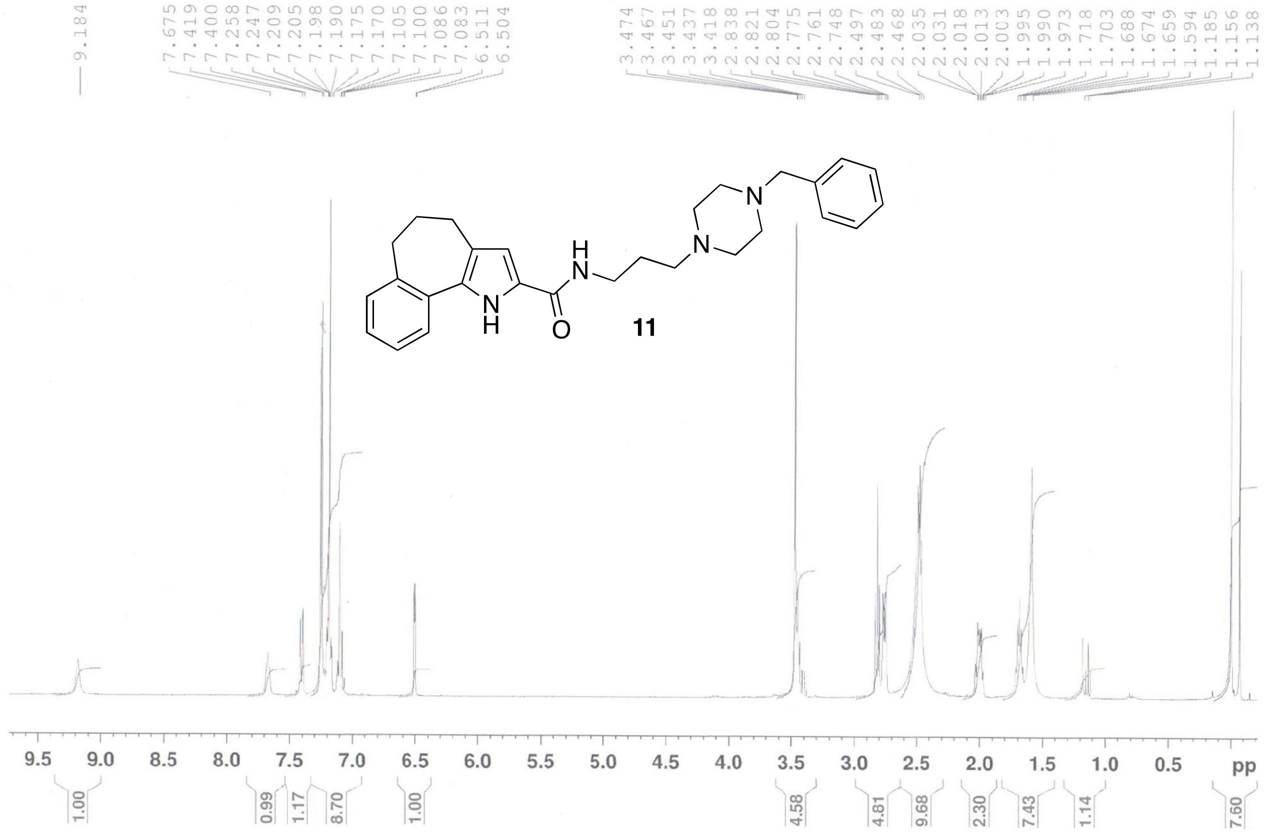

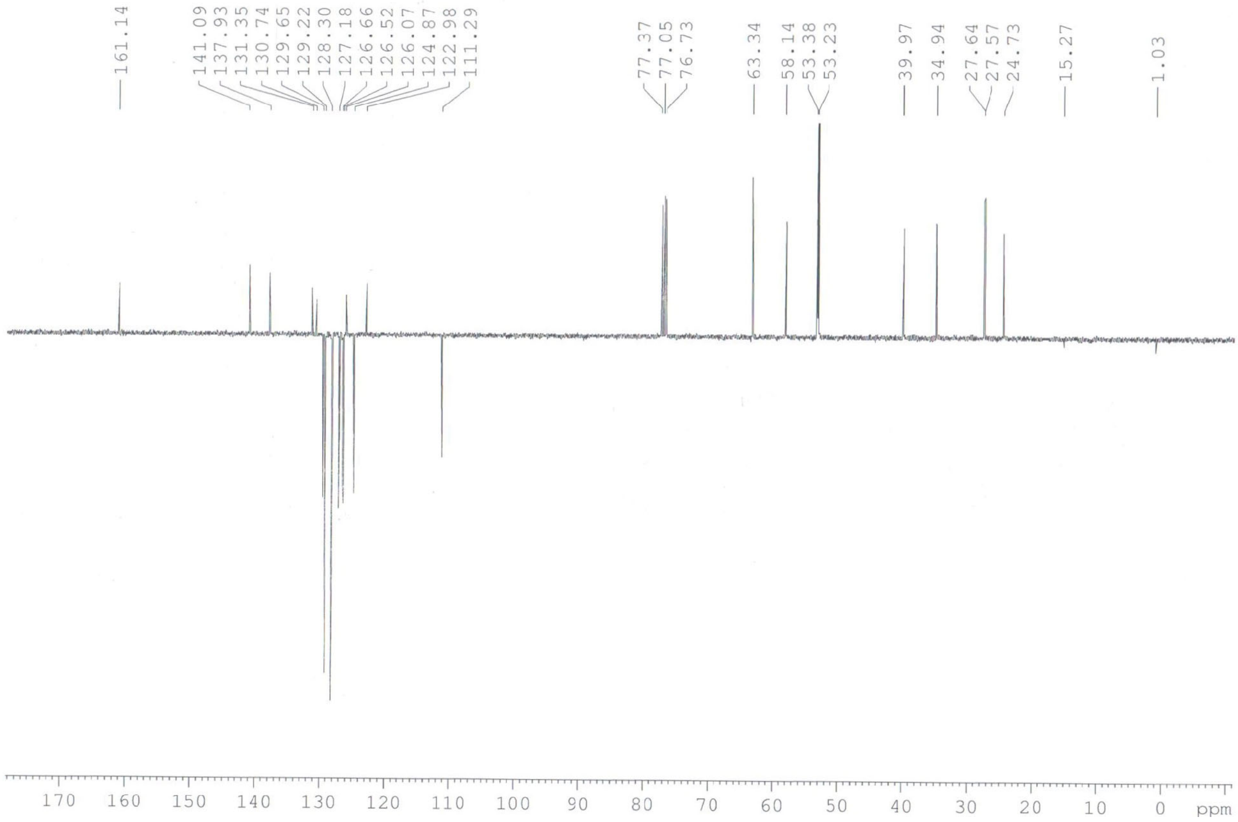

Figure S14: ^{1}H and ^{13}C NMR spectra of compound **12**

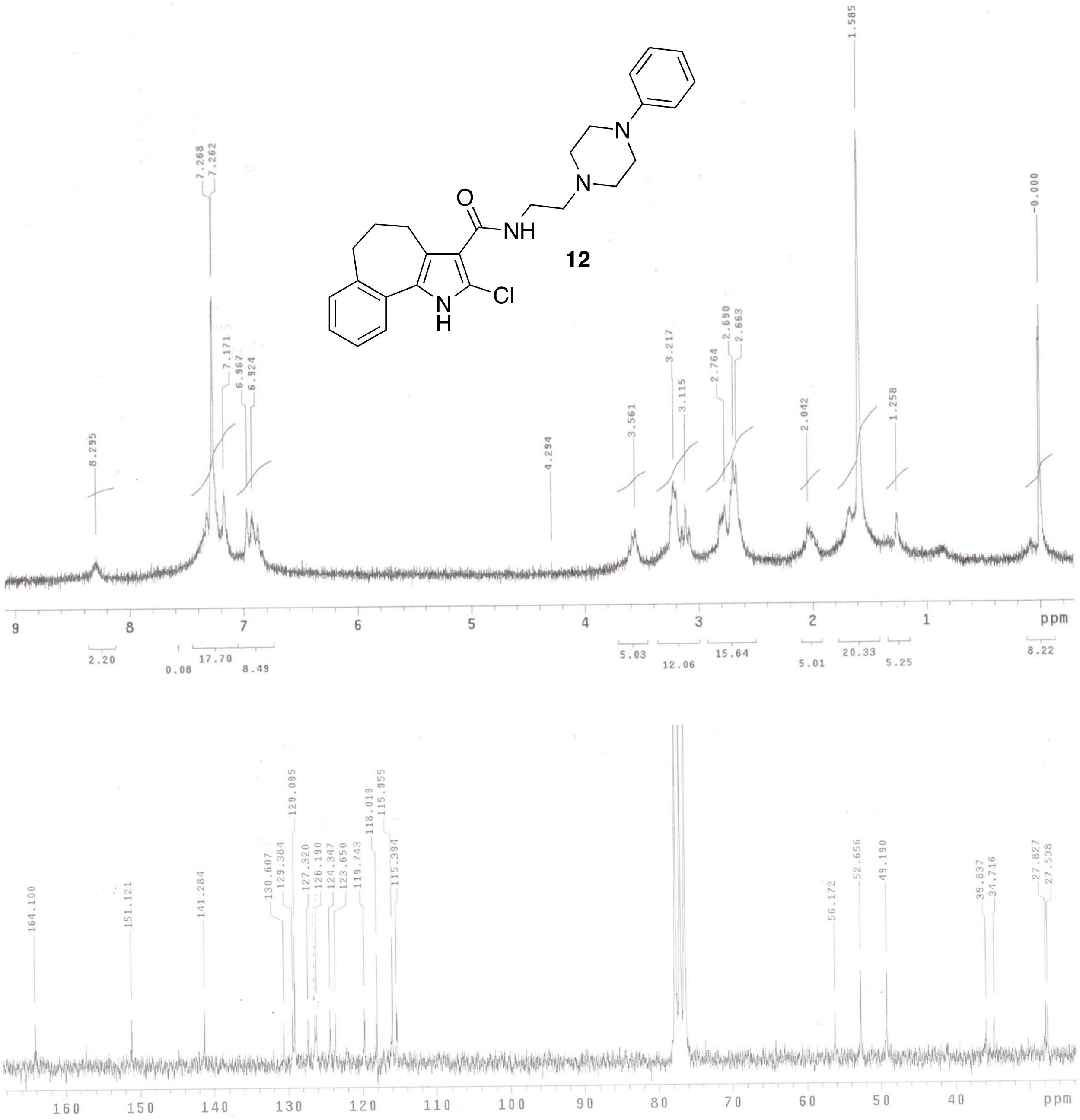

Figure S15: ^{1}H NMR and ^{13}C APT spectra of compound **13**

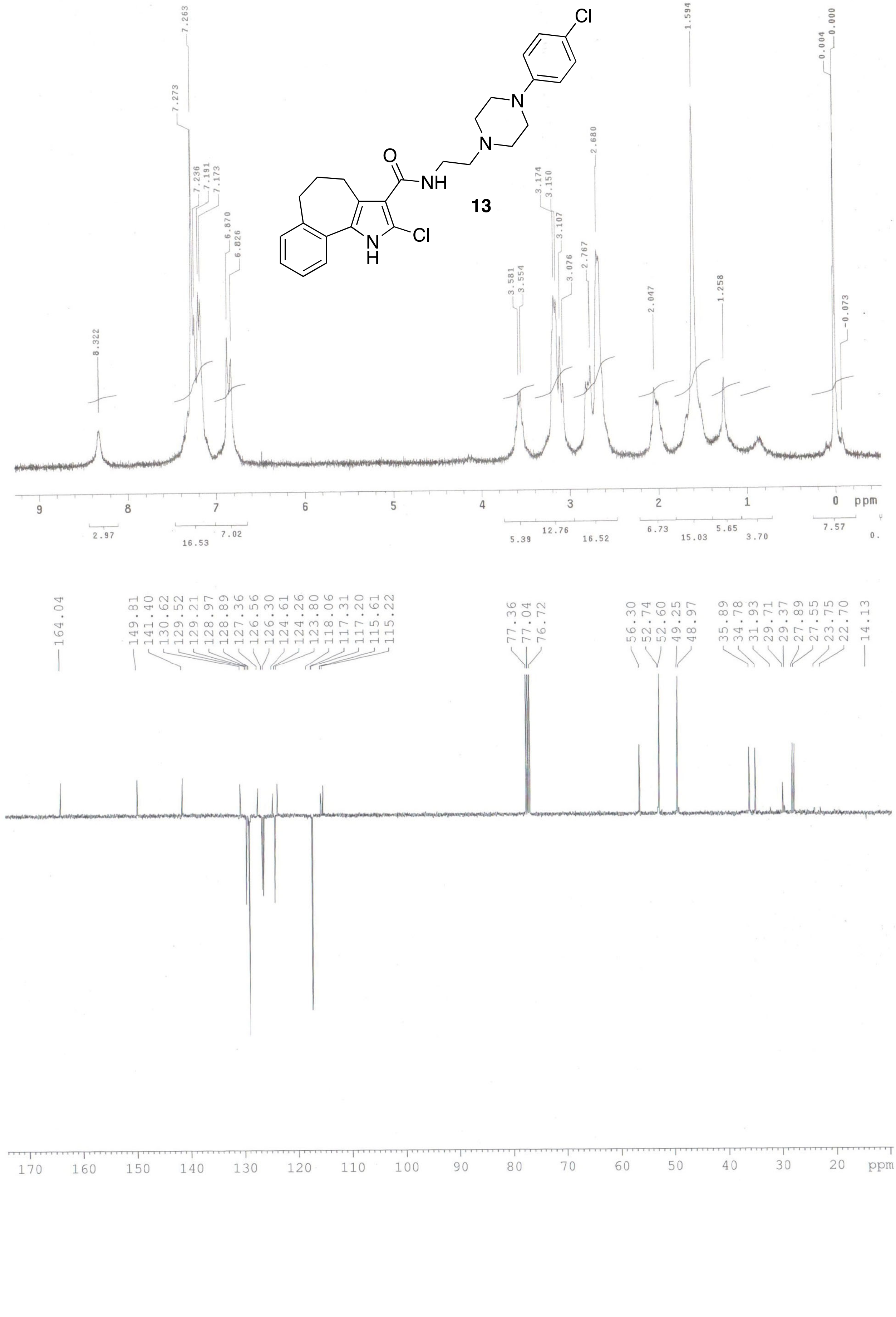

Figure S16: ^{1}H NMR and ^{13}C APT spectra of compound **14**

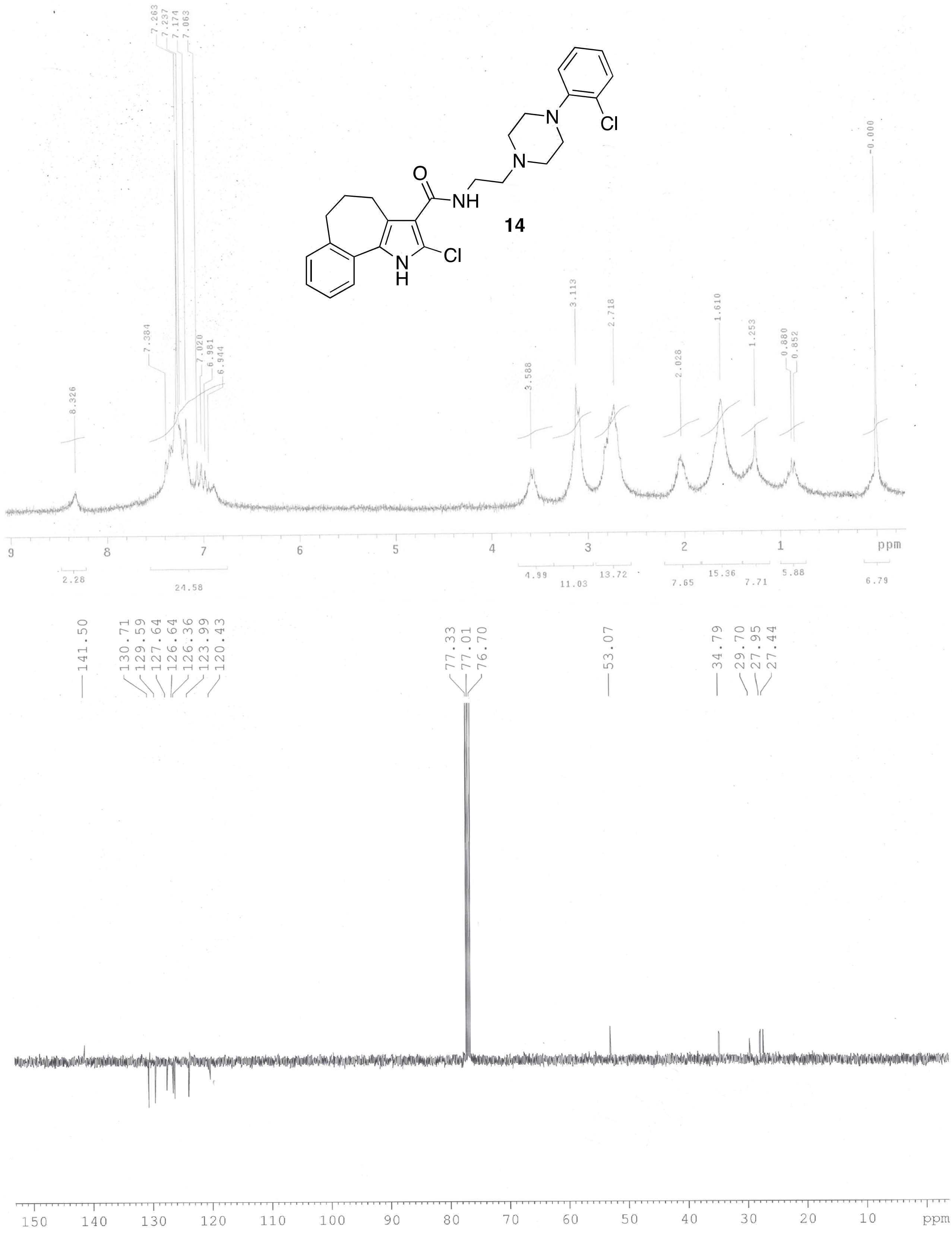

Figure S17: ^{1}H and ^{13}C NMR spectra of compound **15**

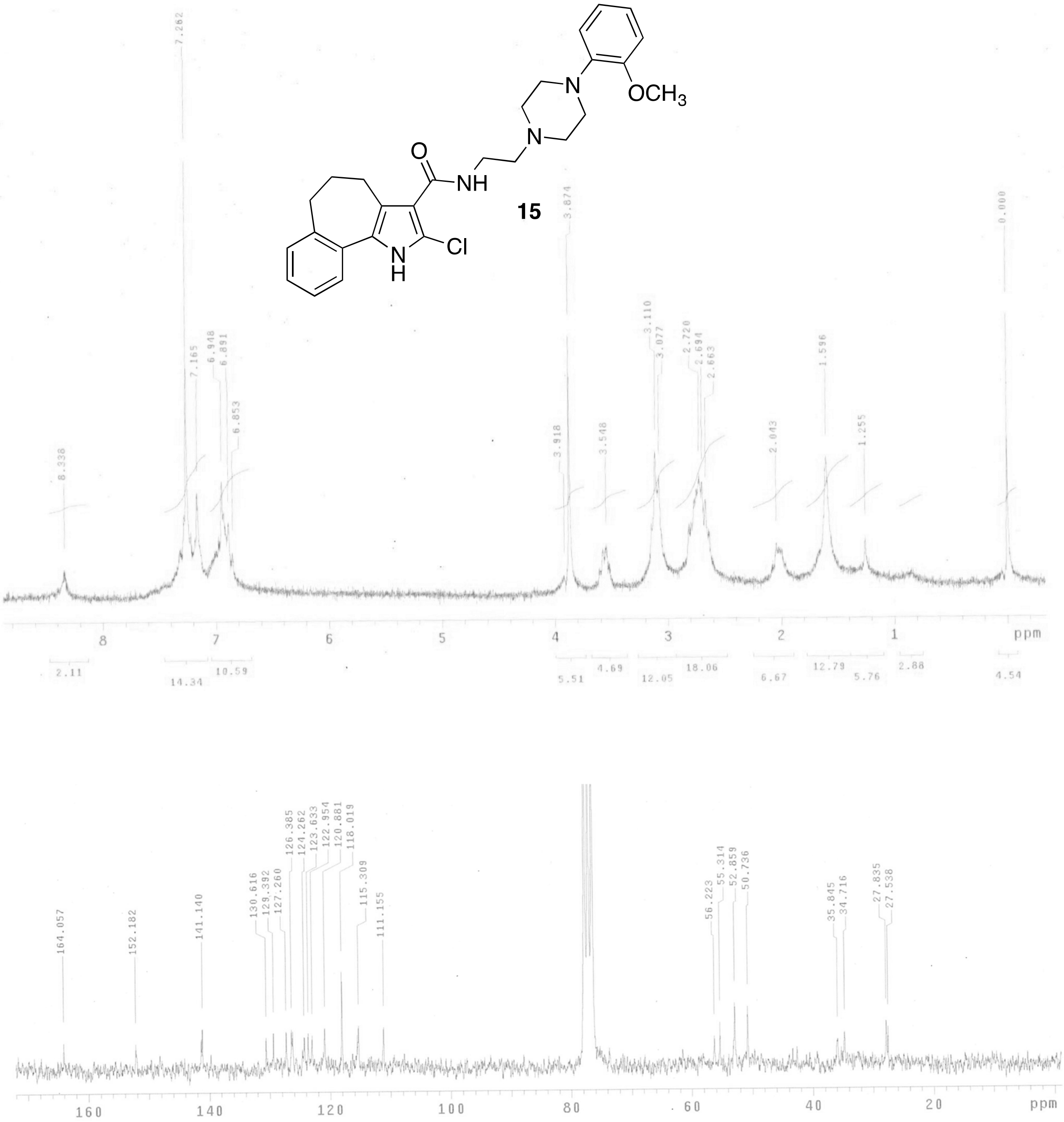

Figure S18: ^{1}H and ^{13}C NMR spectra of compound **16**

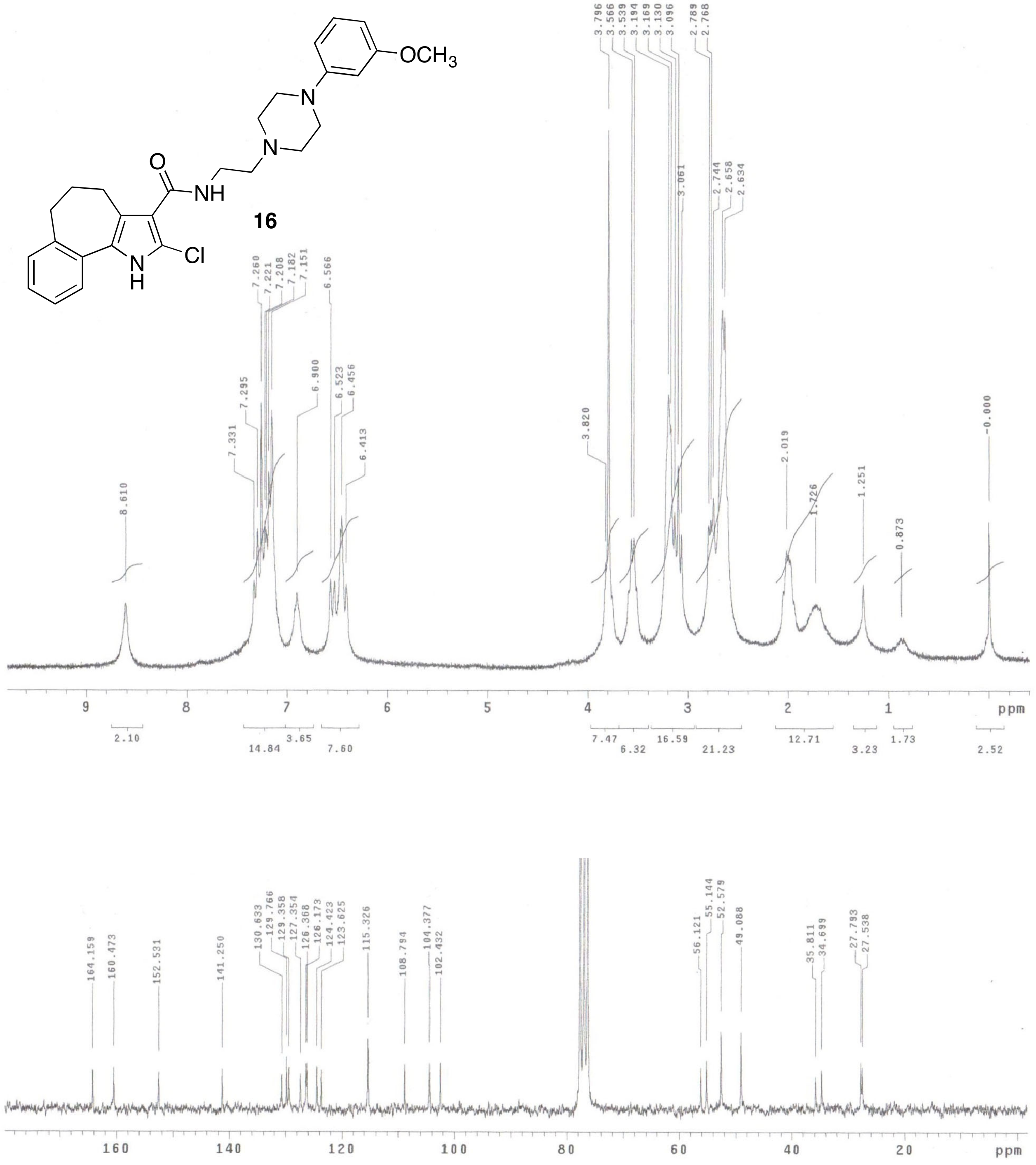

Figure S19: ^{1}H and ^{13}C NMR spectra of compound **17**

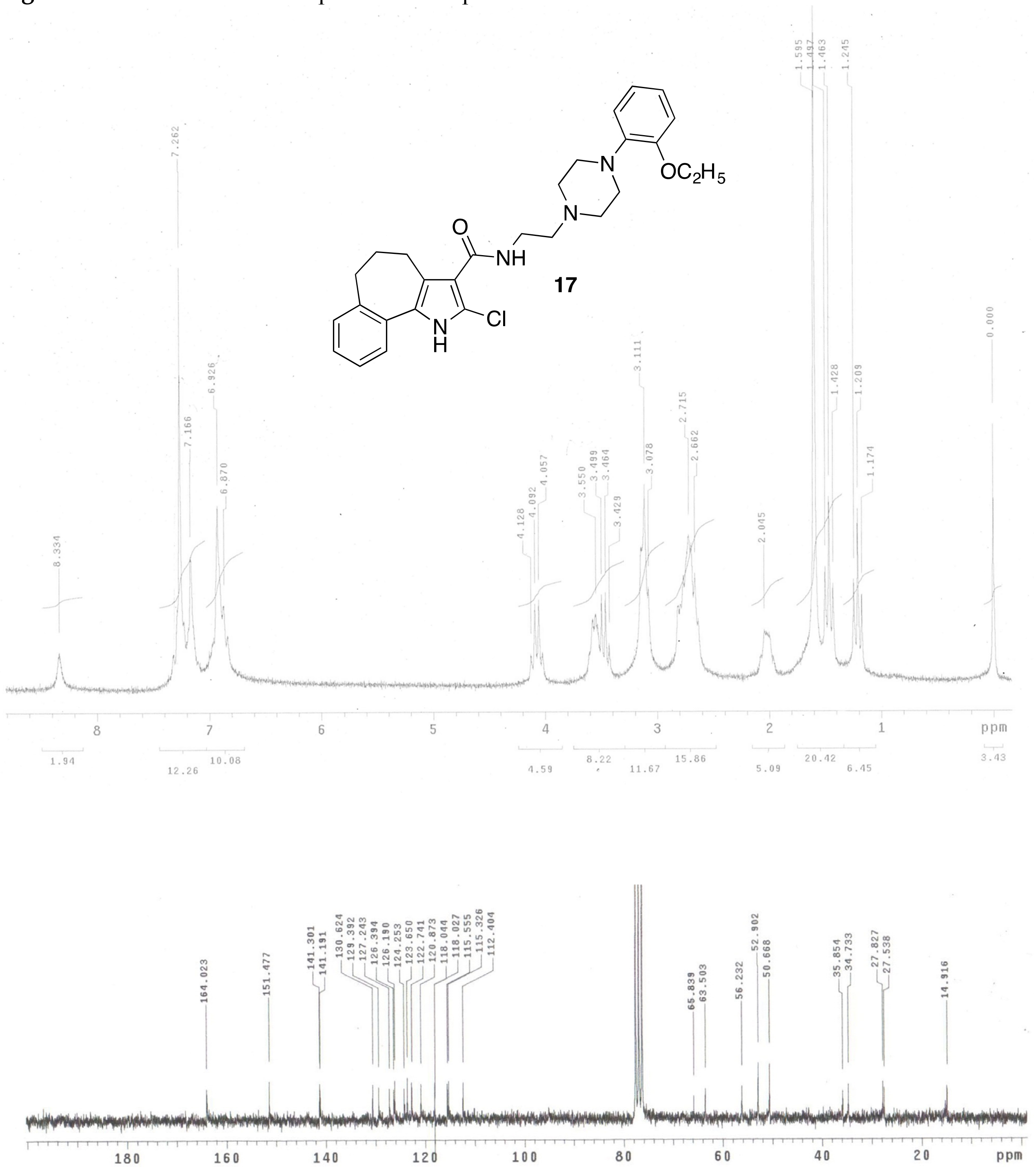

Figure S20: ^{1}H and ^{13}C NMR spectra of compound **18**

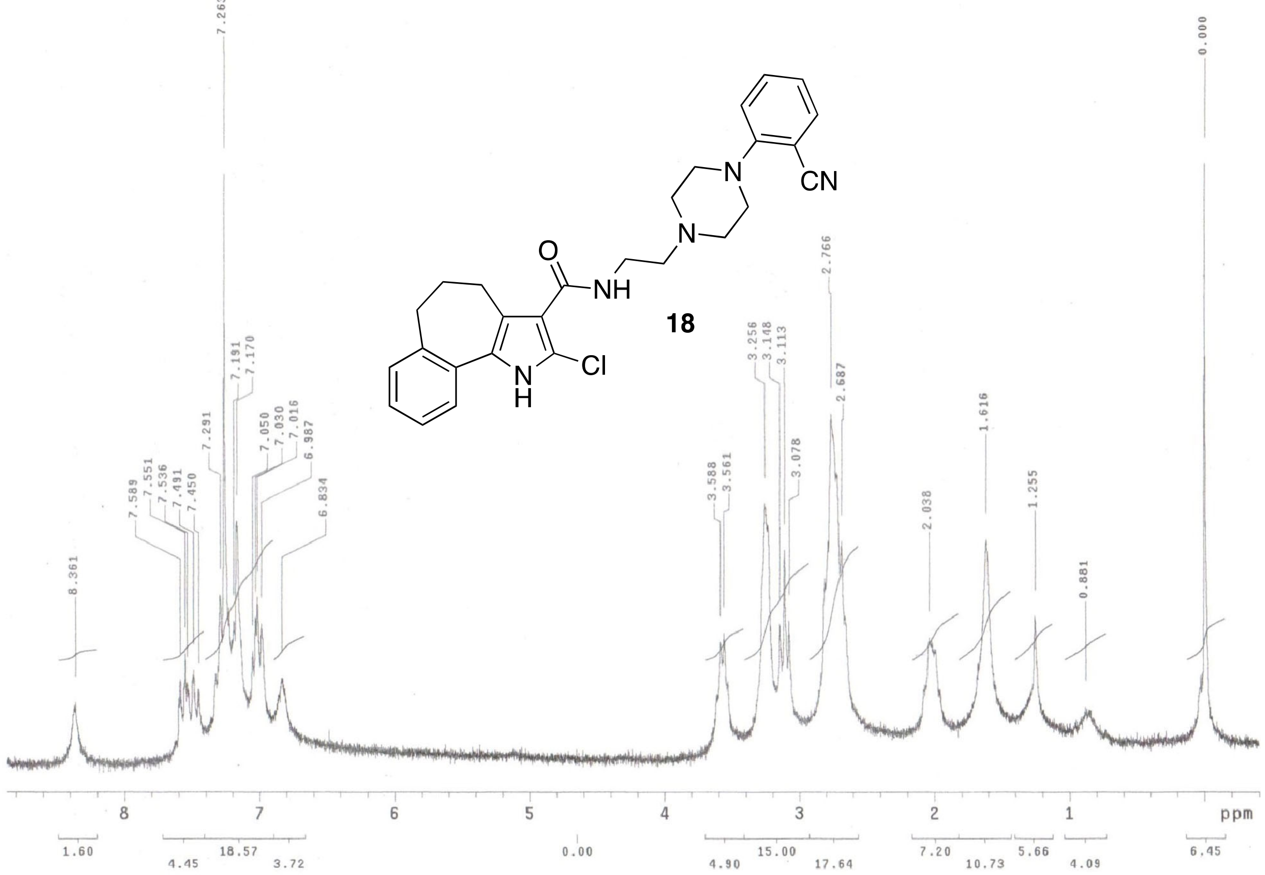

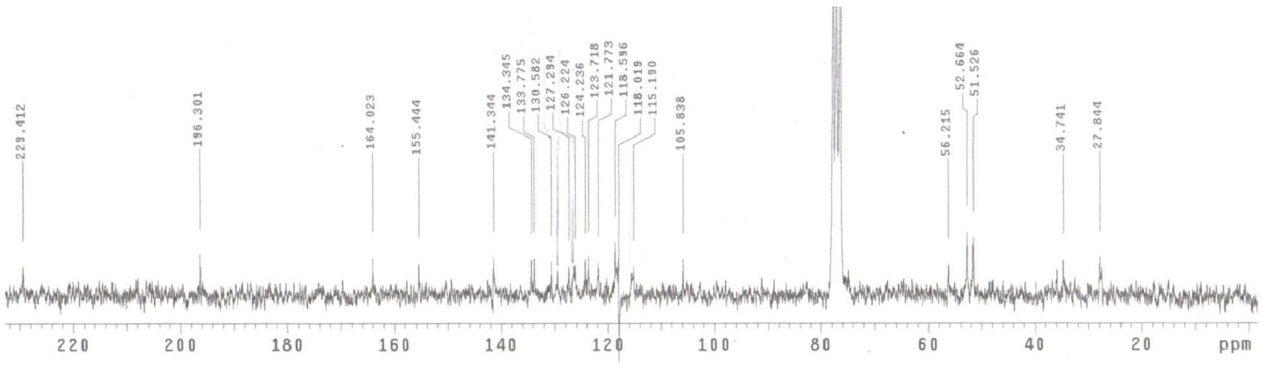

Figure S21: ^{1}H NMR and ^{13}C APT spectra of compound **19**

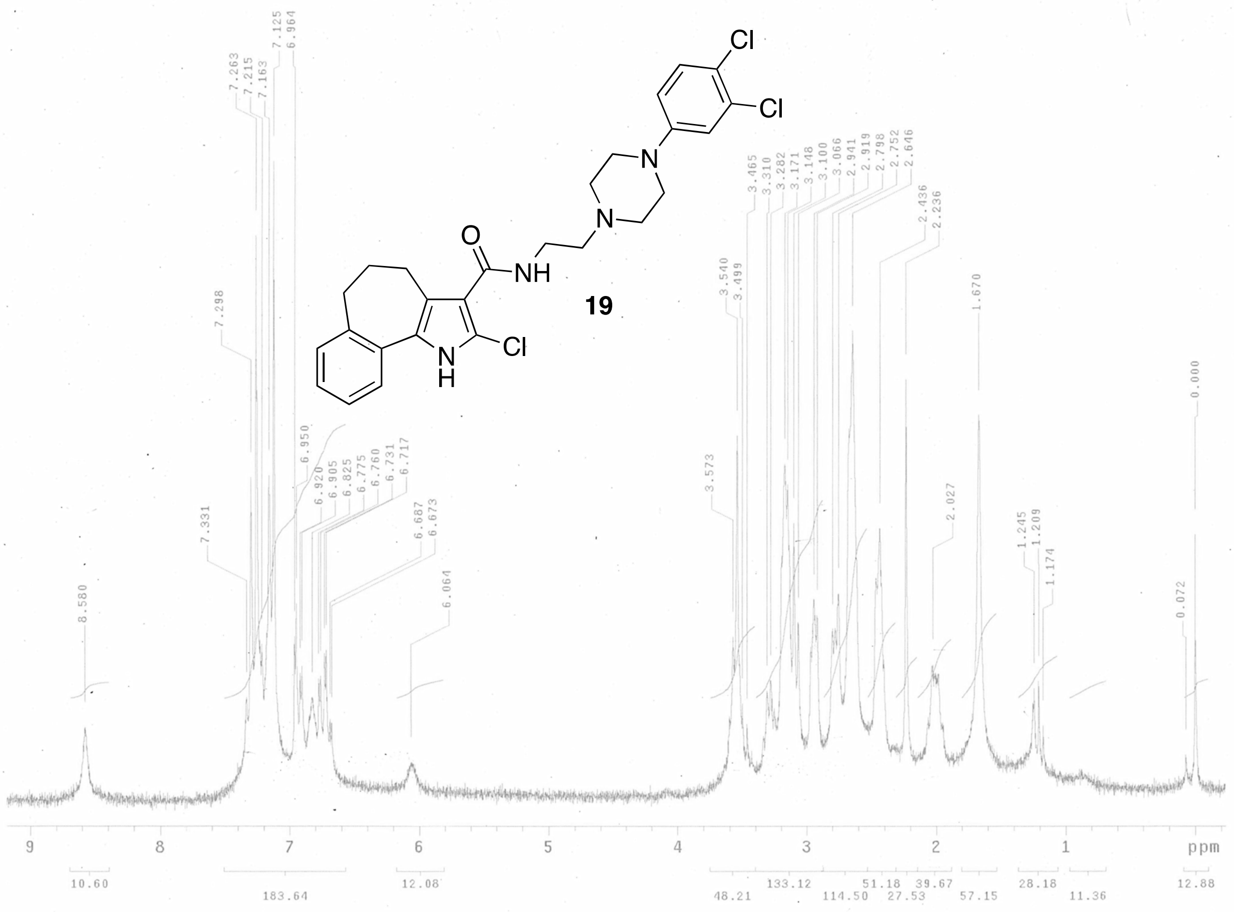

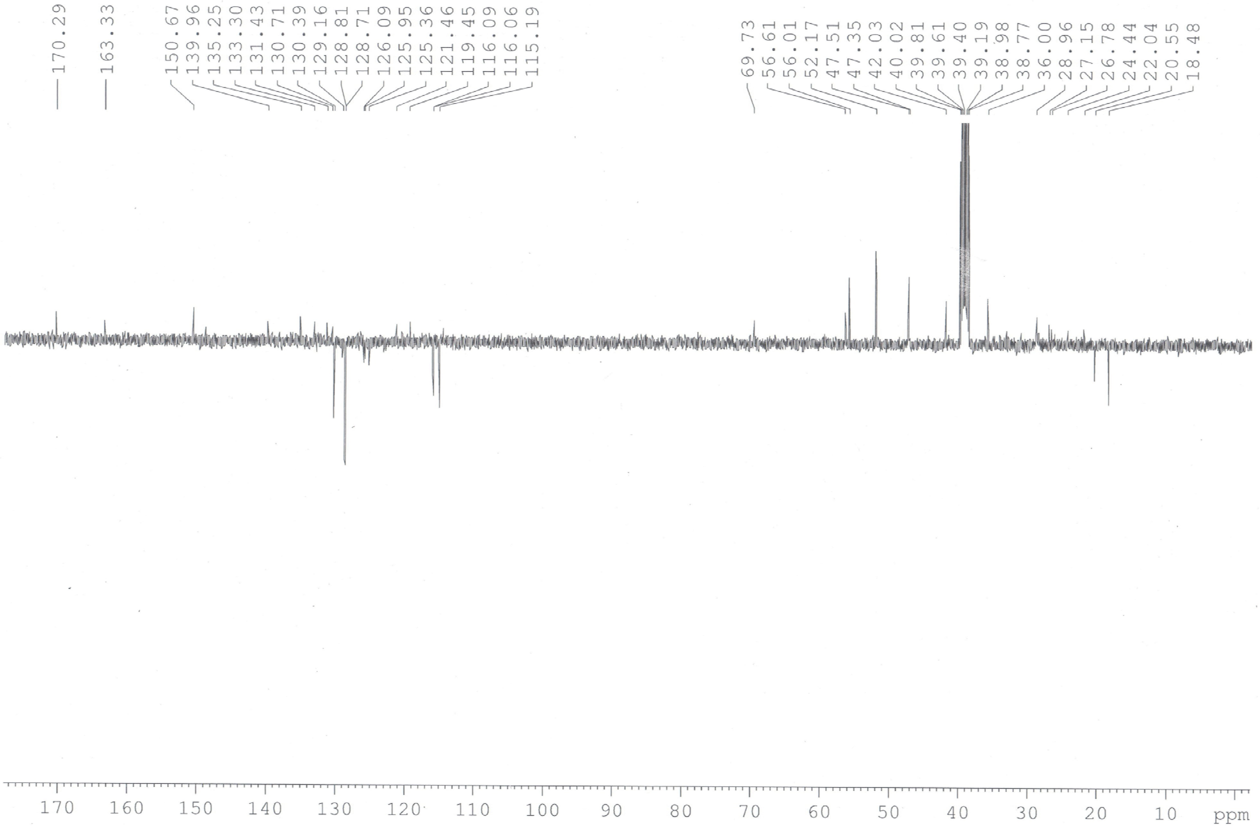

Figure S22: ^{1}H NMR and ^{13}C APT spectra of compound **20**

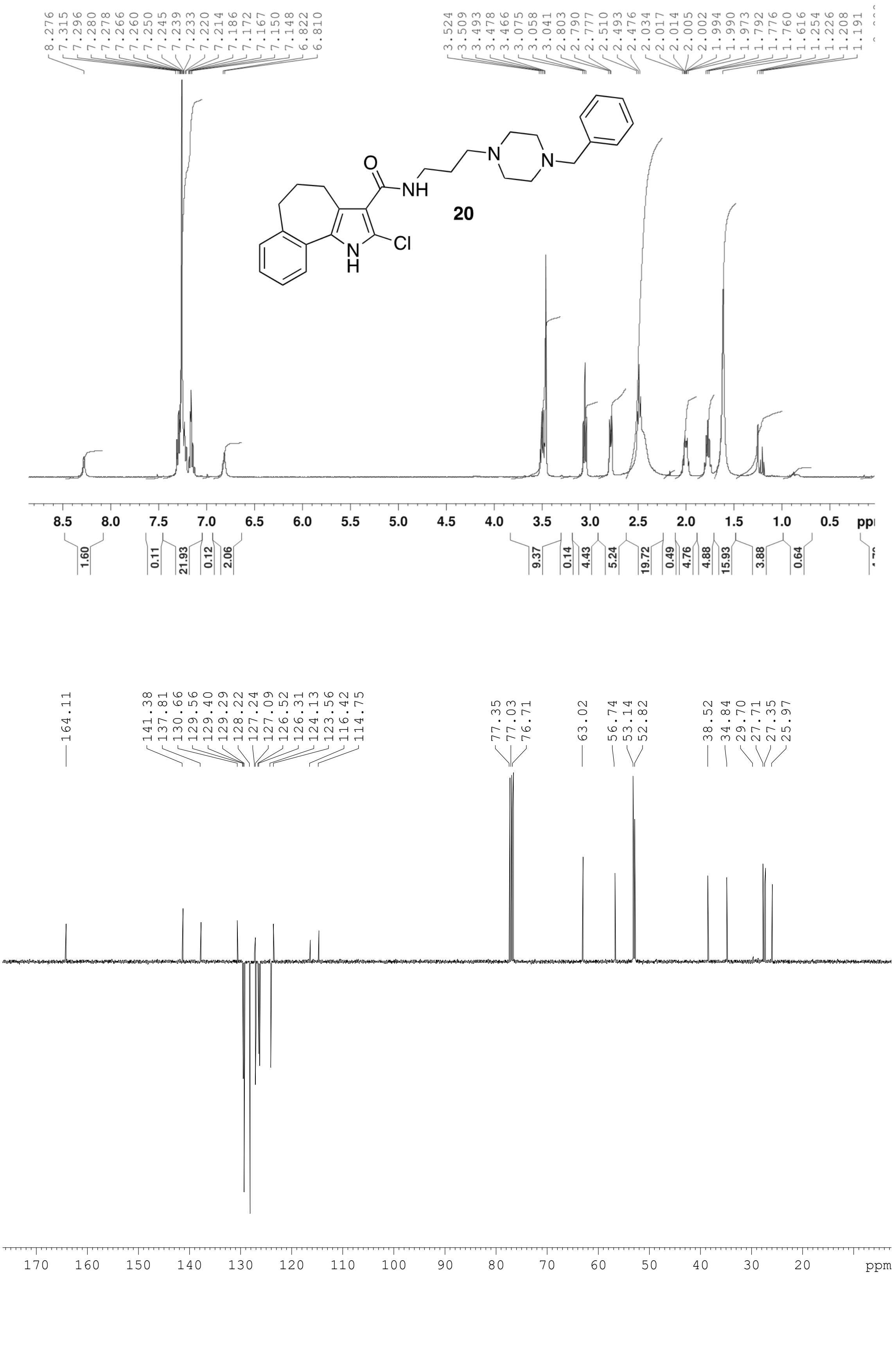

Figure S23: mass spectrum of compound **1**

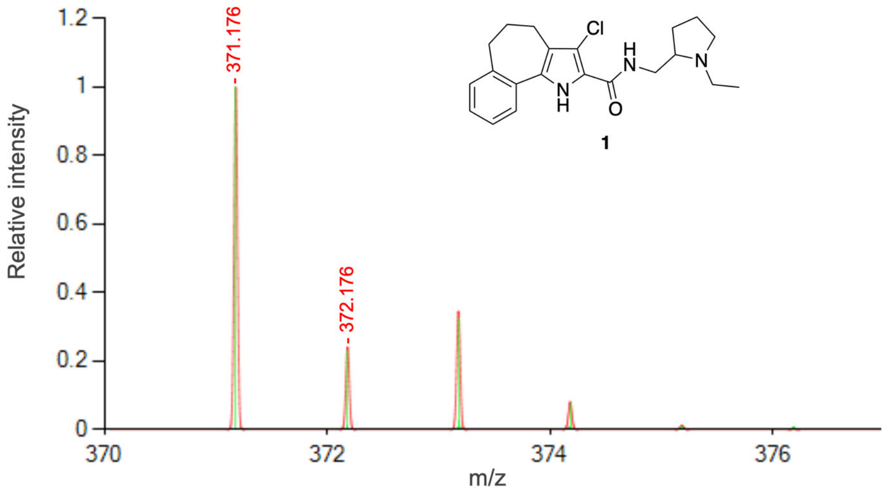

Figure S24: mass spectrum of compound **2**

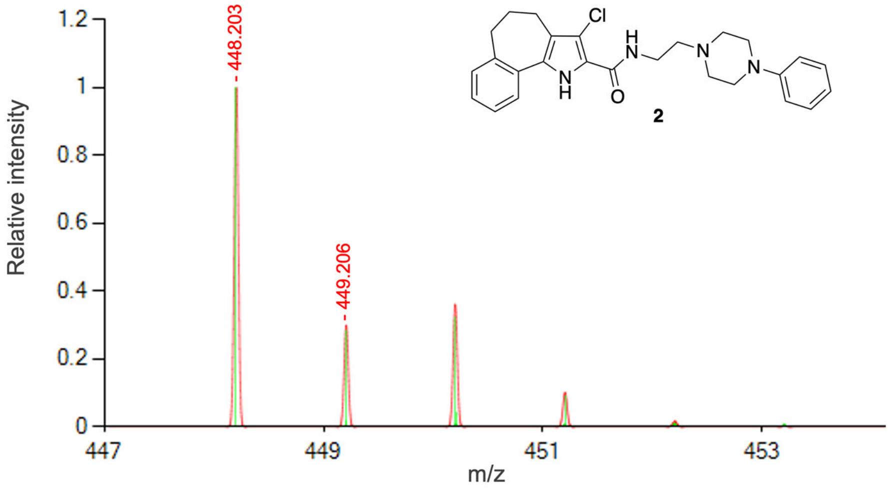

Figure S25: mass spectrum of compound **3**

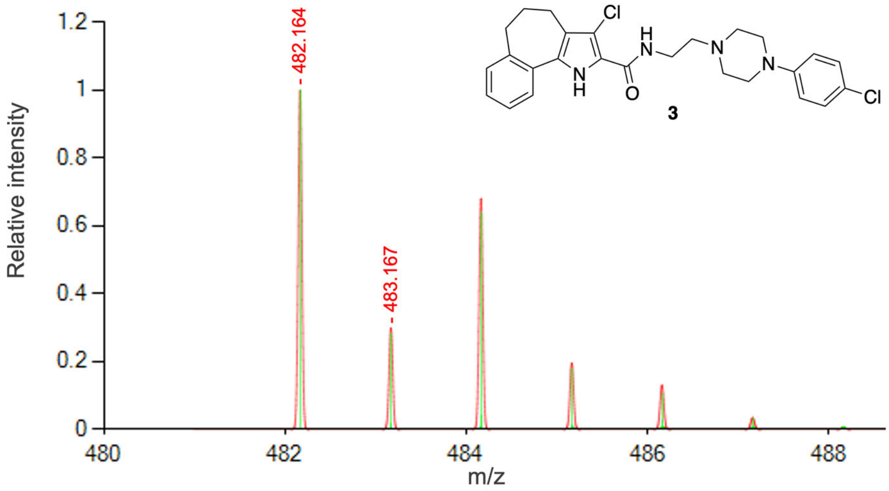

Figure S26: mass spectrum of compound **4**

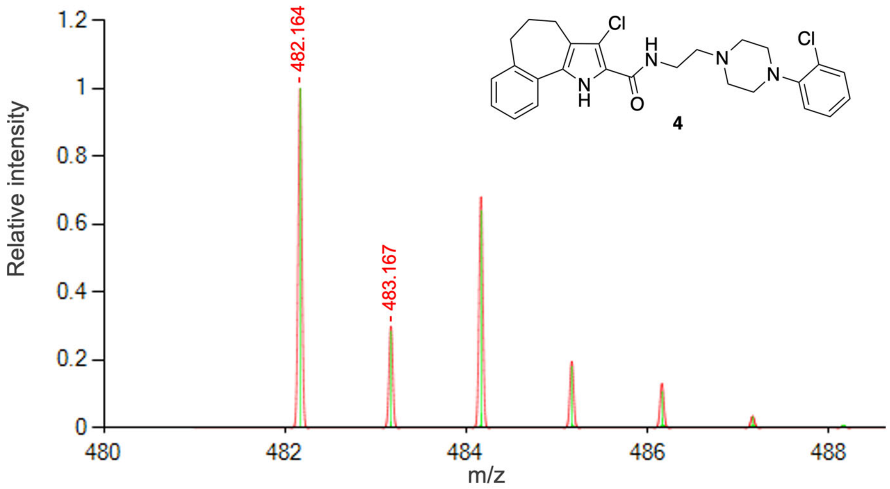

Figure S27: mass spectraum of compound **5**

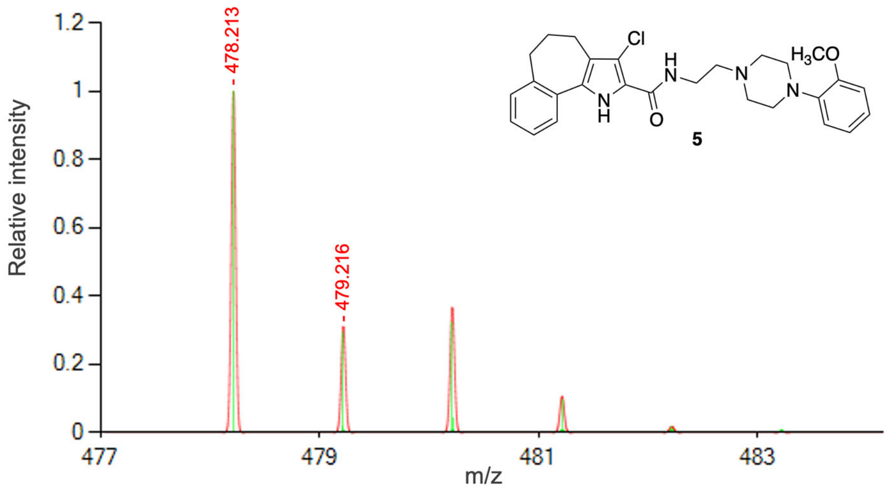

Figure S28: mass spectrum of compound **6**

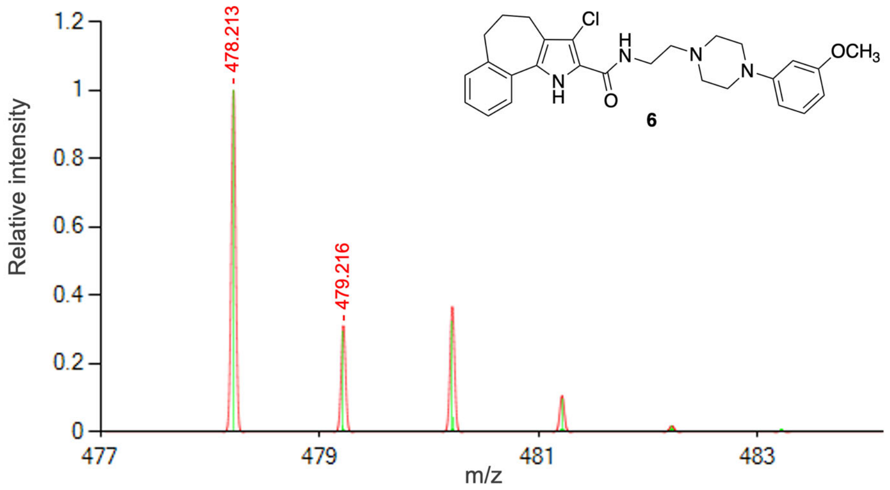

Figure S29: mass spectrum of compound **7**

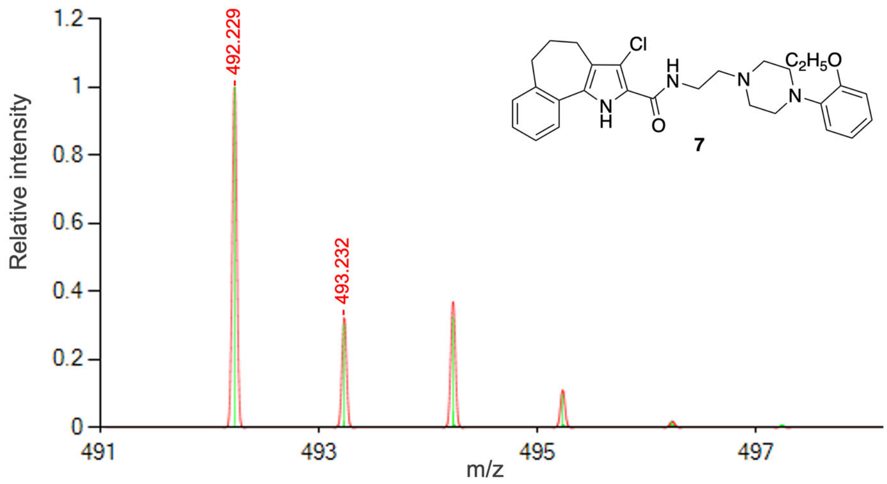

Figure S30: mass spectrum of compound **8**

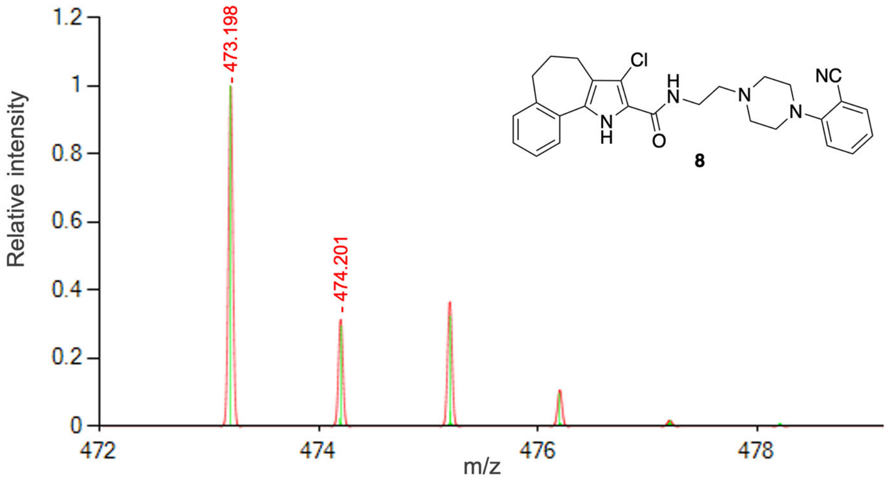

Figure S31: mass spectrum of compound **9**

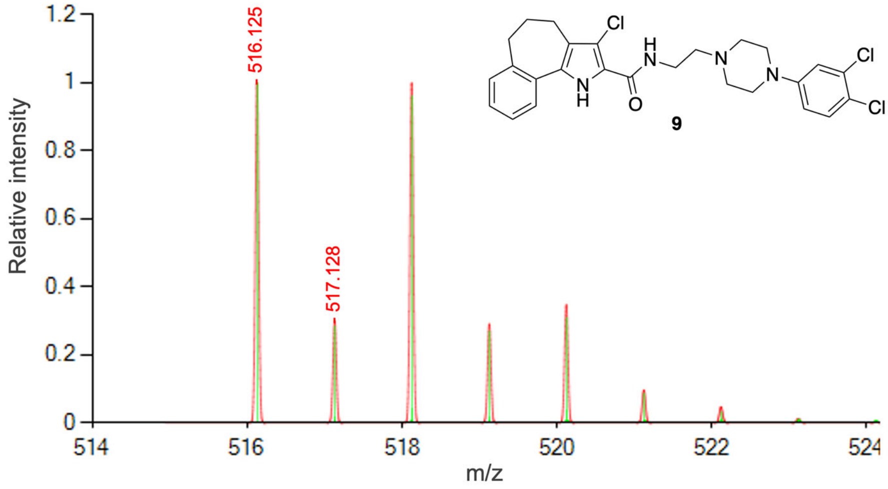

Figure S32: mass spectrum of compound **10**

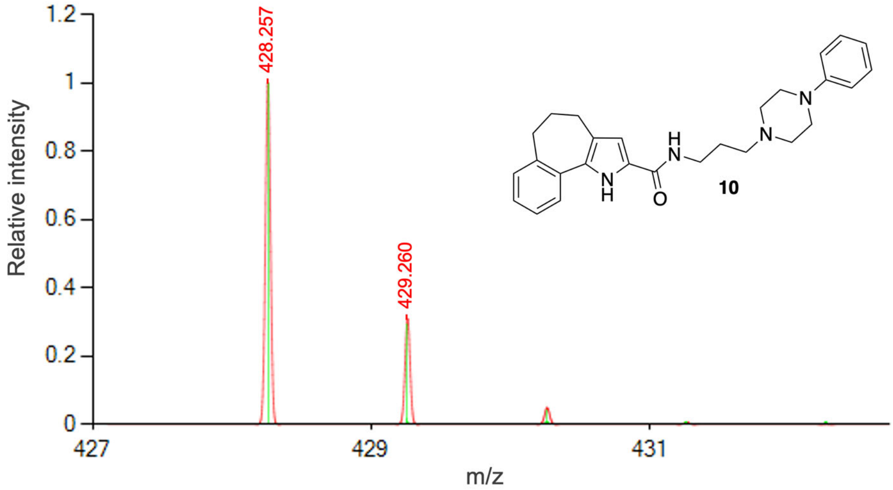

Figure S33: mass spectrum of compound **11**

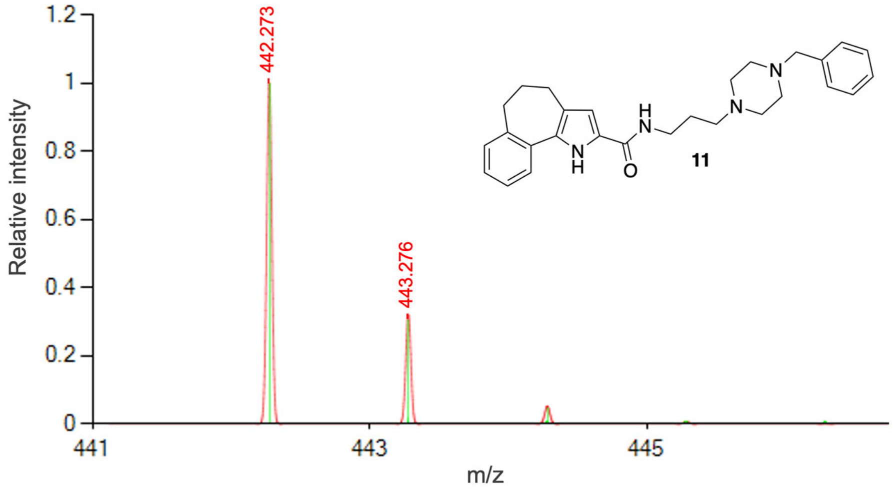

Figure S34: mass spectrum of compound **12**

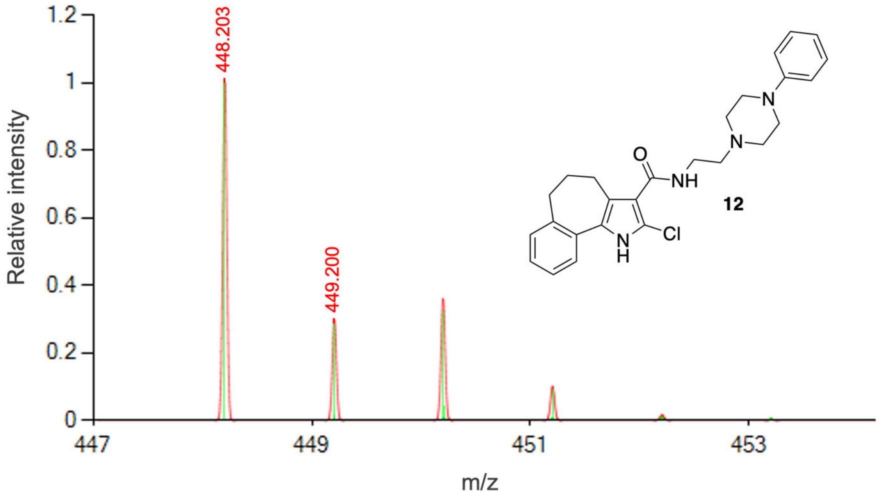

Figure S35: mass spectrum of compound **13**

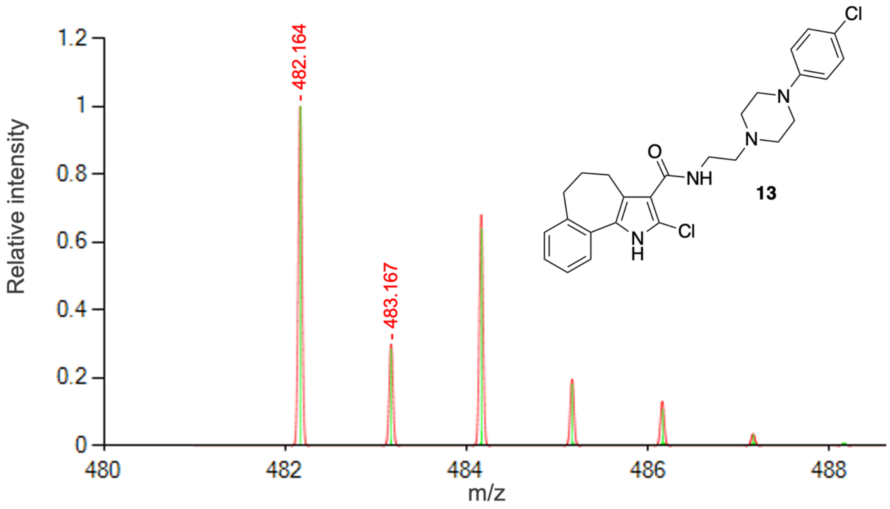

Figure S36: mass spectrum of compound **14**

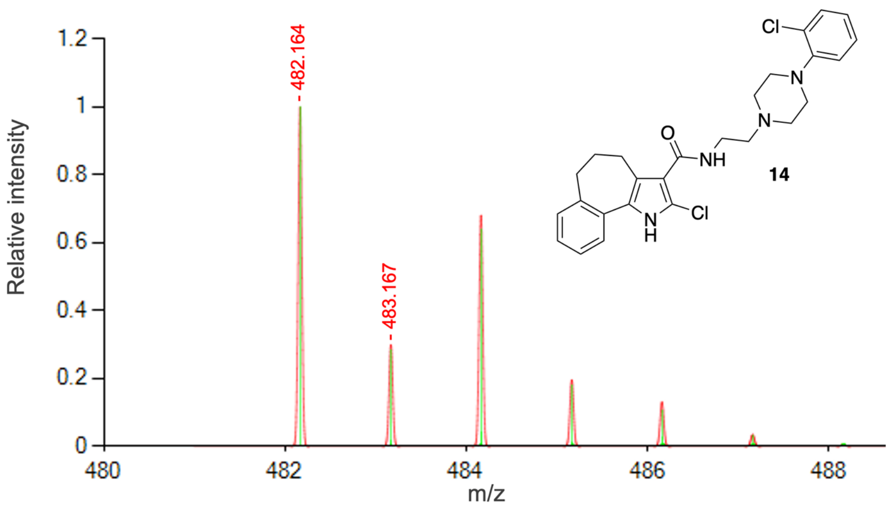

Figure S37: mass spectrum of compound **15**

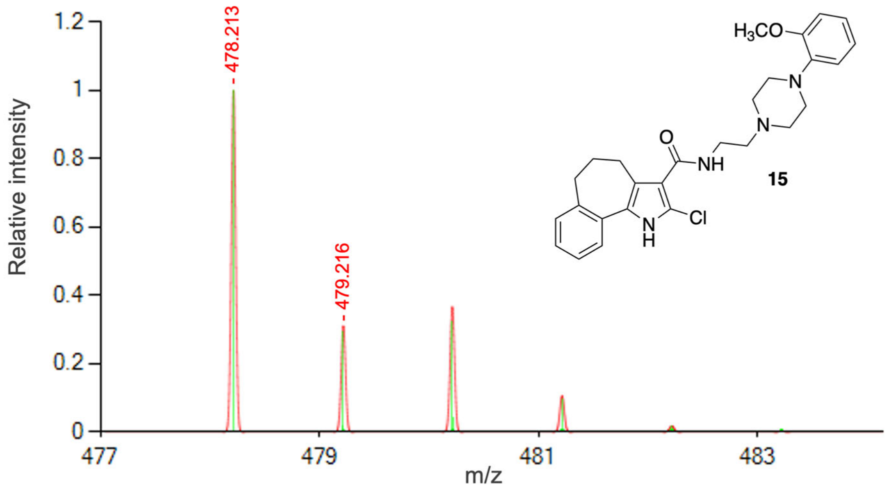

Figure S38: mass spectrum of compound **16**

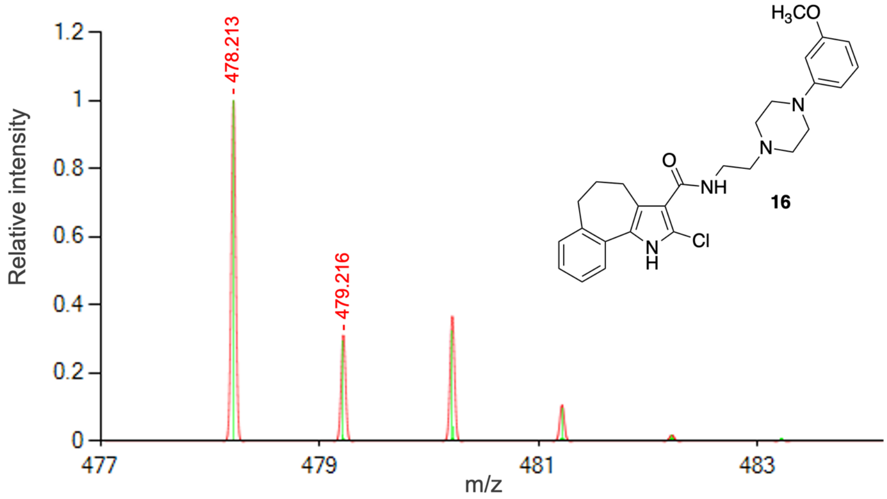

Figure S39: mass spectrum of compound **17**

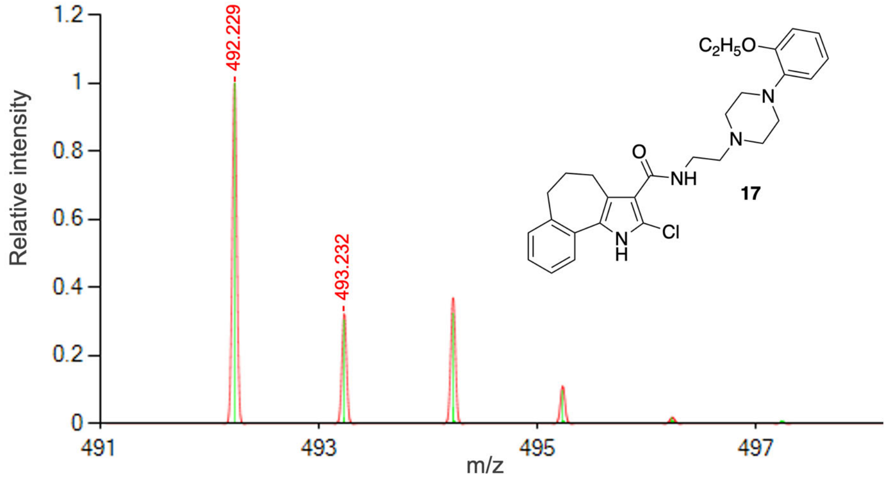

Figure S40: mass spectrum of compound **18**

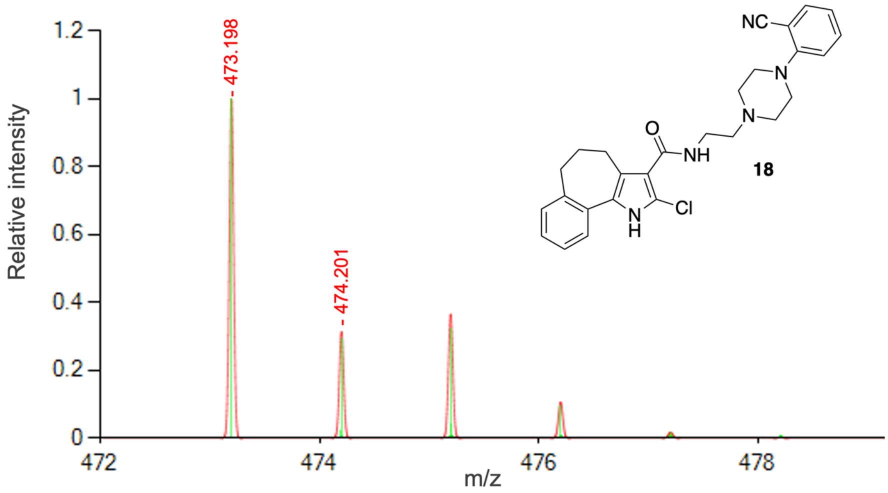

Figure S41: mass spectrum of compound **19**

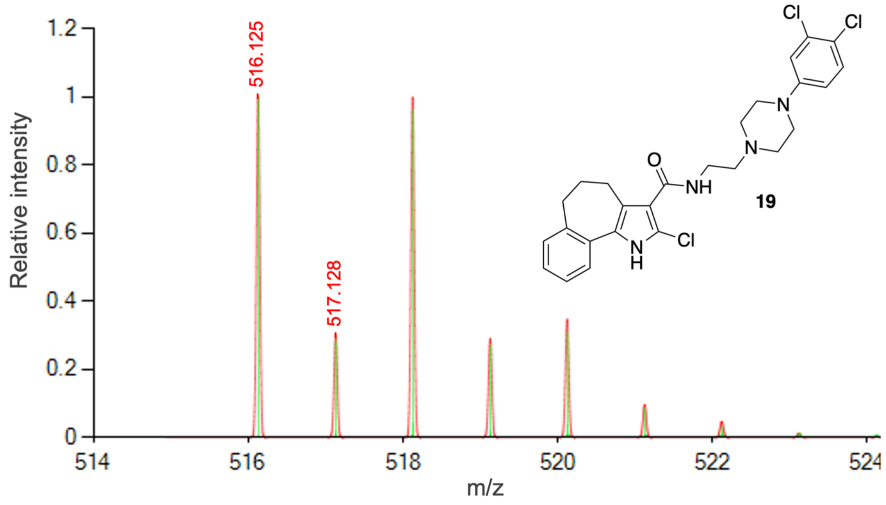

Figure S42: mass spectrum of compound **20**

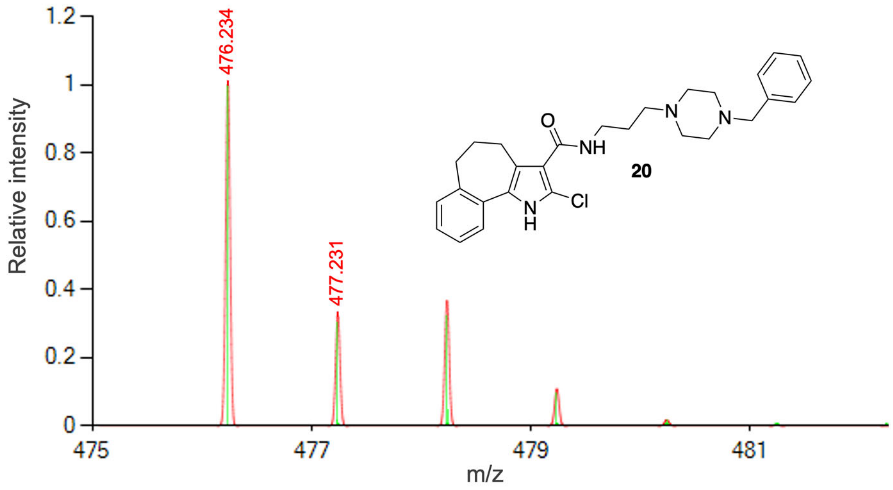

Figure S43: one-dose graph of compound **1** (NSC 846162)

Developmental Therapeutics Program
One Dose Mean Graph

NSC: D-846162 / 1 | **Conc:** 1.00E-5 Molar | **Test Date:** Jul 10, 2023

Experiment ID: 2307OS44 | **Report Date:** Sep 01, 2025

Panel/Cell Line	Growth Percent
Leukemia	
CCRF-CEM	24.52
HL-60(TB)	-38.55
K-562	6.02
MOLT-4	3.81
RPMI-8226	1.36
SR	3.72
Non-Small Cell Lung Cancer	
A549/ATCC	40.64
EKVX	31.23
HOP-62	42.37
NCI-H226	49.54
NCI-H23	54.81
NCI-H322M	67.86
NCI-H460	-54.58
NCI-H522	26.51
Colon Cancer	
COLO 205	-52.22
HCC-2998	-21.02
HCT-116	-25.27
HCT-15	-52.66
HT29	-32.25
KM12	16.17
SW-620	-30.78
CNS Cancer	
SF-268	64.93
SF-295	-24.35
SF-539	18.07
SNB-75	24.32
U251	33.58
Melanoma	
LOX IMVI	-25.00
MALME-3M	-90.67
M14	-68.50
MDA-MB-435	-78.15
SK-MEL-2	14.19
SK-MEL-28	-85.77
SK-MEL-5	-88.03
UACC-257	-55.18
UACC-62	-73.43
Ovarian Cancer	
IGROV1	35.49
OVCAR-3	60.92
OVCAR-4	63.61
OVCAR-5	34.30
OVCAR-8	71.41
NCI/ADR-RES	63.01
SK-OV-3	48.85
Renal Cancer	
786-0	-22.61
A498	75.68
ACHN	40.69
CAKI-1	-27.03
RXF 393	20.33
SN12C	63.83
UO-31	17.00
Prostate Cancer	
PC-3	47.51
DU-145	36.90
Breast Cancer	
MCF7	12.77
MDA-MB-231/ATCC	11.03
HS 578T	49.79
BT-549	37.13
T-47D	33.78
MDA-MB-468	-3.75
Mean	6.98
Delta	97.65
Range	166.35

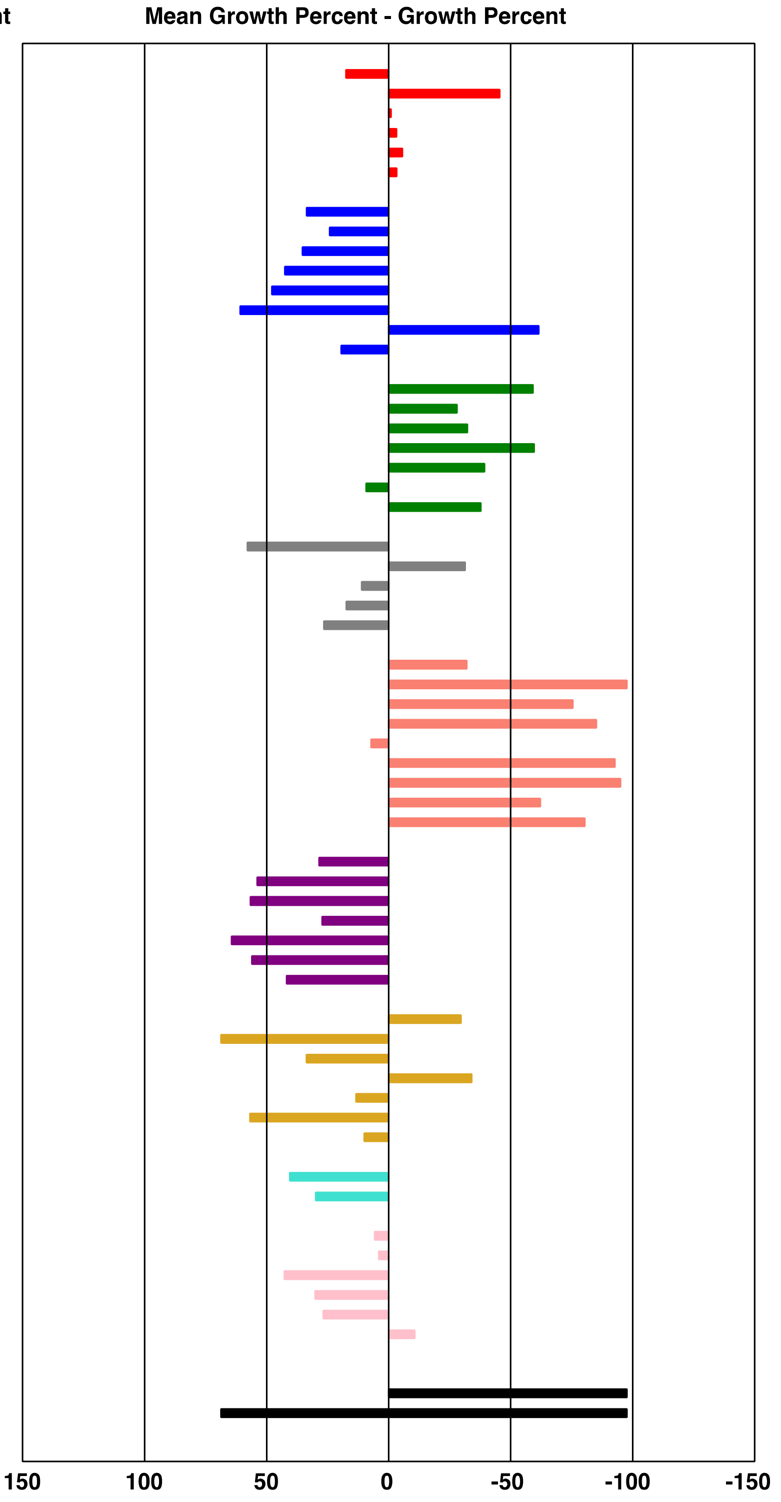

Figure S44: one-dose graph of compound **2** (NSC 846163)

Developmental Therapeutics Program One Dose Mean Graph	NSC: D-846163 / 1	Conc: 1.00E-5 Molar	Test Date: Jul 10, 2023
	Experiment ID: 2307OS44		Report Date: Aug 11, 2023

Panel/Cell Line	Growth Percent
Leukemia	
CCRF-CEM	107.24
HL-60(TB)	87.12
K-562	60.61
MOLT-4	79.05
RPMI-8226	73.64
SR	89.12
Non-Small Cell Lung Cancer	
A549/ATCC	100.84
EKVX	82.10
HOP-62	100.55
NCI-H226	85.00
NCI-H23	83.46
NCI-H322M	121.27
NCI-H460	97.43
NCI-H522	88.38
Colon Cancer	
COLO 205	102.73
HCC-2998	107.74
HCT-116	83.23
HCT-15	85.19
HT29	77.63
KM12	96.95
SW-620	100.90
CNS Cancer	
SF-268	97.76
SF-295	91.56
SF-539	103.24
SNB-75	83.37
U251	91.37
Melanoma	
LOX IMVI	85.25
MALME-3M	94.24
M14	99.60
MDA-MB-435	93.77
SK-MEL-2	104.28
SK-MEL-28	106.13
SK-MEL-5	76.93
UACC-257	113.57
UACC-62	77.92
Ovarian Cancer	
IGROV1	94.01
OVCAR-3	108.92
OVCAR-4	95.38
OVCAR-5	109.79
OVCAR-8	92.86
NCI/ADR-RES	89.07
SK-OV-3	95.13
Renal Cancer	
786-0	105.57
A498	116.79
ACHN	100.57
CAKI-1	81.05
RXF 393	104.57
SN12C	97.81
UO-31	70.64
Prostate Cancer	
PC-3	95.35
DU-145	99.00
Breast Cancer	
MCF7	75.62
MDA-MB-231/ATCC	99.33
HS 578T	107.23
BT-549	105.81
T-47D	85.18
MDA-MB-468	120.13
Mean	94.37
Delta	33.76
Range	60.66

Mean Growth Percent - Growth Percent

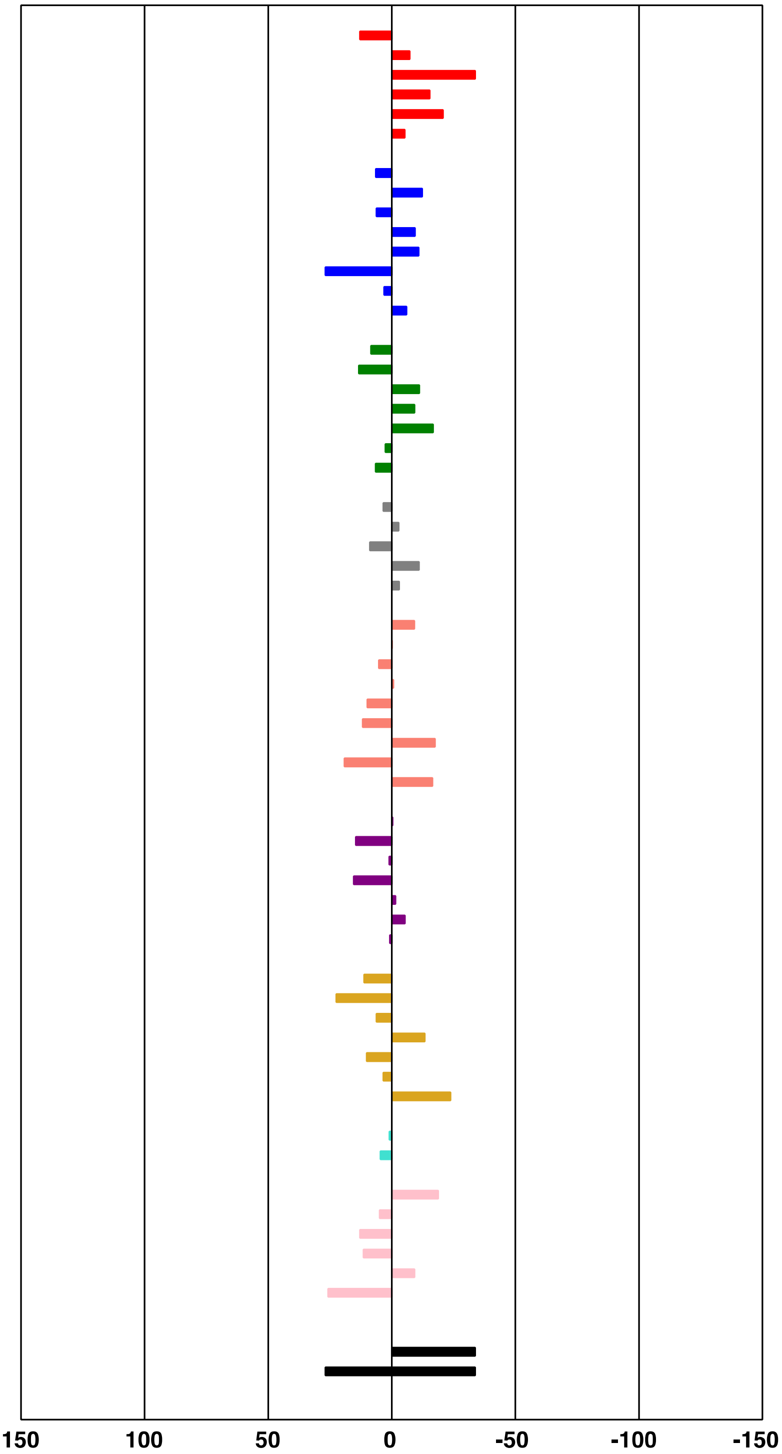

Figure S45: one-dose graph of compound **3** (NSC 846164)

Developmental Therapeutics Program One Dose Mean Graph	NSC: D-846164 / 1	Conc: 1.00E-5 Molar	Test Date: Jul 10, 2023
	Experiment ID: 2307OS44		Report Date: Aug 11, 2023

Panel/Cell Line	Growth Percent
Leukemia	
CCRF-CEM	86.17
HL-60(TB)	82.26
K-562	60.56
MOLT-4	75.73
RPMI-8226	70.42
SR	75.09
Non-Small Cell Lung Cancer	
A549/ATCC	100.61
EKVX	82.50
HOP-62	83.51
NCI-H226	80.31
NCI-H23	85.81
NCI-H322M	122.13
NCI-H460	100.47
NCI-H522	87.68
Colon Cancer	
COLO 205	99.41
HCC-2998	105.07
HCT-116	83.99
HCT-15	83.25
HT29	75.00
KM12	97.76
SW-620	95.41
CNS Cancer	
SF-268	97.06
SF-295	96.98
SF-539	103.38
SNB-75	80.82
U251	101.63
Melanoma	
LOX IMVI	85.94
MALME-3M	98.91
M14	96.57
MDA-MB-435	92.66
SK-MEL-2	103.22
SK-MEL-28	110.01
SK-MEL-5	79.34
UACC-257	107.76
UACC-62	79.47
Ovarian Cancer	
IGROV1	93.86
OVCAR-3	97.83
OVCAR-4	88.63
OVCAR-5	106.53
OVCAR-8	97.44
NCI/ADR-RES	92.47
SK-OV-3	91.79
Renal Cancer	
786-0	106.33
A498	135.75
ACHN	100.24
CAKI-1	80.93
RXF 393	116.49
SN12C	90.76
UO-31	69.89
Prostate Cancer	
PC-3	78.61
DU-145	107.12
Breast Cancer	
MCF7	73.82
MDA-MB-231/ATCC	89.85
HS 578T	124.93
BT-549	102.47
T-47D	84.16
MDA-MB-468	99.73
Mean	92.92
Delta	32.36
Range	75.19

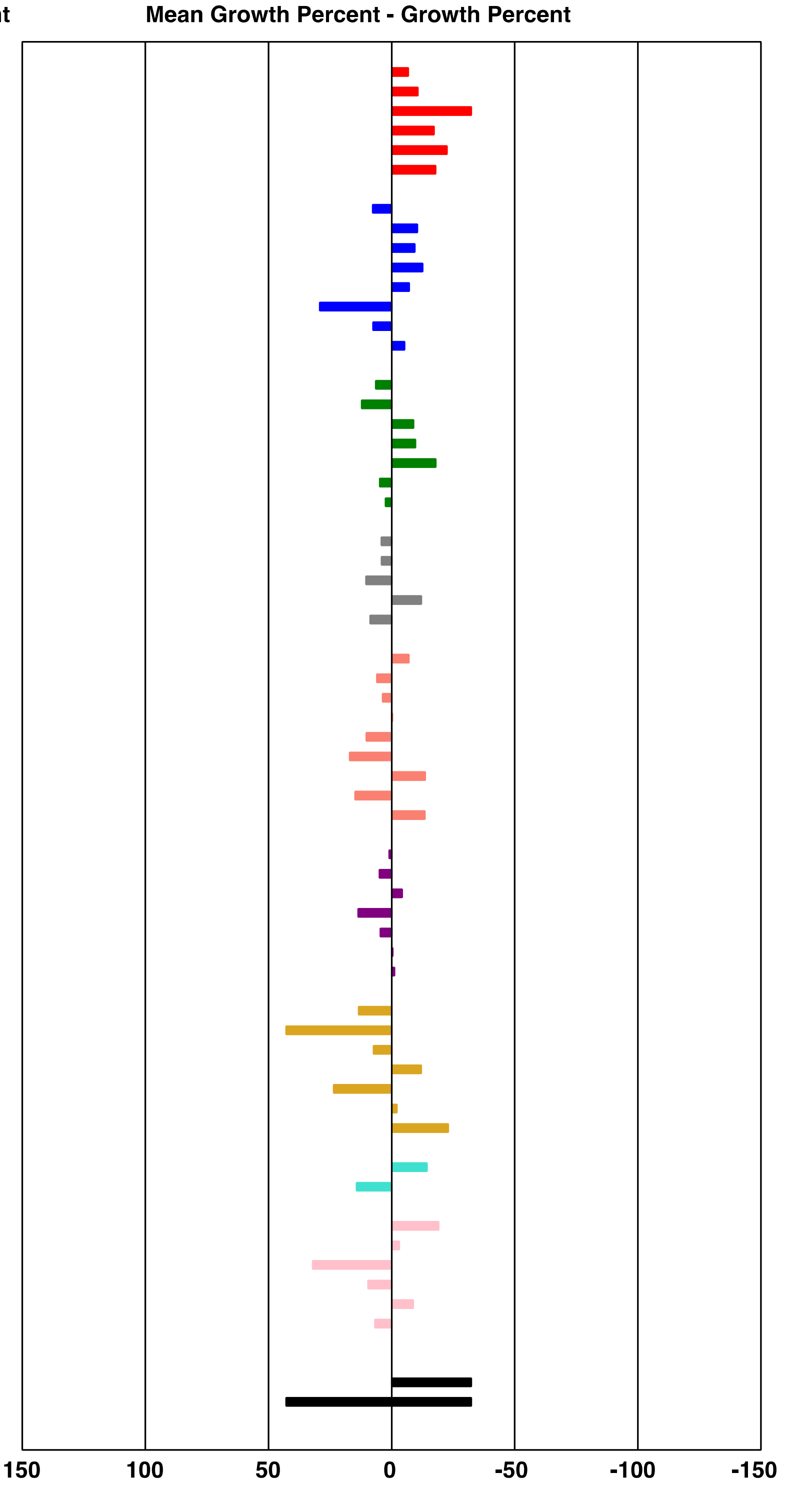

Figure S46: one-dose graph of compound **4** (NSC 846165)

Developmental Therapeutics Program

One Dose Mean Graph

**NSC:** D-846165 / 1	**Conc:** 1.00E-5 Molar	**Test Date:** Jul 10, 2023	
**Experiment ID:** 2307OS44		**Report Date:** Aug 11, 2023	

Panel/Cell Line	Growth Percent
Leukemia	
CCRF-CEM	72.41
HL-60(TB)	64.43
K-562	42.61
MOLT-4	54.27
RPMI-8226	53.60
SR	47.95
Non-Small Cell Lung Cancer	
A549/ATCC	87.58
EKVX	73.01
HOP-62	85.83
NCI-H226	71.74
NCI-H23	70.73
NCI-H322M	105.60
NCI-H460	87.84
NCI-H522	75.31
Colon Cancer	
COLO 205	70.68
HCC-2998	89.26
HCT-116	58.60
HCT-15	56.22
HT29	48.38
KM12	80.24
SW-620	77.56
CNS Cancer	
SF-268	88.22
SF-295	96.10
SF-539	95.89
SNB-75	73.53
U251	83.85
Melanoma	
LOX IMVI	76.72
MALME-3M	97.34
M14	75.09
MDA-MB-435	80.35
SK-MEL-2	93.76
SK-MEL-28	94.53
SK-MEL-5	53.54
UACC-257	95.49
UACC-62	75.49
Ovarian Cancer	
IGROV1	80.72
OVCAR-3	100.49
OVCAR-4	72.14
OVCAR-5	87.45
OVCAR-8	83.14
NCI/ADR-RES	76.14
SK-OV-3	98.45
Renal Cancer	
786-0	86.39
A498	122.44
ACHN	94.58
CAKI-1	69.80
RXF 393	83.36
SN12C	57.33
UO-31	66.43
Prostate Cancer	
PC-3	61.09
DU-145	84.37
Breast Cancer	
MCF7	39.98
MDA-MB-231/ATCC	86.59
HS 578T	84.02
BT-549	82.71
T-47D	67.10
MDA-MB-468	89.34
Mean	77.68
Delta	37.70
Range	82.46

Mean Growth Percent - Growth Percent

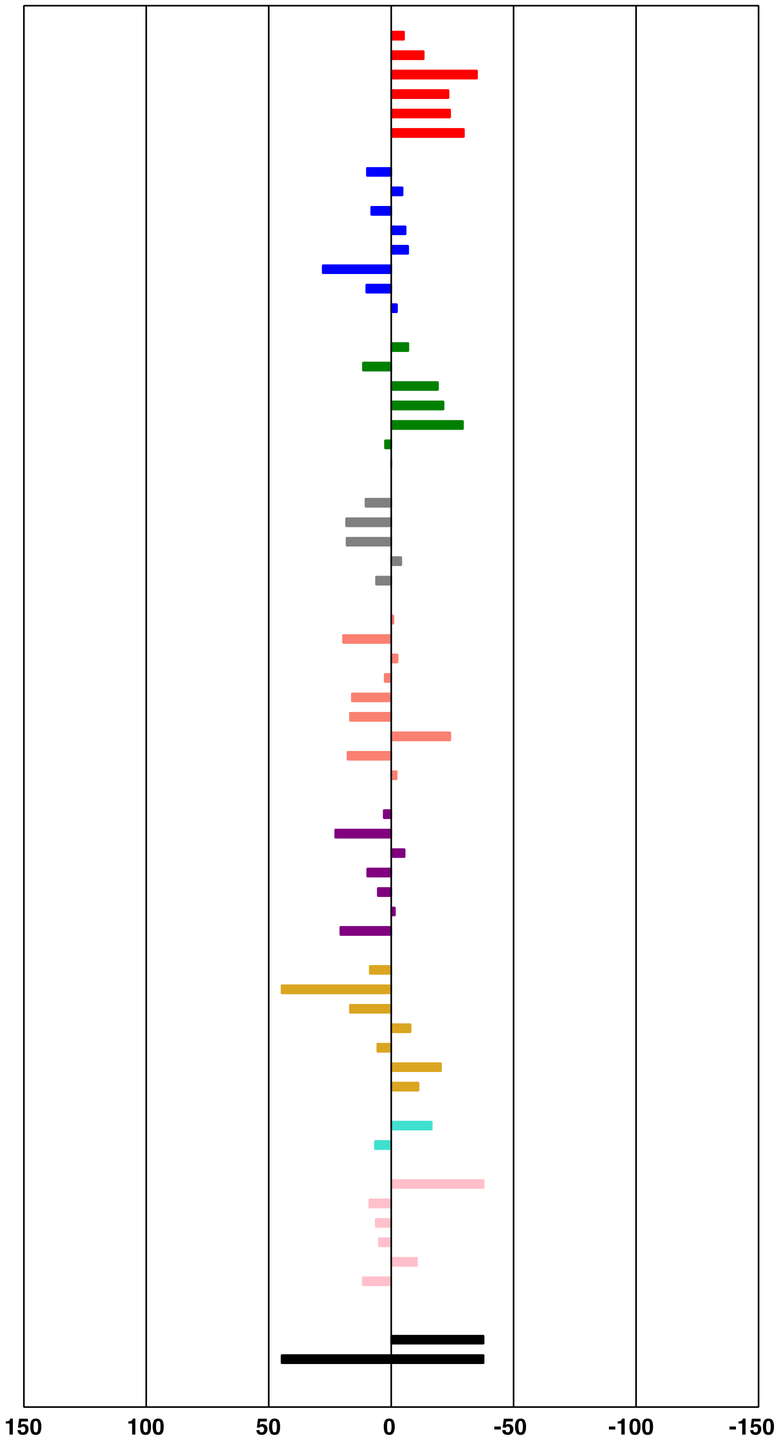

Figure S47: one-dose graph of compound **5** (NSC 846166)

Developmental Therapeutics Program One Dose Mean Graph	NSC: D-846166 / 1	Conc: 1.00E-5 Molar	Test Date: Jul 10, 2023
	Experiment ID: 2307OS44		Report Date: Aug 11, 2023

Panel/Cell Line	Growth Percent
Leukemia	
CCRF-CEM	28.36
HL-60(TB)	-8.91
K-562	4.19
MOLT-4	7.02
RPMI-8226	31.90
SR	9.91
Non-Small Cell Lung Cancer	
A549/ATCC	42.57
EKVX	5.77
HOP-62	66.10
NCI-H226	43.47
NCI-H23	36.79
NCI-H322M	110.55
NCI-H460	20.84
NCI-H522	14.56
Colon Cancer	
COLO 205	-48.74
HCC-2998	40.36
HCT-116	5.36
HCT-15	7.54
HT29	-17.44
KM12	32.32
SW-620	32.57
CNS Cancer	
SF-268	57.09
SF-295	26.05
SF-539	31.10
SNB-75	53.51
U251	33.25
Melanoma	
LOX IMVI	17.18
MALME-3M	51.16
M14	-22.16
MDA-MB-435	38.16
SK-MEL-2	66.91
SK-MEL-28	50.02
SK-MEL-5	-62.59
UACC-257	54.19
UACC-62	52.19
Ovarian Cancer	
IGROV1	65.31
OVCAR-3	64.06
OVCAR-4	39.95
OVCAR-5	59.10
OVCAR-8	45.93
NCI/ADR-RES	31.18
SK-OV-3	68.12
Renal Cancer	
786-0	37.01
A498	87.77
ACHN	40.37
CAKI-1	22.13
RXF 393	10.58
SN12C	28.18
UO-31	30.01
Prostate Cancer	
PC-3	23.69
DU-145	60.33
Breast Cancer	
MCF7	18.40
MDA-MB-231/ATCC	50.33
HS 578T	53.43
BT-549	30.40
T-47D	32.10
MDA-MB-468	11.16
Mean	31.94
Delta	94.53
Range	173.14

Mean Growth Percent - Growth Percent

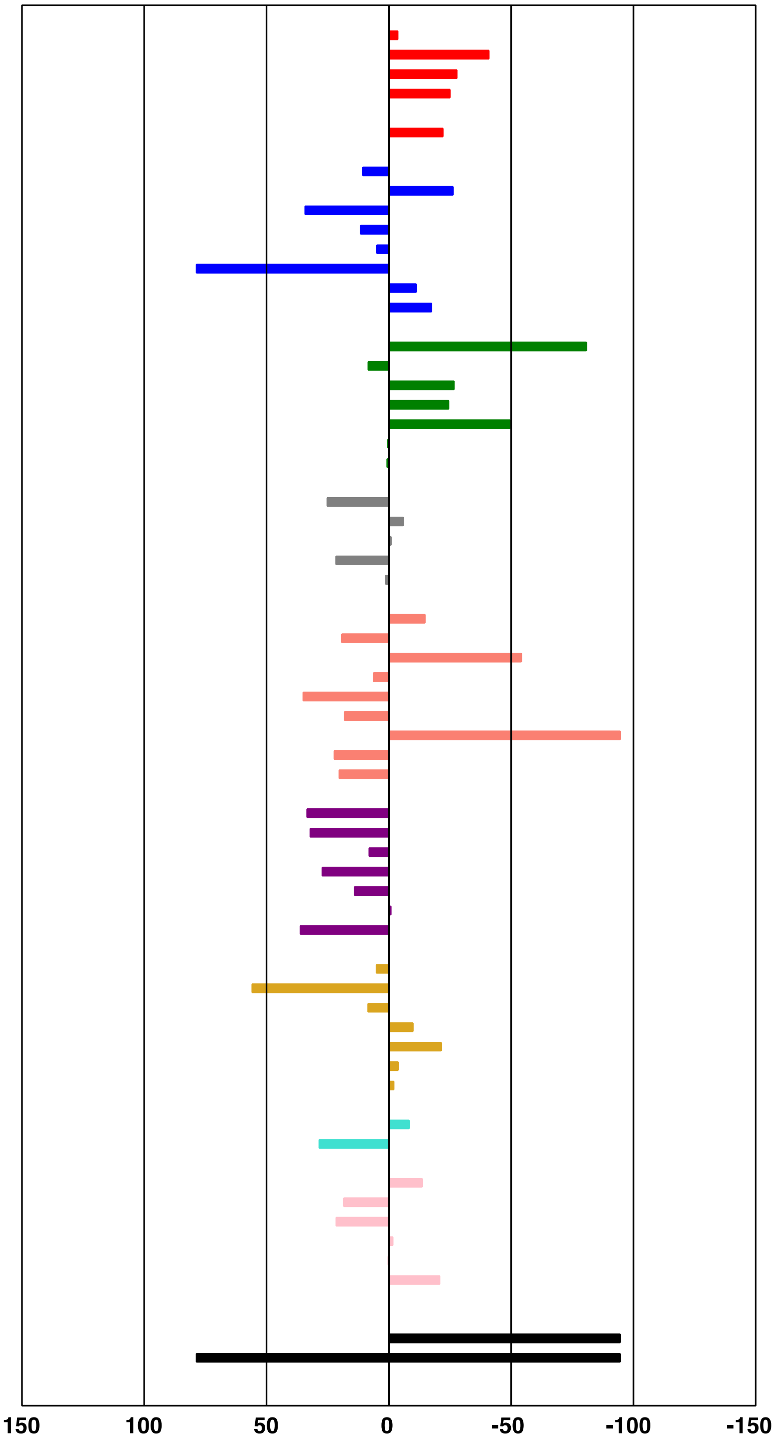

Figure S48: one-dose graph of compound **6** (NSC 846167)

Developmental Therapeutics Program One Dose Mean Graph	NSC: D-846167 / 1	Conc: 1.00E-5 Molar	Test Date: Jul 10, 2023
	Experiment ID: 2307OS44		Report Date: Aug 11, 2023

Panel/Cell Line	Growth Percent
Leukemia	
CCRF-CEM	87.78
HL-60(TB)	65.23
K-562	44.65
MOLT-4	61.40
RPMI-8226	62.76
SR	66.09
Non-Small Cell Lung Cancer	
A549/ATCC	67.35
EKVX	57.24
HOP-62	67.12
NCI-H226	58.71
NCI-H23	68.52
NCI-H322M	117.54
NCI-H460	68.73
NCI-H522	58.11
Colon Cancer	
COLO 205	87.12
HCC-2998	87.08
HCT-116	36.03
HCT-15	56.93
HT29	40.25
KM12	93.57
SW-620	80.11
CNS Cancer	
SF-268	83.82
SF-295	68.49
SF-539	82.01
SNB-75	64.45
U251	84.11
Melanoma	
LOX IMVI	73.51
MALME-3M	99.94
M14	83.07
MDA-MB-435	79.40
SK-MEL-2	97.58
SK-MEL-28	92.21
SK-MEL-5	50.49
UACC-257	104.95
UACC-62	81.21
Ovarian Cancer	
IGROV1	102.79
OVCAR-3	89.21
OVCAR-4	69.81
OVCAR-5	95.04
OVCAR-8	80.26
NCI/ADR-RES	62.60
SK-OV-3	114.51
Renal Cancer	
786-0	76.75
A498	119.42
ACHN	81.74
CAKI-1	65.97
RXF 393	70.38
SN12C	78.49
UO-31	73.09
Prostate Cancer	
PC-3	65.51
DU-145	86.30
Breast Cancer	
MCF7	58.15
MDA-MB-231/ATCC	77.69
HS 578T	51.61
BT-549	78.46
T-47D	70.40
MDA-MB-468	61.23
Mean	75.56
Delta	39.53
Range	83.39

Mean Growth Percent - Growth Percent

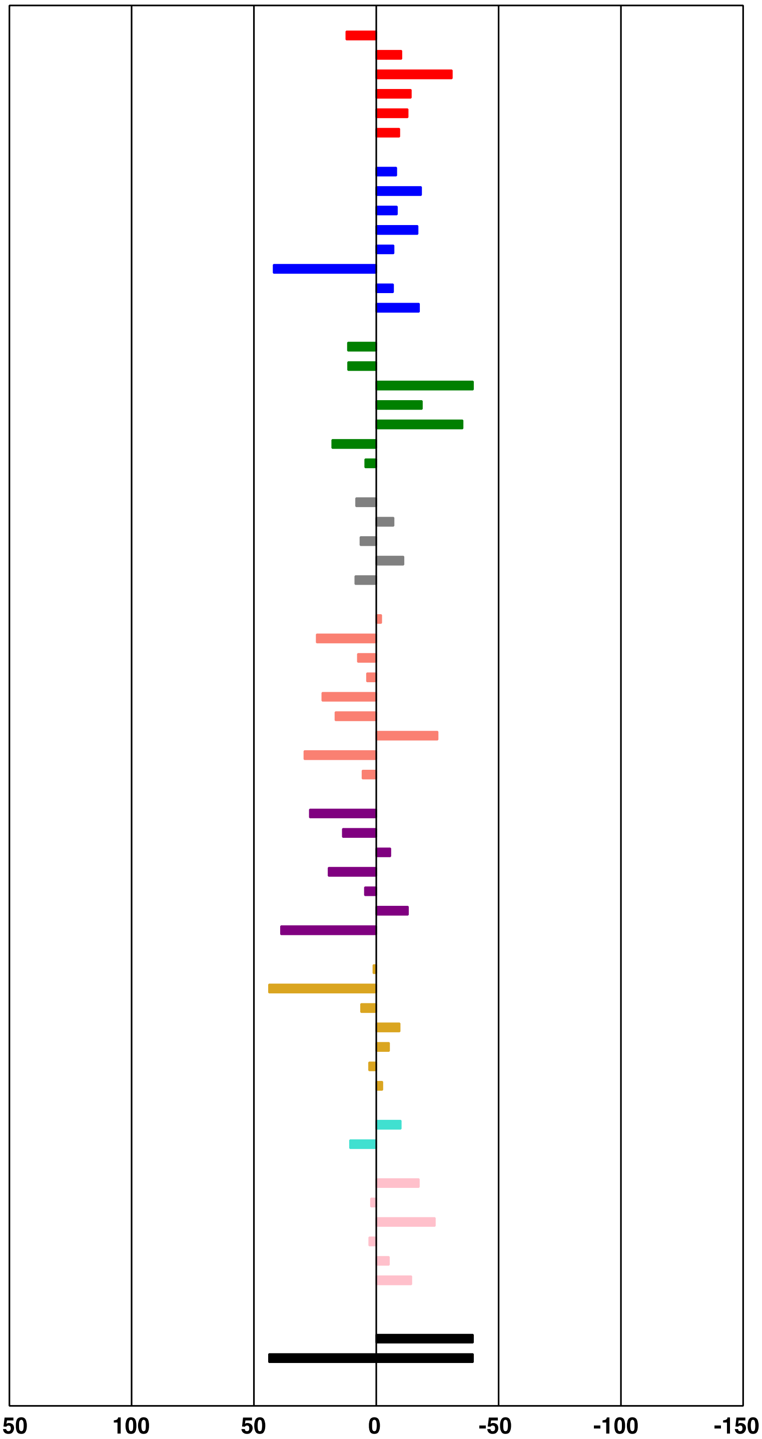

Figure S49: one-dose graph of compound **7** (NSC 846168)

Developmental Therapeutics Program One Dose Mean Graph	NSC: D-846168 / 1	Conc: 1.00E-5 Molar	Test Date: Jul 10, 2023
	Experiment ID: 2307OS44		Report Date: Aug 11, 2023

Panel/Cell Line	Growth Percent
**Leukemia**	
CCRF-CEM	50.52
HL-60(TB)	40.48
K-562	17.42
MOLT-4	33.46
RPMI-8226	36.61
SR	30.00
**Non-Small Cell Lung Cancer**	
A549/ATCC	65.46
EKVX	50.23
HOP-62	91.48
NCI-H226	58.59
NCI-H23	53.08
NCI-H322M	123.92
NCI-H460	39.24
NCI-H522	52.93
**Colon Cancer**	
COLO 205	11.20
HCC-2998	73.09
HCT-116	23.94
HCT-15	17.93
HT29	27.31
KM12	66.82
SW-620	45.32
**CNS Cancer**	
SF-268	83.89
SF-295	49.12
SF-539	82.57
SNB-75	91.12
U251	67.05
**Melanoma**	
LOX IMVI	45.04
MALME-3M	93.82
M14	11.37
MDA-MB-435	64.76
SK-MEL-2	88.17
SK-MEL-28	78.14
SK-MEL-5	22.29
UACC-257	90.50
UACC-62	70.23
**Ovarian Cancer**	
IGROV1	82.47
OVCAR-3	83.47
OVCAR-4	58.21
OVCAR-5	64.90
OVCAR-8	71.98
NCI/ADR-RES	56.92
SK-OV-3	100.36
**Renal Cancer**	
786-0	65.00
A498	113.84
ACHN	78.68
CAKI-1	43.89
RXF 393	62.90
SN12C	56.38
UO-31	47.17
**Prostate Cancer**	
PC-3	42.42
DU-145	77.02
**Breast Cancer**	
MCF7	41.30
MDA-MB-231/ATCC	79.24
HS 578T	71.44
BT-549	59.98
T-47D	49.90
MDA-MB-468	52.01
Mean	59.76
Delta	48.56
Range	112.72

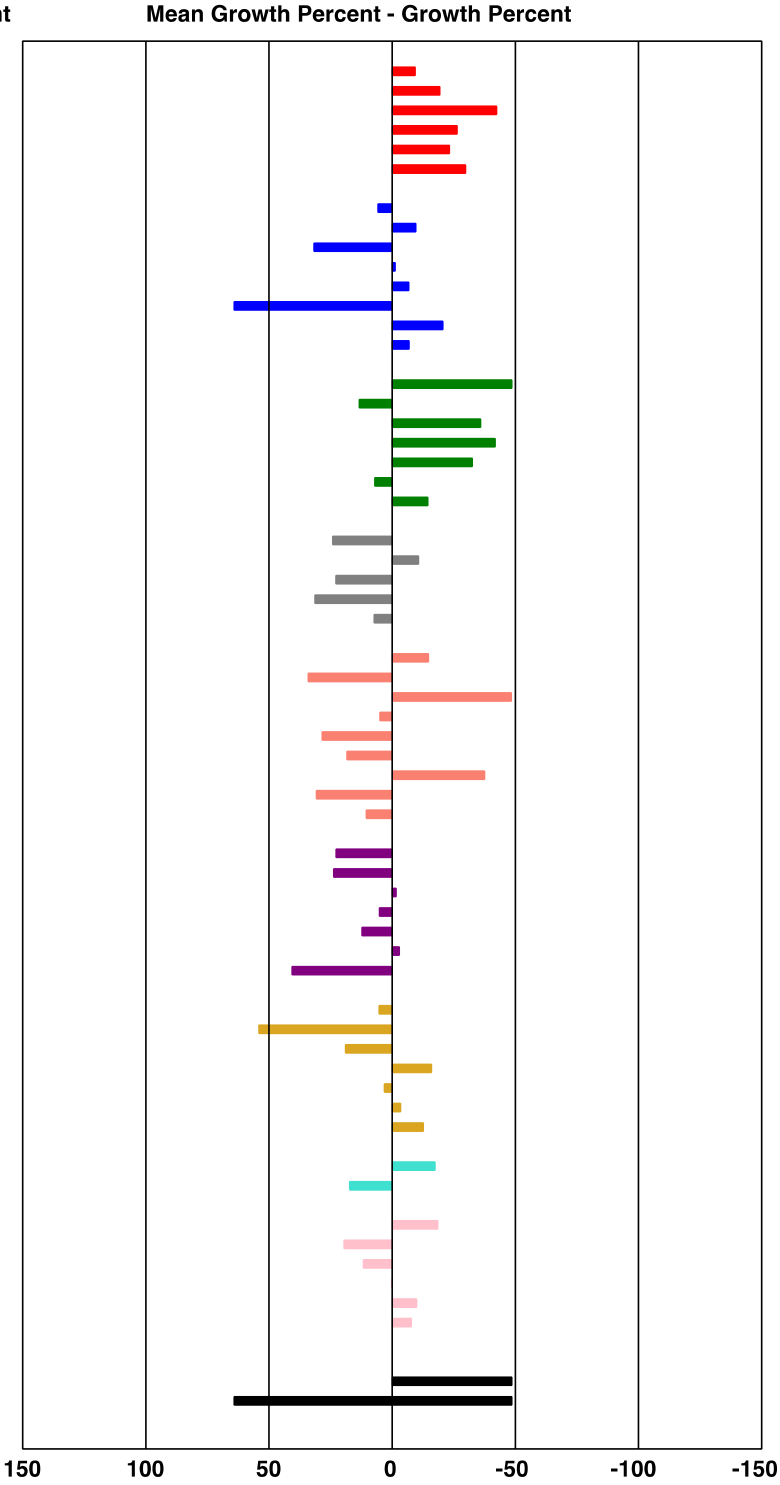

Figure S50: one-dose graph of compound **8** (NSC 846169)

Developmental Therapeutics Program One Dose Mean Graph	NSC: D-846169 / 1	Conc: 1.00E-5 Molar	Test Date: Jul 10, 2023
	Experiment ID: 2307OS44		Report Date: Aug 11, 2023

Panel/Cell Line	Growth Percent
Leukemia	
CCRF-CEM	75.50
HL-60(TB)	61.78
K-562	37.63
MOLT-4	48.98
RPMI-8226	49.67
SR	58.19
Non-Small Cell Lung Cancer	
A549/ATCC	82.01
EKVX	54.21
HOP-62	109.60
NCI-H226	83.95
NCI-H23	75.66
NCI-H322M	131.54
NCI-H460	75.42
NCI-H522	69.14
Colon Cancer	
COLO 205	71.59
HCC-2998	104.76
HCT-116	50.69
HCT-15	61.89
HT29	63.88
KM12	85.37
SW-620	87.21
CNS Cancer	
SF-268	90.95
SF-295	83.10
SF-539	82.84
SNB-75	99.81
U251	81.66
Melanoma	
LOX IMVI	80.00
MALME-3M	105.72
M14	76.14
MDA-MB-435	73.45
SK-MEL-2	89.85
SK-MEL-28	87.66
SK-MEL-5	24.72
UACC-257	87.77
UACC-62	70.29
Ovarian Cancer	
IGROV1	94.00
OVCAR-3	90.85
OVCAR-4	60.91
OVCAR-5	98.44
OVCAR-8	83.23
NCI/ADR-RES	75.27
SK-OV-3	110.54
Renal Cancer	
786-0	84.05
A498	110.38
ACHN	83.43
CAKI-1	59.61
RXF 393	76.29
SN12C	80.53
UO-31	62.50
Prostate Cancer	
PC-3	66.10
DU-145	80.84
Breast Cancer	
MCF7	53.12
MDA-MB-231/ATCC	86.68
HS 578T	74.18
BT-549	80.04
T-47D	61.53
MDA-MB-468	67.71
Mean	77.42
Delta	52.70
Range	106.82

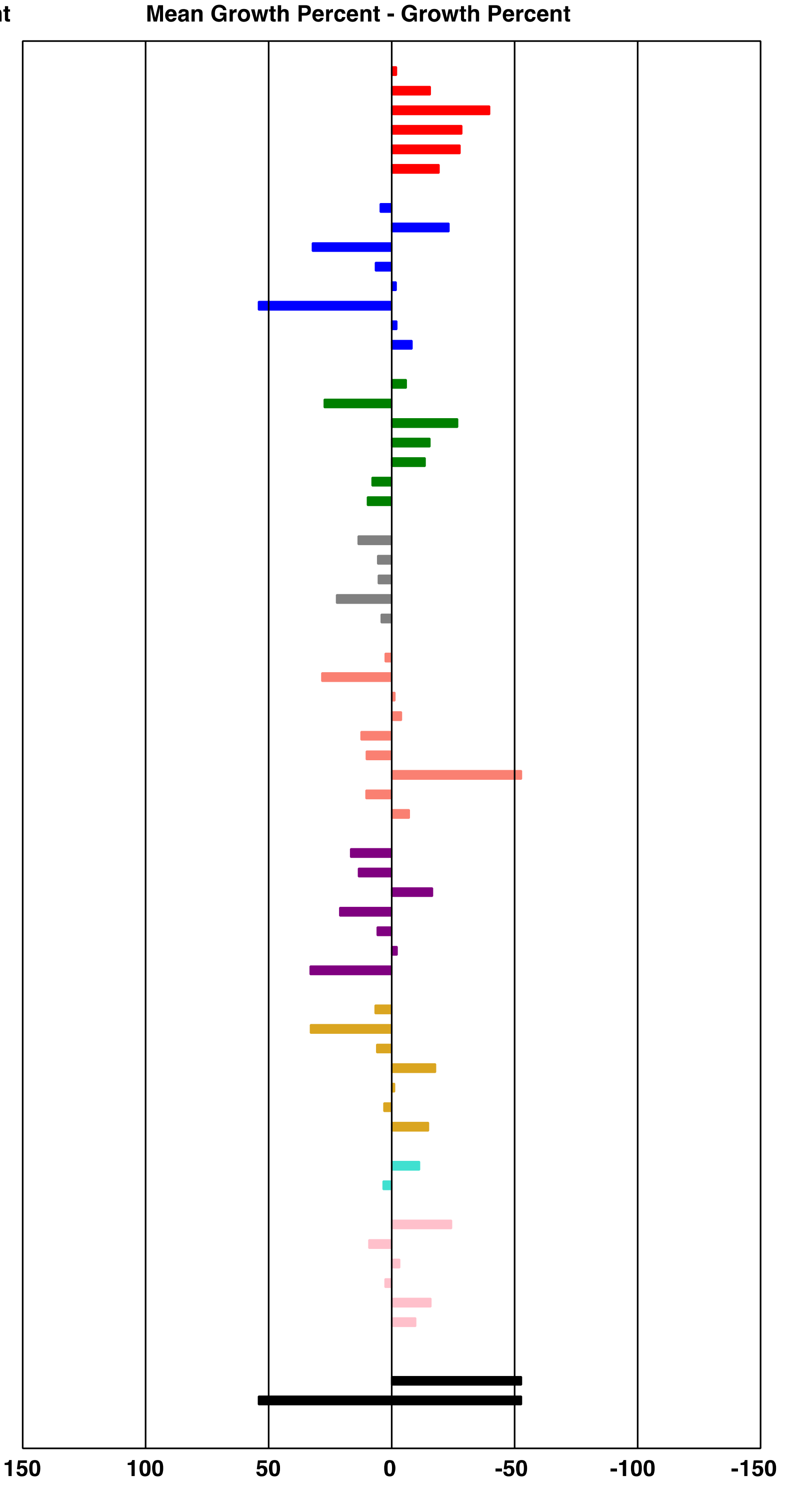

Figure S51: one-dose graph of compound **9** (NSC 846170)

Developmental Therapeutics Program One Dose Mean Graph	NSC: D-846170 / 1	Conc: 1.00E-5 Molar	Test Date: Jul 10, 2023
	Experiment ID: 2307OS44		Report Date: Aug 11, 2023

Panel/Cell Line	Growth Percent
Leukemia	
CCRF-CEM	94.47
HL-60(TB)	64.48
K-562	58.85
MOLT-4	56.72
RPMI-8226	71.90
SR	71.27
Non-Small Cell Lung Cancer	
A549/ATCC	87.79
EKVX	83.95
HOP-62	117.33
NCI-H226	72.93
NCI-H23	78.44
NCI-H322M	138.92
NCI-H460	82.42
NCI-H522	73.10
Colon Cancer	
COLO 205	89.29
HCC-2998	113.26
HCT-116	61.72
HCT-15	73.40
HT29	66.47
KM12	94.75
SW-620	85.87
CNS Cancer	
SF-268	96.82
SF-295	92.80
SF-539	96.64
SNB-75	97.79
U251	99.60
Melanoma	
LOX IMVI	84.84
MALME-3M	106.20
M14	86.45
MDA-MB-435	88.18
SK-MEL-2	90.46
SK-MEL-28	102.88
SK-MEL-5	71.79
UACC-257	102.81
UACC-62	77.77
Ovarian Cancer	
IGROV1	103.57
OVCAR-3	94.00
OVCAR-4	78.54
OVCAR-5	98.64
OVCAR-8	91.36
NCI/ADR-RES	81.07
SK-OV-3	97.83
Renal Cancer	
786-0	100.92
A498	116.86
ACHN	93.66
CAKI-1	73.27
RXF 393	102.96
SN12C	89.12
UO-31	70.87
Prostate Cancer	
PC-3	78.72
DU-145	91.23
Breast Cancer	
MCF7	71.58
MDA-MB-231/ATCC	98.55
HS 578T	89.05
BT-549	116.85
T-47D	92.89
MDA-MB-468	98.20
Mean	88.81
Delta	32.09
Range	82.20

Mean Growth Percent - Growth Percent

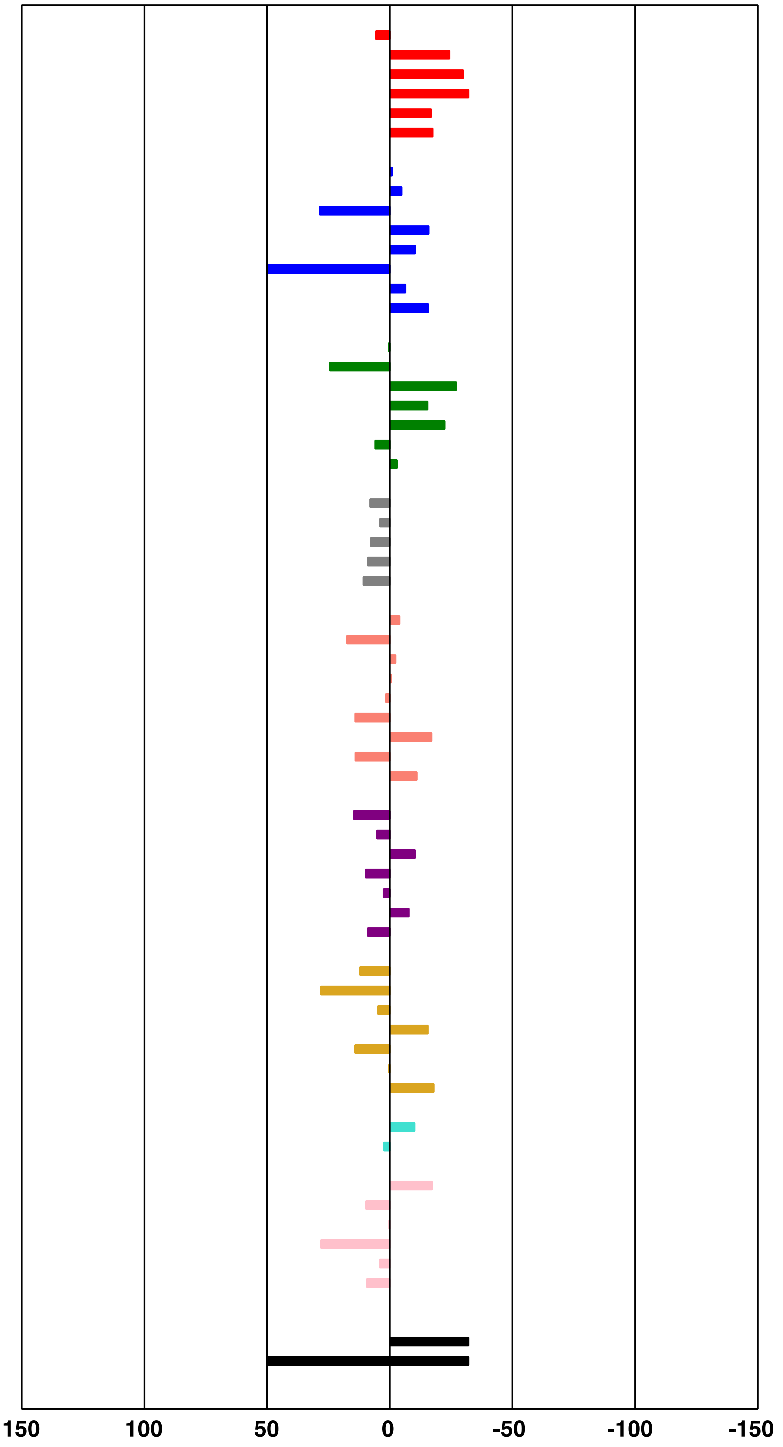

Figure S52: one-dose graph of compound **10** (NSC 846171)

Developmental Therapeutics Program One Dose Mean Graph	NSC: D-846171 / 1	Conc: 1.00E-5 Molar	Test Date: Jul 10, 2023
	Experiment ID: 2307OS44		Report Date: Aug 11, 2023

Panel/Cell Line	Growth Percent
Leukemia	
CCRF-CEM	38.83
HL-60(TB)	-30.92
K-562	0.57
MOLT-4	14.83
RPMI-8226	16.74
SR	-19.75
Non-Small Cell Lung Cancer	
A549/ATCC	44.59
EKVX	25.82
HOP-62	69.52
NCI-H226	24.85
NCI-H23	53.70
NCI-H322M	94.91
NCI-H460	9.27
NCI-H522	28.58
Colon Cancer	
COLO 205	-58.03
HCC-2998	-9.32
HCT-116	4.33
HCT-15	8.76
HT29	-2.47
KM12	26.28
SW-620	19.86
CNS Cancer	
SF-268	62.85
SF-295	9.16
SF-539	49.75
SNB-75	36.84
U251	42.87
Melanoma	
LOX IMVI	4.75
MALME-3M	-72.64
M14	-59.25
MDA-MB-435	24.89
SK-MEL-2	25.05
SK-MEL-28	-29.31
SK-MEL-5	-72.58
UACC-257	-8.50
UACC-62	52.94
Ovarian Cancer	
IGROV1	52.11
OVCAR-3	56.92
OVCAR-4	35.72
OVCAR-5	55.95
OVCAR-8	59.68
NCI/ADR-RES	49.37
SK-OV-3	70.56
Renal Cancer	
786-0	31.28
A498	92.60
ACHN	46.06
CAKI-1	10.58
RXF 393	-8.69
SN12C	48.89
UO-31	41.47
Prostate Cancer	
PC-3	36.50
DU-145	40.54
Breast Cancer	
MCF7	19.77
MDA-MB-231/ATCC	48.90
HS 578T	49.15
BT-549	48.79
T-47D	34.17
MDA-MB-468	-13.75
Mean	23.41
Delta	96.05
Range	167.55

Mean Growth Percent - Growth Percent

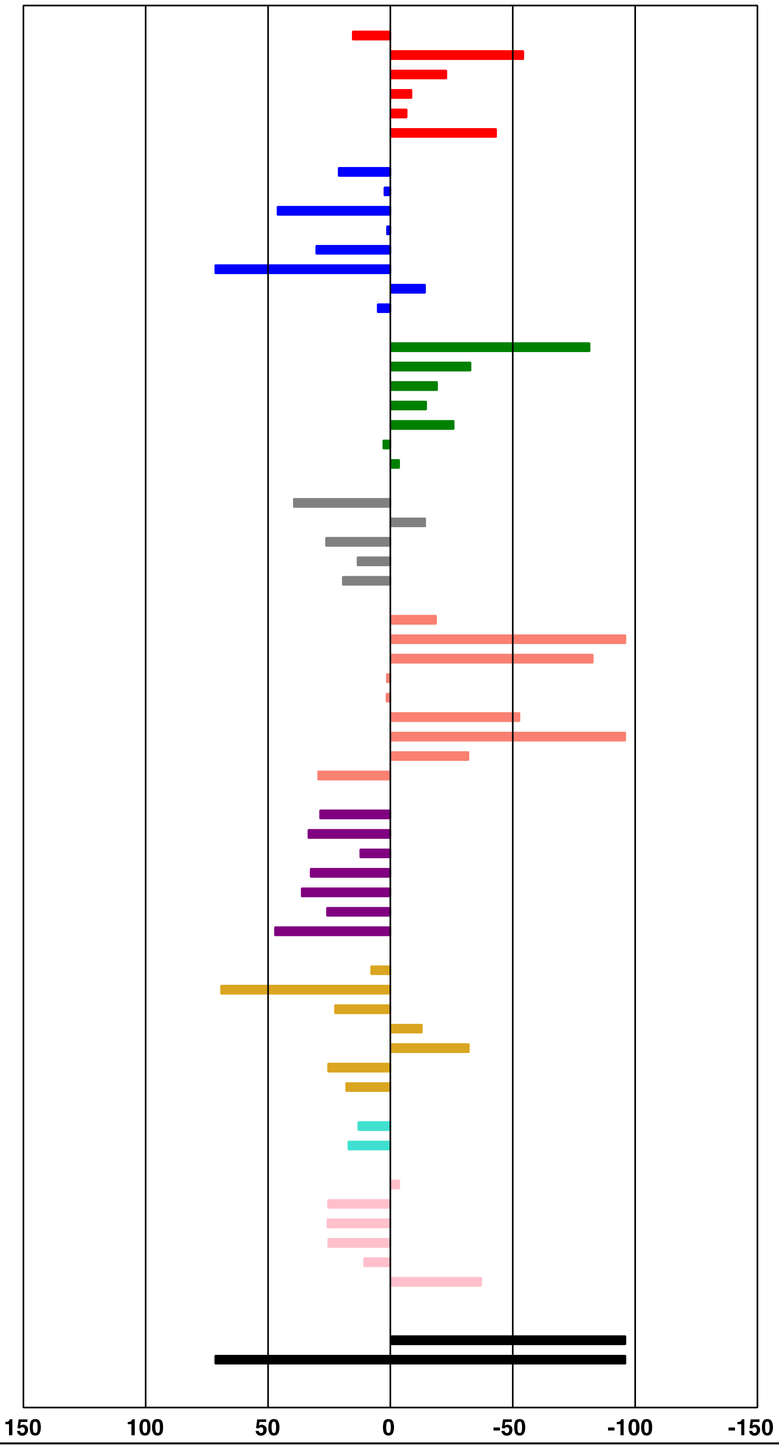

Figure S53: one-dose graph of compound **11** (NSC 846172)

Developmental Therapeutics Program One Dose Mean Graph	NSC: D-846172 / 1	Conc: 1.00E-5 Molar	Test Date: Jul 10, 2023
	Experiment ID: 2307OS44		Report Date: Aug 11, 2023

Panel/Cell Line	Growth Percent
Leukemia	
CCRF-CEM	23.86
HL-60(TB)	-26.72
K-562	-15.60
MOLT-4	0.26
RPMI-8226	7.96
SR	-11.99
Non-Small Cell Lung Cancer	
A549/ATCC	50.78
EKVX	29.17
HOP-62	65.23
NCI-H226	35.13
NCI-H23	54.56
NCI-H322M	77.60
NCI-H460	1.42
NCI-H522	17.76
Colon Cancer	
COLO 205	-58.61
HCC-2998	-49.20
HCT-116	3.16
HCT-15	15.22
HT29	8.97
KM12	22.00
SW-620	13.33
CNS Cancer	
SF-268	68.20
SF-295	-38.81
SF-539	29.04
SNB-75	18.25
U251	27.27
Melanoma	
LOX IMVI	0.27
MALME-3M	-88.18
M14	-56.04
MDA-MB-435	-20.99
SK-MEL-2	-78.42
SK-MEL-28	-73.62
SK-MEL-5	-94.54
UACC-257	-62.94
UACC-62	22.97
Ovarian Cancer	
IGROV1	44.03
OVCAR-3	71.86
OVCAR-4	39.72
OVCAR-5	58.07
OVCAR-8	58.82
NCI/ADR-RES	49.92
SK-OV-3	80.67
Renal Cancer	
786-0	27.12
A498	85.31
ACHN	45.18
CAKI-1	8.47
RXF 393	-9.45
SN12C	46.63
UO-31	36.88
Prostate Cancer	
PC-3	33.42
DU-145	44.91
Breast Cancer	
MCF7	9.66
MDA-MB-231/ATCC	38.29
HS 578T	44.38
BT-549	45.96
T-47D	33.93
MDA-MB-468	-52.92
Mean	13.29
Delta	107.83
Range	179.85

Mean Growth Percent - Growth Percent

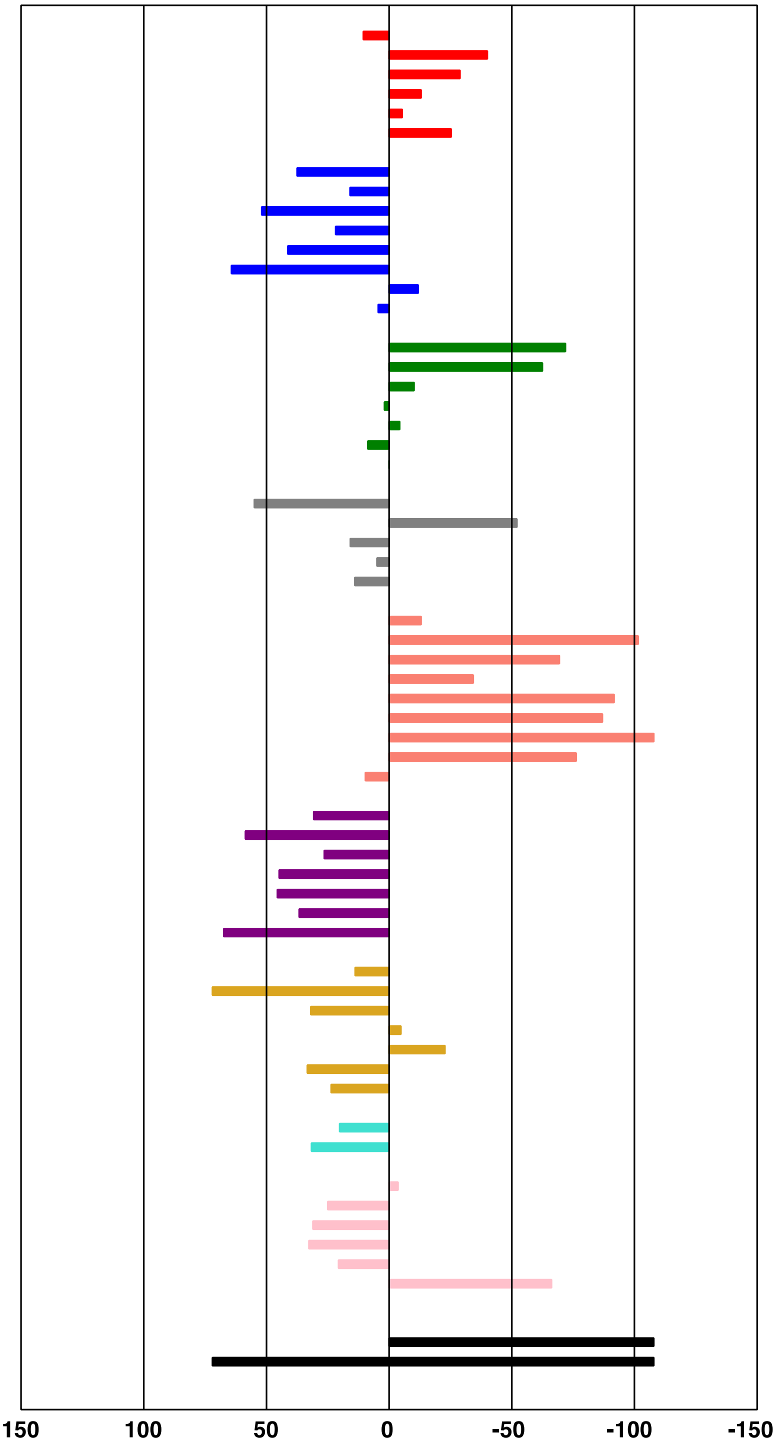

Figure S54: one-dose graph of compound **12** (NSC 846153)

Developmental Therapeutics Program One Dose Mean Graph	**NSC:** D-846153 / 1	**Conc:** 1.00E-5 Molar	**Test Date:** Jul 10, 2023
	**Experiment ID:** 2307OS44		**Report Date:** Sep 01, 2025

Panel/Cell Line	Growth Percent
Leukemia	
CCRF-CEM	54.84
HL-60(TB)	32.78
K-562	29.13
MOLT-4	60.22
RPMI-8226	43.37
SR	21.34
Non-Small Cell Lung Cancer	
A549/ATCC	74.17
EKVX	27.70
HOP-62	111.91
NCI-H226	55.54
NCI-H23	63.52
NCI-H322M	116.48
NCI-H460	75.09
NCI-H522	88.07
Colon Cancer	
COLO 205	115.79
HCC-2998	54.94
HCT-116	34.22
HCT-15	40.74
HT29	34.53
KM12	66.73
SW-620	75.19
CNS Cancer	
SF-268	77.23
SF-295	56.04
SF-539	75.60
SNB-75	66.97
U251	72.40
Melanoma	
LOX IMVI	61.11
MALME-3M	76.51
M14	42.96
MDA-MB-435	62.75
SK-MEL-2	84.70
SK-MEL-28	83.81
SK-MEL-5	-7.83
UACC-257	67.26
UACC-62	66.38
Ovarian Cancer	
IGROV1	104.38
OVCAR-3	71.95
OVCAR-4	43.60
OVCAR-5	84.55
OVCAR-8	74.37
NCI/ADR-RES	68.44
SK-OV-3	117.16
Renal Cancer	
786-0	64.74
A498	124.66
ACHN	84.80
CAKI-1	47.80
RXF 393	64.86
SN12C	69.04
UO-31	47.84
Prostate Cancer	
PC-3	43.31
DU-145	81.01
Breast Cancer	
MCF7	39.12
MDA-MB-231/ATCC	83.05
HS 578T	81.56
BT-549	48.90
T-47D	113.72
MDA-MB-468	24.01
Mean	65.70
Delta	73.53
Range	132.49

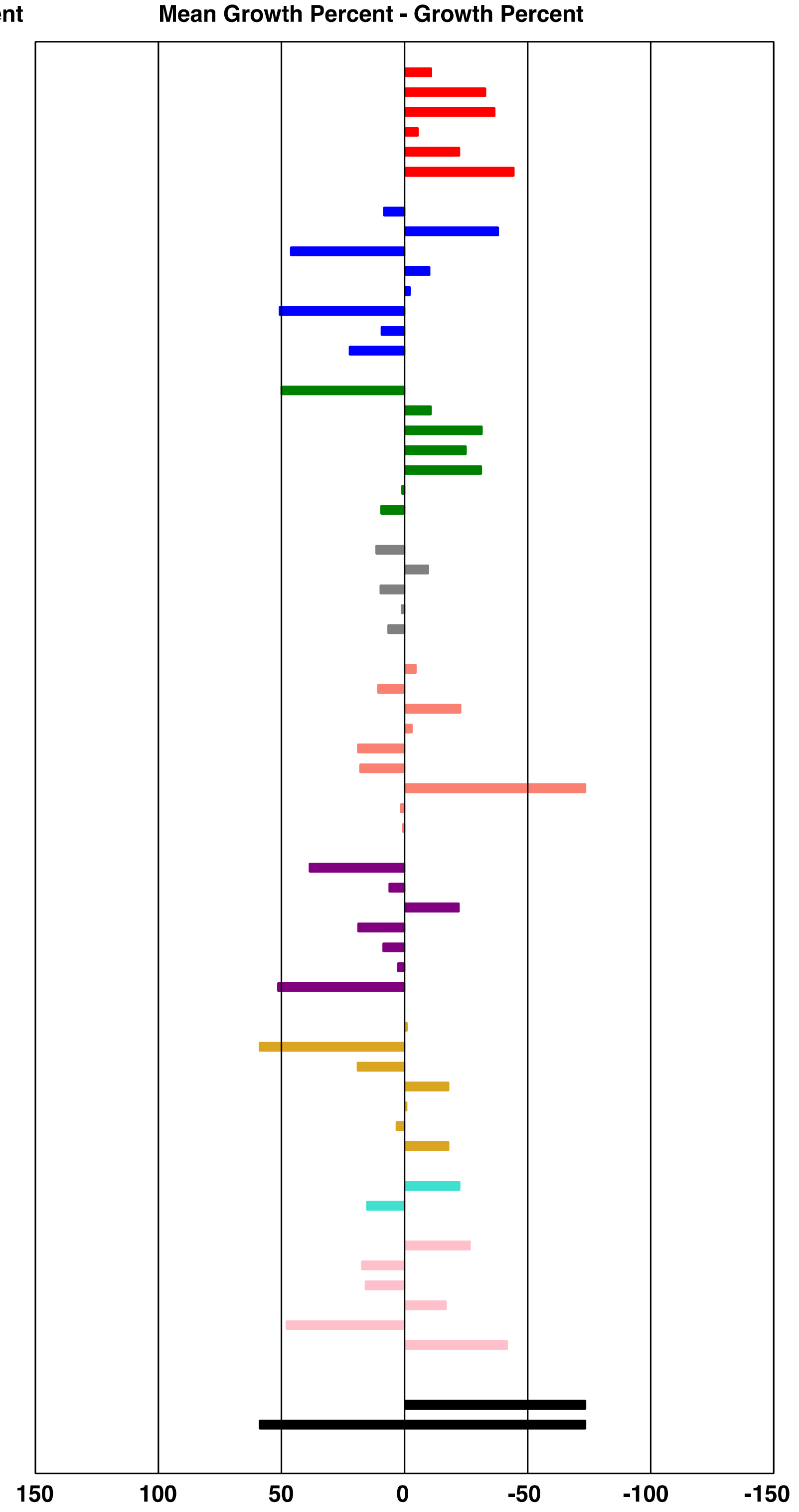

Figure S55: one-dose graph of compound **13** (NSC 846154)

Developmental Therapeutics Program One Dose Mean Graph	NSC: D-846154 / 1	Conc: 1.00E-5 Molar	Test Date: Jul 10, 2023
	Experiment ID: 2307OS44		Report Date: Sep 01, 2025

Panel/Cell Line	Growth Percent
**Leukemia**	
CCRF-CEM	54.37
HL-60(TB)	30.44
K-562	29.12
MOLT-4	38.34
RPMI-8226	32.62
SR	29.27
**Non-Small Cell Lung Cancer**	
A549/ATCC	77.23
EKVX	50.26
HOP-62	108.68
NCI-H226	55.84
NCI-H23	66.39
NCI-H322M	109.47
NCI-H460	67.68
NCI-H522	80.41
**Colon Cancer**	
COLO 205	119.79
HCC-2998	107.87
HCT-116	52.93
HCT-15	41.41
HT29	30.79
KM12	66.61
SW-620	78.22
**CNS Cancer**	
SF-268	90.87
SF-295	66.56
SF-539	85.56
SNB-75	69.96
U251	99.19
**Melanoma**	
LOX IMVI	67.06
MALME-3M	94.80
M14	70.48
MDA-MB-435	56.30
SK-MEL-2	83.59
SK-MEL-28	89.93
SK-MEL-5	-21.64
UACC-257	72.29
UACC-62	73.95
**Ovarian Cancer**	
IGROV1	113.04
OVCAR-3	89.38
OVCAR-4	63.10
OVCAR-5	98.18
OVCAR-8	82.64
NCI/ADR-RES	75.03
SK-OV-3	110.12
**Renal Cancer**	
786-0	97.26
A498	125.54
ACHN	72.56
CAKI-1	53.81
RXF 393	78.57
SN12C	72.86
UO-31	51.55
**Prostate Cancer**	
PC-3	39.61
DU-145	83.30
**Breast Cancer**	
MCF7	43.12
MDA-MB-231/ATCC	76.19
HS 578T	94.88
BT-549	84.15
T-47D	114.18
MDA-MB-468	4.72
Mean	71.06
Delta	92.70
Range	147.18

Mean Growth Percent - Growth Percent

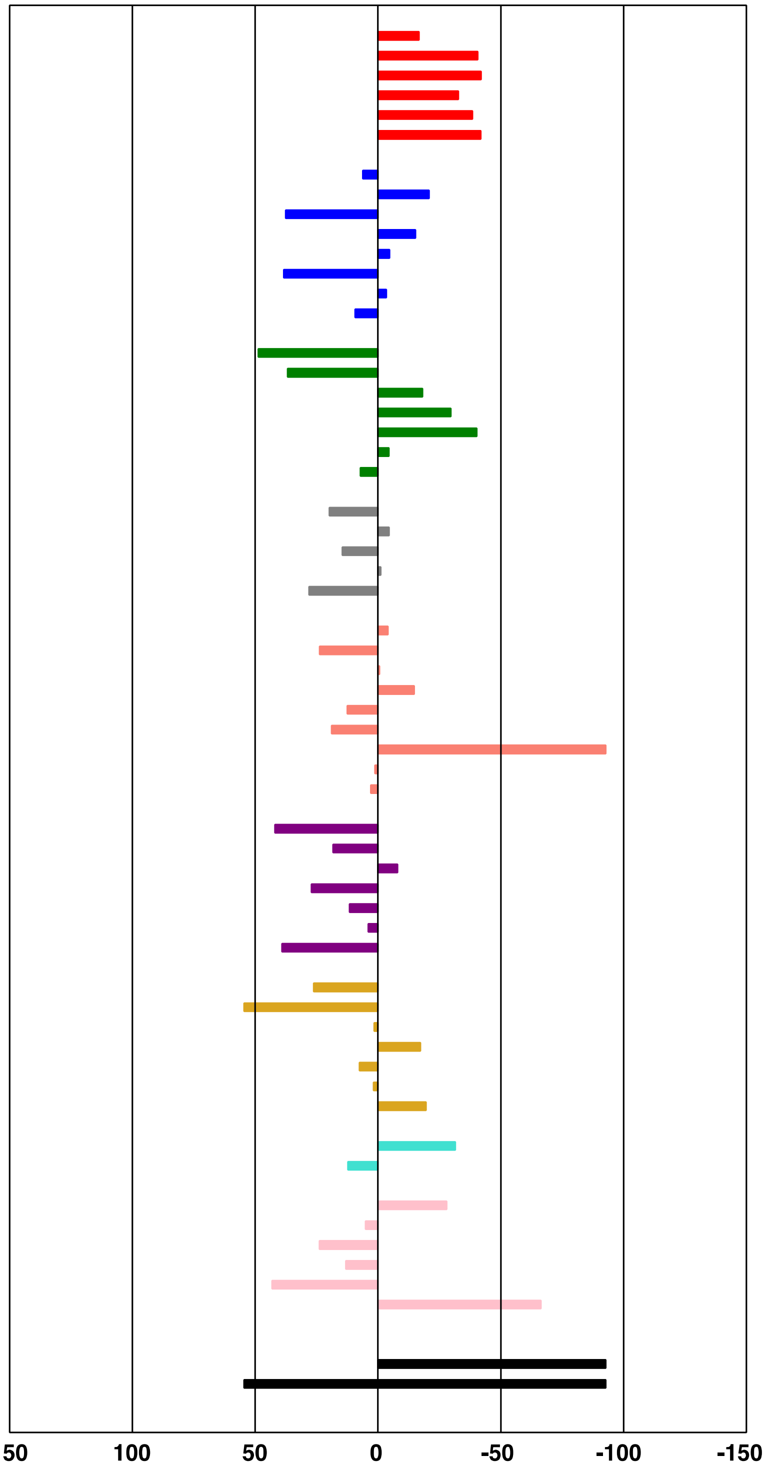

Figure S56: one-dose graph of compound **14** (NSC 846155)

Developmental Therapeutics Program One Dose Mean Graph	NSC: D-846155 / 1	Conc: 1.00E-5 Molar	Test Date: Jul 10, 2023
	Experiment ID: 2307OS44		Report Date: Sep 01, 2025

Panel/Cell Line	Growth Percent
Leukemia	
CCRF-CEM	51.29
HL-60(TB)	28.29
K-562	29.69
MOLT-4	43.70
RPMI-8226	39.15
SR	26.03
Non-Small Cell Lung Cancer	
A549/ATCC	73.66
EKVX	27.35
HOP-62	111.32
NCI-H226	48.23
NCI-H23	53.84
NCI-H322M	105.16
NCI-H460	71.84
NCI-H522	69.30
Colon Cancer	
COLO 205	121.06
HCC-2998	75.45
HCT-116	33.54
HCT-15	53.04
HT29	46.62
KM12	71.16
SW-620	73.51
CNS Cancer	
SF-268	76.43
SF-295	48.51
SF-539	79.68
SNB-75	56.91
U251	69.07
Melanoma	
LOX IMVI	59.11
MALME-3M	75.39
M14	56.12
MDA-MB-435	71.63
SK-MEL-2	82.12
SK-MEL-28	85.11
SK-MEL-5	-3.84
UACC-257	71.14
UACC-62	58.16
Ovarian Cancer	
IGROV1	80.71
OVCAR-3	72.22
OVCAR-4	50.05
OVCAR-5	79.43
OVCAR-8	72.25
NCI/ADR-RES	66.67
SK-OV-3	112.69
Renal Cancer	
786-0	66.54
A498	104.24
ACHN	83.68
CAKI-1	55.28
RXF 393	68.45
SN12C	63.84
UO-31	36.41
Prostate Cancer	
PC-3	35.64
DU-145	82.23
Breast Cancer	
MCF7	43.13
MDA-MB-231/ATCC	76.77
HS 578T	74.74
BT-549	46.98
T-47D	104.03
MDA-MB-468	17.62
Mean	63.73
Delta	67.57
Range	124.90

Mean Growth Percent - Growth Percent

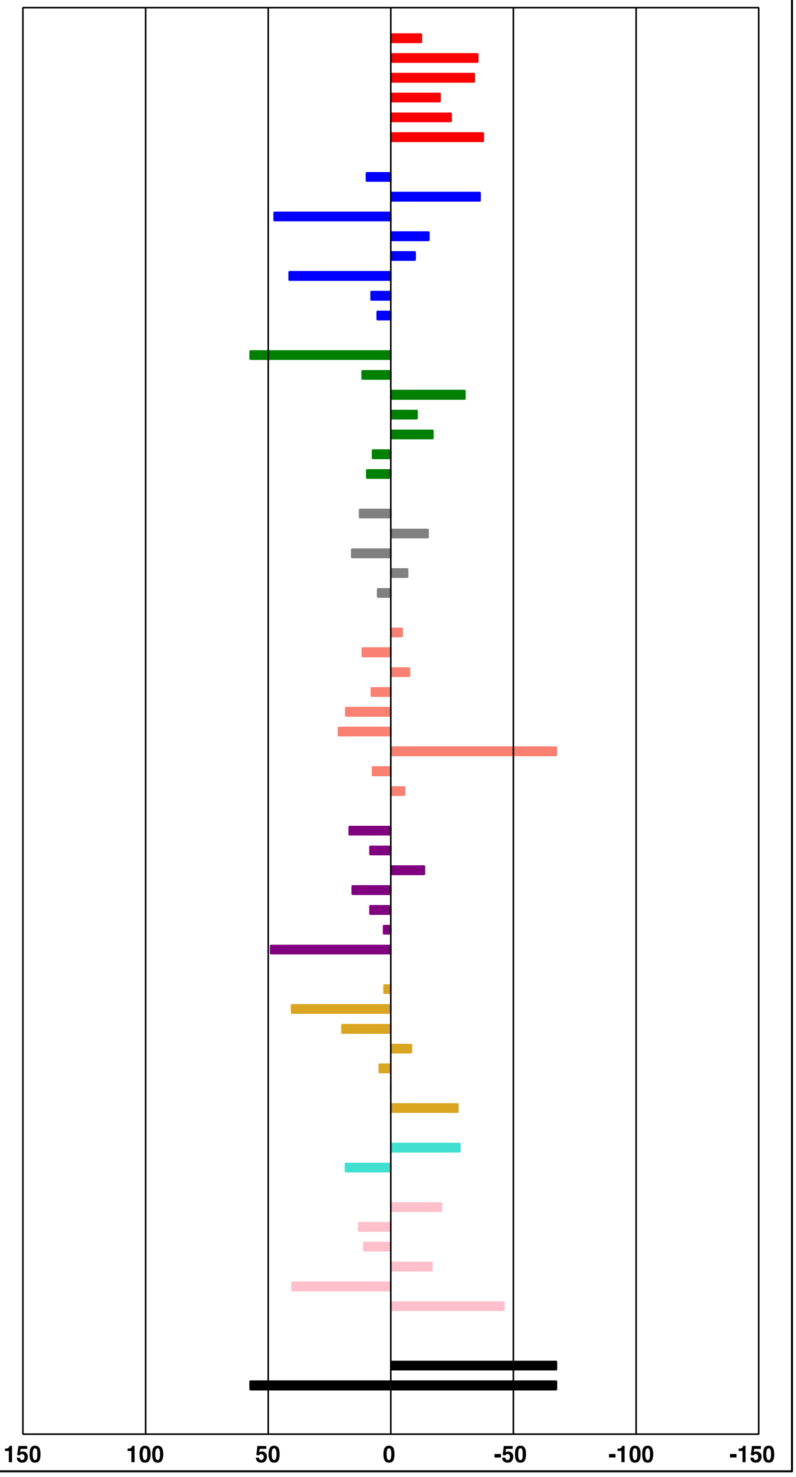

Figure S57: one-dose graph of compound **15** (NSC 846156)

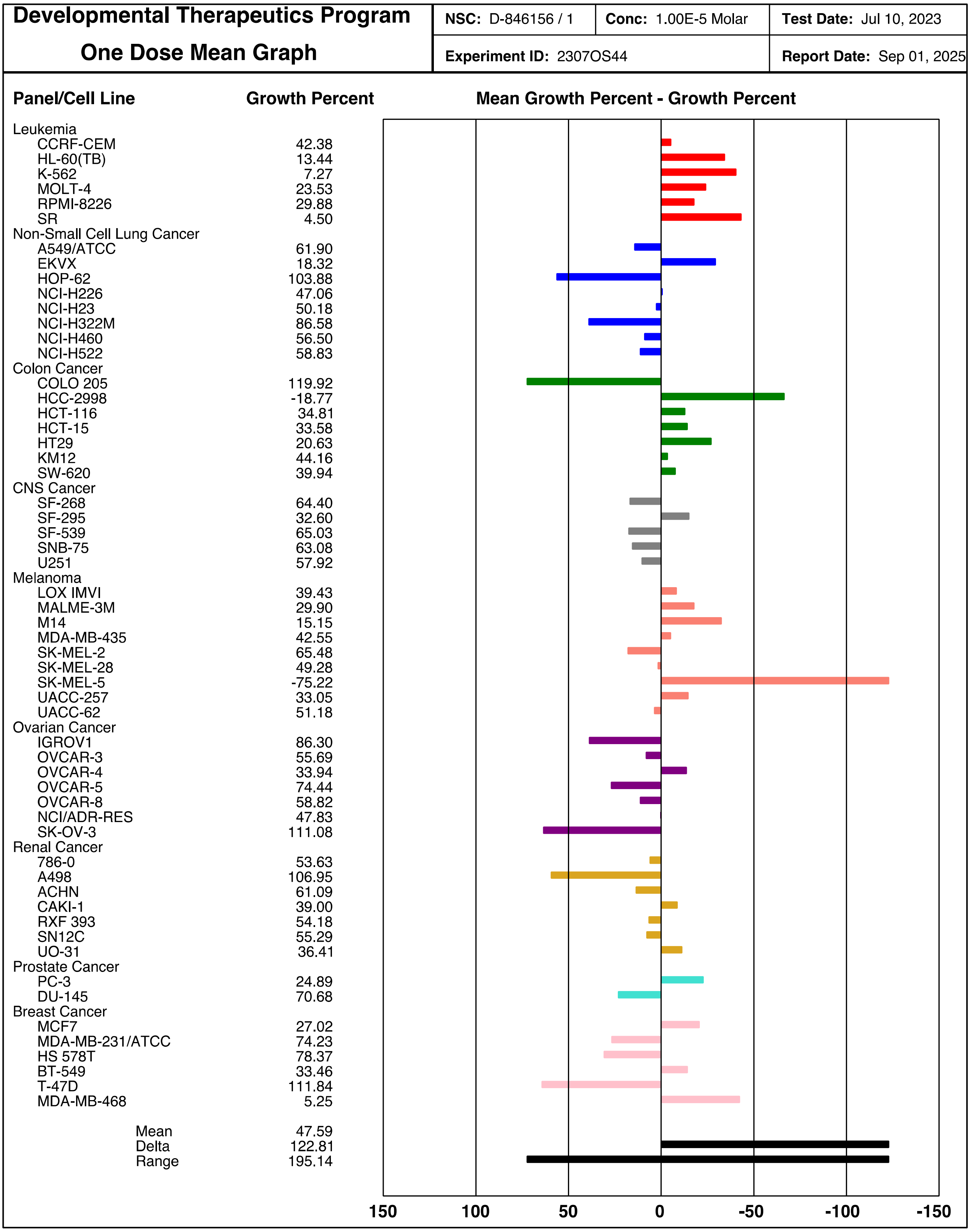

Figure S58: one-dose graph of compound **16** (NSC 846157)

Developmental Therapeutics Program One Dose Mean Graph	NSC: D-846157 / 1	Conc: 1.00E-5 Molar	Test Date: Jul 10, 2023
	Experiment ID: 2307OS44		Report Date: Sep 01, 2025

Panel/Cell Line	Growth Percent
**Leukemia**	
CCRF-CEM	61.13
HL-60(TB)	63.58
K-562	22.41
MOLT-4	48.04
RPMI-8226	39.98
SR	26.38
**Non-Small Cell Lung Cancer**	
A549/ATCC	74.77
EKVX	46.30
HOP-62	112.91
NCI-H226	59.43
NCI-H23	65.81
NCI-H322M	105.38
NCI-H460	82.20
NCI-H522	77.94
**Colon Cancer**	
COLO 205	104.50
HCC-2998	82.29
HCT-116	56.45
HCT-15	61.51
HT29	25.09
KM12	75.83
SW-620	84.73
**CNS Cancer**	
SF-268	78.95
SF-295	62.83
SF-539	74.54
SNB-75	59.97
U251	77.41
**Melanoma**	
LOX IMVI	66.73
MALME-3M	76.37
M14	55.51
MDA-MB-435	79.00
SK-MEL-2	84.65
SK-MEL-28	84.13
SK-MEL-5	11.86
UACC-257	72.17
UACC-62	63.61
**Ovarian Cancer**	
IGROV1	89.64
OVCAR-3	75.93
OVCAR-4	58.48
OVCAR-5	91.92
OVCAR-8	76.31
NCI/ADR-RES	69.81
SK-OV-3	105.12
**Renal Cancer**	
786-0	69.85
A498	110.66
ACHN	83.48
CAKI-1	61.49
RXF 393	74.74
SN12C	69.24
UO-31	48.38
**Prostate Cancer**	
PC-3	52.55
DU-145	86.11
**Breast Cancer**	
MCF7	57.36
MDA-MB-231/ATCC	88.23
HS 578T	90.06
BT-549	65.23
T-47D	104.97
MDA-MB-468	38.07
Mean	70.03
Delta	58.17
Range	101.05

Mean Growth Percent - Growth Percent

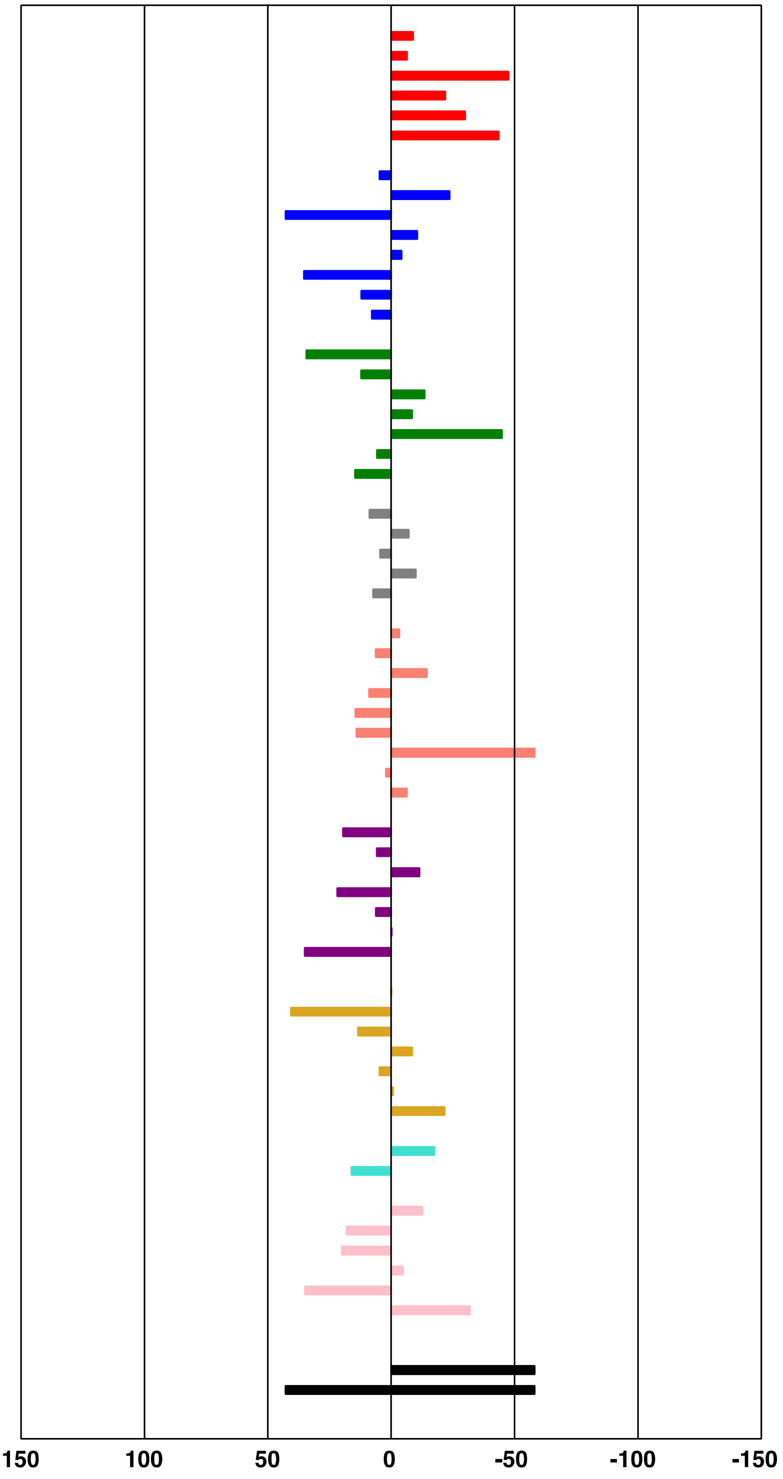

Figure S59: one-dose graph of compound **17** (NSC 846158)

Developmental Therapeutics Program One Dose Mean Graph	NSC: D-846158 / 1	Conc: 1.00E-5 Molar	Test Date: Jul 10, 2023
	Experiment ID: 2307OS44		Report Date: Sep 01, 2025

Panel/Cell Line	Growth Percent
Leukemia	
CCRF-CEM	38.42
HL-60(TB)	-8.21
K-562	0.05
MOLT-4	4.47
RPMI-8226	22.99
SR	-5.19
Non-Small Cell Lung Cancer	
A549/ATCC	54.39
EKVX	12.47
HOP-62	62.86
NCI-H226	39.66
NCI-H23	47.54
NCI-H322M	97.01
NCI-H460	30.03
NCI-H522	34.35
Colon Cancer	
COLO 205	-47.24
HCC-2998	-35.63
HCT-116	11.08
HCT-15	19.00
HT29	18.54
KM12	47.43
SW-620	45.94
CNS Cancer	
SF-268	58.27
SF-295	24.39
SF-539	53.45
SNB-75	42.04
U251	45.00
Melanoma	
LOX IMVI	25.65
MALME-3M	9.67
M14	-7.74
MDA-MB-435	30.69
SK-MEL-2	61.72
SK-MEL-28	28.52
SK-MEL-5	-88.37
UACC-257	28.00
UACC-62	43.26
Ovarian Cancer	
IGROV1	71.57
OVCAR-3	51.40
OVCAR-4	23.15
OVCAR-5	62.35
OVCAR-8	58.36
NCI/ADR-RES	35.90
SK-OV-3	47.34
Renal Cancer	
786-0	40.88
A498	83.29
ACHN	54.23
CAKI-1	28.36
RXF 393	37.93
SN12C	39.30
UO-31	30.72
Prostate Cancer	
PC-3	24.58
DU-145	57.44
Breast Cancer	
MCF7	17.79
MDA-MB-231/ATCC	62.47
HS 578T	60.74
BT-549	19.36
T-47D	22.41
MDA-MB-468	-3.78
Mean	31.06
Delta	119.43
Range	185.38

Mean Growth Percent - Growth Percent

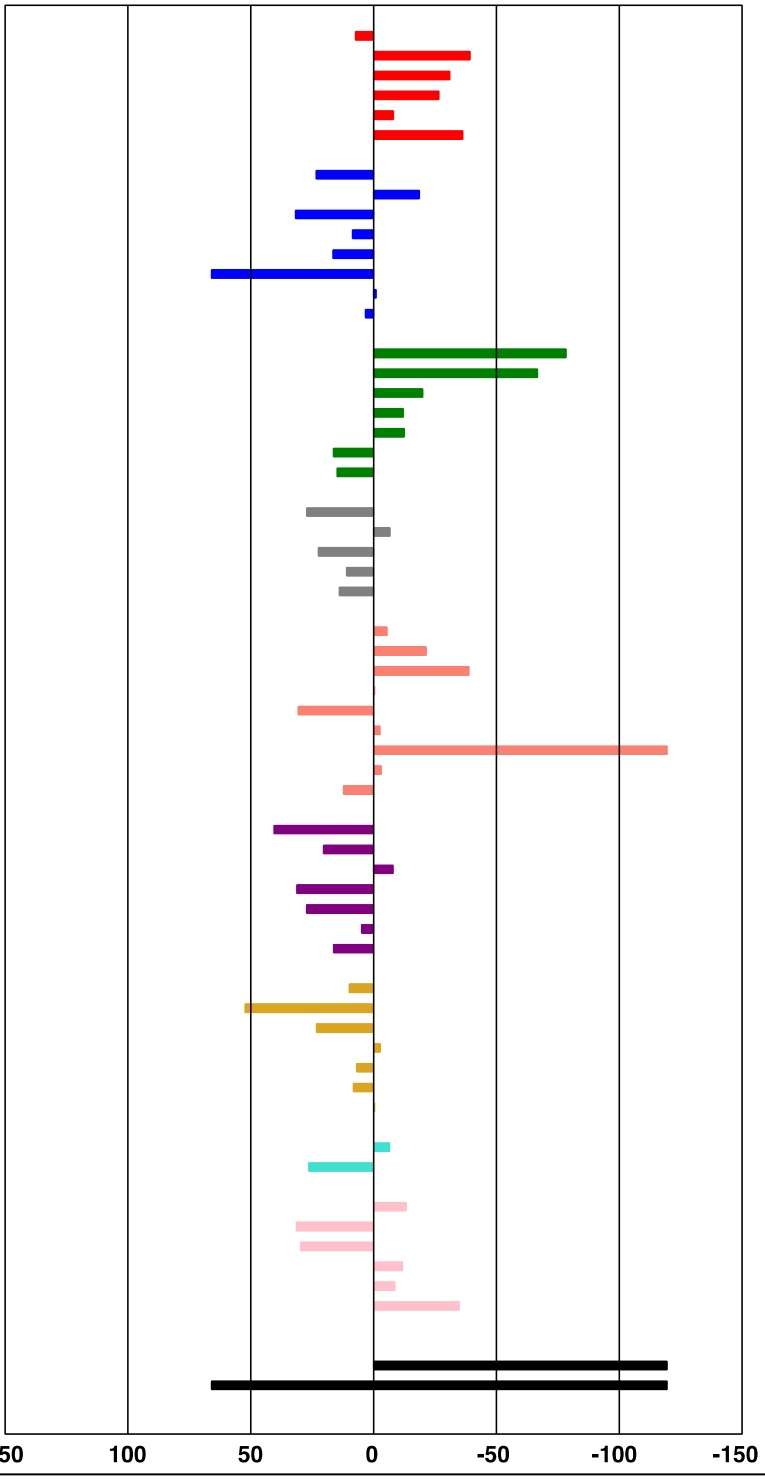

Figure S60: one-dose graph of compound **18** (NSC 846159)

Developmental Therapeutics Program One Dose Mean Graph	**NSC:** D-846159 / 1	**Conc:** 1.00E-5 Molar	**Test Date:** Jul 10, 2023
	**Experiment ID:** 2307OS44		**Report Date:** Sep 01, 2025

Panel/Cell Line	Growth Percent
Leukemia	
CCRF-CEM	48.32
HL-60(TB)	25.66
K-562	24.74
MOLT-4	18.20
RPMI-8226	30.87
SR	25.80
Non-Small Cell Lung Cancer	
A549/ATCC	50.87
EKVX	7.18
HOP-62	68.60
NCI-H226	47.07
NCI-H23	48.85
NCI-H322M	95.70
NCI-H460	50.99
NCI-H522	50.38
Colon Cancer	
COLO 205	17.55
HCC-2998	69.59
HCT-116	29.90
HCT-15	38.73
HT29	35.82
KM12	64.70
SW-620	70.82
CNS Cancer	
SF-268	58.47
SF-295	31.59
SF-539	60.15
SNB-75	35.74
U251	54.76
Melanoma	
LOX IMVI	50.51
MALME-3M	69.58
M14	41.75
MDA-MB-435	35.46
SK-MEL-2	65.22
SK-MEL-28	56.95
SK-MEL-5	-67.02
UACC-257	46.11
UACC-62	40.51
Ovarian Cancer	
IGROV1	116.78
OVCAR-3	53.73
OVCAR-4	22.63
OVCAR-5	82.38
OVCAR-8	60.69
NCI/ADR-RES	50.32
SK-OV-3	47.52
Renal Cancer	
786-0	53.31
A498	72.25
ACHN	59.32
CAKI-1	37.29
RXF 393	59.32
SN12C	50.80
UO-31	32.95
Prostate Cancer	
PC-3	39.59
DU-145	61.75
Breast Cancer	
MCF7	21.50
MDA-MB-231/ATCC	71.71
HS 578T	62.12
BT-549	21.65
T-47D	28.17
MDA-MB-468	0.28
Mean	45.72
Delta	112.74
Range	183.80

Mean Growth Percent - Growth Percent

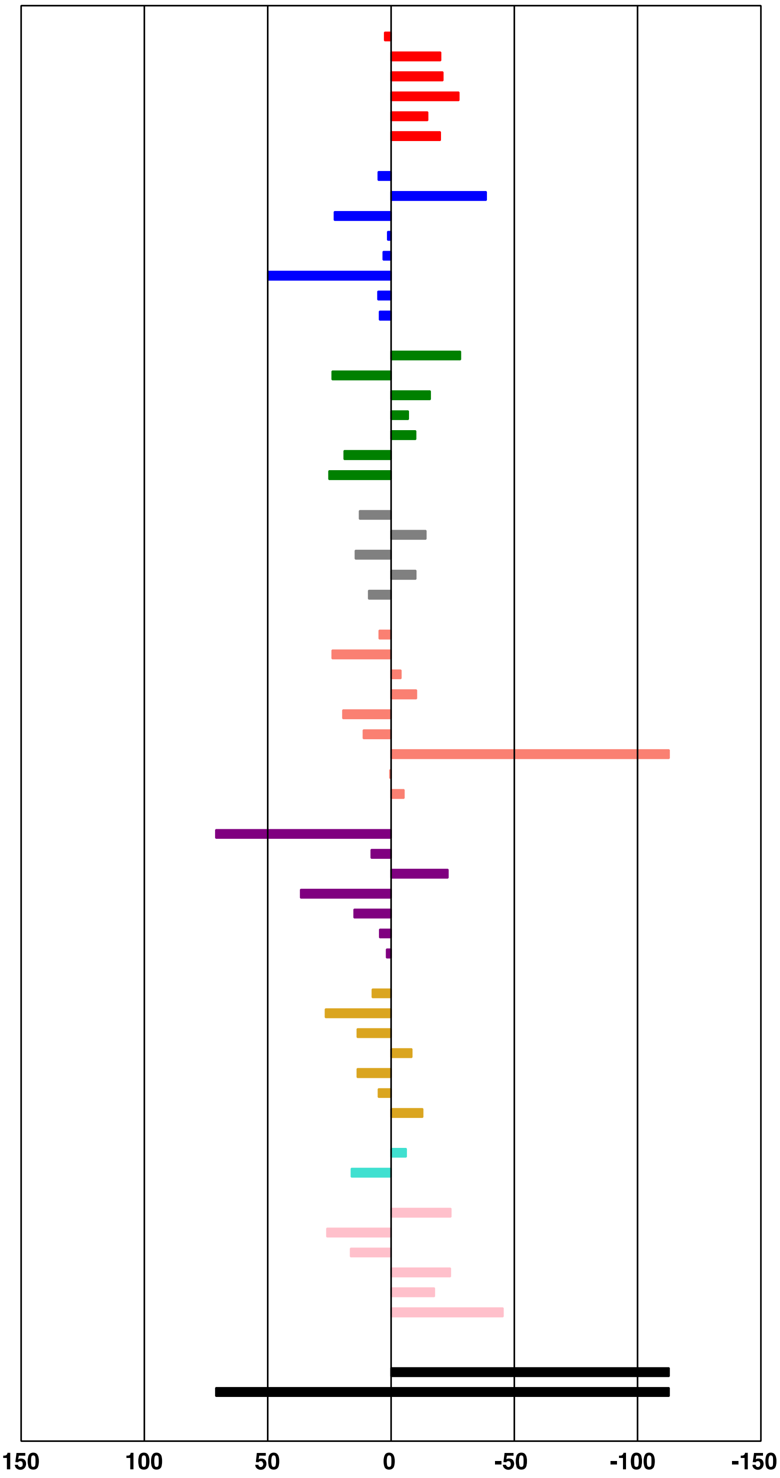

Figure S61: one-dose graph of compound **19** (NSC 846161)

Developmental Therapeutics Program One Dose Mean Graph	NSC: D-846161 / 1	Conc: 1.00E-5 Molar	Test Date: Jul 10, 2023
	Experiment ID: 2307OS44		Report Date: Sep 01, 2025

Panel/Cell Line	Growth Percent
Leukemia	
CCRF-CEM	55.88
HL-60(TB)	18.54
K-562	31.38
MOLT-4	23.30
RPMI-8226	32.16
SR	22.68
Non-Small Cell Lung Cancer	
A549/ATCC	65.09
EKVX	38.28
HOP-62	82.96
NCI-H226	62.77
NCI-H23	56.97
NCI-H322M	103.76
NCI-H460	55.60
NCI-H522	61.87
Colon Cancer	
COLO 205	41.97
HCC-2998	81.41
HCT-116	48.00
HCT-15	42.09
HT29	42.08
KM12	65.72
SW-620	78.56
CNS Cancer	
SF-268	70.38
SF-295	61.21
SF-539	74.08
SNB-75	44.25
U251	78.00
Melanoma	
LOX IMVI	55.55
MALME-3M	98.31
M14	54.06
MDA-MB-435	62.20
SK-MEL-2	55.17
SK-MEL-28	79.83
SK-MEL-5	-24.28
UACC-257	71.33
UACC-62	66.04
Ovarian Cancer	
IGROV1	87.74
OVCAR-3	77.69
OVCAR-4	54.22
OVCAR-5	89.67
OVCAR-8	77.36
NCI/ADR-RES	61.43
SK-OV-3	65.27
Renal Cancer	
786-0	80.27
A498	96.59
ACHN	64.91
CAKI-1	44.87
RXF 393	74.74
SN12C	64.11
UO-31	42.56
Prostate Cancer	
PC-3	47.31
DU-145	69.12
Breast Cancer	
MCF7	30.77
MDA-MB-231/ATCC	65.50
HS 578T	74.95
BT-549	69.67
T-47D	43.21
MDA-MB-468	5.84
Mean	58.68
Delta	82.96
Range	128.04

Mean Growth Percent - Growth Percent

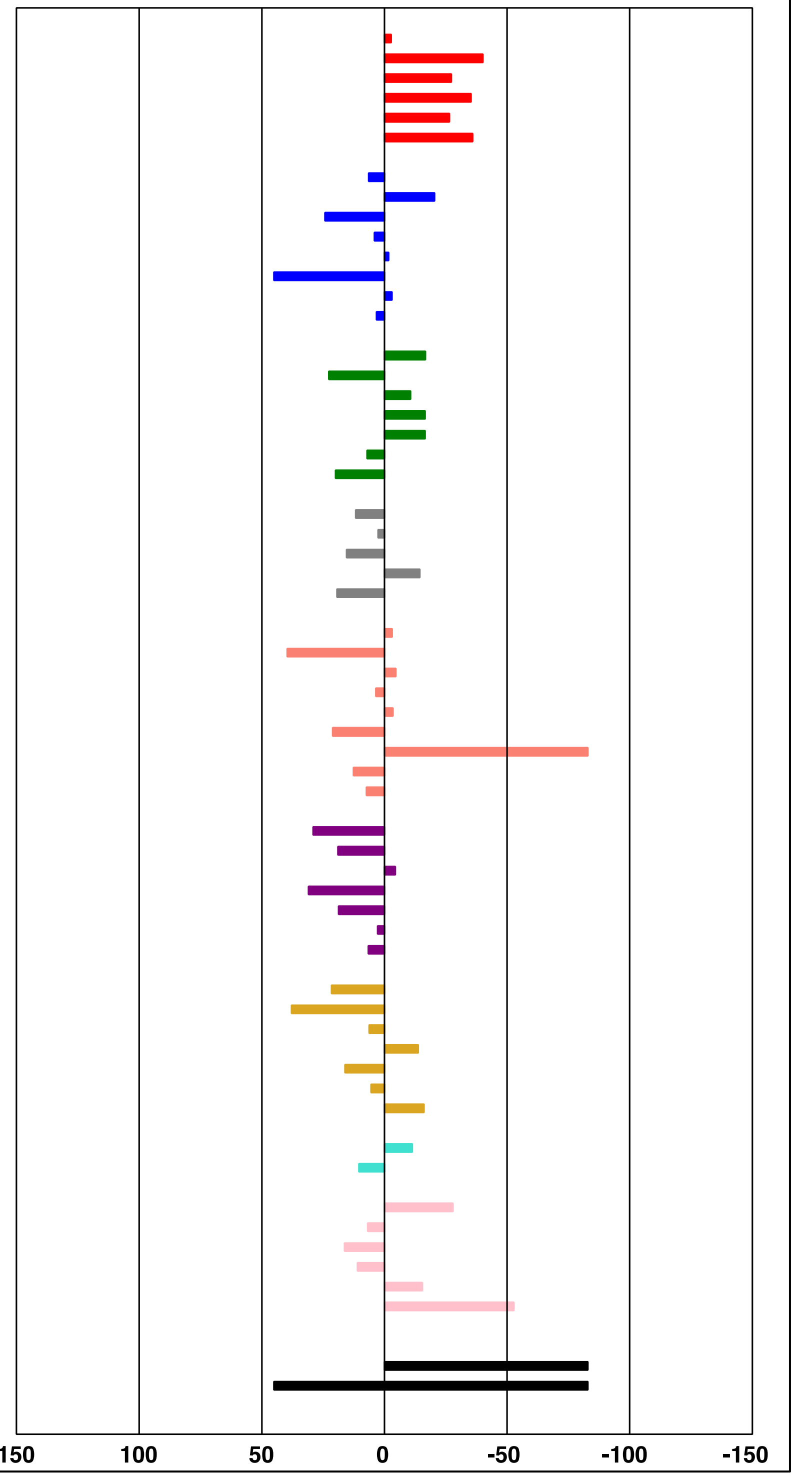

Figure S62: one-dose graph of compound **20** (NSC 846160)

Developmental Therapeutics Program

One Dose Mean Graph

**NSC:** D-846160 / 1	**Conc:** 1.00E-5 Molar	**Test Date:** Jul 10, 2023
**Experiment ID:** 2307OS44		**Report Date:** Sep 01, 2025

Panel/Cell Line	Growth Percent
Leukemia	
CCRF-CEM	33.46
HL-60(TB)	-23.10
K-562	-9.31
MOLT-4	16.19
RPMI-8226	-1.74
SR	0.57
Non-Small Cell Lung Cancer	
A549/ATCC	60.18
EKVX	22.84
HOP-62	61.63
NCI-H226	45.85
NCI-H23	39.80
NCI-H322M	91.12
NCI-H460	16.89
NCI-H522	7.80
Colon Cancer	
COLO 205	-59.92
HCC-2998	-91.84
HCT-116	4.49
HCT-15	0.96
HT29	5.34
KM12	29.69
SW-620	-40.23
CNS Cancer	
SF-268	54.62
SF-295	23.32
SF-539	12.87
SNB-75	21.27
U251	26.72
Melanoma	
LOX IMVI	-69.19
MALME-3M	-84.03
M14	-69.37
MDA-MB-435	-21.33
SK-MEL-2	-52.93
SK-MEL-28	-88.58
SK-MEL-5	-89.68
UACC-257	-58.02
UACC-62	-59.22
Ovarian Cancer	
IGROV1	54.81
OVCAR-3	49.01
OVCAR-4	32.23
OVCAR-5	65.63
OVCAR-8	64.70
NCI/ADR-RES	38.93
SK-OV-3	43.91
Renal Cancer	
786-0	12.98
A498	100.46
ACHN	45.02
CAKI-1	15.31
RXF 393	21.82
SN12C	50.04
UO-31	34.14
Prostate Cancer	
PC-3	34.05
DU-145	54.04
Breast Cancer	
MCF7	4.59
MDA-MB-231/ATCC	37.35
HS 578T	27.64
BT-549	31.63
T-47D	21.45
MDA-MB-468	-29.55
Mean	9.95
Delta	101.79
Range	192.30

Mean Growth Percent - Growth Percent

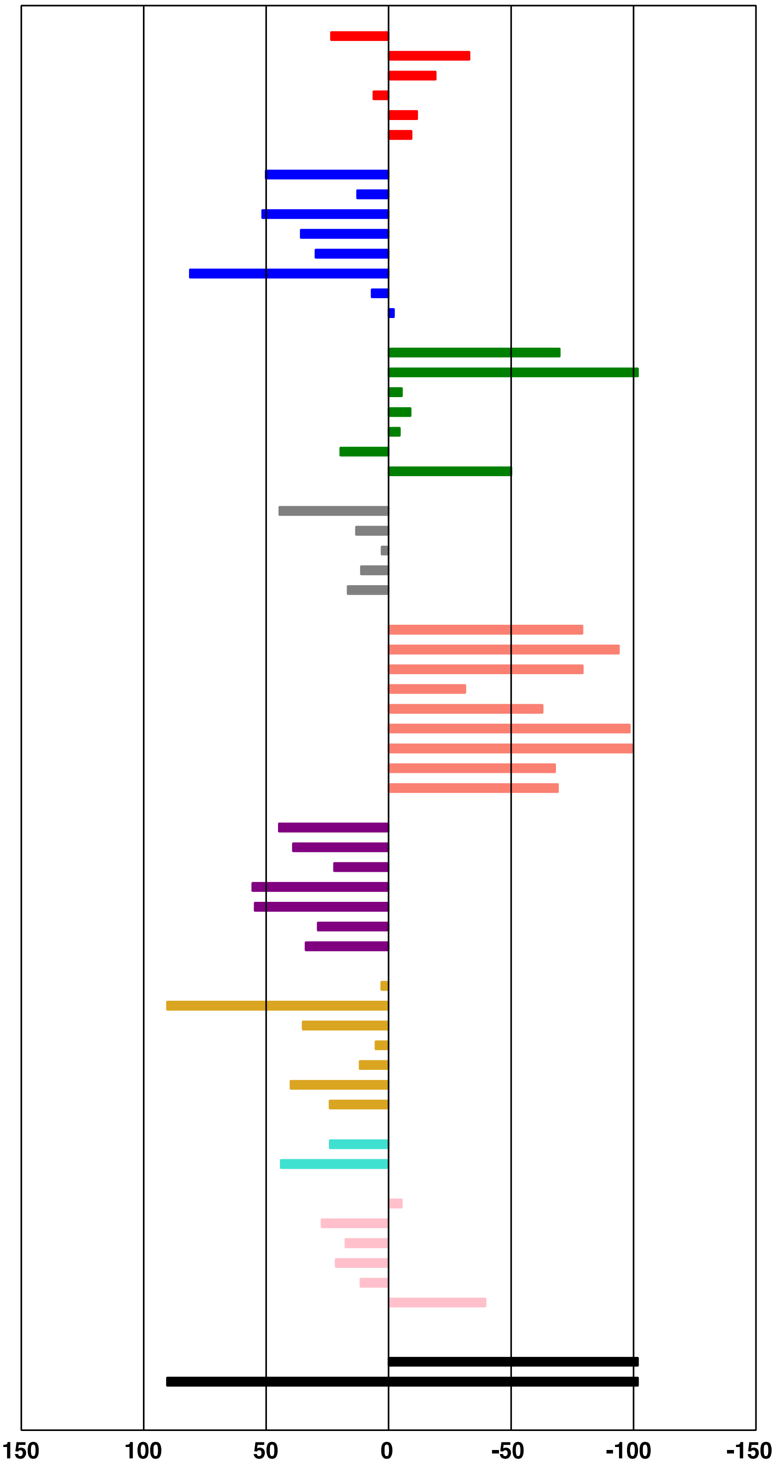

Figure S63: five-dose graph of compound **1** (NSC 846162)

National Cancer Institute Developmental Therapeutics Program<br>In-Vitro Testing Results			
NSC : D - 846162 / 1	Experiment ID : 2310NS93	Test Type : 08	Units : Molar
Report Date : December 01, 2023	Test Date : October 16, 2023	QNS :	MC :
COMI : 44ASA	Stain Reagent : SRB Dual-Pass Related	SSPL : 0J6G	

			Log10 Concentration												
	Time		Mean Optical Densities					Percent Growth							
Panel/Cell Line	Zero	Ctrl	-8.0	-7.0	-6.0	-5.0	-4.0	-8.0	-7.0	-6.0	-5.0	-4.0	GI50	TGI	LC50
Leukemia															
CCRF-CEM	0.490	2.673	2.783	2.829	2.666	1.231	0.267	105	107	100	34	-46	5.69E-6	2.67E-5	> 1.00E-4
HL-60(TB)	0.608	2.794	2.470	2.579	2.421	0.690	0.143	85	90	83	4	-76	2.60E-6	1.11E-5	4.68E-5
K-562	0.271	2.335	2.255	2.238	1.898	0.168	0.086	96	95	79	-38	-68	1.76E-6	4.73E-6	2.48E-5
MOLT-4	0.497	2.529	2.532	2.611	2.389	0.806	0.112	100	104	93	15	-77	3.57E-6	1.46E-5	5.05E-5
RPMI-8226	0.670	2.815	2.738	2.751	2.505	0.581	0.292	96	97	86	-13	-56	2.29E-6	7.33E-6	7.10E-5
SR	0.288	1.815	1.694	1.710	1.435	0.372	0.139	92	93	75	5	-52	2.29E-6	1.25E-5	9.26E-5
Non-Small Cell Lung Cancer															
A549/ATCC	0.434	2.018	1.904	1.930	1.784	1.047	0.064	93	94	85	39	-85	5.71E-6	2.05E-5	5.19E-5
EKVX	0.695	1.925	1.881	1.880	1.848	1.361	0.042	96	96	94	54	-94	1.07E-5	2.32E-5	5.05E-5
HOP-62	0.592	2.023	1.916	1.957	1.933	1.289	0.093	93	95	94	49	-84	9.35E-6	2.32E-5	5.52E-5
HOP-92	0.989	1.671	1.604	1.692	1.504	1.159	0.192	90	103	75	25	-81	3.19E-6	1.72E-5	5.13E-5
NCI-H226	1.181	2.627	2.539	2.583	2.496	1.955	0.421	94	97	91	53	-64	1.07E-5	2.84E-5	7.55E-5
NCI-H23	0.593	2.394	2.356	2.408	2.328	1.525	0.028	98	101	96	52	-95	1.03E-5	2.25E-5	4.92E-5
NCI-H322M	0.756	2.374	2.153	2.233	2.176	1.694	0.028	86	91	88	58	-96	1.13E-5	2.37E-5	5.01E-5
NCI-H460	0.281	2.658	2.702	2.740	2.633	0.123	0.067	102	103	99	-56	-76	2.07E-6	4.33E-6	9.09E-6
NCI-H522	0.878	2.408	2.163	2.255	2.232	1.732	0.055	84	90	88	56	-94	1.09E-5	2.36E-5	5.10E-5
Colon Cancer															
COLO 205	0.397	2.095	2.035	2.143	1.958	0.107	0.132	96	103	92	-73	-67	1.80E-6	3.61E-6	7.25E-6
HCC-2998	0.734	2.844	2.588	2.765	2.680	0.800	0.053	88	96	92	3	-93	2.98E-6	1.08E-5	3.58E-5
HCT-116	0.197	2.271	2.225	2.195	2.052	0.395	0.077	98	96	89	10	-61	3.12E-6	1.36E-5	6.95E-5
HCT-15	0.621	2.675	2.581	2.580	2.481	0.491	0.022	95	95	91	-21	-96	2.31E-6	6.49E-6	2.43E-5
HT29	0.294	1.847	1.754	1.817	1.621	0.158	0.028	94	98	85	-46	-90	1.86E-6	4.45E-6	1.22E-5
KM12	0.618	2.893	2.887	2.877	2.787	0.642	0.047	100	99	95	1	-92	3.02E-6	1.03E-5	3.51E-5
SW-620	0.274	2.080	1.956	1.968	1.773	0.130	0.023	93	94	83	-53	-92	1.75E-6	4.09E-6	9.55E-6
CNS Cancer															
SF-268	0.814	2.569	2.397	2.469	2.411	1.646	0.120	90	94	91	47	-85	8.71E-6	2.28E-5	5.42E-5
SF-295	0.925	2.639	2.489	2.451	2.507	0.361	0.049	91	89	92	-61	-95	1.89E-6	4.00E-6	8.48E-6
SF-539	0.749	2.450	2.311	2.396	2.322	1.170	0.011	92	97	92	25	-99	4.24E-6	1.59E-5	4.04E-5
SNB-19	0.653	2.035	1.921	1.980	1.909	1.475	0.039	92	96	91	59	-94	1.15E-5	2.44E-5	5.17E-5
SNB-75	1.119	2.020	1.900	1.940	1.816	1.361	0.025	87	91	77	27	-98	3.47E-6	1.64E-5	4.14E-5
U251	0.418	1.697	1.589	1.657	1.556	0.750	0.026	92	97	89	26	-94	4.16E-6	1.65E-5	4.30E-5
Melanoma															
LOX IMVI	0.317	2.526	2.399	2.425	2.399	0.047	0.024	94	95	94	-85	-93	1.76E-6	3.35E-6	6.37E-6
MALME-3M	0.636	1.456	1.401	1.410	1.319	0.037	0.023	93	94	83	-94	-96	1.54E-6	2.94E-6	5.64E-6
M14	0.360	1.682	1.556	1.740	1.642	0.161	0.045	91	104	97	-55	-88	2.03E-6	4.33E-6	9.21E-6
MDA-MB-435	0.558	2.532	2.465	2.544	2.341	0.142	0.009	97	101	90	-75	-98	1.76E-6	3.53E-6	7.10E-6
SK-MEL-2	1.711	2.863	2.778	2.764	2.798	2.273	0.039	93	91	94	49	-98	9.38E-6	2.15E-5	4.72E-5
SK-MEL-28	0.725	2.160	2.070	2.164	2.104	0.192	0.002	94	100	96	-74	-100	1.87E-6	3.68E-6	7.26E-6
SK-MEL-5	0.822	3.142	3.039	3.124	2.977	0.013	0.007	96	99	93	-98	-99	1.68E-6	3.06E-6	5.58E-6
UACC-257	1.052	2.340	2.245	2.225	2.236	0.408	0.075	93	91	92	-61	-93	1.88E-6	3.98E-6	8.44E-6
UACC-62	0.769	2.792	2.619	2.656	2.629	0.288	0.014	91	93	92	-63	-98	1.87E-6	3.94E-6	8.29E-6
Ovarian Cancer															
IGROV1	0.522	2.056	1.869	1.985	1.880	0.971	0.073	88	95	88	29	-86	4.47E-6	1.79E-5	4.87E-5
OVCAR-3	0.582	1.956	1.962	1.953	1.834	1.205	0.037	100	100	91	45	-94	7.91E-6	2.12E-5	4.85E-5
OVCAR-4	0.788	2.354	2.284	2.389	2.236	1.763	0.105	96	102	92	62	-87	1.21E-5	2.62E-5	5.67E-5
OVCAR-5	0.509	1.331	1.265	1.304	1.280	0.775	0.011	92	97	94	32	-98	5.15E-6	1.77E-5	4.28E-5
OVCAR-8	0.372	1.919	1.873	1.925	1.801	1.319	0.077	97	100	92	61	-79	1.20E-5	2.73E-5	6.19E-5
NCI/ADR-RES	0.508	1.970	1.885	1.981	1.808	1.341	0.094	94	101	89	57	-81	1.12E-5	2.58E-5	5.92E-5
SK-OV-3	0.648	1.817	1.769	1.783	1.702	1.343	0.064	96	97	90	59	-90	1.16E-5	2.50E-5	5.39E-5
Renal Cancer															
786-0	0.607	2.783	2.746	2.773	2.739	0.808	0.116	98	100	98	9	-81	3.47E-6	1.27E-5	4.54E-5
A498	1.468	2.400	2.262	2.320	2.311	2.038	0.152	85	91	90	61	-90	1.19E-5	2.54E-5	5.46E-5
ACHN	0.435	2.057	1.984	2.104	2.022	1.227	-0.002	95	103	98	49	-100	9.46E-6	2.13E-5	4.61E-5
CAKI-1	1.050	2.731	2.642	2.634	2.525	1.054	0.029	95	94	88	0	-97	2.70E-6	1.01E-5	3.28E-5
RXF 393	1.108	1.884	1.783	1.894	1.745	1.168	0.156	87	101	82	8	-86	2.69E-6	1.21E-5	4.13E-5
SN12C	0.516	2.164	2.079	2.141	1.931	1.435	0.024	95	99	86	56	-95	1.09E-5	2.34E-5	5.00E-5
TK-10	0.961	1.926	1.752	1.741	1.766	1.303	0.020	82	81	83	35	-98	4.96E-6	1.84E-5	4.37E-5
UO-31	0.716	2.114	1.841	1.949	1.855	1.088	0.041	80	88	81	27	-94	3.74E-6	1.66E-5	4.30E-5
Prostate Cancer															
PC-3	0.618	2.476	2.410	2.535	2.441	1.497	0.171	96	103	98	47	-72	8.85E-6	2.49E-5	6.51E-5
DU-145	0.276	1.268	1.232	1.284	1.232	0.697	0.005	96	102	96	42	-98	7.24E-6	2.00E-5	4.53E-5
Breast Cancer															
MCF7	1.238	3.258	3.152	3.112	3.023	1.800	0.246	95	93	88	28	-80	4.30E-6	1.81E-5	5.26E-5
MDA-MB-231/ATCC	0.549	1.248	1.258	1.284	1.237	0.764	0.037	101	105	98	31	-93	5.19E-6	1.77E-5	4.48E-5
HS 578T	1.446	2.461	2.306	2.330	2.306	1.728	0.755	85	87	85	28	-48	4.07E-6	2.33E-5	> 1.00E-4
BT-549	1.082	2.305	2.172	2.344	2.228	1.667	0.082	89	103	94	48	-92	8.96E-6	2.19E-5	4.98E-5
T-47D	0.545	1.489	1.445	1.446	1.325	0.769	0.149	95	95	83	24	-73	3.58E-6	1.76E-5	5.81E-5
MDA-MB-468	1.122	2.599	2.601	2.587	2.442	1.092	0.088	100	99	89	-3	-92	2.68E-6	9.35E-6	3.38E-5

Figure S64: five-dose graph of compound **5** (NSC 846166)

National Cancer Institute Developmental Therapeutics Program
In-Vitro Testing Results

NSC : D - 846166 / 1	Experiment ID : 2310NS93	Test Type : 08	Units : Molar
Report Date : November 29, 2023	Test Date : October 16, 2023	QNS :	MC :
COMI : 31ASA	Stain Reagent : SRB Dual-Pass Related	SSPL : 1CYF	

			Log10 Concentration												
	Time		Mean Optical Densities					Percent Growth							
Panel/Cell Line	Zero	Ctrl	-8.0	-7.0	-6.0	-5.0	-4.0	-8.0	-7.0	-6.0	-5.0	-4.0	GI50	TGI	LC50
Leukemia															
CCRF-CEM	0.490	2.757	2.716	2.717	2.758	1.586	1.763	98	98	100	48	56	.	> 1.00E-4	> 1.00E-4
HL-60(TB)	0.608	2.955	2.722	2.623	2.607	1.364	1.230	90	86	85	32	26	4.61E-6	> 1.00E-4	> 1.00E-4
K-562	0.271	2.382	2.411	2.333	2.258	0.798	1.080	101	98	94	25	38	4.35E-6	> 1.00E-4	> 1.00E-4
MOLT-4	0.497	2.518	2.493	2.427	2.317	1.164	1.057	99	95	90	33	28	5.03E-6	> 1.00E-4	> 1.00E-4
RPMI-8226	0.670	2.702	2.813	2.735	2.698	1.271	1.535	105	102	100	30	43	5.12E-6	> 1.00E-4	> 1.00E-4
SR	0.288	1.793	1.706	1.611	1.726	0.944	1.028	94	88	96	44	49	7.52E-6	> 1.00E-4	> 1.00E-4
Non-Small Cell Lung Cancer															
A549/ATCC	0.434	2.113	2.043	2.025	2.110	1.544	1.699	96	95	100	66	75	> 1.00E-4	> 1.00E-4	> 1.00E-4
EKVX	0.695	1.966	1.877	1.859	1.859	1.456	1.472	93	92	92	60	61	> 1.00E-4	> 1.00E-4	> 1.00E-4
HOP-62	0.592	2.040	1.945	1.862	1.882	1.747	1.800	93	88	89	80	83	> 1.00E-4	> 1.00E-4	> 1.00E-4
HOP-92	0.989	1.715	1.670	1.595	1.570	1.282	1.380	94	83	80	40	54	.	> 1.00E-4	> 1.00E-4
NCI-H226	1.181	2.686	2.588	2.613	2.546	2.083	2.147	93	95	91	60	64	> 1.00E-4	> 1.00E-4	> 1.00E-4
NCI-H23	0.593	2.436	2.361	2.384	2.309	1.849	2.036	96	97	93	68	78	> 1.00E-4	> 1.00E-4	> 1.00E-4
NCI-H322M	0.756	2.305	2.248	2.484	2.385	1.938	1.871	96	112	105	76	72	> 1.00E-4	> 1.00E-4	> 1.00E-4
NCI-H460	0.281	2.614	2.773	2.791	2.747	1.327	1.673	107	108	106	45	60	.	> 1.00E-4	> 1.00E-4
NCI-H522	0.878	2.559	2.390	2.391	2.397	1.986	1.957	90	90	90	66	64	> 1.00E-4	> 1.00E-4	> 1.00E-4
Colon Cancer															
COLO 205	0.397	2.014	1.965	1.928	1.961	0.918	1.018	97	95	97	32	38	5.30E-6	> 1.00E-4	> 1.00E-4
HCC-2998	0.734	2.830	2.605	2.686	2.673	1.900	1.896	89	93	93	56	55	> 1.00E-4	> 1.00E-4	> 1.00E-4
HCT-116	0.197	2.375	2.267	2.273	2.217	0.914	1.035	95	95	93	33	38	5.18E-6	> 1.00E-4	> 1.00E-4
HCT-15	0.621	2.678	2.579	2.456	2.548	1.380	2.163	95	89	94	37	75	.	> 1.00E-4	> 1.00E-4
HT29	0.294	2.061	2.052	2.042	2.024	0.900	0.716	100	99	98	34	24	5.67E-6	> 1.00E-4	> 1.00E-4
KM12	0.618	2.866	2.814	2.815	2.847	1.874	1.751	98	98	99	56	50	> 1.00E-4	> 1.00E-4	> 1.00E-4
SW-620	0.274	2.033	1.953	1.917	1.996	1.297	1.201	95	93	98	58	53	> 1.00E-4	> 1.00E-4	> 1.00E-4
CNS Cancer															
SF-268	0.814	2.583	2.533	2.545	2.517	2.085	2.214	97	98	96	72	79	> 1.00E-4	> 1.00E-4	> 1.00E-4
SF-295	0.925	2.873	2.771	2.560	2.513	1.879	1.519	95	84	82	49	31	9.29E-6	> 1.00E-4	> 1.00E-4
SF-539	0.749	2.440	2.460	2.392	2.420	2.216	1.895	101	97	99	87	68	> 1.00E-4	> 1.00E-4	> 1.00E-4
SNB-19	0.653	2.012	1.931	1.884	1.945	1.762	1.657	94	91	95	82	74	> 1.00E-4	> 1.00E-4	> 1.00E-4
SNB-75	1.119	2.078	2.028	2.042	2.015	1.775	1.738	95	96	93	68	65	> 1.00E-4	> 1.00E-4	> 1.00E-4
U251	0.418	1.812	1.694	1.605	1.648	1.204	1.446	92	85	88	56	74	> 1.00E-4	> 1.00E-4	> 1.00E-4
Melanoma															
LOX IMVI	0.317	2.505	2.448	2.351	2.444	1.773	1.650	97	93	97	67	61	> 1.00E-4	> 1.00E-4	> 1.00E-4
MALME-3M	0.636	1.504	1.512	1.494	1.526	1.170	1.419	101	99	103	62	90	> 1.00E-4	> 1.00E-4	> 1.00E-4
M14	0.360	1.616	1.642	1.623	1.605	0.944	1.248	102	101	99	46	71	.	> 1.00E-4	> 1.00E-4
MDA-MB-435	0.558	2.540	2.514	2.466	2.523	1.940	1.732	99	96	99	70	59	> 1.00E-4	> 1.00E-4	> 1.00E-4
SK-MEL-2	1.711	3.014	2.963	2.904	2.950	2.773	2.907	96	92	95	81	92	> 1.00E-4	> 1.00E-4	> 1.00E-4
SK-MEL-28	0.725	2.166	2.082	2.103	2.088	1.738	1.804	94	96	95	70	75	> 1.00E-4	> 1.00E-4	> 1.00E-4
SK-MEL-5	0.822	3.138	3.031	3.041	3.022	1.911	1.686	95	96	95	47	37	8.67E-6	> 1.00E-4	> 1.00E-4
UACC-257	1.052	2.460	2.336	2.301	2.407	2.002	2.099	91	89	96	67	74	> 1.00E-4	> 1.00E-4	> 1.00E-4
UACC-62	0.769	2.787	2.502	2.424	2.503	2.119	2.077	86	82	86	67	65	> 1.00E-4	> 1.00E-4	> 1.00E-4
Ovarian Cancer															
IGROV1	0.522	2.028	2.552	2.330	2.146	1.711	1.416	135	120	108	79	59	> 1.00E-4	> 1.00E-4	> 1.00E-4
OVCAR-3	0.582	2.024	2.151	2.072	1.952	1.514	1.538	109	103	95	65	66	> 1.00E-4	> 1.00E-4	> 1.00E-4
OVCAR-4	0.788	2.350	2.349	2.332	2.290	1.661	1.704	100	99	96	56	59	> 1.00E-4	> 1.00E-4	> 1.00E-4
OVCAR-5	0.509	1.353	1.273	1.231	1.278	1.045	1.182	90	86	91	63	80	> 1.00E-4	> 1.00E-4	> 1.00E-4
OVCAR-8	0.372	1.933	1.911	1.887	1.885	1.460	1.476	99	97	97	70	71	> 1.00E-4	> 1.00E-4	> 1.00E-4
NCI/ADR-RES	0.508	2.034	2.081	1.973	1.805	1.386	1.550	103	96	85	58	68	> 1.00E-4	> 1.00E-4	> 1.00E-4
SK-OV-3	0.648	1.760	1.785	1.760	1.757	1.598	1.727	102	100	100	85	97	> 1.00E-4	> 1.00E-4	> 1.00E-4
Renal Cancer															
786-0	0.607	2.682	2.709	2.620	2.712	2.146	2.371	101	97	101	74	85	> 1.00E-4	> 1.00E-4	> 1.00E-4
A498	1.468	2.381	2.338	2.265	2.288	2.411	2.596	95	87	90	103	123	> 1.00E-4	> 1.00E-4	> 1.00E-4
ACHN	0.435	2.063	2.047	1.987	2.033	1.471	1.378	99	95	98	64	58	> 1.00E-4	> 1.00E-4	> 1.00E-4
CAKI-1	1.050	2.709	2.569	2.580	2.681	2.213	2.036	92	92	98	70	59	> 1.00E-4	> 1.00E-4	> 1.00E-4
RXF 393	1.108	1.864	1.845	1.840	1.849	1.457	1.448	97	97	98	46	45	8.44E-6	> 1.00E-4	> 1.00E-4
SN12C	0.516	2.140	1.985	1.936	1.953	1.446	0.834	90	87	88	57	20	1.56E-5	> 1.00E-4	> 1.00E-4
TK-10	0.961	2.061	1.918	1.937	2.078	1.835	2.219	87	89	102	79	114	> 1.00E-4	> 1.00E-4	> 1.00E-4
UO-31	0.716	2.151	1.883	1.860	1.866	1.210	1.793	81	80	80	34	75	.	> 1.00E-4	> 1.00E-4
Prostate Cancer															
PC-3	0.618	2.550	2.403	2.454	2.386	1.197	1.468	92	95	92	30	44	4.72E-6	> 1.00E-4	> 1.00E-4
DU-145	0.276	1.208	1.269	1.248	1.266	1.034	0.929	107	104	106	81	70	> 1.00E-4	> 1.00E-4	> 1.00E-4
Breast Cancer															
MCF7	1.238	3.251	3.174	3.097	3.075	2.411	2.876	96	92	91	58	81	> 1.00E-4	> 1.00E-4	> 1.00E-4
MDA-MB-231/ATCC	0.549	1.262	1.243	1.231	1.245	1.158	1.041	97	96	98	85	69	> 1.00E-4	> 1.00E-4	> 1.00E-4
HS 578T	1.446	2.457	2.364	2.354	2.342	2.152	2.209	91	90	89	70	75	> 1.00E-4	> 1.00E-4	> 1.00E-4
BT-549	1.082	2.247	2.173	2.188	2.187	1.924	2.116	94	95	95	72	89	> 1.00E-4	> 1.00E-4	> 1.00E-4
T-47D	0.545	1.483	1.394	1.322	1.334	0.827	1.080	91	83	84	30	57	.	> 1.00E-4	> 1.00E-4
MDA-MB-468	1.122	2.654	2.648	2.605	2.594	1.781	2.003	100	97	96	43	57	.	> 1.00E-4	> 1.00E-4

Figure S65: five-dose graph of compound **10** (NSC 846171)

National Cancer Institute Developmental Therapeutics Program
In-Vitro Testing Results

NSC : D - 846171 / 1	Experiment ID : 2310NS93	Test Type : 08	Units : Molar
Report Date : November 29, 2023	Test Date : October 16, 2023	QNS :	MC :
COMI : 55ASA	Stain Reagent : SRB Dual-Pass Related	SSPL : 1CYF	

			Log10 Concentration													
	Time		Mean Optical Densities					Percent Growth								
Panel/Cell Line	Zero	Ctrl	-8.1	-7.1	-6.1	-5.1	-4.1	-8.1	-7.1	-6.1	-5.1	-4.1		GI50	TGI	LC50
Leukemia																
CCRF-CEM	0.490	2.757	2.647	2.690	2.719	1.607	0.473	95	97	98	49	-4		8.21E-6	7.27E-5	> 8.50E-5
HL-60(TB)	0.608	2.955	2.752	2.748	2.661	1.585	0.408	91	91	87	42	-33		5.58E-6	3.07E-5	> 8.50E-5
K-562	0.271	2.382	2.478	2.400	2.215	0.374	0.255	105	101	92	5	-6		2.58E-6	2.37E-5	> 8.50E-5
MOLT-4	0.497	2.518	2.518	2.564	2.421	1.230	0.408	100	102	95	36	-18		4.97E-6	3.97E-5	> 8.50E-5
RPMI-8226	0.670	2.702	2.733	2.673	2.599	0.930	0.426	102	99	95	13	-36		3.00E-6	1.55E-5	> 8.50E-5
SR	0.288	1.793	1.666	1.738	1.586	0.321	0.258	92	96	86	2	-10		2.29E-6	1.26E-5	> 8.50E-5
Non-Small Cell Lung Cancer																
A549/ATCC	0.434	2.113	1.990	2.084	2.065	1.385	0.539	93	98	97	57	6		1.15E-5	> 8.50E-5	> 8.50E-5
EKVX	0.695	1.966	1.847	1.924	1.878	1.338	0.613	91	97	93	51	-12		8.67E-6	5.49E-5	> 8.50E-5
HOP-62	0.592	2.040	1.975	2.006	1.850	1.718	0.830	96	98	87	78	16		2.41E-5	> 8.50E-5	> 8.50E-5
HOP-92	0.989	1.715	1.686	1.662	1.591	1.201	0.936	96	93	83	29	-5		3.48E-6	5.93E-5	> 8.50E-5
NCI-H226	1.181	2.686	2.560	2.645	2.561	1.911	1.081	92	97	92	49	-8		7.85E-6	6.04E-5	> 8.50E-5
NCI-H23	0.593	2.436	2.355	2.414	2.296	1.742	0.582	96	99	92	62	-2		1.32E-5	7.93E-5	> 8.50E-5
NCI-H322M	0.756	2.305	2.225	2.347	2.040	1.653	0.952	95	103	83	58	13		1.27E-5	> 8.50E-5	> 8.50E-5
NCI-H460	0.281	2.614	2.673	2.666	2.677	0.874	0.128	103	102	103	25	-54		4.09E-6	1.77E-5	7.48E-5
NCI-H522	0.878	2.559	2.344	2.465	2.521	2.110	0.237	87	94	98	73	-73		1.23E-5	2.69E-5	5.92E-5
Colon Cancer																
COLO 205	0.397	2.014	2.084	2.164	1.875	0.297	0.159	104	109	91	-25	-60		1.92E-6	5.16E-6	4.39E-5
HCC-2998	0.734	2.830	2.578	2.922	2.654	0.799	0.282	88	104	92	3	-62		2.51E-6	9.49E-6	5.62E-5
HCT-116	0.197	2.375	2.236	2.158	2.084	1.026	0.147	94	90	87	38	-25		4.83E-6	3.38E-5	> 8.50E-5
HCT-15	0.621	2.678	2.609	2.649	2.591	1.326	0.468	97	99	96	34	-25		4.71E-6	3.24E-5	> 8.50E-5
HT29	0.294	2.061	1.907	2.013	1.987	0.561	0.083	91	97	96	15	-72		3.14E-6	1.27E-5	4.77E-5
KM12	0.618	2.866	2.815	2.898	2.803	1.408	0.129	98	101	97	35	-79		4.89E-6	1.72E-5	4.72E-5
SW-620	0.274	2.033	1.977	1.938	1.846	1.194	0.096	97	95	89	52	-65		8.89E-6	2.37E-5	6.32E-5
CNS Cancer																
SF-268	0.814	2.583	2.450	2.439	2.370	1.909	1.751	92	92	88	62	53		> 8.50E-5	> 8.50E-5	> 8.50E-5
SF-295	0.925	2.873	2.488	2.547	2.508	1.773	0.210	80	83	81	44	-77		5.73E-6	1.95E-5	5.05E-5
SF-539	0.749	2.440	2.267	2.471	2.411	1.741	0.421	90	102	98	59	-44		1.03E-5	3.18E-5	> 8.50E-5
SNB-19	0.653	2.012	1.872	1.927	1.904	1.697	1.285	90	94	92	77	46		6.51E-5	> 8.50E-5	> 8.50E-5
SNB-75	1.119	2.078	1.878	1.948	1.829	1.638	0.851	79	86	74	54	-24		9.60E-6	4.19E-5	> 8.50E-5
U251	0.418	1.812	1.694	1.760	1.707	1.086	0.591	92	96	92	48	12		7.64E-6	> 8.50E-5	> 8.50E-5
Melanoma																
LOX IMVI	0.317	2.505	2.411	2.445	2.371	0.845	0.179	96	97	94	24	-44		3.62E-6	1.93E-5	> 8.50E-5
MALME-3M	0.636	1.504	1.424	1.454	1.366	0.419	0.039	91	94	84	-34	-94		1.65E-6	4.37E-6	1.56E-5
M14	0.360	1.616	1.651	1.625	1.658	0.360	0.111	103	101	103	0	-69		2.78E-6	8.47E-6	4.48E-5
MDA-MB-435	0.558	2.540	2.427	2.493	2.380	1.521	0.023	94	98	92	49	-96		7.88E-6	1.84E-5	4.09E-5
SK-MEL-2	1.711	3.014	2.906	2.936	3.022	2.794	0.820	92	94	101	83	-52		1.49E-5	3.50E-5	8.20E-5
SK-MEL-28	0.725	2.166	2.207	2.191	2.080	0.731	0.057	103	102	94	0	-92		2.51E-6	8.59E-6	2.98E-5
SK-MEL-5	0.822	3.138	3.012	3.115	2.999	0.978	0.014	95	99	94	7	-98		2.71E-6	9.85E-6	2.95E-5
UACC-257	1.052	2.460	2.311	2.461	2.406	0.934	0.168	89	100	96	-11	-84		2.29E-6	6.68E-6	2.90E-5
UACC-62	0.769	2.787	2.515	2.495	2.505	1.837	0.312	86	85	86	53	-59		9.02E-6	2.51E-5	7.00E-5
Ovarian Cancer																
IGROV1	0.522	2.028	1.964	1.974	1.895	1.253	0.557	96	96	91	48	2		7.84E-6	> 8.50E-5	> 8.50E-5
OVCAR-3	0.582	2.024	1.895	1.986	1.818	1.334	0.293	91	97	86	52	-50		8.93E-6	2.76E-5	> 8.50E-5
OVCAR-4	0.788	2.350	2.337	2.380	2.214	1.622	0.788	99	102	91	53	0		9.83E-6	8.48E-5	> 8.50E-5
OVCAR-5	0.509	1.353	1.313	1.348	1.337	0.960	0.191	95	99	98	53	-63		9.09E-6	2.45E-5	6.62E-5
OVCAR-8	0.372	1.933	1.888	1.936	1.839	1.423	0.207	97	100	94	67	-44		1.22E-5	3.41E-5	> 8.50E-5
NCI/ADR-RES	0.508	2.034	2.000	2.097	1.822	1.396	0.309	98	104	86	58	-39		1.03E-5	3.37E-5	> 8.50E-5
SK-OV-3	0.648	1.760	1.741	1.815	1.747	1.530	1.115	98	105	99	79	42		5.18E-5	> 8.50E-5	> 8.50E-5
Renal Cancer																
786-0	0.607	2.682	2.549	2.693	2.637	1.837	0.741	94	101	98	59	6		1.27E-5	> 8.50E-5	> 8.50E-5
A498	1.468	2.381	2.210	2.296	2.274	2.285	1.681	81	91	88	89	23		3.36E-5	> 8.50E-5	> 8.50E-5
ACHN	0.435	2.063	1.963	2.089	1.993	1.333	0.301	94	102	96	55	-31		9.76E-6	3.72E-5	> 8.50E-5
CAKI-1	1.050	2.709	2.584	2.588	2.604	1.782	1.002	92	93	94	44	-5		6.46E-6	6.83E-5	> 8.50E-5
RXF 393	1.108	1.864	1.771	1.833	1.734	1.082	0.688	88	96	83	-2	-38		2.06E-6	7.97E-6	> 8.50E-5
SN12C	0.516	2.140	1.998	2.026	1.934	1.432	0.048	91	93	87	56	-91		9.39E-6	2.05E-5	4.49E-5
TK-10	0.961	2.061	1.896	1.975	2.064	1.768	0.825	85	92	100	73	-14		1.57E-5	5.85E-5	> 8.50E-5
UO-31	0.716	2.151	1.889	2.036	1.893	1.218	1.371	82	92	82	35	46		4.08E-6	> 8.50E-5	> 8.50E-5
Prostate Cancer																
PC-3	0.618	2.550	2.391	2.499	2.381	1.217	0.468	92	97	91	31	-24		4.11E-6	3.09E-5	> 8.50E-5
DU-145	0.276	1.208	1.224	1.276	1.207	0.857	0.078	102	107	100	62	-72		1.05E-5	2.48E-5	5.85E-5
Breast Cancer																
MCF7	1.238	3.251	3.111	3.136	3.109	2.460	0.143	93	94	93	61	-88		1.00E-5	2.17E-5	4.70E-5
MDA-MB-231/ATCC	0.549	1.262	1.264	1.281	1.253	1.017	0.173	100	103	99	66	-68		1.11E-5	2.62E-5	6.19E-5
HS 578T	1.446	2.457	2.281	2.295	2.273	2.008	1.490	83	84	82	56	4		1.09E-5	> 8.50E-5	> 8.50E-5
BT-549	1.082	2.247	2.135	2.231	2.190	1.761	0.305	90	99	95	58	-72		9.84E-6	2.38E-5	5.77E-5
T-47D	0.545	1.483	1.421	1.459	1.426	1.032	0.375	93	97	94	52	-31		8.97E-6	3.58E-5	> 8.50E-5
MDA-MB-468	1.122	2.654	2.627	2.711	2.464	1.392	0.189	98	104	88	18	-83		2.93E-6	1.27E-5	3.98E-5

Figure S66: five-dose graph of compound **11** (NSC 846172)

National Cancer Institute Developmental Therapeutics Program
In-Vitro Testing Results

NSC : D - 846172 / 1	Experiment ID : 2310NS93	Test Type : 08	Units : Molar
Report Date : November 29, 2023	Test Date : October 16, 2023	QNS :	MC :
COMI : 61ASA	Stain Reagent : SRB Dual-Pass Related	SSPL : 1CYF	

			Log10 Concentration												
	Time		Mean Optical Densities					Percent Growth							
Panel/Cell Line	Zero	Ctrl	-8.0	-7.0	-6.0	-5.0	-4.0	-8.0	-7.0	-6.0	-5.0	-4.0	GI50	TGI	LC50
Leukemia															
CCRF-CEM	0.490	2.748	2.716	2.635	2.581	0.950	0.118	99	95	93	20	-76	3.89E-6	1.63E-5	5.38E-5
HL-60(TB)	0.608	2.576	2.410	2.408	2.363	0.707	0.184	92	91	89	5	-70	2.92E-6	1.17E-5	5.44E-5
K-562	0.271	2.329	2.338	2.245	2.136	0.249	0.136	100	96	91	-8	-50	2.57E-6	8.24E-6	> 1.00E-4
MOLT-4	0.497	2.530	2.354	2.266	2.220	0.579	0.188	91	87	85	4	-62	2.69E-6	1.15E-5	6.55E-5
RPMI-8226	0.670	2.649	2.628	2.560	2.433	0.502	0.250	99	96	89	-25	-63	2.20E-6	6.02E-6	4.58E-5
SR	0.288	1.842	1.885	1.675	1.544	0.333	0.292	103	89	81	3	0	2.49E-6	> 1.00E-4	> 1.00E-4
Non-Small Cell Lung Cancer															
A549/ATCC	0.434	1.994	1.922	1.916	1.919	1.202	0.082	95	95	95	49	-81	9.61E-6	2.38E-5	5.76E-5
EKVX	0.695	1.935	1.861	1.826	1.847	1.181	0.021	94	91	93	39	-97	6.28E-6	1.94E-5	4.51E-5
HOP-62	0.592	2.006	1.960	1.913	1.934	1.511	0.075	97	93	95	65	-87	1.25E-5	2.67E-5	5.69E-5
HOP-92	0.989	1.623	1.546	1.527	1.476	1.087	0.133	88	85	77	15	-87	2.73E-6	1.42E-5	4.38E-5
NCI-H226	1.181	2.626	2.623	2.625	2.488	1.764	0.369	100	100	90	40	-69	6.41E-6	2.34E-5	6.73E-5
NCI-H23	0.593	2.399	2.398	2.263	2.245	1.389	0.028	100	92	91	44	-95	7.50E-6	2.07E-5	4.73E-5
NCI-H322M	0.756	2.283	2.116	2.105	2.066	1.704	0.089	89	88	86	62	-88	1.20E-5	2.59E-5	5.57E-5
NCI-H460	0.281	2.610	2.816	2.786	2.687	0.437	0.034	109	108	103	7	-88	3.56E-6	1.18E-5	3.97E-5
NCI-H522	0.878	2.509	2.405	2.321	2.323	1.708	0.096	94	89	89	51	-89	1.01E-5	2.31E-5	5.26E-5
Colon Cancer															
COLO 205	0.397	2.057	2.079	2.025	1.818	0.087	0.050	101	98	86	-78	-87	1.65E-6	3.33E-6	6.74E-6
HCC-2998	0.734	2.947	2.766	2.848	2.616	0.283	0.049	92	96	85	-62	-93	1.73E-6	3.80E-6	8.35E-6
HCT-116	0.197	2.411	2.331	2.258	2.134	0.563	0.057	96	93	87	17	-71	3.37E-6	1.54E-5	5.75E-5
HCT-15	0.621	2.701	2.665	2.656	2.551	0.970	0.009	98	98	93	17	-99	3.65E-6	1.40E-5	3.79E-5
HT29	0.294	2.011	1.870	1.907	1.749	0.312	0.041	92	94	85	1	-86	2.60E-6	1.03E-5	3.86E-5
KM12	0.618	2.914	2.909	2.819	2.815	1.105	0.042	100	96	96	21	-93	4.11E-6	1.53E-5	4.19E-5
SW-620	0.274	2.068	2.064	2.050	1.933	0.556	0.035	100	99	92	16	-87	3.57E-6	1.42E-5	4.34E-5
CNS Cancer															
SF-268	0.814	2.605	2.560	2.433	2.523	1.479	0.120	97	90	95	37	-85	6.02E-6	2.01E-5	5.15E-5
SF-295	0.925	2.735	2.694	2.629	2.705	0.413	0.049	98	94	98	-55	-95	2.06E-6	4.36E-6	9.23E-6
SF-539	0.749	2.475	2.420	2.431	2.390	1.165	0.012	97	97	95	24	-98	4.32E-6	1.57E-5	4.02E-5
SNB-19	0.653	2.011	1.894	1.846	1.884	1.381	0.003	91	88	91	54	-100	1.06E-5	2.24E-5	4.74E-5
SNB-75	1.119	2.041	2.058	1.897	1.915	1.627	0.102	102	84	86	55	-91	1.08E-5	2.38E-5	5.24E-5
U251	0.418	1.646	1.588	1.566	1.575	0.588	0.042	95	93	94	14	-90	3.55E-6	1.36E-5	4.11E-5
Melanoma															
LOX IMVI	0.317	2.489	2.458	2.344	2.366	0.197	0.010	99	93	94	-38	-97	2.16E-6	5.16E-6	1.60E-5
MALME-3M	0.636	1.551	1.563	1.639	1.572	0.093	0.085	101	110	102	-85	-87	1.90E-6	3.51E-6	6.47E-6
M14	0.360	1.675	1.634	1.657	1.609	0.084	0.034	97	99	95	-77	-91	1.83E-6	3.58E-6	6.99E-6
MDA-MB-435	0.558	2.600	2.530	2.502	2.511	0.476	0.066	97	95	96	-15	-88	2.59E-6	7.36E-6	3.02E-5
SK-MEL-2	1.711	3.001	2.938	2.865	2.969	2.284	0.089	95	89	98	44	-95	7.85E-6	2.08E-5	4.77E-5
SK-MEL-28	0.725	2.165	2.128	2.075	2.081	0.087	0.005	97	94	94	-88	-99	1.75E-6	3.29E-6	6.18E-6
SK-MEL-5	0.822	3.162	3.079	3.047	3.057	0.015	0.003	96	95	96	-98	-100	1.72E-6	3.11E-6	5.64E-6
UACC-257	1.052	2.284	2.197	2.157	2.180	0.192	0.060	93	90	92	-82	-94	1.74E-6	3.38E-6	6.56E-6
UACC-62	0.769	2.752	2.508	2.397	2.456	0.761	0.010	88	82	85	-1	-99	2.55E-6	9.73E-6	3.17E-5
Ovarian Cancer															
IGROV1	0.522	2.033	1.913	2.065	1.829	1.148	0.198	92	102	86	41	-62	6.44E-6	2.51E-5	7.64E-5
OVCAR-3	0.582	1.954	2.056	2.042	1.942	1.193	0.037	107	106	99	44	-94	7.93E-6	2.10E-5	4.83E-5
OVCAR-4	0.788	2.410	2.398	2.298	2.260	1.417	0.084	99	93	91	39	-89	6.08E-6	2.01E-5	4.93E-5
OVCAR-5	0.509	1.373	1.292	1.278	1.276	0.915	0.007	91	89	89	47	-99	8.45E-6	2.10E-5	4.63E-5
OVCAR-8	0.372	1.795	1.777	1.741	1.699	1.034	0.115	99	96	93	46	-69	8.41E-6	2.52E-5	6.82E-5
NCI/ADR-RES	0.508	1.981	1.987	1.868	1.777	1.133	0.073	100	92	86	42	-86	6.71E-6	2.14E-5	5.27E-5
SK-OV-3	0.648	1.809	1.912	1.791	1.789	1.419	0.041	109	98	98	66	-94	1.27E-5	2.60E-5	5.34E-5
Renal Cancer															
786-0	0.607	2.760	2.740	2.686	2.627	1.497	0.113	99	97	94	41	-81	6.83E-6	2.17E-5	5.55E-5
A498	1.468	2.417	2.336	2.281	2.296	2.121	0.113	91	86	87	69	-92	1.31E-5	2.67E-5	5.46E-5
ACHN	0.435	2.062	2.049	2.085	2.040	1.134	-0.001	99	101	99	43	-100	7.48E-6	2.00E-5	4.47E-5
CAKI-1	1.050	2.711	2.617	2.544	2.600	1.328	0.145	94	90	93	17	-86	3.68E-6	1.45E-5	4.45E-5
RXF 393	1.108	1.918	1.893	1.779	1.717	0.988	0.105	97	83	75	-11	-91	1.96E-6	7.47E-6	3.10E-5
SN12C	0.516	2.108	1.976	1.888	1.875	1.189	0.026	92	86	85	42	-95	6.61E-6	2.03E-5	4.70E-5
TK-10	0.961	2.055	1.950	1.930	1.967	1.684	0.036	90	89	92	66	-96	1.26E-5	2.55E-5	5.19E-5
UO-31	0.716	2.308	2.043	2.003	1.899	1.154	0.125	83	81	74	28	-83	3.30E-6	1.78E-5	5.06E-5
Prostate Cancer															
PC-3	0.618	2.569	2.479	2.449	2.392	1.051	0.071	95	94	91	22	-89	3.94E-6	1.59E-5	4.48E-5
DU-145	0.276	1.252	1.297	1.239	1.274	0.655	0.007	105	99	102	39	-97	6.66E-6	1.93E-5	4.48E-5
Breast Cancer															
MCF7	1.238	3.258	3.206	3.151	3.127	1.177	0.241	97	95	94	-5	-81	2.77E-6	8.90E-6	3.94E-5
MDA-MB-231/ATCC	0.549	1.234	1.257	1.191	1.237	0.853	0.031	103	94	100	44	-94	7.92E-6	2.09E-5	4.78E-5
HS 578T	1.446	2.466	2.367	2.289	2.290	1.771	0.520	90	83	83	32	-64	4.39E-6	2.15E-5	7.14E-5
BT-549	1.082	2.326	2.221	2.255	2.186	1.664	0.051	92	94	89	47	-95	8.38E-6	2.13E-5	4.80E-5
T-47D	0.545	1.466	1.413	1.405	1.362	0.800	0.168	94	93	89	28	-69	4.30E-6	1.93E-5	6.34E-5
MDA-MB-468	1.122	2.722	2.714	2.752	2.649	1.007	0.052	99	102	95	-10	-95	2.69E-6	8.00E-6	2.93E-5

Figure S67: five-dose graph of compound **17** (NSC 846158)

National Cancer Institute Developmental Therapeutics Program
In-Vitro Testing Results

NSC : D - 846158 / 1	Experiment ID : 2310NS93	Test Type : 08	Units : Molar
Report Date : September 01, 2025	Test Date : October 16, 2023	QNS :	MC :
COMI : 51ASA	Stain Reagent : SRB Dual-Pass Related	SSPL : 0J6G	

			Log10 Concentration												
	Time		Mean Optical Densities					Percent Growth							
Panel/Cell Line	Zero	Ctrl	-8.0	-7.0	-6.0	-5.0	-4.0	-8.0	-7.0	-6.0	-5.0	-4.0	GI50	TGI	LC50
Leukemia															
CCRF-CEM	0.490	2.748	2.743	2.628	2.519	1.612	0.588	100	95	90	50	4	9.83E-6	> 1.00E-4	> 1.00E-4
HL-60(TB)	0.608	2.576	2.416	2.504	2.232	1.612	0.425	92	96	83	51	-30	1.03E-5	4.25E-5	> 1.00E-4
K-562	0.271	2.329	2.345	2.297	2.067	0.616	0.223	101	98	87	17	-18	3.37E-6	3.04E-5	> 1.00E-4
MOLT-4	0.497	2.530	2.514	2.573	2.502	1.337	0.573	99	102	99	41	4	7.05E-6	> 1.00E-4	> 1.00E-4
RPMI-8226	0.670	2.649	2.614	2.620	2.473	1.290	0.575	98	99	91	31	-14	4.87E-6	4.88E-5	> 1.00E-4
SR	0.288	1.842	1.704	1.757	1.622	0.603	0.291	91	95	86	20	0	3.52E-6	> 1.00E-4	> 1.00E-4
Non-Small Cell Lung Cancer															
A549/ATCC	0.434	1.994	1.852	1.938	1.863	1.571	1.028	91	96	92	73	38	4.54E-5	> 1.00E-4	> 1.00E-4
EKVX	0.695	1.935	1.837	1.874	1.831	1.340	0.933	92	95	92	52	19	1.15E-5	> 1.00E-4	> 1.00E-4
HOP-62	0.592	2.006	1.962	1.939	1.855	1.551	1.365	97	95	89	68	55	> 1.00E-4	> 1.00E-4	> 1.00E-4
HOP-92	0.989	1.623	1.569	1.623	1.564	1.087	0.801	91	100	91	15	-19	3.47E-6	2.80E-5	> 1.00E-4
NCI-H226	1.181	2.626	2.490	2.543	2.427	1.969	1.695	91	94	86	54	36	1.72E-5	> 1.00E-4	> 1.00E-4
NCI-H23	0.593	2.399	2.341	2.351	2.280	1.809	0.879	97	97	93	67	16	2.17E-5	> 1.00E-4	> 1.00E-4
NCI-H322M	0.756	2.283	2.182	2.201	2.167	1.714	1.562	93	95	92	63	53	> 1.00E-4	> 1.00E-4	> 1.00E-4
NCI-H460	0.281	2.610	2.596	2.781	2.622	1.507	0.505	99	107	101	53	10	1.15E-5	> 1.00E-4	> 1.00E-4
NCI-H522	0.878	2.509	2.332	2.417	2.331	2.059	0.828	89	94	89	72	-6	1.93E-5	8.44E-5	> 1.00E-4
Colon Cancer															
COLO 205	0.397	2.057	2.126	2.149	1.934	0.882	0.067	104	106	93	29	-83	4.69E-6	1.82E-5	5.07E-5
HCC-2998	0.734	2.947	2.613	2.790	2.823	1.209	0.112	85	93	94	21	-85	4.06E-6	1.59E-5	4.71E-5
HCT-116	0.197	2.411	2.206	2.297	2.202	0.920	0.311	91	95	91	33	5	5.01E-6	> 1.00E-4	> 1.00E-4
HCT-15	0.621	2.701	2.656	2.592	2.592	1.669	0.792	98	95	95	50	8	1.02E-5	> 1.00E-4	> 1.00E-4
HT29	0.294	2.011	1.900	1.944	1.911	0.878	0.454	94	96	94	34	9	5.42E-6	> 1.00E-4	> 1.00E-4
KM12	0.618	2.914	2.919	2.972	2.752	2.094	0.918	100	103	93	64	13	1.90E-5	> 1.00E-4	> 1.00E-4
SW-620	0.274	2.068	1.963	1.955	1.829	1.397	0.254	94	94	87	63	-7	1.51E-5	7.86E-5	> 1.00E-4
CNS Cancer															
SF-268	0.814	2.605	2.359	2.479	2.424	2.001	1.436	86	93	90	66	35	3.28E-5	> 1.00E-4	> 1.00E-4
SF-295	0.925	2.735	2.660	2.475	2.423	1.712	0.818	96	86	83	43	-12	6.82E-6	6.16E-5	> 1.00E-4
SF-539	0.749	2.475	2.308	2.434	2.365	1.982	0.687	90	98	94	71	-8	1.86E-5	7.87E-5	> 1.00E-4
SNB-19	0.653	2.011	1.846	1.951	1.835	1.786	1.155	88	96	87	83	37	5.24E-5	> 1.00E-4	> 1.00E-4
SNB-75	1.119	2.041	1.958	1.928	1.919	1.666	1.350	91	88	87	59	25	1.86E-5	> 1.00E-4	> 1.00E-4
U251	0.418	1.646	1.541	1.550	1.583	1.218	0.825	91	92	95	65	33	2.97E-5	> 1.00E-4	> 1.00E-4
Melanoma															
LOX IMVI	0.317	2.489	2.350	2.402	2.247	1.602	0.049	94	96	89	59	-85	1.16E-5	2.58E-5	5.74E-5
MALME-3M	0.636	1.551	1.471	1.497	1.432	0.896	0.233	91	94	87	28	-63	4.28E-6	2.04E-5	7.15E-5
M14	0.360	1.675	1.672	1.693	1.589	0.905	0.323	100	101	93	41	-10	6.85E-6	6.33E-5	> 1.00E-4
MDA-MB-435	0.558	2.600	2.503	2.487	2.431	1.827	0.457	95	94	92	62	-18	1.42E-5	5.95E-5	> 1.00E-4
SK-MEL-2	1.711	3.001	2.940	2.959	2.948	2.765	0.697	95	97	96	82	-59	1.68E-5	3.80E-5	8.60E-5
SK-MEL-28	0.725	2.165	2.113	2.141	1.938	1.499	0.438	96	98	84	54	-40	1.10E-5	3.76E-5	> 1.00E-4
SK-MEL-5	0.822	3.162	3.084	3.112	3.055	1.528	0.003	97	98	95	30	-100	4.97E-6	1.71E-5	4.15E-5
UACC-257	1.052	2.284	2.186	2.235	2.203	1.733	0.599	92	96	93	55	-43	1.13E-5	3.65E-5	> 1.00E-4
UACC-62	0.769	2.752	2.446	2.436	2.404	1.823	0.708	85	84	82	53	-8	1.13E-5	7.41E-5	> 1.00E-4
Ovarian Cancer															
IGROV1	0.522	2.033	1.951	2.023	1.945	1.527	1.090	95	99	94	67	38	3.72E-5	> 1.00E-4	> 1.00E-4
OVCAR-3	0.582	1.954	1.939	1.933	1.951	1.474	0.585	99	98	100	65	0	1.70E-5	> 1.00E-4	> 1.00E-4
OVCAR-4	0.788	2.410	2.311	2.382	2.280	1.607	1.017	94	98	92	50	14	1.03E-5	> 1.00E-4	> 1.00E-4
OVCAR-5	0.509	1.373	1.298	1.302	1.243	1.022	0.880	91	92	85	59	43	3.72E-5	> 1.00E-4	> 1.00E-4
OVCAR-8	0.372	1.795	1.749	1.795	1.751	1.350	0.697	97	100	97	69	23	2.55E-5	> 1.00E-4	> 1.00E-4
NCI/ADR-RES	0.508	1.981	1.913	1.930	1.805	1.343	0.758	95	97	88	57	17	1.47E-5	> 1.00E-4	> 1.00E-4
SK-OV-3	0.648	1.809	1.715	1.737	1.676	1.498	1.231	92	94	89	73	50	> 1.00E-4	> 1.00E-4	> 1.00E-4
Renal Cancer															
786-0	0.607	2.760	2.637	2.698	2.635	1.934	1.163	94	97	94	62	26	2.11E-5	> 1.00E-4	> 1.00E-4
A498	1.468	2.417	2.275	2.366	2.284	2.223	1.793	85	95	86	80	34	4.49E-5	> 1.00E-4	> 1.00E-4
ACHN	0.435	2.062	1.899	1.955	1.853	1.427	0.749	90	93	87	61	19	1.83E-5	> 1.00E-4	> 1.00E-4
CAKI-1	1.050	2.711	2.611	2.608	2.550	1.987	1.172	94	94	90	56	7	1.35E-5	> 1.00E-4	> 1.00E-4
RXF 393	1.108	1.918	1.859	1.846	1.638	1.335	0.971	93	91	65	28	-12	2.57E-6	4.93E-5	> 1.00E-4
SN12C	0.516	2.108	2.079	1.955	1.909	1.342	0.848	98	90	88	52	21	1.15E-5	> 1.00E-4	> 1.00E-4
TK-10	0.961	2.055	1.931	1.950	2.099	1.748	1.206	89	90	104	72	22	2.77E-5	> 1.00E-4	> 1.00E-4
UO-31	0.716	2.308	1.987	2.016	1.969	1.200	0.946	80	82	79	30	14	3.93E-6	> 1.00E-4	> 1.00E-4
Prostate Cancer															
PC-3	0.618	2.569	2.505	2.465	2.322	1.312	0.777	97	95	87	36	8	5.26E-6	> 1.00E-4	> 1.00E-4
DU-145	0.276	1.252	1.242	1.303	1.194	1.003	0.774	99	105	94	75	51	> 1.00E-4	> 1.00E-4	> 1.00E-4
Breast Cancer															
MCF7	1.238	3.258	3.161	3.186	3.141	2.527	0.336	95	96	94	64	-73	1.26E-5	2.93E-5	6.80E-5
MDA-MB-231/ATCC	0.549	1.234	1.220	1.251	1.172	1.063	0.913	98	103	91	75	53	> 1.00E-4	> 1.00E-4	> 1.00E-4
HS 578T	1.446	2.466	2.277	2.324	2.295	1.935	1.545	81	86	83	48	10	8.72E-6	> 1.00E-4	> 1.00E-4
BT-549	1.082	2.326	2.200	2.264	2.200	1.675	0.920	90	95	90	48	-15	8.81E-6	5.76E-5	> 1.00E-4
T-47D	0.545	1.466	1.396	1.434	1.381	0.819	0.584	92	96	91	30	4	4.65E-6	> 1.00E-4	> 1.00E-4
MDA-MB-468	1.122	2.722	2.716	2.734	2.507	1.691	0.975	100	101	87	36	-13	5.21E-6	5.38E-5	> 1.00E-4

Figure S68: five-dose graph of compound **20** (NSC 846160)

National Cancer Institute Developmental Therapeutics Program
In-Vitro Testing Results

NSC : D - 846160 / 1	Experiment ID : 2310NS93	Test Type : 08	Units : Molar
Report Date : December 01, 2023	Test Date : October 16, 2023	QNS :	MC :
COMI : 62ASA	Stain Reagent : SRB Dual-Pass Related	SSPL : 0J6G	

			Log10 Concentration												
	Time		Mean Optical Densities					Percent Growth							
Panel/Cell Line	Zero	Ctrl	-8.0	-7.0	-6.0	-5.0	-4.0	-8.0	-7.0	-6.0	-5.0	-4.0	GI50	TGI	LC50
Leukemia															
CCRF-CEM	0.490	2.673	2.646	2.587	2.591	1.619	0.477	99	96	96	52	-3	1.07E-5	8.90E-5	> 1.00E-4
HL-60(TB)	0.608	2.794	2.587	2.577	2.691	1.286	0.387	91	90	95	31	-36	5.06E-6	2.88E-5	> 1.00E-4
K-562	0.271	2.335	2.296	2.221	2.110	0.403	0.251	98	94	89	6	-7	2.97E-6	2.91E-5	> 1.00E-4
MOLT-4	0.497	2.529	2.524	2.388	2.388	1.101	0.422	100	93	93	30	-15	4.78E-6	4.61E-5	> 1.00E-4
RPMI-8226	0.670	2.815	2.730	2.683	2.616	1.076	0.624	96	94	91	19	-7	3.69E-6	5.39E-5	> 1.00E-4
SR	0.288	1.815	1.740	1.631	0.832	0.564	0.337	95	88	36	18	3	5.31E-7	> 1.00E-4	> 1.00E-4
Non-Small Cell Lung Cancer															
A549/ATCC	0.434	2.018	1.995	1.967	2.011	1.527	0.077	99	97	100	69	-82	1.33E-5	2.86E-5	6.12E-5
EKVX	0.695	1.925	1.907	1.865	1.878	1.356	0.061	98	95	96	54	-91	1.06E-5	2.35E-5	5.19E-5
HOP-62	0.592	2.023	1.947	1.973	1.915	1.727	0.074	95	96	92	79	-88	1.50E-5	2.99E-5	5.95E-5
HOP-92	0.989	1.671	1.566	1.583	1.552	1.168	0.226	85	87	83	26	-77	3.78E-6	1.79E-5	5.46E-5
NCI-H226	1.181	2.627	2.561	2.512	2.509	2.064	0.397	95	92	92	61	-66	1.22E-5	3.01E-5	7.44E-5
NCI-H23	0.593	2.394	2.363	2.345	2.310	1.595	0.036	98	97	95	56	-94	1.09E-5	2.35E-5	5.08E-5
NCI-H322M	0.756	2.374	2.472	2.158	2.151	1.735	0.033	106	87	86	61	-96	1.17E-5	2.44E-5	5.10E-5
NCI-H460	0.281	2.658	2.730	2.779	2.739	1.347	0.061	103	105	103	45	-78	8.16E-6	2.31E-5	5.88E-5
NCI-H522	0.878	2.408	2.220	2.193	2.254	1.924	0.069	88	86	90	68	-92	1.30E-5	2.67E-5	5.46E-5
Colon Cancer															
COLO 205	0.397	2.095	2.140	2.116	2.114	0.102	0.075	103	101	101	-74	-81	1.96E-6	3.77E-6	7.27E-6
HCC-2998	0.734	2.844	2.707	2.692	2.704	0.142	0.046	94	93	93	-81	-94	1.77E-6	3.44E-6	6.66E-6
HCT-116	0.197	2.271	2.225	2.176	2.231	0.885	0.061	98	95	98	33	-69	5.50E-6	2.11E-5	6.48E-5
HCT-15	0.621	2.675	2.546	2.517	2.450	1.550	0.040	94	92	89	45	-94	7.78E-6	2.12E-5	4.85E-5
HT29	0.294	1.847	1.871	1.850	1.839	0.571	0.033	101	100	99	18	-89	4.03E-6	1.47E-5	4.32E-5
KM12	0.618	2.893	2.876	2.916	2.924	1.722	0.061	99	101	101	49	-90	9.38E-6	2.24E-5	5.14E-5
SW-620	0.274	2.080	2.078	2.043	2.046	0.482	0.077	100	98	98	12	-72	3.59E-6	1.37E-5	5.44E-5
CNS Cancer															
SF-268	0.814	2.569	2.488	2.557	2.431	1.883	0.129	95	99	92	61	-84	1.19E-5	2.63E-5	5.81E-5
SF-295	0.925	2.639	2.514	2.450	2.491	1.948	0.021	93	89	91	60	-98	1.15E-5	2.39E-5	4.97E-5
SF-539	0.749	2.450	2.432	2.361	2.407	1.660	0.015	99	95	97	54	-98	1.06E-5	2.26E-5	4.82E-5
SNB-19	0.653	2.035	1.939	1.883	1.955	1.743	0.016	93	89	94	79	-98	1.46E-5	2.80E-5	5.37E-5
SNB-75	1.119	2.020	2.051	2.044	2.035	1.817	0.095	103	103	102	77	-92	1.45E-5	2.87E-5	5.68E-5
U251	0.418	1.697	1.632	1.536	1.647	1.077	0.027	95	87	96	52	-94	1.02E-5	2.26E-5	5.00E-5
Melanoma															
LOX IMVI	0.317	2.526	2.447	2.357	2.387	0.869	0.010	96	92	94	25	-97	4.33E-6	1.60E-5	4.12E-5
MALME-3M	0.636	1.456	1.467	1.429	1.474	0.203	0.051	101	97	102	-68	-92	2.02E-6	3.98E-6	7.83E-6
M14	0.360	1.682	1.660	1.649	1.648	0.149	0.049	98	98	97	-59	-86	2.01E-6	4.21E-6	8.81E-6
MDA-MB-435	0.558	2.532	2.523	2.503	2.525	1.105	0.082	100	99	100	28	-85	4.90E-6	1.76E-5	4.87E-5
SK-MEL-2	1.711	2.863	2.819	2.790	2.829	2.553	0.076	96	94	97	73	-96	1.37E-5	2.71E-5	5.37E-5
SK-MEL-28	0.725	2.160	2.086	2.043	2.083	0.413	0.005	95	92	95	-43	-99	2.11E-6	4.87E-6	1.33E-5
SK-MEL-5	0.822	3.142	3.092	2.983	3.028	0.139	0.006	98	93	95	-83	-99	1.79E-6	3.42E-6	6.52E-6
UACC-257	1.052	2.340	2.274	2.248	2.017	0.878	0.065	95	93	75	-17	-94	1.87E-6	6.60E-6	2.71E-5
UACC-62	0.769	2.792	2.550	2.444	2.557	1.634	0.016	88	83	88	43	-98	6.93E-6	2.01E-5	4.56E-5
Ovarian Cancer															
IGROV1	0.522	2.056	2.117	2.108	1.912	1.325	0.073	104	103	91	52	-86	1.04E-5	2.39E-5	5.49E-5
OVCAR-3	0.582	1.956	2.039	1.974	1.983	1.462	0.017	106	101	102	64	-97	1.22E-5	2.50E-5	5.10E-5
OVCAR-4	0.788	2.354	2.361	2.344	2.310	1.638	0.115	100	99	97	54	-85	1.07E-5	2.45E-5	5.58E-5
OVCAR-5	0.509	1.331	1.289	1.236	1.265	1.037	0.006	95	88	92	64	-99	1.22E-5	2.47E-5	5.01E-5
OVCAR-8	0.372	1.919	1.882	1.766	1.884	1.431	0.099	98	90	98	68	-73	1.35E-5	3.04E-5	6.84E-5
NCI/ADR-RES	0.508	1.970	1.896	1.836	1.819	1.422	0.107	95	91	90	63	-79	1.23E-5	2.76E-5	6.24E-5
SK-OV-3	0.648	1.817	1.751	1.778	1.813	1.426	0.052	94	97	100	67	-92	1.27E-5	2.63E-5	5.43E-5
Renal Cancer															
786-0	0.607	2.783	2.790	2.607	2.778	1.842	0.117	100	92	100	57	-81	1.12E-5	2.59E-5	5.98E-5
A498	1.468	2.400	2.406	2.341	2.317	2.302	0.088	101	94	91	89	-94	1.64E-5	3.07E-5	5.75E-5
ACHN	0.435	2.057	2.058	1.963	2.010	1.403	0.002	100	94	97	60	-100	1.15E-5	2.37E-5	4.88E-5
CAKI-1	1.050	2.731	2.628	2.609	2.605	1.712	0.093	94	93	92	39	-91	6.30E-6	2.00E-5	4.84E-5
RXF 393	1.108	1.884	1.877	1.848	1.787	1.362	0.102	99	95	87	33	-91	4.83E-6	1.84E-5	4.67E-5
SN12C	0.516	2.164	2.019	1.921	1.959	1.484	0.031	91	85	88	59	-94	1.14E-5	2.42E-5	5.15E-5
TK-10	0.961	1.926	1.820	1.821	1.911	1.809	0.047	89	89	98	88	-95	1.61E-5	3.02E-5	5.66E-5
UO-31	0.716	2.114	1.948	1.839	1.846	1.096	0.067	88	80	81	27	-91	3.75E-6	1.70E-5	4.52E-5
Prostate Cancer															
PC-3	0.618	2.476	2.388	2.407	2.337	1.373	0.367	95	96	93	41	-41	6.59E-6	3.16E-5	> 1.00E-4
DU-145	0.276	1.268	1.278	1.286	1.308	0.984	0.044	101	102	104	71	-84	1.37E-5	2.88E-5	6.03E-5
Breast Cancer															
MCF7	1.238	3.258	3.183	3.154	3.106	2.200	0.169	96	95	92	48	-86	8.84E-6	2.27E-5	5.35E-5
MDA-MB-231/ATCC	0.549	1.248	1.239	1.260	1.247	1.053	0.043	99	102	100	72	-92	1.36E-5	2.75E-5	5.53E-5
HS 578T	1.446	2.461	2.373	2.294	2.318	1.789	0.734	91	83	86	34	-49	4.88E-6	2.55E-5	> 1.00E-4
BT-549	1.082	2.305	2.231	2.167	2.220	1.671	0.107	94	89	93	48	-90	9.08E-6	2.23E-5	5.13E-5
T-47D	0.545	1.489	1.431	1.409	1.384	0.823	0.208	94	92	89	29	-62	4.50E-6	2.10E-5	7.42E-5
MDA-MB-468	1.122	2.599	2.625	2.537	2.483	1.557	0.138	102	96	92	29	-88	4.70E-6	1.78E-5	4.77E-5

Figure S69: waterfall graph GI_{50} of compound **1** (NSC 846162)

National Cancer Institute Developmental Therapeutics Program Waterfall Graph GI50	NSC : D - 846162/1	Units :Molar	SSPL :0J6G	EXP. ID :2310NS93
	Report Date :December 01, 2023		Test Date :October 16, 2023	

Panel	Cell Name	Hollow Fiber	GI50
Melanoma	MALME-3M		-5.81
Melanoma	SK-MEL-5		-5.78
Colon Cancer	SW-620	*	-5.76
Melanoma	MDA-MB-435	*	-5.76
Melanoma	LOX IMVI	*	-5.75
Leukemia	K-562		-5.75
Colon Cancer	COLO 205	*	-5.75
Colon Cancer	HT29		-5.73
Melanoma	UACC-62	*	-5.73
Melanoma	SK-MEL-28		-5.73
Melanoma	UACC-257		-5.73
CNS Cancer	SF-295	*	-5.72
Melanoma	M14		-5.69
Non-Small Cell Lung Cancer	NCI-H460		-5.69
Leukemia	RPMI-8226		-5.64
Leukemia	SR		-5.64
Colon Cancer	HCT-15		-5.64
Leukemia	HL-60(TB)		-5.58
Breast Cancer	MDA-MB-468		-5.57
Renal Cancer	RXF 393		-5.57
Renal Cancer	CAKI-1		-5.57
Colon Cancer	HCC-2998		-5.53
Colon Cancer	KM12		-5.52
Colon Cancer	HCT-116		-5.51
Non-Small Cell Lung Cancer	HOP-92		-5.50
Renal Cancer	786-0		-5.46
CNS Cancer	SNB-75		-5.46
Leukemia	MOLT-4		-5.45
Breast Cancer	T-47D		-5.45
Renal Cancer	UO-31		-5.43
Breast Cancer	HS 578T		-5.39
CNS Cancer	U251	*	-5.38
CNS Cancer	SF-539		-5.37
Breast Cancer	MCF7		-5.37
Ovarian Cancer	IGROV1		-5.35
Renal Cancer	TK-10		-5.30
Ovarian Cancer	OVCAR-5	*	-5.29
Breast Cancer	MDA-MB-231/ATCC	*	-5.29
Leukemia	CCRF-CEM		-5.24
Non-Small Cell Lung Cancer	A549/ATCC		-5.24
Prostate Cancer	DU-145		-5.14
Ovarian Cancer	OVCAR-3	*	-5.10
CNS Cancer	SF-268		-5.06
Prostate Cancer	PC-3		-5.05
Breast Cancer	BT-549		-5.05
Non-Small Cell Lung Cancer	HOP-62		-5.03
Melanoma	SK-MEL-2		-5.03
Renal Cancer	ACHN		-5.02
Non-Small Cell Lung Cancer	NCI-H23	*	-4.99
Non-Small Cell Lung Cancer	EKVX		-4.97
Non-Small Cell Lung Cancer	NCI-H226		-4.97
Renal Cancer	SN12C		-4.96
Non-Small Cell Lung Cancer	NCI-H522	*	-4.96
Ovarian Cancer	NCI/ADR-RES		-4.95
Non-Small Cell Lung Cancer	NCI-H322M		-4.95
CNS Cancer	SNB-19		-4.94
Ovarian Cancer	SK-OV-3		-4.94
Renal Cancer	A498		-4.93
Ovarian Cancer	OVCAR-8		-4.92
Ovarian Cancer	OVCAR-4		-4.92

Log10 High Conc :-4.0

Figure S70: waterfall graph TGI of compound **1** (NSC 846162)

National Cancer Institute Developmental Therapeutics Program Waterfall Graph TGI	NSC : D - 846162/1	Units :Molar	SSPL :0J6G	EXP. ID :2310NS93
	Report Date :December 01, 2023		Test Date :October 16, 2023	

Panel	Cell Name	Hollow Fiber	TGI
Melanoma	MALME-3M		-5.53
Melanoma	SK-MEL-5		-5.51
Melanoma	LOX IMVI	*	-5.47
Melanoma	MDA-MB-435	*	-5.45
Colon Cancer	COLO 205	*	-5.44
Melanoma	SK-MEL-28		-5.43
Melanoma	UACC-62	*	-5.40
Melanoma	UACC-257		-5.40
CNS Cancer	SF-295	*	-5.40
Colon Cancer	SW-620	*	-5.39
Melanoma	M14		-5.36
Non-Small Cell Lung Cancer	NCI-H460		-5.36
Colon Cancer	HT29		-5.35
Leukemia	K-562		-5.33
Colon Cancer	HCT-15		-5.19
Leukemia	RPMI-8226		-5.14
Breast Cancer	MDA-MB-468		-5.03
Renal Cancer	CAKI-1		-5.00
Colon Cancer	KM12		-4.99
Colon Cancer	HCC-2998		-4.97
Leukemia	HL-60(TB)		-4.95
Renal Cancer	RXF 393		-4.92
Leukemia	SR		-4.90
Renal Cancer	786-0		-4.90
Colon Cancer	HCT-116		-4.86
Leukemia	MOLT-4		-4.84
CNS Cancer	SF-539		-4.80
CNS Cancer	SNB-75		-4.78
CNS Cancer	U251	*	-4.78
Renal Cancer	UO-31		-4.78
Non-Small Cell Lung Cancer	HOP-92		-4.76
Breast Cancer	T-47D		-4.75
Breast Cancer	MDA-MB-231/ATCC	*	-4.75
Ovarian Cancer	OVCAR-5	*	-4.75
Ovarian Cancer	IGROV1		-4.75
Breast Cancer	MCF7		-4.74
Renal Cancer	TK-10		-4.73
Prostate Cancer	DU-145		-4.70
Non-Small Cell Lung Cancer	A549/ATCC		-4.69
Ovarian Cancer	OVCAR-3	*	-4.67
Renal Cancer	ACHN		-4.67
Melanoma	SK-MEL-2		-4.67
Breast Cancer	BT-549		-4.66
Non-Small Cell Lung Cancer	NCI-H23	*	-4.65
CNS Cancer	SF-268		-4.64
Non-Small Cell Lung Cancer	EKVX		-4.63
Non-Small Cell Lung Cancer	HOP-62		-4.63
Breast Cancer	HS 578T		-4.63
Renal Cancer	SN12C		-4.63
Non-Small Cell Lung Cancer	NCI-H522	*	-4.63
Non-Small Cell Lung Cancer	NCI-H322M		-4.62
CNS Cancer	SNB-19		-4.61
Prostate Cancer	PC-3		-4.60
Ovarian Cancer	SK-OV-3		-4.60
Renal Cancer	A498		-4.59
Ovarian Cancer	NCI/ADR-RES		-4.59
Ovarian Cancer	OVCAR-4		-4.58
Leukemia	CCRF-CEM		-4.57
Ovarian Cancer	OVCAR-8		-4.56
Non-Small Cell Lung Cancer	NCI-H226		-4.55

Log10 High Conc : -4.0

Figure S71: waterfall graph LC_{50} of compound **1** (NSC 846162)

National Cancer Institute Developmental Therapeutics Program Waterfall Graph LC50	NSC : D - 846162/1	Units :Molar	SSPL :0J6G	EXP. ID :2310NS93
	Report Date :December 01, 2023		Test Date :October 16, 2023	

Panel	Cell Name	Hollow Fiber	LC50
Melanoma	SK-MEL-5		-5.25
Melanoma	MALME-3M		-5.25
Melanoma	LOX IMVI	*	-5.20
Melanoma	MDA-MB-435	*	-5.15
Colon Cancer	COLO 205	*	-5.14
Melanoma	SK-MEL-28		-5.14
Melanoma	UACC-62	*	-5.08
Melanoma	UACC-257		-5.07
CNS Cancer	SF-295	*	-5.07
Non-Small Cell Lung Cancer	NCI-H460		-5.04
Melanoma	M14		-5.04
Colon Cancer	SW-620	*	-5.02
Colon Cancer	HT29		-4.92
Colon Cancer	HCT-15		-4.62
Leukemia	K-562		-4.61
Renal Cancer	CAKI-1		-4.48
Breast Cancer	MDA-MB-468		-4.47
Colon Cancer	KM12		-4.45
Colon Cancer	HCC-2998		-4.45
CNS Cancer	SF-539		-4.39
Renal Cancer	RXF 393		-4.38
CNS Cancer	SNB-75		-4.38
Ovarian Cancer	OVCAR-5	*	-4.37
Renal Cancer	UO-31		-4.37
CNS Cancer	U251	*	-4.37
Renal Cancer	TK-10		-4.36
Breast Cancer	MDA-MB-231/ATCC	*	-4.35
Prostate Cancer	DU-145		-4.34
Renal Cancer	786-0		-4.34
Renal Cancer	ACHN		-4.34
Leukemia	HL-60(TB)		-4.33
Melanoma	SK-MEL-2		-4.33
Ovarian Cancer	OVCAR-3	*	-4.31
Ovarian Cancer	IGROV1		-4.31
Non-Small Cell Lung Cancer	NCI-H23	*	-4.31
Breast Cancer	BT-549		-4.30
Renal Cancer	SN12C		-4.30
Non-Small Cell Lung Cancer	NCI-H322M		-4.30
Non-Small Cell Lung Cancer	EKVX		-4.30
Leukemia	MOLT-4		-4.30
Non-Small Cell Lung Cancer	NCI-H522	*	-4.29
Non-Small Cell Lung Cancer	HOP-92		-4.29
CNS Cancer	SNB-19		-4.29
Non-Small Cell Lung Cancer	A549/ATCC		-4.29
Breast Cancer	MCF7		-4.28
Ovarian Cancer	SK-OV-3		-4.27
CNS Cancer	SF-268		-4.27
Renal Cancer	A498		-4.26
Non-Small Cell Lung Cancer	HOP-62		-4.26
Ovarian Cancer	OVCAR-4		-4.25
Breast Cancer	T-47D		-4.24
Ovarian Cancer	NCI/ADR-RES		-4.23
Ovarian Cancer	OVCAR-8		-4.21
Prostate Cancer	PC-3		-4.19
Colon Cancer	HCT-116		-4.16
Leukemia	RPMI-8226		-4.15
Non-Small Cell Lung Cancer	NCI-H226		-4.12
Leukemia	SR		-4.03
Leukemia	CCRF-CEM		> -4.00
Breast Cancer	HS 578T		> -4.00

Log10 High Conc : -4.0

Figure S72: waterfall graph GI50 of compound **5** (NSC 846166)

National Cancer Institute Developmental Therapeutics Program<br>Waterfall Graph GI50	NSC : D - 846166/1	Units :Molar	SSPL :1CYF	EXP. ID :2310NS93
	Report Date :November 29, 2023		Test Date :October 16, 2023	

Panel	Cell Name	Hollow Fiber	GI50
Leukemia	K-562		-5.36
Leukemia	HL-60(TB)		-5.34
Prostate Cancer	PC-3		-5.33
Leukemia	MOLT-4		-5.30
Leukemia	RPMI-8226		-5.29
Colon Cancer	HCT-116		-5.29
Colon Cancer	COLO 205	*	-5.28
Colon Cancer	HT29		-5.25
Leukemia	SR		-5.12
Renal Cancer	RXF 393		-5.07
Melanoma	SK-MEL-5		-5.06
CNS Cancer	SF-295	*	-5.03
Renal Cancer	SN12C		-4.81
Non-Small Cell Lung Cancer	A549/ATCC		> -4.00
Non-Small Cell Lung Cancer	EKVX		> -4.00
Non-Small Cell Lung Cancer	HOP-62		> -4.00
Non-Small Cell Lung Cancer	NCI-H226		> -4.00
Non-Small Cell Lung Cancer	NCI-H23	*	> -4.00
Non-Small Cell Lung Cancer	NCI-H322M		> -4.00
Non-Small Cell Lung Cancer	NCI-H522	*	> -4.00
Colon Cancer	HCC-2998		> -4.00
Colon Cancer	KM12		> -4.00
Colon Cancer	SW-620	*	> -4.00
CNS Cancer	SF-268		> -4.00
CNS Cancer	SF-539		> -4.00
CNS Cancer	SNB-19		> -4.00
CNS Cancer	SNB-75		> -4.00
CNS Cancer	U251	*	> -4.00
Melanoma	LOX IMVI	*	> -4.00
Melanoma	MALME-3M		> -4.00
Melanoma	MDA-MB-435	*	> -4.00
Melanoma	SK-MEL-2		> -4.00
Melanoma	SK-MEL-28		> -4.00
Melanoma	UACC-257		> -4.00
Melanoma	UACC-62	*	> -4.00
Ovarian Cancer	IGROV1		> -4.00
Ovarian Cancer	OVCAR-3	*	> -4.00
Ovarian Cancer	OVCAR-4		> -4.00
Ovarian Cancer	OVCAR-5	*	> -4.00
Ovarian Cancer	OVCAR-8		> -4.00
Ovarian Cancer	NCI/ADR-RES		> -4.00
Ovarian Cancer	SK-OV-3		> -4.00
Renal Cancer	786-0		> -4.00
Renal Cancer	A498		> -4.00
Renal Cancer	ACHN		> -4.00
Renal Cancer	CAKI-1		> -4.00
Renal Cancer	TK-10		> -4.00
Prostate Cancer	DU-145		> -4.00
Breast Cancer	MCF7		> -4.00
Breast Cancer	MDA-MB-231/ATCC	*	> -4.00
Breast Cancer	HS 578T		> -4.00
Breast Cancer	BT-549		> -4.00

Log10 High Conc :-4.0

Figure S73: waterfall graph TGI of compound **5** (NSC 846166)

National Cancer Institute Developmental Therapeutics Program<br>Waterfall Graph TGI	NSC : D - 846166/1	Units :Molar	SSPL :1CYF	EXP. ID :2310NS93
	Report Date :November 29, 2023		Test Date :October 16, 2023	

Panel	Cell Name	Hollow Fiber	TGI
Leukemia	CCRF-CEM		> -4.00
Leukemia	HL-60(TB)		> -4.00
Leukemia	K-562		> -4.00
Leukemia	MOLT-4		> -4.00
Leukemia	RPMI-8226		> -4.00
Leukemia	SR		> -4.00
Non-Small Cell Lung Cancer	A549/ATCC		> -4.00
Non-Small Cell Lung Cancer	EKVX		> -4.00
Non-Small Cell Lung Cancer	HOP-62		> -4.00
Non-Small Cell Lung Cancer	HOP-92		> -4.00
Non-Small Cell Lung Cancer	NCI-H226		> -4.00
Non-Small Cell Lung Cancer	NCI-H23	*	> -4.00
Non-Small Cell Lung Cancer	NCI-H322M		> -4.00
Non-Small Cell Lung Cancer	NCI-H460		> -4.00
Non-Small Cell Lung Cancer	NCI-H522	*	> -4.00
Colon Cancer	COLO 205	*	> -4.00
Colon Cancer	HCC-2998		> -4.00
Colon Cancer	HCT-116		> -4.00
Colon Cancer	HCT-15		> -4.00
Colon Cancer	HT29		> -4.00
Colon Cancer	KM12		> -4.00
Colon Cancer	SW-620	*	> -4.00
CNS Cancer	SF-268		> -4.00
CNS Cancer	SF-295	*	> -4.00
CNS Cancer	SF-539		> -4.00
CNS Cancer	SNB-19		> -4.00
CNS Cancer	SNB-75		> -4.00
CNS Cancer	U251	*	> -4.00
Melanoma	LOX IMVI	*	> -4.00
Melanoma	MALME-3M		> -4.00
Melanoma	M14		> -4.00
Melanoma	MDA-MB-435	*	> -4.00
Melanoma	SK-MEL-2		> -4.00
Melanoma	SK-MEL-28		> -4.00
Melanoma	SK-MEL-5		> -4.00
Melanoma	UACC-257		> -4.00
Melanoma	UACC-62	*	> -4.00
Ovarian Cancer	IGROV1		> -4.00
Ovarian Cancer	OVCAR-3	*	> -4.00
Ovarian Cancer	OVCAR-4		> -4.00
Ovarian Cancer	OVCAR-5	*	> -4.00
Ovarian Cancer	OVCAR-8		> -4.00
Ovarian Cancer	NCI/ADR-RES		> -4.00
Ovarian Cancer	SK-OV-3		> -4.00
Renal Cancer	786-0		> -4.00
Renal Cancer	A498		> -4.00
Renal Cancer	ACHN		> -4.00
Renal Cancer	CAKI-1		> -4.00
Renal Cancer	RXF 393		> -4.00
Renal Cancer	SN12C		> -4.00
Renal Cancer	TK-10		> -4.00
Renal Cancer	UO-31		> -4.00
Prostate Cancer	PC-3		> -4.00
Prostate Cancer	DU-145		> -4.00
Breast Cancer	MCF7		> -4.00
Breast Cancer	MDA-MB-231/ATCC	*	> -4.00
Breast Cancer	HS 578T		> -4.00
Breast Cancer	BT-549		> -4.00
Breast Cancer	T-47D		> -4.00
Breast Cancer	MDA-MB-468		> -4.00

Log10 High Conc : -4.0

Figure S74: waterfall graph LC_{50} of compound **5** (NSC 846166)

National Cancer Institute Developmental Therapeutics Program<br>Waterfall Graph LC50	NSC : D - 846166/1	Units :Molar	SSPL :1CYF	EXP. ID :2310NS93
	Report Date :November 29, 2023		Test Date :October 16, 2023	

Panel	Cell Name	Hollow Fiber	LC50
Leukemia	CCRF-CEM		> -4.00
Leukemia	HL-60(TB)		> -4.00
Leukemia	K-562		> -4.00
Leukemia	MOLT-4		> -4.00
Leukemia	RPMI-8226		> -4.00
Leukemia	SR		> -4.00
Non-Small Cell Lung Cancer	A549/ATCC		> -4.00
Non-Small Cell Lung Cancer	EKVX		> -4.00
Non-Small Cell Lung Cancer	HOP-62		> -4.00
Non-Small Cell Lung Cancer	HOP-92		> -4.00
Non-Small Cell Lung Cancer	NCI-H226		> -4.00
Non-Small Cell Lung Cancer	NCI-H23	*	> -4.00
Non-Small Cell Lung Cancer	NCI-H322M		> -4.00
Non-Small Cell Lung Cancer	NCI-H460		> -4.00
Non-Small Cell Lung Cancer	NCI-H522	*	> -4.00
Colon Cancer	COLO 205	*	> -4.00
Colon Cancer	HCC-2998		> -4.00
Colon Cancer	HCT-116		> -4.00
Colon Cancer	HCT-15		> -4.00
Colon Cancer	HT29		> -4.00
Colon Cancer	KM12		> -4.00
Colon Cancer	SW-620	*	> -4.00
CNS Cancer	SF-268		> -4.00
CNS Cancer	SF-295	*	> -4.00
CNS Cancer	SF-539		> -4.00
CNS Cancer	SNB-19		> -4.00
CNS Cancer	SNB-75		> -4.00
CNS Cancer	U251	*	> -4.00
Melanoma	LOX IMVI	*	> -4.00
Melanoma	MALME-3M		> -4.00
Melanoma	M14		> -4.00
Melanoma	MDA-MB-435	*	> -4.00
Melanoma	SK-MEL-2		> -4.00
Melanoma	SK-MEL-28		> -4.00
Melanoma	SK-MEL-5		> -4.00
Melanoma	UACC-257		> -4.00
Melanoma	UACC-62	*	> -4.00
Ovarian Cancer	IGROV1		> -4.00
Ovarian Cancer	OVCAR-3	*	> -4.00
Ovarian Cancer	OVCAR-4		> -4.00
Ovarian Cancer	OVCAR-5	*	> -4.00
Ovarian Cancer	OVCAR-8		> -4.00
Ovarian Cancer	NCI/ADR-RES		> -4.00
Ovarian Cancer	SK-OV-3		> -4.00
Renal Cancer	786-0		> -4.00
Renal Cancer	A498		> -4.00
Renal Cancer	ACHN		> -4.00
Renal Cancer	CAKI-1		> -4.00
Renal Cancer	RXF 393		> -4.00
Renal Cancer	SN12C		> -4.00
Renal Cancer	TK-10		> -4.00
Renal Cancer	UO-31		> -4.00
Prostate Cancer	PC-3		> -4.00
Prostate Cancer	DU-145		> -4.00
Breast Cancer	MCF7		> -4.00
Breast Cancer	MDA-MB-231/ATCC	*	> -4.00
Breast Cancer	HS 578T		> -4.00
Breast Cancer	BT-549		> -4.00
Breast Cancer	T-47D		> -4.00
Breast Cancer	MDA-MB-468		> -4.00

Log10 High Conc : -4.0

Figure S75: waterfall graph GI_{50} of compound **10** (NSC 846171)

National Cancer Institute Developmental Therapeutics Program Waterfall Graph GI50	NSC : D - 846171/1	Units :Molar	SSPL :1CYF	EXP. ID :2310NS93
	Report Date :November 29, 2023		Test Date :October 16, 2023	

Panel	Cell Name	Hollow Fiber	GI50
Melanoma	MALME-3M		-5.78
Colon Cancer	COLO 205	*	-5.72
Renal Cancer	RXF 393		-5.69
Melanoma	UACC-257		-5.64
Leukemia	SR		-5.64
Colon Cancer	HCC-2998		-5.60
Melanoma	SK-MEL-28		-5.60
Leukemia	K-562		-5.59
Melanoma	SK-MEL-5		-5.57
Melanoma	M14		-5.56
Breast Cancer	MDA-MB-468		-5.53
Leukemia	RPMI-8226		-5.52
Colon Cancer	HT29		-5.50
Non-Small Cell Lung Cancer	HOP-92		-5.46
Melanoma	LOX IMVI	*	-5.44
Renal Cancer	UO-31		-5.39
Non-Small Cell Lung Cancer	NCI-H460		-5.39
Prostate Cancer	PC-3		-5.39
Colon Cancer	HCT-15		-5.33
Colon Cancer	HCT-116		-5.32
Colon Cancer	KM12		-5.31
Leukemia	MOLT-4		-5.30
Leukemia	HL-60(TB)		-5.25
CNS Cancer	SF-295	*	-5.24
Renal Cancer	CAKI-1		-5.19
CNS Cancer	U251	*	-5.12
Ovarian Cancer	IGROV1		-5.11
Non-Small Cell Lung Cancer	NCI-H226		-5.11
Melanoma	MDA-MB-435	*	-5.10
Leukemia	CCRF-CEM		-5.09
Non-Small Cell Lung Cancer	EKVX		-5.06
Colon Cancer	SW-620	*	-5.05
Ovarian Cancer	OVCAR-3	*	-5.05
Breast Cancer	T-47D		-5.05
Melanoma	UACC-62	*	-5.04
Ovarian Cancer	OVCAR-5	*	-5.04
Renal Cancer	SN12C		-5.03
CNS Cancer	SNB-75		-5.02
Renal Cancer	ACHN		-5.01
Ovarian Cancer	OVCAR-4		-5.01
Breast Cancer	BT-549		-5.01
Breast Cancer	MCF7		-5.00
Ovarian Cancer	NCI/ADR-RES		-4.99
CNS Cancer	SF-539		-4.99
Prostate Cancer	DU-145		-4.98
Breast Cancer	HS 578T		-4.96
Breast Cancer	MDA-MB-231/ATCC	*	-4.95
Non-Small Cell Lung Cancer	A549/ATCC		-4.94
Ovarian Cancer	OVCAR-8		-4.92
Non-Small Cell Lung Cancer	NCI-H522	*	-4.91
Non-Small Cell Lung Cancer	NCI-H322M		-4.90
Renal Cancer	786-0		-4.89
Non-Small Cell Lung Cancer	NCI-H23	*	-4.88
Melanoma	SK-MEL-2		-4.83
Renal Cancer	TK-10		-4.80
Non-Small Cell Lung Cancer	HOP-62		-4.62
Renal Cancer	A498		-4.47
Ovarian Cancer	SK-OV-3		-4.29
CNS Cancer	SNB-19		-4.19
CNS Cancer	SF-268		> -4.07

Log10 High Conc :-4.1

Figure S76: waterfall graph TGI of compound **10** (NSC 846171)

National Cancer Institute Developmental Therapeutics Program Waterfall Graph TGI	NSC : D - 846171/1	Units :Molar	SSPL :1CYF	EXP. ID :2310NS93
	Report Date :November 29, 2023		Test Date :October 16, 2023	

Panel	Cell Name	Hollow Fiber	TGI
Melanoma	MALME-3M		-5.36
Colon Cancer	COLO 205	*	-5.29
Melanoma	UACC-257		-5.18
Renal Cancer	RXF 393		-5.10
Melanoma	M14		-5.07
Melanoma	SK-MEL-28		-5.07
Colon Cancer	HCC-2998		-5.02
Melanoma	SK-MEL-5		-5.01
Leukemia	SR		-4.90
Colon Cancer	HT29		-4.90
Breast Cancer	MDA-MB-468		-4.90
Leukemia	RPMI-8226		-4.81
Colon Cancer	KM12		-4.76
Non-Small Cell Lung Cancer	NCI-H460		-4.75
Melanoma	MDA-MB-435	*	-4.73
Melanoma	LOX IMVI	*	-4.71
CNS Cancer	SF-295	*	-4.71
Renal Cancer	SN12C		-4.69
Breast Cancer	MCF7		-4.66
Leukemia	K-562		-4.63
Colon Cancer	SW-620	*	-4.63
Breast Cancer	BT-549		-4.62
Ovarian Cancer	OVCAR-5	*	-4.61
Prostate Cancer	DU-145		-4.61
Melanoma	UACC-62	*	-4.60
Breast Cancer	MDA-MB-231/ATCC	*	-4.58
Non-Small Cell Lung Cancer	NCI-H522	*	-4.57
Ovarian Cancer	OVCAR-3	*	-4.56
Leukemia	HL-60(TB)		-4.51
Prostate Cancer	PC-3		-4.51
CNS Cancer	SF-539		-4.50
Colon Cancer	HCT-15		-4.49
Ovarian Cancer	NCI/ADR-RES		-4.47
Colon Cancer	HCT-116		-4.47
Ovarian Cancer	OVCAR-8		-4.47
Melanoma	SK-MEL-2		-4.46
Breast Cancer	T-47D		-4.45
Renal Cancer	ACHN		-4.43
Leukemia	MOLT-4		-4.40
CNS Cancer	SNB-75		-4.38
Non-Small Cell Lung Cancer	EKVX		-4.26
Renal Cancer	TK-10		-4.23
Non-Small Cell Lung Cancer	HOP-92		-4.23
Non-Small Cell Lung Cancer	NCI-H226		-4.22
Renal Cancer	CAKI-1		-4.17
Leukemia	CCRF-CEM		-4.14
Non-Small Cell Lung Cancer	NCI-H23	*	-4.10
Ovarian Cancer	OVCAR-4		-4.07
Non-Small Cell Lung Cancer	A549/ATCC		> -4.07
Non-Small Cell Lung Cancer	HOP-62		> -4.07
Non-Small Cell Lung Cancer	NCI-H322M		> -4.07
CNS Cancer	SF-268		> -4.07
CNS Cancer	SNB-19		> -4.07
CNS Cancer	U251	*	> -4.07
Ovarian Cancer	IGROV1		> -4.07
Ovarian Cancer	SK-OV-3		> -4.07
Renal Cancer	786-0		> -4.07
Renal Cancer	A498		> -4.07
Renal Cancer	UO-31		> -4.07
Breast Cancer	HS 578T		> -4.07

Log10 High Conc : -4.1

Figure S77: waterfall graph LC_{50} of compound **10** (NSC 846171)

National Cancer Institute Developmental Therapeutics Program<br>Waterfall Graph LC50	NSC : D - 846171/1	Units :Molar	SSPL :1CYF	EXP. ID :2310NS93
	Report Date :November 29, 2023		Test Date :October 16, 2023	

Panel	Cell Name	Hollow Fiber	LC50
Melanoma	MALME-3M		-4.81
Melanoma	UACC-257		-4.54
Melanoma	SK-MEL-5		-4.53
Melanoma	SK-MEL-28		-4.53
Breast Cancer	MDA-MB-468		-4.40
Melanoma	MDA-MB-435	*	-4.39
Colon Cancer	COLO 205	*	-4.36
Melanoma	M14		-4.35
Renal Cancer	SN12C		-4.35
Breast Cancer	MCF7		-4.33
Colon Cancer	KM12		-4.33
Colon Cancer	HT29		-4.32
CNS Cancer	SF-295	*	-4.30
Colon Cancer	HCC-2998		-4.25
Breast Cancer	BT-549		-4.24
Prostate Cancer	DU-145		-4.23
Non-Small Cell Lung Cancer	NCI-H522	*	-4.23
Breast Cancer	MDA-MB-231/ATCC	*	-4.21
Colon Cancer	SW-620	*	-4.20
Ovarian Cancer	OVCAR-5	*	-4.18
Melanoma	UACC-62	*	-4.16
Non-Small Cell Lung Cancer	NCI-H460		-4.13
Melanoma	SK-MEL-2		-4.09
Leukemia	CCRF-CEM		> -4.07
Leukemia	HL-60(TB)		> -4.07
Leukemia	K-562		> -4.07
Leukemia	MOLT-4		> -4.07
Leukemia	RPMI-8226		> -4.07
Leukemia	SR		> -4.07
Non-Small Cell Lung Cancer	A549/ATCC		> -4.07
Non-Small Cell Lung Cancer	EKVX		> -4.07
Non-Small Cell Lung Cancer	HOP-62		> -4.07
Non-Small Cell Lung Cancer	HOP-92		> -4.07
Non-Small Cell Lung Cancer	NCI-H226		> -4.07
Non-Small Cell Lung Cancer	NCI-H23	*	> -4.07
Non-Small Cell Lung Cancer	NCI-H322M		> -4.07
Colon Cancer	HCT-116		> -4.07
Colon Cancer	HCT-15		> -4.07
CNS Cancer	SF-268		> -4.07
CNS Cancer	SF-539		> -4.07
CNS Cancer	SNB-19		> -4.07
CNS Cancer	SNB-75		> -4.07
CNS Cancer	U251	*	> -4.07
Melanoma	LOX IMVI	*	> -4.07
Ovarian Cancer	IGROV1		> -4.07
Ovarian Cancer	OVCAR-3	*	> -4.07
Ovarian Cancer	OVCAR-4		> -4.07
Ovarian Cancer	OVCAR-8		> -4.07
Ovarian Cancer	NCI/ADR-RES		> -4.07
Ovarian Cancer	SK-OV-3		> -4.07
Renal Cancer	786-0		> -4.07
Renal Cancer	A498		> -4.07
Renal Cancer	ACHN		> -4.07
Renal Cancer	CAKI-1		> -4.07
Renal Cancer	RXF 393		> -4.07
Renal Cancer	TK-10		> -4.07
Renal Cancer	UO-31		> -4.07
Prostate Cancer	PC-3		> -4.07
Breast Cancer	HS 578T		> -4.07
Breast Cancer	T-47D		> -4.07

Log10 High Conc : -4.1

Figure S78: waterfall graph GI50 of compound **11** (NSC 846172)

National Cancer Institute Developmental Therapeutics Program Waterfall Graph GI50	NSC : D - 846172/1	Units :Molar	SSPL :1CYF	EXP. ID :2310NS93
	Report Date :November 29, 2023		Test Date :October 16, 2023	

Panel	Cell Name	Hollow Fiber	GI50
Colon Cancer	COLO 205	*	-5.78
Melanoma	SK-MEL-5		-5.77
Colon Cancer	HCC-2998		-5.76
Melanoma	UACC-257		-5.76
Melanoma	SK-MEL-28		-5.76
Melanoma	M14		-5.74
Melanoma	MALME-3M		-5.72
Renal Cancer	RXF 393		-5.71
CNS Cancer	SF-295	*	-5.69
Melanoma	LOX IMVI	*	-5.67
Leukemia	RPMI-8226		-5.66
Leukemia	SR		-5.60
Melanoma	UACC-62	*	-5.59
Leukemia	K-562		-5.59
Melanoma	MDA-MB-435	*	-5.59
Colon Cancer	HT29		-5.58
Breast Cancer	MDA-MB-468		-5.57
Leukemia	MOLT-4		-5.57
Non-Small Cell Lung Cancer	HOP-92		-5.56
Breast Cancer	MCF7		-5.56
Leukemia	HL-60(TB)		-5.53
Renal Cancer	UO-31		-5.48
Colon Cancer	HCT-116		-5.47
CNS Cancer	U251	*	-5.45
Non-Small Cell Lung Cancer	NCI-H460		-5.45
Colon Cancer	SW-620	*	-5.45
Colon Cancer	HCT-15		-5.44
Renal Cancer	CAKI-1		-5.43
Leukemia	CCRF-CEM		-5.41
Prostate Cancer	PC-3		-5.40
Colon Cancer	KM12		-5.39
Breast Cancer	T-47D		-5.37
CNS Cancer	SF-539		-5.36
Breast Cancer	HS 578T		-5.36
CNS Cancer	SF-268		-5.22
Ovarian Cancer	OVCAR-4		-5.22
Non-Small Cell Lung Cancer	EKVX		-5.20
Non-Small Cell Lung Cancer	NCI-H226		-5.19
Ovarian Cancer	IGROV1		-5.19
Renal Cancer	SN12C		-5.18
Prostate Cancer	DU-145		-5.18
Ovarian Cancer	NCI/ADR-RES		-5.17
Renal Cancer	786-0		-5.17
Renal Cancer	ACHN		-5.13
Non-Small Cell Lung Cancer	NCI-H23	*	-5.12
Melanoma	SK-MEL-2		-5.11
Breast Cancer	MDA-MB-231/ATCC	*	-5.10
Ovarian Cancer	OVCAR-3	*	-5.10
Breast Cancer	BT-549		-5.08
Ovarian Cancer	OVCAR-8		-5.08
Ovarian Cancer	OVCAR-5	*	-5.07
Non-Small Cell Lung Cancer	A549/ATCC		-5.02
Non-Small Cell Lung Cancer	NCI-H522	*	-4.99
CNS Cancer	SNB-19		-4.98
CNS Cancer	SNB-75		-4.97
Non-Small Cell Lung Cancer	NCI-H322M		-4.92
Non-Small Cell Lung Cancer	HOP-62		-4.90
Renal Cancer	TK-10		-4.90
Ovarian Cancer	SK-OV-3		-4.90
Renal Cancer	A498		-4.88

Log10 High Conc :-4.0

Figure S79: waterfall graph TGI of compound **11** (NSC 846172)

National Cancer Institute Developmental Therapeutics Program<br>Waterfall Graph TGI	NSC : D - 846172/1	Units :Molar	SSPL :1CYF	EXP. ID :2310NS93
	Report Date :November 29, 2023		Test Date :October 16, 2023	

Panel	Cell Name	Hollow Fiber	TGI
Melanoma	SK-MEL-5		-5.51
Melanoma	SK-MEL-28		-5.48
Colon Cancer	COLO 205	*	-5.48
Melanoma	UACC-257		-5.47
Melanoma	MALME-3M		-5.46
Melanoma	M14		-5.45
Colon Cancer	HCC-2998		-5.42
CNS Cancer	SF-295	*	-5.36
Melanoma	LOX IMVI	*	-5.29
Leukemia	RPMI-8226		-5.22
Melanoma	MDA-MB-435	*	-5.13
Renal Cancer	RXF 393		-5.13
Breast Cancer	MDA-MB-468		-5.10
Leukemia	K-562		-5.08
Breast Cancer	MCF7		-5.05
Melanoma	UACC-62	*	-5.01
Colon Cancer	HT29		-4.99
Leukemia	MOLT-4		-4.94
Leukemia	HL-60(TB)		-4.93
Non-Small Cell Lung Cancer	NCI-H460		-4.93
CNS Cancer	U251	*	-4.87
Colon Cancer	HCT-15		-4.85
Non-Small Cell Lung Cancer	HOP-92		-4.85
Colon Cancer	SW-620	*	-4.85
Renal Cancer	CAKI-1		-4.84
Colon Cancer	KM12		-4.81
Colon Cancer	HCT-116		-4.81
CNS Cancer	SF-539		-4.80
Prostate Cancer	PC-3		-4.80
Leukemia	CCRF-CEM		-4.79
Renal Cancer	UO-31		-4.75
Prostate Cancer	DU-145		-4.72
Breast Cancer	T-47D		-4.71
Non-Small Cell Lung Cancer	EKVX		-4.71
Renal Cancer	ACHN		-4.70
Ovarian Cancer	OVCAR-4		-4.70
CNS Cancer	SF-268		-4.70
Renal Cancer	SN12C		-4.69
Non-Small Cell Lung Cancer	NCI-H23	*	-4.68
Melanoma	SK-MEL-2		-4.68
Breast Cancer	MDA-MB-231/ATCC	*	-4.68
Ovarian Cancer	OVCAR-3	*	-4.68
Ovarian Cancer	OVCAR-5	*	-4.68
Breast Cancer	BT-549		-4.67
Ovarian Cancer	NCI/ADR-RES		-4.67
Breast Cancer	HS 578T		-4.67
Renal Cancer	786-0		-4.66
CNS Cancer	SNB-19		-4.65
Non-Small Cell Lung Cancer	NCI-H522	*	-4.64
Non-Small Cell Lung Cancer	NCI-H226		-4.63
CNS Cancer	SNB-75		-4.62
Non-Small Cell Lung Cancer	A549/ATCC		-4.62
Ovarian Cancer	IGROV1		-4.60
Ovarian Cancer	OVCAR-8		-4.60
Renal Cancer	TK-10		-4.59
Non-Small Cell Lung Cancer	NCI-H322M		-4.59
Ovarian Cancer	SK-OV-3		-4.59
Non-Small Cell Lung Cancer	HOP-62		-4.57
Renal Cancer	A498		-4.57
Leukemia	SR		> -4.00

Log10 High Conc : -4.0

Figure S80: waterfall graph LC_{50} of compound **11** (NSC 846172)

National Cancer Institute Developmental Therapeutics Program Waterfall Graph LC50	NSC : D - 846172/1	Units :Molar	SSPL :1CYF	EXP. ID :2310NS93
	Report Date :November 29, 2023		Test Date :October 16, 2023	

Panel	Cell Name	Hollow Fiber	LC50
Melanoma	SK-MEL-5		-5.25
Melanoma	SK-MEL-28		-5.21
Melanoma	MALME-3M		-5.19
Melanoma	UACC-257		-5.18
Colon Cancer	COLO 205	*	-5.17
Melanoma	M14		-5.16
Colon Cancer	HCC-2998		-5.08
CNS Cancer	SF-295	*	-5.03
Melanoma	LOX IMVI	*	-4.80
Breast Cancer	MDA-MB-468		-4.53
Melanoma	MDA-MB-435	*	-4.52
Renal Cancer	RXF 393		-4.51
Melanoma	UACC-62	*	-4.50
Colon Cancer	HCT-15		-4.42
Colon Cancer	HT29		-4.41
Breast Cancer	MCF7		-4.40
Non-Small Cell Lung Cancer	NCI-H460		-4.40
CNS Cancer	SF-539		-4.40
CNS Cancer	U251	*	-4.39
Colon Cancer	KM12		-4.38
Colon Cancer	SW-620	*	-4.36
Non-Small Cell Lung Cancer	HOP-92		-4.36
Renal Cancer	CAKI-1		-4.35
Renal Cancer	ACHN		-4.35
Prostate Cancer	PC-3		-4.35
Prostate Cancer	DU-145		-4.35
Non-Small Cell Lung Cancer	EKVX		-4.35
Leukemia	RPMI-8226		-4.34
Ovarian Cancer	OVCAR-5	*	-4.33
Renal Cancer	SN12C		-4.33
Non-Small Cell Lung Cancer	NCI-H23	*	-4.32
CNS Cancer	SNB-19		-4.32
Melanoma	SK-MEL-2		-4.32
Breast Cancer	MDA-MB-231/ATCC	*	-4.32
Breast Cancer	BT-549		-4.32
Ovarian Cancer	OVCAR-3	*	-4.32
Ovarian Cancer	OVCAR-4		-4.31
Renal Cancer	UO-31		-4.30
CNS Cancer	SF-268		-4.29
Renal Cancer	TK-10		-4.29
CNS Cancer	SNB-75		-4.28
Non-Small Cell Lung Cancer	NCI-H522	*	-4.28
Ovarian Cancer	NCI/ADR-RES		-4.28
Ovarian Cancer	SK-OV-3		-4.27
Leukemia	CCRF-CEM		-4.27
Leukemia	HL-60(TB)		-4.26
Renal Cancer	A498		-4.26
Renal Cancer	786-0		-4.26
Non-Small Cell Lung Cancer	NCI-H322M		-4.25
Non-Small Cell Lung Cancer	HOP-62		-4.25
Colon Cancer	HCT-116		-4.24
Non-Small Cell Lung Cancer	A549/ATCC		-4.24
Breast Cancer	T-47D		-4.20
Leukemia	MOLT-4		-4.18
Non-Small Cell Lung Cancer	NCI-H226		-4.17
Ovarian Cancer	OVCAR-8		-4.17
Breast Cancer	HS 578T		-4.15
Ovarian Cancer	IGROV1		-4.12
Leukemia	K-562		> -4.00
Leukemia	SR		> -4.00

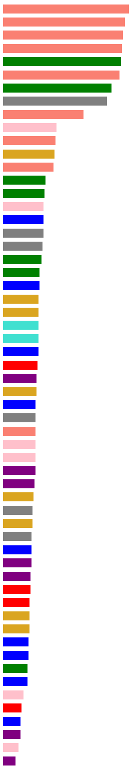

Log10 High Conc : -4.0

Figure S81: waterfall graph GI_{50} of compound **17** (NSC 846158)

National Cancer Institute Developmental Therapeutics Program Waterfall Graph GI50	NSC : D - 846158/1	Units :Molar	SSPL :0J6G	EXP. ID :2310NS93
	Report Date :September 01, 2025		Test Date :October 16, 2023	

Panel	Cell Name	Hollow Fiber	GI50
Renal Cancer	RXF 393		-5.59
Leukemia	K-562		-5.47
Non-Small Cell Lung Cancer	HOP-92		-5.46
Leukemia	SR		-5.45
Renal Cancer	UO-31		-5.41
Colon Cancer	HCC-2998		-5.39
Melanoma	MALME-3M		-5.37
Breast Cancer	T-47D		-5.33
Colon Cancer	COLO 205	*	-5.33
Leukemia	RPMI-8226		-5.31
Melanoma	SK-MEL-5		-5.30
Colon Cancer	HCT-116		-5.30
Breast Cancer	MDA-MB-468		-5.28
Prostate Cancer	PC-3		-5.28
Colon Cancer	HT29		-5.27
CNS Cancer	SF-295	*	-5.17
Melanoma	M14		-5.16
Leukemia	MOLT-4		-5.15
Breast Cancer	HS 578T		-5.06
Breast Cancer	BT-549		-5.06
Leukemia	CCRF-CEM		-5.01
Colon Cancer	HCT-15		-4.99
Leukemia	HL-60(TB)		-4.99
Ovarian Cancer	OVCAR-4		-4.99
Melanoma	SK-MEL-28		-4.96
Melanoma	UACC-62	*	-4.95
Melanoma	UACC-257		-4.95
Renal Cancer	SN12C		-4.94
Non-Small Cell Lung Cancer	NCI-H460		-4.94
Non-Small Cell Lung Cancer	EKVX		-4.94
Melanoma	LOX IMVI	*	-4.94
Breast Cancer	MCF7		-4.90
Renal Cancer	CAKI-1		-4.87
Melanoma	MDA-MB-435	*	-4.85
Ovarian Cancer	NCI/ADR-RES		-4.83
Colon Cancer	SW-620	*	-4.82
Melanoma	SK-MEL-2		-4.78
Ovarian Cancer	OVCAR-3	*	-4.77
Non-Small Cell Lung Cancer	NCI-H226		-4.76
Renal Cancer	ACHN		-4.74
CNS Cancer	SF-539		-4.73
CNS Cancer	SNB-75		-4.73
Colon Cancer	KM12		-4.72
Non-Small Cell Lung Cancer	NCI-H522	*	-4.71
Renal Cancer	786-0		-4.67
Non-Small Cell Lung Cancer	NCI-H23	*	-4.66
Ovarian Cancer	OVCAR-8		-4.59
Renal Cancer	TK-10		-4.56
CNS Cancer	U251	*	-4.53
CNS Cancer	SF-268		-4.48
Ovarian Cancer	IGROV1		-4.43
Ovarian Cancer	OVCAR-5	*	-4.43
Renal Cancer	A498		-4.35
Non-Small Cell Lung Cancer	A549/ATCC		-4.34
CNS Cancer	SNB-19		-4.28
Non-Small Cell Lung Cancer	HOP-62		> -4.00
Non-Small Cell Lung Cancer	NCI-H322M		> -4.00
Ovarian Cancer	SK-OV-3		> -4.00
Prostate Cancer	DU-145		> -4.00
Breast Cancer	MDA-MB-231/ATCC	*	> -4.00

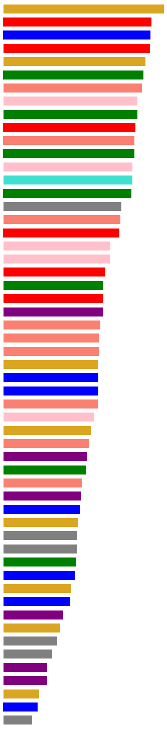

Log10 High Conc :-4.0

Figure S82: waterfall graph TGI of compound **17** (NSC 846158)

National Cancer Institute Developmental Therapeutics Program Waterfall Graph TGI	NSC : D - 846158/1	Units :Molar	SSPL :0J6G	EXP. ID :2310NS93
	Report Date :September 01, 2025		Test Date :October 16, 2023	

Panel	Cell Name	Hollow Fiber	TGI
Colon Cancer	HCC-2998		-4.80
Melanoma	SK-MEL-5		-4.77
Colon Cancer	COLO 205	*	-4.74
Melanoma	MALME-3M		-4.69
Melanoma	LOX IMVI	*	-4.59
Non-Small Cell Lung Cancer	HOP-92		-4.55
Breast Cancer	MCF7		-4.53
Leukemia	K-562		-4.52
Melanoma	UACC-257		-4.44
Melanoma	SK-MEL-28		-4.42
Melanoma	SK-MEL-2		-4.42
Leukemia	HL-60(TB)		-4.37
Leukemia	RPMI-8226		-4.31
Renal Cancer	RXF 393		-4.31
Breast Cancer	MDA-MB-468		-4.27
Breast Cancer	BT-549		-4.24
Melanoma	MDA-MB-435	*	-4.23
CNS Cancer	SF-295	*	-4.21
Melanoma	M14		-4.20
Melanoma	UACC-62	*	-4.13
Colon Cancer	SW-620	*	-4.10
CNS Cancer	SF-539		-4.10
Non-Small Cell Lung Cancer	NCI-H522	*	-4.07
Leukemia	CCRF-CEM		> -4.00
Leukemia	MOLT-4		> -4.00
Leukemia	SR		> -4.00
Non-Small Cell Lung Cancer	A549/ATCC		> -4.00
Non-Small Cell Lung Cancer	EKVX		> -4.00
Non-Small Cell Lung Cancer	HOP-62		> -4.00
Non-Small Cell Lung Cancer	NCI-H226		> -4.00
Non-Small Cell Lung Cancer	NCI-H23	*	> -4.00
Non-Small Cell Lung Cancer	NCI-H322M		> -4.00
Non-Small Cell Lung Cancer	NCI-H460		> -4.00
Colon Cancer	HCT-116		> -4.00
Colon Cancer	HCT-15		> -4.00
Colon Cancer	HT29		> -4.00
Colon Cancer	KM12		> -4.00
CNS Cancer	SF-268		> -4.00
CNS Cancer	SNB-19		> -4.00
CNS Cancer	SNB-75		> -4.00
CNS Cancer	U251	*	> -4.00
Ovarian Cancer	IGROV1		> -4.00
Ovarian Cancer	OVCAR-3	*	> -4.00
Ovarian Cancer	OVCAR-4		> -4.00
Ovarian Cancer	OVCAR-5	*	> -4.00
Ovarian Cancer	OVCAR-8		> -4.00
Ovarian Cancer	NCI/ADR-RES		> -4.00
Ovarian Cancer	SK-OV-3		> -4.00
Renal Cancer	786-0		> -4.00
Renal Cancer	A498		> -4.00
Renal Cancer	ACHN		> -4.00
Renal Cancer	CAKI-1		> -4.00
Renal Cancer	SN12C		> -4.00
Renal Cancer	TK-10		> -4.00
Renal Cancer	UO-31		> -4.00
Prostate Cancer	PC-3		> -4.00
Prostate Cancer	DU-145		> -4.00
Breast Cancer	MDA-MB-231/ATCC	*	> -4.00
Breast Cancer	HS 578T		> -4.00
Breast Cancer	T-47D		> -4.00

Log10 High Conc : -4.0

Figure S83: waterfall graph LC_{50} of compound **17** (NSC 846158)

National Cancer Institute Developmental Therapeutics Program Waterfall Graph LC50	NSC : D - 846158/1	Units :Molar	SSPL :0J6G	EXP. ID :2310NS93
	Report Date :September 01, 2025		Test Date :October 16, 2023	

Panel	Cell Name	Hollow Fiber	LC50
Melanoma	SK-MEL-5		-4.38
Colon Cancer	HCC-2998		-4.33
Colon Cancer	COLO 205	*	-4.29
Melanoma	LOX IMVI	*	-4.24
Breast Cancer	MCF7		-4.17
Melanoma	MALME-3M		-4.15
Melanoma	SK-MEL-2		-4.07
Leukemia	CCRF-CEM		> -4.00
Leukemia	HL-60(TB)		> -4.00
Leukemia	K-562		> -4.00
Leukemia	MOLT-4		> -4.00
Leukemia	RPMI-8226		> -4.00
Leukemia	SR		> -4.00
Non-Small Cell Lung Cancer	A549/ATCC		> -4.00
Non-Small Cell Lung Cancer	EKVX		> -4.00
Non-Small Cell Lung Cancer	HOP-62		> -4.00
Non-Small Cell Lung Cancer	HOP-92		> -4.00
Non-Small Cell Lung Cancer	NCI-H226		> -4.00
Non-Small Cell Lung Cancer	NCI-H23	*	> -4.00
Non-Small Cell Lung Cancer	NCI-H322M		> -4.00
Non-Small Cell Lung Cancer	NCI-H460		> -4.00
Non-Small Cell Lung Cancer	NCI-H522	*	> -4.00
Colon Cancer	HCT-116		> -4.00
Colon Cancer	HCT-15		> -4.00
Colon Cancer	HT29		> -4.00
Colon Cancer	KM12		> -4.00
Colon Cancer	SW-620	*	> -4.00
CNS Cancer	SF-268		> -4.00
CNS Cancer	SF-295	*	> -4.00
CNS Cancer	SF-539		> -4.00
CNS Cancer	SNB-19		> -4.00
CNS Cancer	SNB-75		> -4.00
CNS Cancer	U251	*	> -4.00
Melanoma	M14		> -4.00
Melanoma	MDA-MB-435	*	> -4.00
Melanoma	SK-MEL-28		> -4.00
Melanoma	UACC-257		> -4.00
Melanoma	UACC-62	*	> -4.00
Ovarian Cancer	IGROV1		> -4.00
Ovarian Cancer	OVCAR-3	*	> -4.00
Ovarian Cancer	OVCAR-4		> -4.00
Ovarian Cancer	OVCAR-5	*	> -4.00
Ovarian Cancer	OVCAR-8		> -4.00
Ovarian Cancer	NCI/ADR-RES		> -4.00
Ovarian Cancer	SK-OV-3		> -4.00
Renal Cancer	786-0		> -4.00
Renal Cancer	A498		> -4.00
Renal Cancer	ACHN		> -4.00
Renal Cancer	CAKI-1		> -4.00
Renal Cancer	RXF 393		> -4.00
Renal Cancer	SN12C		> -4.00
Renal Cancer	TK-10		> -4.00
Renal Cancer	UO-31		> -4.00
Prostate Cancer	PC-3		> -4.00
Prostate Cancer	DU-145		> -4.00
Breast Cancer	MDA-MB-231/ATCC	*	> -4.00
Breast Cancer	HS 578T		> -4.00
Breast Cancer	BT-549		> -4.00
Breast Cancer	T-47D		> -4.00
Breast Cancer	MDA-MB-468		> -4.00

Log10 High Conc : -4.0

Figure S84: waterfall graph GI_{50} of compound **20** (NSC 846160)

National Cancer Institute Developmental Therapeutics Program Waterfall Graph GI50	NSC : D - 846160/1	Units :Molar	SSPL :0J6G	EXP. ID :2310NS93
	Report Date :December 01, 2023		Test Date :October 16, 2023	

Panel	Cell Name	Hollow Fiber	GI50
Leukemia	SR		-6.27
Colon Cancer	HCC-2998		-5.75
Melanoma	SK-MEL-5		-5.75
Melanoma	UACC-257		-5.73
Colon Cancer	COLO 205	*	-5.71
Melanoma	M14		-5.70
Melanoma	MALME-3M		-5.69
Melanoma	SK-MEL-28		-5.68
Leukemia	K-562		-5.53
Colon Cancer	SW-620	*	-5.44
Leukemia	RPMI-8226		-5.43
Renal Cancer	UO-31		-5.43
Non-Small Cell Lung Cancer	HOP-92		-5.42
Colon Cancer	HT29		-5.39
Melanoma	LOX IMVI	*	-5.36
Breast Cancer	T-47D		-5.35
Breast Cancer	MDA-MB-468		-5.33
Leukemia	MOLT-4		-5.32
Renal Cancer	RXF 393		-5.32
Breast Cancer	HS 578T		-5.31
Melanoma	MDA-MB-435	*	-5.31
Leukemia	HL-60(TB)		-5.30
Colon Cancer	HCT-116		-5.26
Renal Cancer	CAKI-1		-5.20
Prostate Cancer	PC-3		-5.18
Melanoma	UACC-62	*	-5.16
Colon Cancer	HCT-15		-5.11
Non-Small Cell Lung Cancer	NCI-H460		-5.09
Breast Cancer	MCF7		-5.05
Breast Cancer	BT-549		-5.04
Colon Cancer	KM12		-5.03
CNS Cancer	U251	*	-4.99
Ovarian Cancer	IGROV1		-4.98
CNS Cancer	SF-539		-4.98
Non-Small Cell Lung Cancer	EKVX		-4.97
Ovarian Cancer	OVCAR-4		-4.97
Leukemia	CCRF-CEM		-4.97
Non-Small Cell Lung Cancer	NCI-H23	*	-4.96
Renal Cancer	786-0		-4.95
Renal Cancer	SN12C		-4.94
Renal Cancer	ACHN		-4.94
CNS Cancer	SF-295	*	-4.94
Non-Small Cell Lung Cancer	NCI-H322M		-4.93
CNS Cancer	SF-268		-4.92
Non-Small Cell Lung Cancer	NCI-H226		-4.91
Ovarian Cancer	OVCAR-3	*	-4.91
Ovarian Cancer	OVCAR-5	*	-4.91
Ovarian Cancer	NCI/ADR-RES		-4.91
Ovarian Cancer	SK-OV-3		-4.90
Non-Small Cell Lung Cancer	NCI-H522	*	-4.89
Non-Small Cell Lung Cancer	A549/ATCC		-4.87
Ovarian Cancer	OVCAR-8		-4.87
Breast Cancer	MDA-MB-231/ATCC	*	-4.87
Melanoma	SK-MEL-2		-4.86
Prostate Cancer	DU-145		-4.86
CNS Cancer	SNB-75		-4.84
CNS Cancer	SNB-19		-4.84
Non-Small Cell Lung Cancer	HOP-62		-4.82
Renal Cancer	TK-10		-4.79
Renal Cancer	A498		-4.79

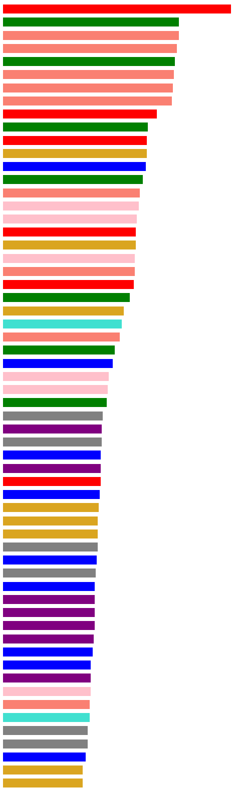

Log10 High Conc :-4.0

Figure S85: waterfall graph TGI of compound **20** (NSC 846160)

National Cancer Institute Developmental Therapeutics Program Waterfall Graph TGI	NSC : D - 846160/1	Units :Molar	SSPL :0J6G	EXP. ID :2310NS93
	Report Date :December 01, 2023		Test Date :October 16, 2023	

Panel	Cell Name	Hollow Fiber	TGI
Melanoma	SK-MEL-5		-5.47
Colon Cancer	HCC-2998		-5.46
Colon Cancer	COLO 205	*	-5.42
Melanoma	MALME-3M		-5.40
Melanoma	M14		-5.38
Melanoma	SK-MEL-28		-5.31
Melanoma	UACC-257		-5.18
Colon Cancer	SW-620	*	-4.86
Colon Cancer	HT29		-4.83
Melanoma	LOX IMVI	*	-4.80
Renal Cancer	UO-31		-4.77
Melanoma	MDA-MB-435	*	-4.75
Breast Cancer	MDA-MB-468		-4.75
Non-Small Cell Lung Cancer	HOP-92		-4.75
Renal Cancer	RXF 393		-4.74
Renal Cancer	CAKI-1		-4.70
Melanoma	UACC-62	*	-4.70
Breast Cancer	T-47D		-4.68
Colon Cancer	HCT-116		-4.68
Colon Cancer	HCT-15		-4.67
Breast Cancer	BT-549		-4.65
Colon Cancer	KM12		-4.65
CNS Cancer	SF-539		-4.65
CNS Cancer	U251	*	-4.65
Breast Cancer	MCF7		-4.64
Non-Small Cell Lung Cancer	NCI-H460		-4.64
Non-Small Cell Lung Cancer	EKVX		-4.63
Non-Small Cell Lung Cancer	NCI-H23	*	-4.63
Renal Cancer	ACHN		-4.63
Ovarian Cancer	IGROV1		-4.62
CNS Cancer	SF-295	*	-4.62
Renal Cancer	SN12C		-4.62
Non-Small Cell Lung Cancer	NCI-H322M		-4.61
Ovarian Cancer	OVCAR-4		-4.61
Ovarian Cancer	OVCAR-5	*	-4.61
Ovarian Cancer	OVCAR-3	*	-4.60
Breast Cancer	HS 578T		-4.59
Renal Cancer	786-0		-4.59
Ovarian Cancer	SK-OV-3		-4.58
CNS Cancer	SF-268		-4.58
Non-Small Cell Lung Cancer	NCI-H522	*	-4.57
Melanoma	SK-MEL-2		-4.57
Breast Cancer	MDA-MB-231/ATCC	*	-4.56
Ovarian Cancer	NCI/ADR-RES		-4.56
CNS Cancer	SNB-19		-4.55
Non-Small Cell Lung Cancer	A549/ATCC		-4.54
CNS Cancer	SNB-75		-4.54
Prostate Cancer	DU-145		-4.54
Leukemia	HL-60(TB)		-4.54
Leukemia	K-562		-4.54
Non-Small Cell Lung Cancer	HOP-62		-4.52
Non-Small Cell Lung Cancer	NCI-H226		-4.52
Renal Cancer	TK-10		-4.52
Ovarian Cancer	OVCAR-8		-4.52
Renal Cancer	A498		-4.51
Prostate Cancer	PC-3		-4.50
Leukemia	MOLT-4		-4.34
Leukemia	RPMI-8226		-4.27
Leukemia	CCRF-CEM		-4.05
Leukemia	SR		> -4.00

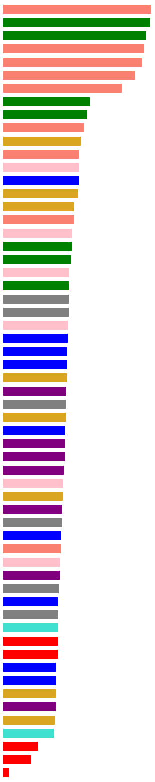

Log10 High Conc : -4.0

Figure S86: waterfall graph LC_{50} of compound **20** (NSC 846160)

National Cancer Institute Developmental Therapeutics Program Waterfall Graph LC50	NSC : D - 846160/1	Units :Molar	SSPL :0J6G	EXP. ID :2310NS93
	Report Date :December 01, 2023		Test Date :October 16, 2023	

Panel	Cell Name	Hollow Fiber	LC50
Melanoma	SK-MEL-5		-5.19
Colon Cancer	HCC-2998		-5.18
Colon Cancer	COLO 205	*	-5.14
Melanoma	MALME-3M		-5.11
Melanoma	M14		-5.06
Melanoma	SK-MEL-28		-4.88
Melanoma	UACC-257		-4.57
Melanoma	LOX IMVI	*	-4.39
Colon Cancer	HT29		-4.36
Renal Cancer	UO-31		-4.35
Melanoma	UACC-62	*	-4.34
Renal Cancer	RXF 393		-4.33
Breast Cancer	MDA-MB-468		-4.32
CNS Cancer	SF-539		-4.32
Renal Cancer	CAKI-1		-4.32
Colon Cancer	HCT-15		-4.31
Melanoma	MDA-MB-435	*	-4.31
Renal Cancer	ACHN		-4.31
CNS Cancer	SF-295	*	-4.30
CNS Cancer	U251	*	-4.30
Ovarian Cancer	OVCAR-5	*	-4.30
Non-Small Cell Lung Cancer	NCI-H23	*	-4.29
Ovarian Cancer	OVCAR-3	*	-4.29
Non-Small Cell Lung Cancer	NCI-H322M		-4.29
Breast Cancer	BT-549		-4.29
Colon Cancer	KM12		-4.29
Renal Cancer	SN12C		-4.29
Non-Small Cell Lung Cancer	EKVX		-4.28
Breast Cancer	MCF7		-4.27
Melanoma	SK-MEL-2		-4.27
CNS Cancer	SNB-19		-4.27
Ovarian Cancer	SK-OV-3		-4.26
Colon Cancer	SW-620	*	-4.26
Non-Small Cell Lung Cancer	NCI-H522	*	-4.26
Non-Small Cell Lung Cancer	HOP-92		-4.26
Ovarian Cancer	IGROV1		-4.26
Breast Cancer	MDA-MB-231/ATCC	*	-4.26
Ovarian Cancer	OVCAR-4		-4.25
Renal Cancer	TK-10		-4.25
CNS Cancer	SNB-75		-4.25
Renal Cancer	A498		-4.24
CNS Cancer	SF-268		-4.24
Non-Small Cell Lung Cancer	NCI-H460		-4.23
Non-Small Cell Lung Cancer	HOP-62		-4.23
Renal Cancer	786-0		-4.22
Prostate Cancer	DU-145		-4.22
Non-Small Cell Lung Cancer	A549/ATCC		-4.21
Ovarian Cancer	NCI/ADR-RES		-4.21
Colon Cancer	HCT-116		-4.19
Ovarian Cancer	OVCAR-8		-4.16
Breast Cancer	T-47D		-4.13
Non-Small Cell Lung Cancer	NCI-H226		-4.13
Leukemia	CCRF-CEM		> -4.00
Leukemia	HL-60(TB)		> -4.00
Leukemia	K-562		> -4.00
Leukemia	MOLT-4		> -4.00
Leukemia	RPMI-8226		> -4.00
Leukemia	SR		> -4.00
Prostate Cancer	PC-3		> -4.00
Breast Cancer	HS 578T		> -4.00

Log10 High Conc : -4.0

Figure S87: preliminary ZIKV/Huh-7 antiviral screening results for compounds **1–20**

Compd	Structure	n	m	Ar	ZIKV/Huh-7		
					CC_{50}[b]	EC_{50}[b]	SI
**1**	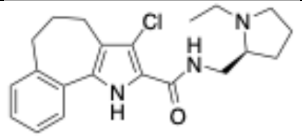	-	-	-	3.88	2.07	1.87
**2**	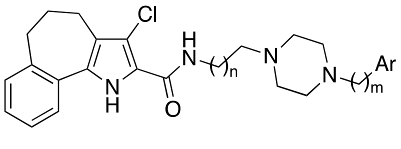	1	-	Ph	<50	43.91	<1.14
**3**		1	-	4-Cl-Ph	62.63	3.24	19.31
**4**		1	-	2-Cl-Ph	59.88	4.39	13.63
**5**		1	-	2-OCH_3-Ph	24.31	3.82	6.37
**6**		1	-	3-OCH_3-Ph	53.48	3.24	16.49
**7**		1	-	2-OC_2H_5-Ph	50.74	3.82	13.29
**8**		1	-	2-CN-Ph	50.56	2.87	17.62
**9**		1	-	3,4-Cl-Ph	65.46	3.24	20.19
**10**[a]		2	-	Ph	12.42	3.59	3.46
**11**[a]		2	1	Ph	4.89	3.49	1.40
**12**	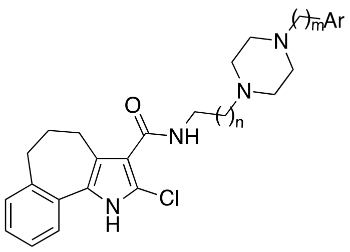	1	-	Ph	34.17	1.54	22.20
**13**		1	-	4-Cl-Ph	57.87	1.30	44.58
**14**		1	-	2-Cl-Ph	30.15	4.74	6.37
**15**		1	-	2-OCH_3-Ph	9.43	4.74	1.99
**16**		1	-	3-OCH_3-Ph	225.90	0.00	-
**17**		1	-	2-OC_2H_5-Ph	16.35	4.46	3.67
**18**		1	-	2-CN-Ph	15.51	4.11	3.77
**19**		1	-	3,4-Cl-Ph	12.02	4.08	2.95
**20**		2	1	Ph	6.23	4.74	1.32
**Sofosbuvir**					300.10	2.07	144.98

[a]Compounds **10** and **11** do not contain the chlorine atom on the pyrrole ring; [b]μM.

Figure S88: preliminary IAV/MDCK antiviral screening results for compounds **1–20**

**Compd**	**Structure**	**n**	**m**	**Ar**	**IAV/MDCK**		
					**CC_{50}[b]**	**EC_{50}[b]**	**SI**
**1**	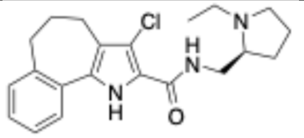	-	-	-	1.75	17.73	0.10
**2**	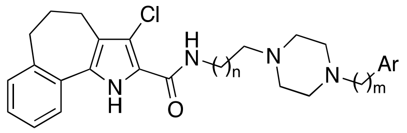	1	-	Ph	0.00	0.00	-
**3**		1	-	4-Cl-Ph	1347.00	0.00	-
**4**		1	-	2-Cl-Ph	14.71	0.00	-
**5**		1	-	2-OCH_3-Ph	10.99	0.00	-
**6**		1	-	3-OCH_3-Ph	66.00	0.00	-
**7**		1	-	2-OC_2H_5-Ph	5.60	0.00	-
**8**		1	-	2-CN-Ph	119.00	0.00	-
**9**		1	-	3,4-Cl-Ph	427.40	0.00	-
**10***		2	-	Ph	67.81	0.00	-
**11***		2	1	Ph	5.38	17.73	0.30
**12**	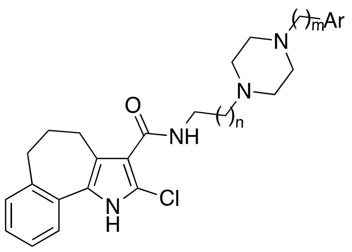	1	-	Ph	32.06	3.49	9.18
**13**		1	-	4-Cl-Ph	225.00	41.27	5.45
**14**		1	-	2-Cl-Ph	15.63	0.00	-
**15**		1	-	2-OCH_3-Ph	8.29	17.73	0.47
**16**		1	-	3-OCH_3-Ph	42.92	0.00	-
**17**		1	-	2-OC_2H_5-Ph	8.13	34.93	0.23
**18**		1	-	2-CN-Ph	13.91	49.38	0.28
**19**		1	-	3,4-Cl-Ph	<50	4.11	<12.17
**20**		2	1	Ph	6.12	17.73	0.35
**EIDD-1931**					<50	2.07	<24.15

[a]Compounds **10** and **11** do not contain the chlorine atom on the pyrrole ring; [b]µM.

Figure S89: preliminary SARS-CoV-2/Vero TMPRSS antiviral screening results for compounds **1–20**

Compd	Structure	n	m	Ar	SARS-CoV-2/Vero TMPRSS		
					CC_{50}[b]	EC_{50}[b]	SI
**1**	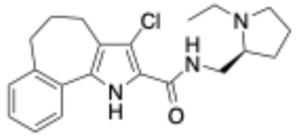	-	-	-	14.69	9.61	1.54
**2**	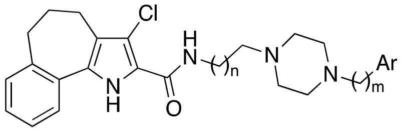	1	-	Ph	362.00	0.00	-
**3**		1	-	4-Cl-Ph	143.20	0.00	-
**4**		1	-	2-Cl-Ph	57.94	42.45	1.36
**5**		1	-	2-OCH_3-Ph	29.37	34.93	0.84
**6**		1	-	3-OCH_3-Ph	120.60	0.00	-
**7**		1	-	2-OC_2H_5-Ph	38.45	37.29	1.03
**8**		1	-	2-CN-Ph	67.53	58.42	1.16
**9**		1	-	3,4-Cl-Ph	67.73	0.00	-
**10***		2	-	Ph	55.72	0.00	-
**11***		2	1	Ph	15.44	17.73	0.87
**12**	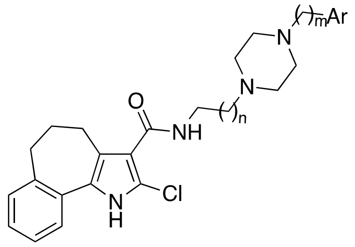	1	-	Ph	72.82	2.26	32.22
**13**		1	-	4-Cl-Ph	77.18	>0.5	<154.36
**14**		1	-	2-Cl-Ph	36.69	0.00	-
**15**		1	-	2-OCH_3-Ph	38.03	40.63	0.94
**16**		1	-	3-OCH_3-Ph	50.24	0.00	-
**17**		1	-	2-OC_2H_5-Ph	37.24	0.00	-
**18**		1	-	2-CN-Ph	35.01	0.00	-
**19**		1	-	3,4-Cl-Ph	40.76	0-00	-
**20**		2	1	Ph	15.40	17.73	0.87
**EIDD-1931**					13.38	0.11	121.64

[a]Compounds **10** and **11** do not contain the chlorine atom on the pyrrole ring; [b]μM.
